# Stimulating T cell responses against patient-derived breast cancer cells with neoantigen peptide-loaded peripheral blood mononuclear cells

**DOI:** 10.1007/s00262-024-03627-3

**Published:** 2024-02-13

**Authors:** Natthaporn Sueangoen, Harald Grove, Nisa Chuangchot, Jaturawitt Prasopsiri, Thanyada Rungrotmongkol, Kamonpan Sanachai, Nitchakan Darai, Suyanee Thongchot, Prapat Suriyaphol, Doonyapat Sa-Nguanraksa, Peti Thuwajit, Pa-thai Yenchitsomanus, Chanitra Thuwajit

**Affiliations:** 1 Graduate Program in Immunology, Faculty of Medicine Siriraj Hospital, Mahidol University, Bangkok, Thailand; 2 Department of Immunology, Faculty of Medicine Siriraj Hospital, Mahidol University, Bangkok, Thailand; 3 Division of Bioinformatics and Data Management for Research, Research Group and Research Network Division, Faculty of Medicine Siriraj Hospital, Mahidol University, Bangkok, Thailand; 4 Siriraj Center of Research Excellence for Cancer Immunotherapy (SiCORE-CIT), Research Department, Faculty of Medicine Siriraj Hospital, Mahidol University, Bangkok, Thailand; 5 Center of Excellence in Biocatalyst and Sustainable Biotechnology, Department of Biochemistry, Faculty of Science, Chulalongkorn University, Bangkok, Thailand; 6 Program in Bioinformatics and Computational Biology, Graduate School, Chulalongkorn University, Bangkok, Thailand; 7 Department of Biochemistry, Faculty of Science, Khon Kaen University, Khon Kaen, Thailand; 8 ASEAN Institute for Health Development, Mahidol University, Nakon Pathom, Thailand; 9 Division of Head Neck and Breast Surgery, Department of Surgery, Faculty of Medicine Siriraj Hospital, Mahidol University, Bangkok, Thailand

**Keywords:** Breast cancer, Neoantigen peptide, pVAC-Seq pipeline, Whole-genome sequence, Whole transcriptomic sequence

## Abstract

Breast cancer stands as a formidable global health challenge for women. While neoantigens exhibit efficacy in activating T cells specific to cancer and instigating anti-tumor immune responses, the accuracy of neoantigen prediction remains suboptimal. In this study, we identified neoantigens from the patient-derived breast cancer cells, PC-B-142CA and PC-B-148CA cells, utilizing whole-genome and RNA sequencing. The pVAC-Seq pipeline was employed, with minor modification incorporating criteria (1) binding affinity of mutant (MT) peptide with HLA (IC_50_ MT) ≤ 500 nm in 3 of 5 algorithms and (2) IC_50_ wild type (WT)/MT > 1. Sequencing results unveiled 2513 and 3490 somatic mutations, and 646 and 652 non-synonymous mutations in PC-B-142CA and PC-B-148CA, respectively. We selected the top 3 neoantigens to perform molecular dynamic simulation and synthesized 9–12 amino acid neoantigen peptides, which were then pulsed onto healthy donor peripheral blood mononuclear cells (PBMCs). Results demonstrated that T cells activated by ADGRL1^E274K^, PARP1^E619K^, and SEC14L2^R43Q^ peptides identified from PC-B-142CA exhibited significantly increased production of interferon-gamma (IFN-*γ*), while PARP1^E619K^ and SEC14L2^R43Q^ peptides induced the expression of CD107a on T cells. The % tumor cell lysis was notably enhanced by T cells activated with MT peptides across all three healthy donors. Moreover, ALKBH6^V83M^ and GAA^I823T^ peptides from PC-B-148CA remarkably stimulated IFN-*γ*- and CD107a-positive T cells, displaying high cell-killing activity against target cancer cells. In summary, our findings underscore the successful identification of neoantigens with anti-tumor T cell functions and highlight the potential of personalized neoantigens as a promising avenue for breast cancer treatment.

**Supplementary Information:**

The online version contains supplementary material available at 10.1007/s00262-024-03627-3.

## Introduction

Breast cancer (BCA) is the first rank female cancer worldwide [1] with a 15.5% mortality rate [2]. Although the treatment of breast cancer has been developed, in particular, targeted therapy with a successful response [3], more patients develop acquired resistance and progress to exhibit metastatic disease [4]. Moreover, the standard treatments for aggressive breast cancer are still limited due to a lack of estrogen receptor, progesterone receptor, and human epidermal growth factor receptor-2 (HER-2) expressions [5]. Therefore, the development of alternative treatment approaches becomes challenging.

Immunotherapy is the new hope for advanced stages and drug-resistant cancers [6]. The analysis of cancer genomes has discovered diverse tumor mutational landscapes in patients [7], and this tumor mutational burden is the prognostic marker for immunotherapeutic response [8]. The effective activation of the host immune response against cancer cells is determined by whether the cancer-specific antigens are identified [9]. The somatic gene mutation-encoding mutant (MT) peptides or neoantigens found in cancer cells but not existing in normal tissues have been revealed as effective antigens to activate cancer-specific T cells resulting in eliciting anti-tumor immunity [10].

Advances in next-generation sequencing technologies and several computational tools that are accessible for neoantigen identification have been reviewed to benefit the creation of personalized neoantigen vaccines that improve patient outcomes in various cancer types [11]. Zhang et al. successfully identified and validated candidate neoantigens for immune targeting in advanced BCA [12]. Neoantigen prediction algorithms mostly rely on the binding affinity value (IC_50_) of mutant peptides and human leukocyte antigen (HLA) compared with corresponding wildtype (WT) peptides and lack the actual binding process. Thus, optimizing neoantigen identification and validating the prioritized candidate neoantigens are required to enhance neoantigen-specific T cell responses.

Herein, neoantigens were identified in two primary cancer cells derived from in-house patient-derived primary breast cancer cells [13]. The prioritized candidate neoantigens determined the actual binding with the HLA class I allele restricted to the patient by molecular dynamic (MD) simulation. These neoantigen short peptides could activate neoantigen-specific T cells from healthy donor peripheral blood mononuclear cells (PBMCs) significantly more than that of the corresponding normal peptides ensured by increasing interferon-gamma (IFN-*γ*) and degranulation marker, CD107a. Importantly, these neoantigen-specific T cells could destroy patient-derived cancer cells harboring these neoantigens. Taken all, the findings herein highlight the successful neoantigens identified in BCA patients for T cell activation leading to the elimination of patient-derived cancer cells.

## Materials and methods

### Breast cancer cell lines and culture

The PC-B-142CA (HER2^+^ subtype) and PC-B-148CA (triple-negative subtype) BCA cells were derived from the cancer tissues of two patients admitted at the Faculty of Medicine Siriraj Hospital, Mahidol University [13]. The cells were maintained in DMEM/F12 medium (Gibco, ThermoFisher Scientific, Waltham, MA) supplemented with 10% fetal bovine serum (FBS) and 1 U/ml penicillin G sodium and 1 mg/ml streptomycin (ThermoFissher Scientific, Waltham, MA) at 37 °C in humidified 5% CO_2_ incubator.

### White blood cells (WBCs) and peripheral blood mononuclear cells (PBMCs) collection

The WBCs of BCA patients were extracted and used for DNA sequencing. The obtained DNA sequences were used as normal sequence references to identify the non-synonymous mutation (NSM) of PC-B-142CA and PC-B-148CA cells. PBMCs from healthy donors partially HLA-matched to these two cells were used to activate by neoantigen peptides for investigation of the neoantigen-activated T cell response. This process was approved by SIRB (COA no. Si 776/2019). All donors signed the informed consent forms.

### Whole-genome sequencing (WGS) and whole-transcriptome sequencing (WTS)

**Fig. 1:**
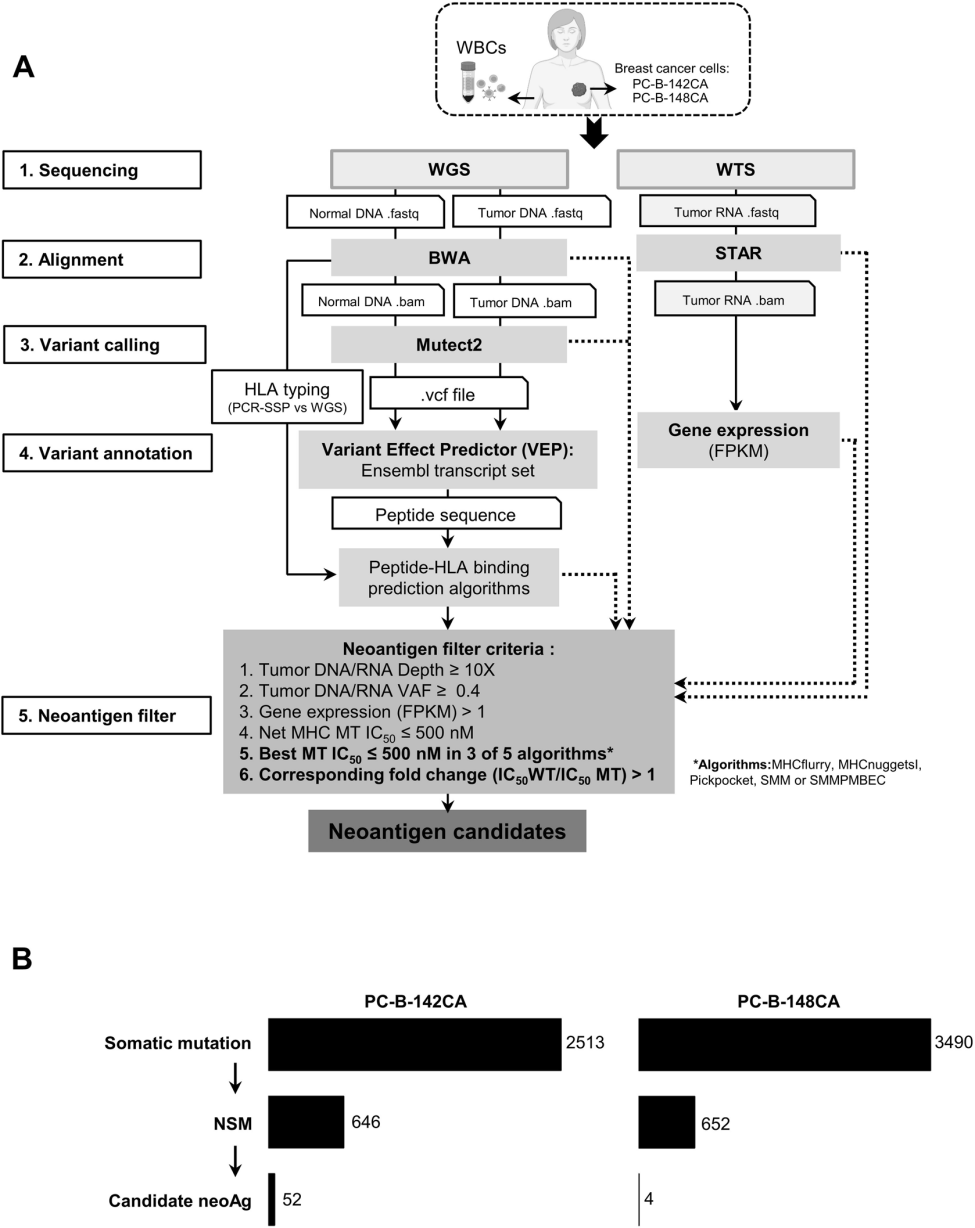
Neoantigen identification in BCA patients. **a**. A workflow of neoantigen identification using modified pVAC-Seq pipeline. WGS and WTS were performed in the two in-house established BCA cells (PC-B-142CA and PC-B-148CA) and matched the WBC of each patient to identify candidate neoantigen by filtering NSMs by neoantigen filter criteria. **b**. Number of mutations and candidate neoantigens in PC-B-142CA and PC-B-148CA BCA cells

Both genomic DNA and total RNA were extracted from PC-B-142CA and PC-B-148CA cells using an AllPrep DNA/RNA mini kit (Qiagen, Hilden, Germany), whereas germline control DNA was extracted from the patient’s WBCs (Fig. 1a). Genome libraries were prepared using TruSeq DNA Nano kit (Illumina, San Diego, CA), and sequenced on Illumina NovaSeq-6000 sequencer to generate 150 PE reads (Macrogen®, Seoul, South Korea). RNA sequence libraries were prepared using TruSeq Stranded mRNA Library Prep kit (Illumina) and sequenced as 100-bp paired-end reads using NovaSeq 6000 as a service of Macrogen®.

### Identification of neoantigens

DNA sequence reads were aligned to the human reference genome (GRCh38/hg38) using BWA-mem (v0.7.15). Duplicated reads were marked and removed using the Picard tool. Somatic single-nucleotide variants (SNVs) were called using MuTect2 (v2.1) from the GATK package (v4.1.5). A matched normal sample was used as part of the somatic calling. The SNVs were annotated for NSM by variant effect predictor (VEP) with the Ensembl transcript set. Transcriptome sequence reads were processed using FASTQC for sequencing base quality control and aligned to the (GRCh38/hg38) using Spliced Transcripts Alignment to a Reference (STAR) tool. The number of reads that aligned to each gene was used to calculate gene expression as fragments per kilobase of transcript per million mapped reads (FPKM) (Fig. 1a).

For binding affinity prediction of neoantigen candidates, the HLA alleles of each patient were determined by PCR-sequence-specific primers at the Department of Transfusion Medicine Siriraj Hospital, Mahidol University. The 8- to 12-mer peptide sequences that contained the MT peptide and corresponding WT peptide sequence were predicted for binding affinities with HLA class I matched to the patient using MHCflurry [14], MHCnuggetsI [15], NetMHC [16], SMM [17], SMMPMBEC [18], and Pickpocket [19].

### Neoantigens filtering

Neoantigen candidates were predicted using Personalized variant antigens by cancer sequencing (pVAC-Seq) pipeline [20] with some modifications including: (1) tumor DNA/RNA depth ≥ 10*x*, (2) tumor DNA/RNA variant allele fraction (VAF) ≥ 0.4, (3) FPKM > 1, (4) NetMHC of MT peptide (IC_50_) ≤ 500 nM, (5) IC_50_ MT ≤ 500 in 3 of 5 algorithms including MHCflurry, MHCnuggetsI, SMM, SMMPMBEC, and Pickpocket, and (6) Corresponding fold change (IC_50_ WT/ MT) > 1 (Fig. 1a).

### Neoepitope peptide-HLA class I binding by molecular dynamics (MD) simulation

The initial atomic coordinates were built from the HLA-A*11:01 X-ray crystal structure complex with SSCSSCPLSK positive peptide (10 amino acids) (Protein Data Bank (PDB) ID: 5GRD) [21], HLA-A*24:02 complex with TYQWIIRNW positive peptide (9 amino acids) (PDB ID: 7JYW) and TYQWIIRNWET positive peptide (11 amino acids) (PDB ID: 7JYX) [22], whereas the HLA-C*07:02 complex with RYRPGTVAL positive peptide (9 amino acids) (PDB ID: 5VGE) [23]. The amino acid side chains of the positive peptides within HLA were modified to the neoantigen peptide by the Discovery Studio program (BIOVIA, Dassault Systèmes, Discovery Studio Visualizer Software, V21.1.0.20298, 2020). The missing hydrogen atoms of the HLA protein were added with the help of the LEaP module in the Assisted Model Building with Energy Refinement (AMBER) version 2020 [24]. The AMBER ff14SB [25] was taken for both the HLA and peptides. The complex geometries with the added hydrogen atoms were minimized with 1000 steps of the steepest descent method and subsequently by 3000 steps of conjugated gradient [26]. The solvation of each system was performed by the transferable intermolecular potential 3P water molecules in a periodic box. The systems were neutralized using Cl^−^ or Na^+^ counter ions. The water molecules were minimized only with 1000 steps of steepest descents and continued by 3000 steps of the conjugate gradient. Lastly, all systems were fully minimized by the same minimization process. The MD simulations were performed for 100 ns to reach equilibrium. The final 20 ns (from 80 to 100 ns) was then used to calculate the binding free energy of peptides using the molecular mechanics with generalized Born and surface area solvation (MM/GBSA) method. The steps of this method are summarized in Supplementary Figure S1.

### Confirmation of neoantigens in breast cancer cell lines by Sanger sequencing

Total RNA was extracted using a Total RNA extraction kit (Genemark, Taichung, Taiwan) and converted into cDNA by reverse transcription-polymerase chain reaction using SuperScript III First-strand Synthesis system (Invitrogen, ThermoFisher Scientific, CA). The cDNA template was amplified using specific primers (Supplementary Table S1) for each neoantigen with the Platinum™ Taq DNA Polymerase kit (Invitrogen). PCR products were run in 2% agarose gel, and the DNA was extracted from the gel with QIAquick gel extraction kit (Qiagen), measured the amount by NanodropTM Spectrophotometer (ThermoFisher Scientific, Waltham, MA), and sent for Sanger DNA sequencing (Celemics, Inc., Seoul, South Korea).

### Synthesis of neoantigen peptides

Neoantigen peptides and the corresponding WT peptides were purchased from GeneScript (Piscataway, NJ) with more than 98% purity. Reverse-phase HPLC was used to produce lyophilized peptides which then were reconstituted in DMSO (Sigma-Aldrich, Merck, Burlington, MA) and used for neoantigen-specific T cell stimulation.

### Generation of neoantigen-specific T cells by neoantigen peptide-pulsed PBMCs

Donors 1–3 (Supplementary Table S2) had HLA partially matched to PC-B-142CA cells, while donors 4–6 were partially matched to the PC-B-148CA cell. The PBMCs were used to generate neoantigen-specific T cells [27], with minor modifications. Briefly, PBMCs were isolated from 50 ml of peripheral blood using density gradient centrifugation in Lymphocyte Separation Medium (Corning, Corning, NY). RBCs were lysed using red blood cell lysis buffer. PBMCs were cultured at a density of 2 × 10^6^ cells/well with 10 μg/mL MT or WT peptides in AIM-V medium (Invitrogen) supplemented with 10% human AB serum (Sigma-Aldrich) and 20 U/ml recombinant human IL-2 (ImmunoTools, Friesoythe, Germany) and 10 ng/ml IL-7 (ImmunoTools) (only day 0) for 14 days at 37 °C in a 5% CO_2_ atmosphere. PBMCs were re-stimulated with peptides in a medium containing 20 U/ml IL-2 at days 4, 8, and 12. PBMCs without peptide stimulation and cytomegalovirus, Epstein–Barr virus, and influenza virus (CEF) pooled peptides (Mabtech, Cincinnati, OH) were used as the negative and positive control.

### Surface markers and intracellular cytokine staining using flow cytometry

The neoantigen-specific T cells were rested in plain AIM-V medium for 24 h and re-stimulated with either 10 μg/ml MT or WT peptides at 37 °C for 6 h. The 1:1000 GolgiPlug and GolgiStop (BD Biosciences, Franklin Lakes, MA) were added for 6 h. Lymphocytes were stained with 1:100 of anti-CD3-eFluor450, anti-CD4-Alexa Fluor700, anti-CD8-APC-Cy7, and anti-CD69-PerCP (ThermoFisher Scientific) for 30 min in a light protected box at 4 °C. Lymphocytes were fixed and permeabilized using CytoFix/ CytoPerm kit (BD Biosciences) and stained with anti-CD107a-FITC and anti-IFN-*γ*-APC (ThermoFisher Scientific) for 30 min. The signals were analyzed in the CytoFLEX Flow Cytometer (Beckman Coulter, Brea, CA) and by Flowjo version 10 software (BD Biosciences).

### Enzyme-linked immunospot (ELISpot) assay

IFN-*γ* production of neoantigen-specific T cells was determined using an IFN-*γ* ELISpot assay kit (Mabtech, Inc, OH). The effector lymphocytes (E) were added together with target cancer cells (T) in a ratio of E:T as 10:1. The 1 µg/ml biotinylated Mab 7-B6-1 (Mabtech, Inc, Cincinnati, OH) in 0.5% human AB serum was incubated at room temperature for 2 h and then 1:1000 alkaline phosphatase (ALP)-conjugated streptavidin (Mabtech, Inc) for 1 h. Then, 100 µl/well of BCIP/NBT plus (Mabtech) was added. The spots were photographed and automatically calculated by the CTL ImmunoSpot® Software (ImmunoSpot, Cleveland, OH).

### In vitro T cell cytotoxicity assay

1 × 10^4^ PC-B-142CA and PC-B-148CA cells were cultured in 96-well plates and peptide-pulsed lymphocytes were added at E:T of 20:1 and 40:1 for 24 h. The CellTiter 96 Aqueous One Solution Reagent (3-(4,5-dimethylthiazol-2-yl)-5-(3-carboxymethoxyphenyl)-2-(4-sulfophenyl)-2H-tetrazolium, inner salt; MTS) (Promega Corporation, Madison, WI) was used to measure the viable cancer cells by a microplate reader at OD490 nm. The cancer cell lysis was calculated according to the following formula:

$$\mathrm{\% Cancer.cell.lysis}=100-\left(\frac{{\text{OD}}. {\text{of}}. {\text{co}}-{\text{culture}}}{{\text{OD}} .{\text{of}}. {\text{target}}. {\text{cancer}} .{\text{cells}}}\right){\text{x}}100$$

### Statistical analysis

Statistical analysis was performed using GraphPad Prism V (GraphPad Software, San Diego, CA). The results were presented as mean ± standard error of the mean (SEM) of three independent experiments. Statistical comparisons of two groups were performed using Student’s *t* test, and comparisons of more than two groups were performed using one-way analysis of variance (one-way ANOVA) with Tukey’s post hoc test. Data were considered significantly different when the *P* value < 0.05.

## Results

### Mutation identification and neoantigen prediction in breast cancer patients

A total of 2513 and 3490 somatic mutations in PC-B-142CA and PC-B-148CA cells were detected (Fig. 1b, Supplementary Table S3a and b). After variant annotation, 646 NSMs were identified for PC-B-142CA and 652 for PC-B-148CA (Fig. 1b, Supplementary Tables S4a and b). The results showed 52 candidate neoantigens for PC-B-142CA cells and 4 neoantigens for PC-B-148CA cells (Fig. 1b, Supplementary Table S5a and b). The top 3 candidate neoantigens ranked by the highest IC_50_ WT/IC_50_ MT fold change and the characters of each peptide including amino acid substitution, HLA specificity, and binding affinity are summarized (Table 1). For PC-B-142CA cells, neoantigens identified from both cells and cancer tissue (Supplementary Table S6) were selected. No cancer tissue of PC-B-148CA was available; hence, the neoantigens were identified from only PC-B-148CA cells. The final 3 neoantigens of PC-B-142CA cells were ADGRL1^E274K^, adhesion G protein-coupled receptor L1 with a mutation at position 274 where E (glutamate) was replaced by K (lysine); PARP1^E619K^, poly (ADP-ribose) polymerases-1 with a mutation at position 619 where E was replaced by K; and SEC14L2^R43Q^, SEC14-like lipid binding 2 with a mutation at position 43 where R (arginine) was replaced by Q (glutamine). Those of PC-B-148CA cells were LSR^I158P^, lipolysis-stimulated lipoprotein receptor with a mutation at position 158 where I (isoleucine) was replaced by P (Proline); ALKBH6^V83M^, alkB homolog 6 with a mutation at position 83 where V (valine) was replaced by M (methionine); and GAA^I823T^, alpha-glucosidase with a mutation at position 823 where I was replaced by T (Threonine).

**Table 1:** Description of candidate neoantigens of the primary breast cancer cells screened for immunogenicity

Cell line	Gene	Amino acid substitution	HLA Allele	Mutant (MT) peptide		Wildtype (WT) sequence		Corresponding Fold Change (IC_50_ of WT/ MT)
				Sequence	Binding affinity (IC_50_, nM)	Sequences	Binding affinity (IC_50_, nM)	
PC-B-142CA	*ADGRL1*	E274K	HLA-A*11:01	KTDIDLAVDK	38.970	KTDIDLAVDE	24,602.85	631.32
	*PARP1*	E619K	HLA-A*11:01	AIEHFMKLYK	13.346	AIEHFMKLYE	2,110.72	158.15
	*SEC14L2*	R43Q	HLA-A*11:01	LQARSFDLQK	118.433	LRARSFDLQK	3,646.31	30.78
PC-B-148CA	*LSR*	I158F	HLA-A*24:02	YYQGRRFTI	10.190	YYQGRRITI	33.99	3.33
	*ALKBH6*	V83M	HLA-A*24:02	RYMDKVSNLSLF	8.182	RYVDKVSNLSLF	22.59	2.76
	*GAA*	I823T	HLA-C*07:02	LRAGYTIPL	61.563	LRAGYIIPL	88.89	1.44

### The presence of mutations of the predicted neoantigens in PC-B-142CA and PC-B-148CA cells

**Fig. 2:**
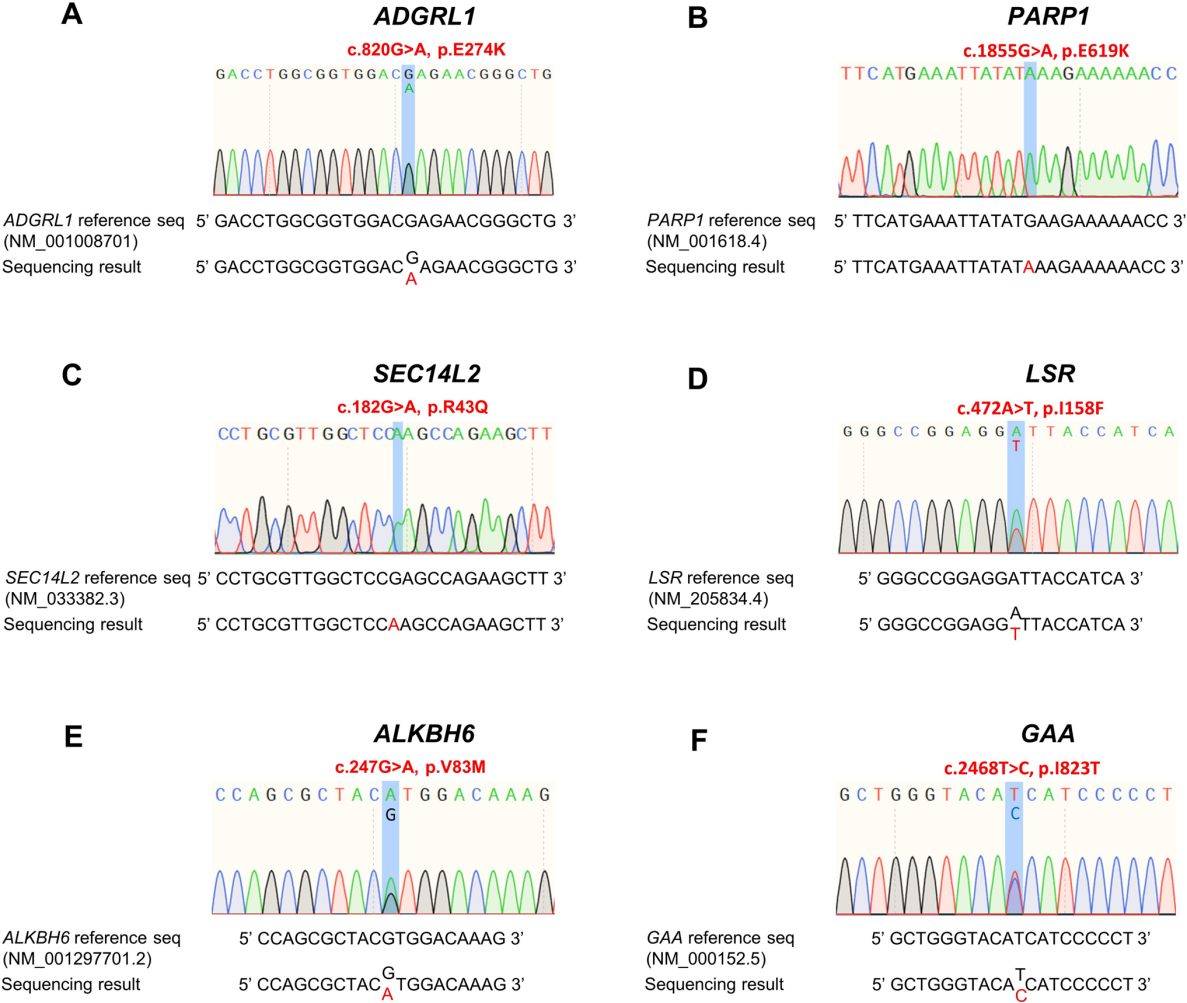
Detection of mutation in primary BCA cells by Sanger Sequencing. Electropherogram demonstrating the point mutational patterns of PC-B-142CA that contain mutations of **a**. ADGRL1, **b**. PARP1 and **c**. SEC14L2 and PC-B-148CA that contain mutations of **d**. LSR, **e**. ALKBH6 and **f**. GAA

The Sanger sequencing results confirmed that PC-B-142CA cells contained the heterozygous point mutation of *ADGRL1* (G–A transition) and homozygous point mutations of *PARP1* (G–A transition) and *SEC14L2* (G–A transition) (Fig. 2a–c), while PC-B-148CA had heterozygous point mutations of *LSR* (A–T transition), *ALKBH6* (G–A transition), and *GAA* (T–C transition) (Fig. 2d–f).

### Analysis of binding affinity of candidate neoantigen peptides and HLA Molecules

**Fig. 3:**
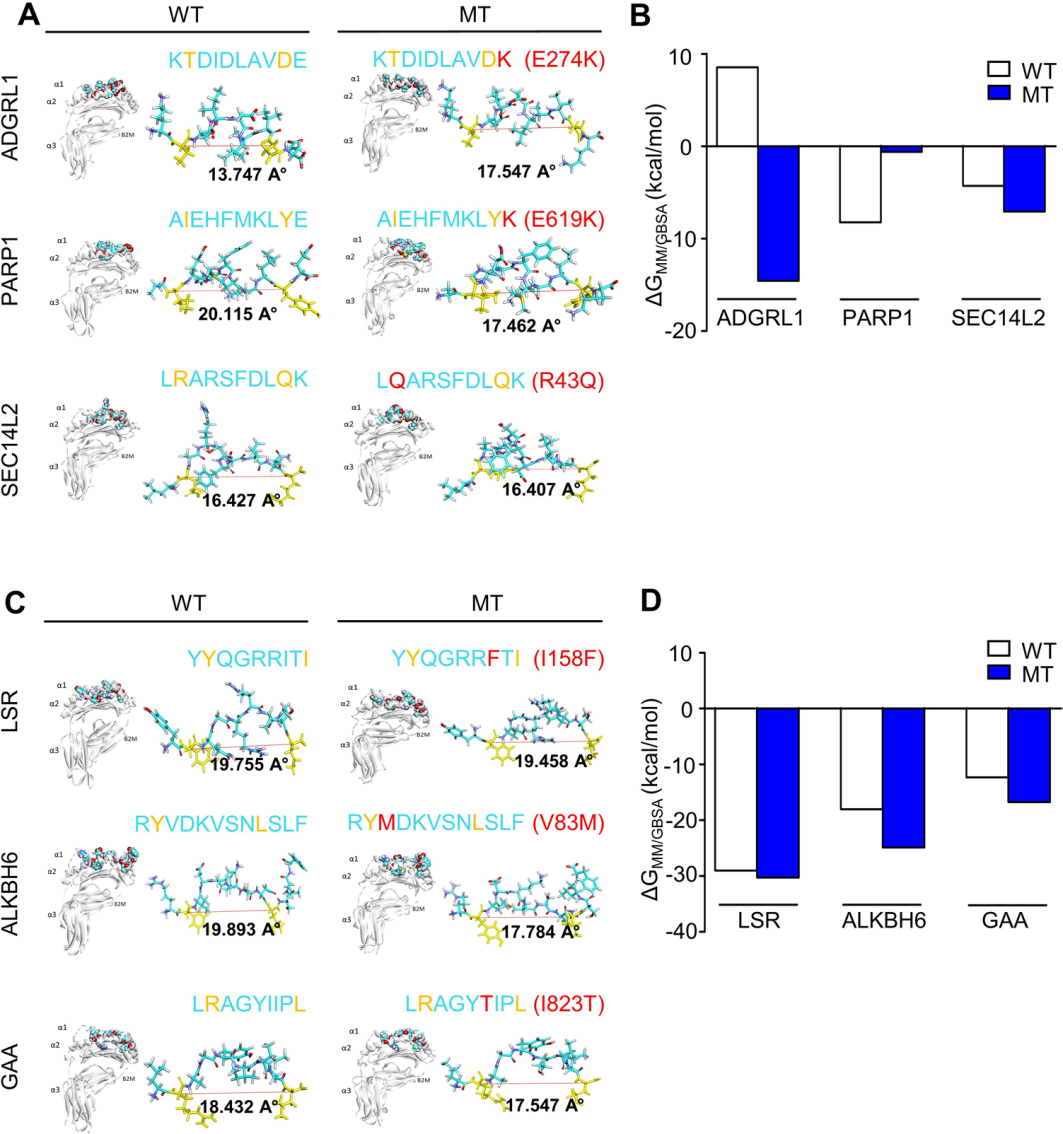
Molecular dynamic of neoantigen peptide with restricted HLA molecules. **a**. Structural model of PC-B-142CA neoantigen, ADGRL1^E274K^, PARP1^E619K^, SEC14L2^R43Q^ or corresponding WT peptide complexes with HLA-A*11:01 (left panel) and the side view of neoantigen peptides from two patients demonstrated the binding distance (Å) between Cα of P2 and P9 positions (yellow). Measurement of the peptide structure was conducted using the distance function of the Discovery Studio Visualizer Software, V21.1.0.20298, 2020. Amino acid substitution was shown in red (right panel). **b**. The ΔG_MM/GBSA_ value of each PC-B-142CA neoantigen peptide complexed with their restricted HLA molecule was calculated by AMBER software using the RMSD value from the period of 20 ns. **c**. Structural model of PC-B-148CA neoantigen, LSR^I1583F^ or ALKBH6^V83M^ complexed with HLA-A*24:02 as well as GAA^I823T^ complexed with HLA-C*07:02 (left panel) and the side view of peptides from two patients demonstrates the binding distance (Å) of between Cα of P2 and P9 position (yellow), amino acid substitution as shown in red (right panel). The side view of the peptide from two patients demonstrates the binding distance (Å) between Cα of P2 and P9 position (yellow), amino acid substitution as shown in red (right panel). d. The ΔG_MM/GBSA_ value of each PC-B-148CA neoantigen peptide complexed with their restricted HLA molecule

The anchor residues of candidate neoantigen peptides that well bound in pocket groove of HLA class I molecules were measured as the binding distance between the P2 and the P9 of the candidate neoantigen peptides (Supplementary Figure. S2a). The results showed 16 Å or longer binding distance between the P2 and the P9 of candidate neoantigen peptides or corresponding WT peptide (Fig. 3a and 3c) demonstrating the suitable distance for the peptide binding to HLA [28].

The binding affinity of candidate neoantigen peptide and HLA class I molecule was calculated by MM/GBSA method at the 20-ns MD simulation where the system’s conformational stability is showing in the number of atom contacts, number of hydrogen bonds (Supplementary Figure. S2b–c), and video files (Supplementary Video. S1–S16). The binding affinity of the control peptides to the specific HLA class I molecules is shown in Supplementary Figure. S2d). The result showed that the ΔG_MM/GBSA_ of complexes between neoantigen peptides and HLA molecule: ADGRL1^E274K^, PARP1^E619K^ or SEC14L2^R43Q^ with HLA-A*11:01 were − 14.54 kcal/mol, − 0.60 kcal/mol, and − 7.07 kcal/mol (Fig. 3b), while the ΔG_MM/GBSA_ of WT peptides with HLA-A*11:01 were 8.54 kcal/mol for ADGRL1^WT^, − 8.23 kcal/mol for PARP1^WT^, and − 4.28 kcal/mol for SEC14L2^WT^ (Fig. 3b). For PC-B-148CA-derived neoantigens, the ΔG_MM/GBSA_ of MT peptides complexed with HLA-A*24:02 were − 30.27 kcal/mol for LSR^I158F^ and − 24.88 kcal/mol for ALKBH6^V83M^, while GAA^I823T^ peptide complexed with HLA-C*07:02 revealed − 16.73 kcal/mol (Fig. 3d). In comparison, the ΔG_MM/GBSA_ of LSR^WT^, ALKBH6^WT^ and GAA^WT^ were − 29.02, − 18.01, and − 12.33 kcal/mol (Fig. 3d).

### Induction of IFN‑γ secretion of neoantigen-specific T cells

**Fig. 4:**
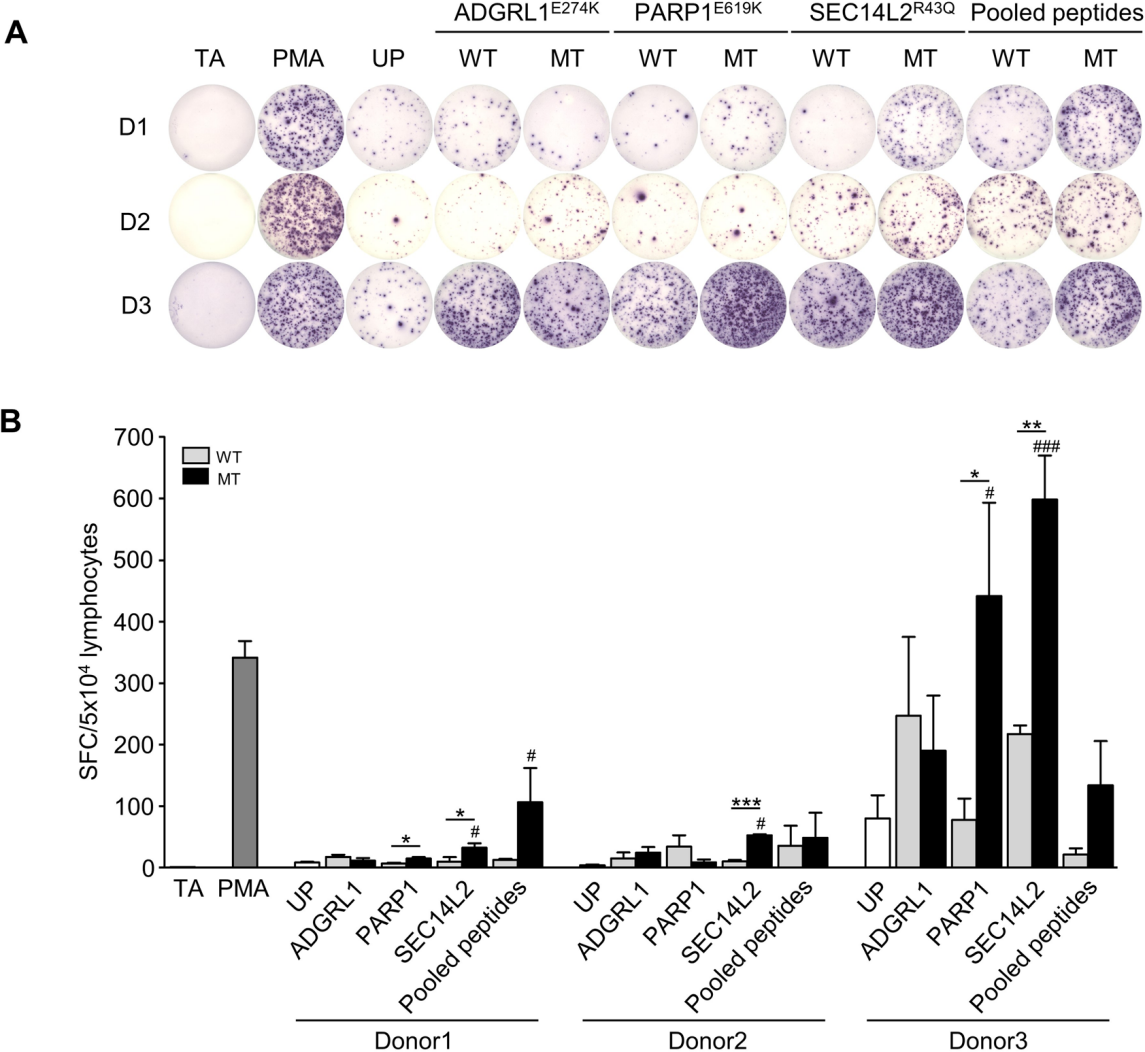
PC-B-142CA neoantigen-specific T cell response by IFN-*γ* secretion. **a**. PC-B-142CA-derived neoantigen peptides, ADGRL1^E274K^, PARP1^E619K^, SEC14L2^R43Q^, or pooled MT peptides pulsed in 3 HLA-A*11:01 healthy donor PBMCs for 14 days. Neoantigen-specific T cell responses of each neoantigen were then measured by an IFN-*γ* ELISpot assay. **b**. The number of IFN-*γ*–producing cells was counted. PMA and unpulsed (UP) conditions were used as positive and negative controls. Data were analyzed using one-way ANOVA and presented as mean ± SEM of three independent experiments. ***p < 0.001, **p < 0.01, and *p < 0.05 compared with PBMCs stimulated with WT peptide and ^###^*p* < 0.001, ^##^*p* < 0.01, and ^#^*p* < 0.05 compared with UP. TA, target cell alone (PC-B-142CA); SFC, spot-forming cell; SEM, standard error of the mean

For PC-B-142CA-derived neoantigens, the SEC14L2^R43Q^ peptides induced neoantigen-specific T cell response exhibited significantly increased IFN-*γ* secretion compared to SEC14L2^WT^ peptide in all 3 donors (donor1: 32.33 ± 7.31 vs. 9.67 ± 7.31%, donor2: 52.67 ± 1.45 vs. 10.00 ± 2.51%, donor3: 598.30 ± 71.61 vs. 217.30 ± 13.86%) (Fig. 4a and b). The PARP1^E619K^ peptide significantly induced IFN-*γ* compared to PARP1^WT^ peptide in 2 donors (donor1: 14.67 ± 2.33 vs. 6.67 ± 1.33%, donor3: 442.00 ± 151.00 vs. 77.33 ± 35.24%) (Fig. 4a and b). The ADGRL1^E274K^ peptide, however, showed no induction of T cells compared to its WT peptide in all donors. Interestingly, the pooled MT peptides could activate T cells to produce IFN-*γ* with statistical significance to peptide-unpulsed T cells (UP) in donor 1. In addition, induction of IFN-*γ* secretion from SEC14L2^R43Q^ peptide-activated T cells was observed in all donors (Fig. 4a and b).

**Fig. 5:**
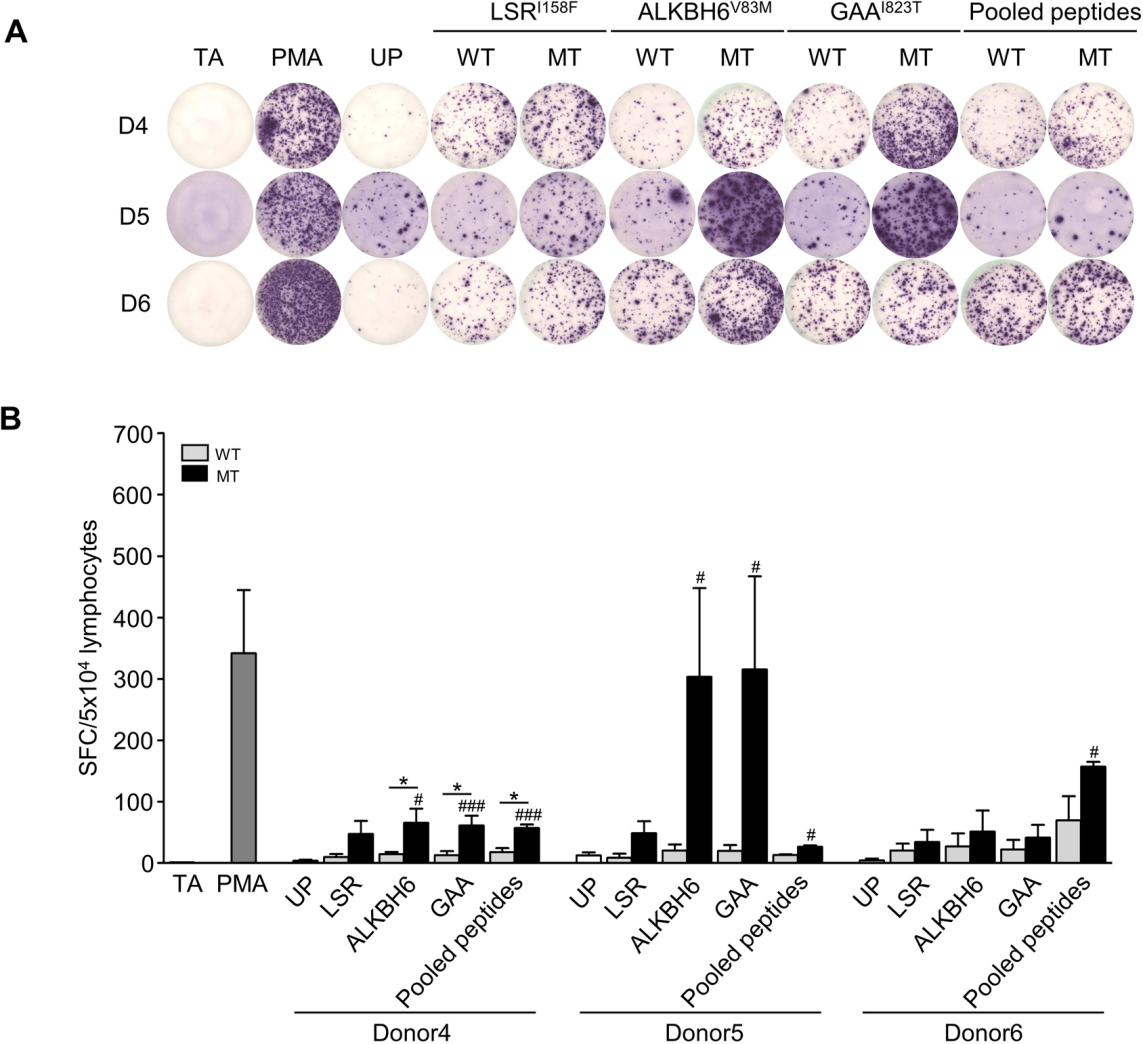
PC-B-148CA neoantigen-specific T cell response by IFN-*γ* secretion. **a**. PC-B-148CA-derived neoantigen peptides, LSR^I1583F^ ALKBH6^V83M^ GAA^I823T^ and pooled MT peptides pulsed in 3 HLA-A*24:02 and HLA-C*07:02 healthy donor PBMCs for 14 days. Neoantigen-specific T cell responses of each neoantigen were then measured by an IFN-*γ* ELISpot assay. **b**. The number of IFN-γ–producing cells was counted. PMA and unpulsed (UP) conditions were used as positive and negative controls, respectively. Data were analyzed using one-way ANOVA and presented as mean ± SEM of three independent experiments. ***p < 0.001, **p < 0.01, and *p < 0.05 compared with PBMCs stimulated with WT peptide and ^###^*p* < 0.001, ^##^*p* < 0.01, and ^#^*p* < 0.05 compared with UP. TA, target cell alone (PC-B-148CA); SFC, spot-forming cell; SEM, standard error of the mean

ALKBH6^V83M^, GAA^I823T^, and pooled MT peptides significantly induced IFN-*γ* from neoantigen-specific T cells obtained from only donor4 compared with the WT peptide counterpart (ALKBH6: 65.33 ± 23.18 vs. 14.50 ± 3.57%, GAA: 61.00 ± 16.09 vs. 12.67 ± 6.74%, pooled MT peptides: 57.00 ± 5.77 vs. 17.67 ± 6.69%) (Fig. 5a and b). The ALKBH6^V83M^ and GAA^I823T^ peptides could induce IFN-*γ* from T cells significantly more than those of unpulsed T cells in 2 donors (donors 4 and 5). Interestingly, the pooled MT peptides exhibited a significant increment of IFN-*γ* from peptide-activated T cells compared to the unpulsed T cells in all 3 donors. All 3 MT peptides could not activate T cells in donor6, while the pooled MT peptides induced T cells to produce IFN-*γ* more than that of unpulsed T cells.

### ***Stimulation with breast cancer neoantigen peptides promotes CD8***^+^***T cell activation***

**Fig. 6:**
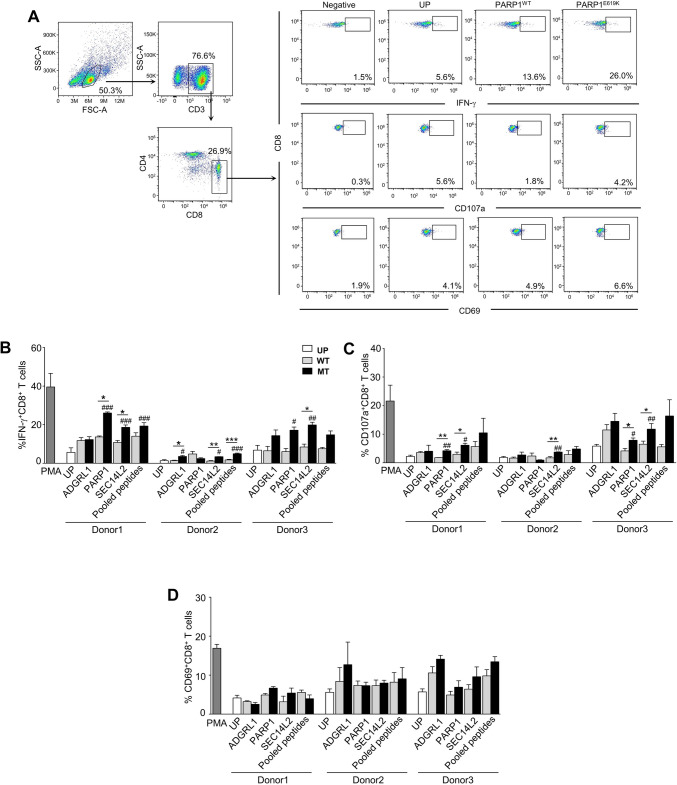
Characterization of PC-B-142CA neoantigen–specific T cells from healthy donors. **a**. Representative dot plot data of PARP1^E619K^ -specific CD8^+^ T cells from donor1 after neoantigen stimulation. PBMCs were pre-gated on CD3^+^ and CD8^+^ T cells. IFN-γ^+^, CD107a^+^, and CD69^+^ in CD8^+^ T cells were gated from the control. The frequencies of **b**. IFN-*γ*^+^ CD8^+^ T cells, **c**. CD107a^+^ CD8^+^ T cells, and **d**. CD69^+^ CD8^+^ T cells after PC-B-142CA-derived neoantigens stimulation were quantified by flow cytometry. Data were analyzed using one-way ANOVA and presented as mean ± SEM of three independent experiments. ***p < 0.001, **p < 0.01, and *p < 0.05 compared with PBMCs stimulated with WT peptide and ^###^*p* < 0.001, ^##^*p* < 0.01, and ^#^*p* < 0.05 compared with UP

To further characterize the responses induced by each candidate neoantigen, intracellular cytokine staining was performed to measure the levels of IFN-*γ*, CD107a, and CD69 in CD8^+^ T cells. The representative gating strategy of PARP1^E619K^ -induced CD8^+^ T cells activation from donor 1 and the percentages of IFN-*γ*, CD107a, and CD69 in CD8^+^ T cells were detected higher in PARP1^E619K^ MT peptide-pulsed T cells than those of WT peptide (Fig. 6a).

In PC-B-142-derived candidate neoantigens, the frequency of IFN-*γ*^+^CD8^+^ T cells in response to all 3 MT peptides (ADGRL1^E274K^, PARP1^E619K^, and SEC14L2^R43Q^) or pooled MT peptide stimulation was significantly higher than that of unpulsed peptide condition in different donors (Fig. 6b). When compared to the corresponding WT peptide, SEC14L2^R43Q^ stimulation significantly increased IFN-*γ*^+^CD8^+^ T cells in all 3 donors (donor1: 18.65 ± 1.22 vs. 10.83 ± 1.00%, donor2: 3.40 ± 0.05 vs. 1.16 ± 0.25%, and donor3: 19.87 ± 1.42 vs. 8.43 ± 1.50%). PARP1^E619K^ significantly induced IFN-γ^+^CD8^+^ T cells in donor1 (26.07 ± 0.55 vs. 13.67 ± 0.50%). ADGRL1^E274K^ and pooled MT peptides increased IFN-*γ*^+^CD8^+^ T cells in donor2 (ADGRL1: 3.53 ± 0.50 vs. 1.38 ± 0.22%, pooled MT peptides: 4.93 ± 0.36 vs. 1.74 ± 0.28%) (Fig. 6b). The frequency of CD107a^+^CD8^+^ T cells that responded to PARP1^E619K^ was significantly increased in two donors (donor1: 4.23 ± 0.37 vs. 1.80 ± 0.06% and donor 3: 7.89 ± 0.79 vs. 4.16 ± 0.85%), and to SEC14L2^R43Q^ in all 3 donors (donor1: 6.16 ± 0.68 vs. 2.99 ± 0.75%, donor2: 3.76 ± 0.09 vs. 1.72 ± 0.43% and donor3: 11.78 ± 1.95 vs. 6.51 ± 1.08%) compared to those of WT peptides (Fig. 6c). CD69^+^CD8^+^ T cells were not significantly increased by the MT peptides (Fig. 6d).

**Fig. 7:**
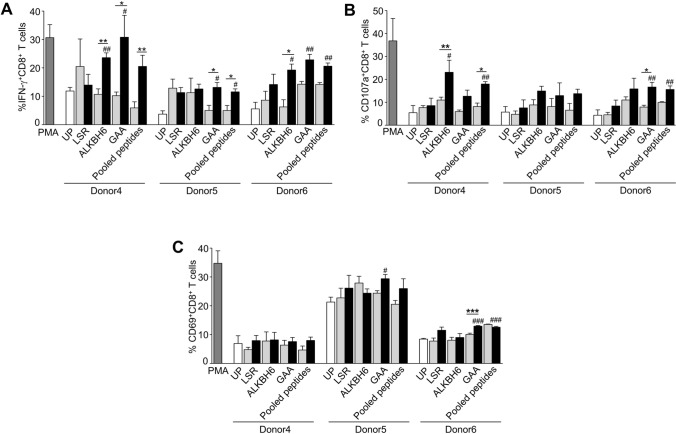
Characterization of PC-B-148CA neoantigen–specific T cells from healthy donors. PBMCs were pre-gated on CD3^+^ and CD8^+^ T cells. IFN-*γ*^+^, CD107a^+^, and CD69^+^ in CD8^+^ T cells were gated from the control. The frequencies of **a**. IFN-*γ*^+^ CD8^+^ T cells, **b**. CD107a^+^ CD8^+^ T cells, and **c**. CD69^+^ CD8^+^ T cells after PC-B-148CA-derived neoantigens stimulation were quantified by flow cytometry. Data were analyzed using one-way ANOVA and presented as mean ± SEM of three independent experiments. ***p < 0.001, **p < 0.01, and *p < 0.05 compared with PBMCs stimulated with WT peptide and ^###^*p* < 0.001, ^##^*p* < 0.01, and ^#^*p* < 0.05 compared with UP

ALKBH6^V83M^, GAA^I823T^, and pooled MT peptides significantly induced IFN-*γ*^+^CD8^+^ T cells compared to that of unpulsed peptide (Fig. 7a). The IFN-*γ*^+^CD8^+^ T cells responded to ALKBH6^V83M^ peptide stimulation was significantly higher than ALKBH6^WT^ peptide stimulation in two donors (donor4: 23.60 ± 1.68 vs. 10.70 ± 1.93% and donor6: 19.27 ± 2.07 vs. 6.26 ± 2.53%); and to GAA^I823T^ was significantly higher than GAA^WT^ stimulation in all 3 donors (donor4: 30.80 ± 7.72 vs. 10.20 ± 1.30%, donor5: 13.15 ± 1.70 vs. 5.030 ± 1.810% and donor6: 22.83 ± 1.93 vs. 14.27 ± 0.98%), and to pooled MT peptides was significantly higher than pooled WT peptides in donor4 (20.53 ± 3.92 vs. 5.92 ± 2.15%) and donor5 (11.55 ± 1.07 vs. 4.990 ± 1.79%) (Fig. 7a). The numbers of CD107a^+^CD8^+^ T cells were significantly increased by ALKBH6^V83M^ and pooled MT peptides stimulation compared to corresponding WT peptides stimulation in donor4 (ALKBH6: 23.10 ± 5.26 vs. 10.99 ± 1.27% and pooled MT peptides: 17.98 ± 1.05 vs. 8.150 ± 1.52%) and by GAA^I823T^ peptide stimulation in donor6 (16.67 ± 2.05 vs. 7.93 ± 0.80%) (Fig. 7b). GAA^I823T^ peptide significantly induced CD69^+^CD8^+^ T cells in donor6 compared to GAA^WT^ (12.93 ± 0.240 vs. 10.06 ± 0.443%) and in donors 5 and 6 compared to unpulsed condition (donor5: 12.93 ± 0.24 vs. 8.44 ± 0.25% and donor6: 12.93 ± 0.24 vs. 8.443 ± 0.25%) (Fig. 7c). The pooled MT peptides activated CD69^+^CD8^+^ T cells more than that of unpulsed in only donor6 (12.57 ± 0.29 vs. 8.443 ± 0.25%) (Fig. 7c).

### The ability of neoantigen-specific T cells in cancer cell killing

**Fig. 8:**
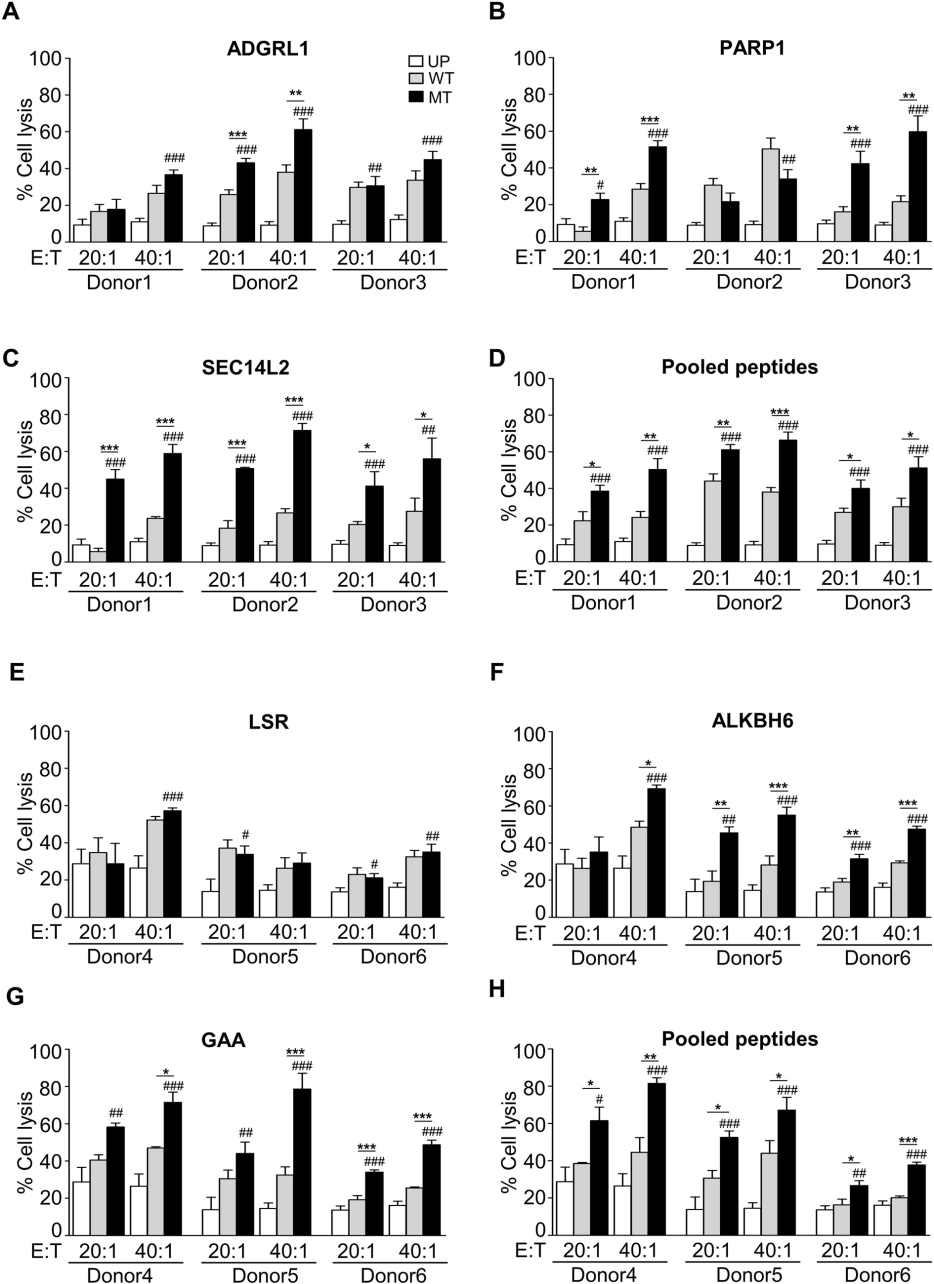
Cytotoxicity of neoantigen-specific T cells raised by in vitro stimulation of PBMCs. PBMCs from an individual healthy donor with HLA restricted to the patient were stimulated with each PC-B-142CA-derived neoantigen, **a**. ADGRL1^E274K^, **b**. PARP1^E619K^, **c**. SEC14L2^R43Q^, and **d**. MT peptide pool for 14 days. After that, neoantigen-specific T cells were co-cultured with PC-B-142CA. For PC-B-148CA-derived neoantigen, **e**. LSR^I1583F^, **f**. ALKBH6^V83^, **g**. GAA^I823T^ and **h**. MT peptide pool-specific T cells were co-cultured with PC-B-148 CA at E:T ratio of 20:1 and 40:1 for 24 h. The cytotoxicity against cancer cells was measured by MTS assay and calculated as the percentage of cell lysis. Data were analyzed using one-way ANOVA and presented as mean ± SEM of three independent experiments. ***p < 0.001, **p < 0.01, and *p < 0.05 compared with PBMCs stimulated with corresponding WT peptide and ###*p* < 0.001, ##*p* < 0.01, and #*p* < 0.05 compared with UP

The ADGRL1^E274K^ peptide-pulsed T cells from donor 2 (Fig. 8a) and PARP1^E619K^ peptide-pulsed T cells from donors 1 and 3 (Fig. 8b) significantly increased cytotoxicity against PC-B-142CA cells compared to those of WT peptide in a dose-dependent manner. SEC14L2^R43Q^ and pooled MT peptides-pulsed T cells from all donors exclusively killed PC-B-142CA target cells compared to those of WT peptides (Fig. 8c and d). No difference in PC-B-148CA cell lysis after co-cultured with LSR^I158F^ or LSR^WT^ peptide-activated T cells (Fig. 8e). However, the ALKBH6^V83M^ -, GAA^I823T^ peptide- and pooled MT peptides-pulsed T cells significantly induced PC-B-148CA cell killing at 40:1 ratio (Fig. 8f–h).

## Discussion

The neoantigens have been shown to serve as immunogenic agents for immunotherapy in BCA, and the patients with high neoantigen expression and activated T cell status exhibited improved overall survival [29]. In two advanced melanoma patients, in silico prediction of HLA binding affinity exhibited only 0.4% (2 of 501) and 1.3% (3 of 226) of neoantigens could trigger the neoantigen-specific CD8^+^ T cells responses [30]. In BCA, 20–43% of predicted neoantigens could successfully induce T cell responses [12, 31–33]. The accuracy of neoantigen prediction from the obtained DNA/RNA sequences and the capability of the neoantigen peptides to activate anti-tumor immune response are of great interest to explore. Herein, effectively detected neoantigens in patient-derived BCA cells using the modified version of the classical pVAC-Seq pipeline [20] were performed. ADGRL1^E274K^, PARP1^E619K^, and SEC14L2^R43Q^ peptides from PC-B-142CA; ALKBH6^V83M^ and GAA^I823T^ from PC-B-148CA demonstrated an elevated level of IFN-*γ* and CD107a, activated T cell markers. Interestingly, the cytotoxic activities of these T cells activated by 83% of the predicted neoantigens (5/6 neoantigens) were observed.

The somatic mutations found in the 2 patient-derived BCA cells were 2500–3500 with the NSMs around 600 estimating 17–24% of total mutations. In comparison with other studies, an average of 62 NSMs out of the total of 2096 somatic mutations (3%) in 20 advanced ovarian cancer patients [34] and 1452 mutations in 10 gastrointestinal tumors that harbor 773 NSMs (53%) were reported [35]. Moreover, 42% of the total of total 92 somatic mutations were identified as NSMs in ovarian cancer [36]. This evidence confirms the reports that BCA has been classified as a low mutation burden.

Nonomura et al. demonstrated that the prediction of 9-mer candidate neoantigen peptides, having high binding affinity to HLA class I, elicited a CD8^+^ T cell response and IFN-γ production in melanoma [37]. Contrarily, candidate neoantigens prioritized based on the highest binding affinity scores, failed to stimulate detectable T cell responses [33]. The top 5 candidate neoantigens in hepatocellular carcinoma selected by the median binding affinity (IC_50_ < 50 nM) of NetMHC, NetMHCpan, NetMHCcons, MHCflurry, MHCnuggets, PickPocket, SMM, and SMMPMBEC algorithms [33] revealed fewer than 50% of predicted neoantigens triggered cancer cell apoptosis [33]. The candidate neoantigens were predicted, in this study, based on a binding affinity < 500 nM in ≥ 3 of 5 algorithms (MHCflurry, MHCnuggetsI, Pickpocket, SMM, and SMMPMBEC) plus an IC_50_ WT/MT > 1. These criteria ensure more binding of MT peptides with HLA than WT peptides leading to avoiding the immune tolerance mechanism [38]. The top 3 candidate neoantigens from each primary BCA cell showed a suitable binding distance of amino acid anchor residues (P2-P9) in the HLA class I binding cleft ranging from 15 to 21 Å exhibited the most tightly bound of the antigenic peptides with HLA-A2.1 [28] indicating a successful T cell activation.

T cell response after activation by the predicted neoantigen peptides does not ensure clinically detectable anti-tumor activity. The ex vivo experiments to confirm neoantigen immunogenic function by stimulation of healthy donors PBMCs with the candidate neoantigen peptides were performed. Significantly, the identified neoantigens from the primary BCA cells of patients were capable of stimulating IFN-*γ* and CD107a in neoantigen-specific T cells. Similarly, survivin-derived mutant epitopes could trigger cellular immune responses including IFN-*γ*, CD107a, granzyme B, and perforin [39]. Moreover, the immunogenic peptides originating from neoantigens in hepatocellular carcinoma organoids and tissue were determined by the increased CD107a and IFN-γ by CD8^+^ T cells from healthy donor PBMCs [40]. The neoantigen-specific T cells secreted IFN-*γ* after co-culturing with autologous tumor cells were reported indicating that neoantigens on the surface of cancer cells directly activate T cell response [41]. Interestingly, CD69, an early T cell activation marker [42] and tumor-infiltrating T cell exhaustion marker [43], was partially detected in the neoantigen-specific T cells. It may be possible that a long period of T cell stimulation in some antigens (≥ 4 days) exhibited CD69 reduction in T cells [42].

It is accepted that the binding affinity of peptides to HLA molecule does not directly correlate with anti-tumor T cell activation. Not all neoantigens predicted from the algorithms with a different binding affinity between WT and MT peptides showed good immunogenic [44]; hence, the cancer cell-killing activity of the activated T cells is the key function supported by the increasing IFN-*γ*- and CD107a-producing T cells from neoantigen peptide stimulation and their cancer cell-killing activity [41, 45]. Nevertheless, LSR^I158F^-specific T cells had no killing activity against PC-B-148CA, even though this peptide had the highest binding with HLA. It confirms the concept that the in silico neoantigen prediction needs an ex vivo validation of the immunogenicity in vitro. Interestingly, the pooled MT peptides stimulation of PBMCs generated substantial T cell responses which can be supported by the finding that T cell responses to neoepitope-derived multi-peptide vaccines contribute to the clinical outcome in melanoma and pancreatic cancer patients [46, 47] and in mouse hepatocellular carcinoma [48].

In conclusion, this study proposes a modified algorithm for neoantigen prediction with high accuracy for immunogenic responses. It is the proof of concept for the identification and prioritization of neoantigens in BCA, and the obtained neoantigen peptides stimulated healthy donor PBMCs are a model for validation of the anti-tumor response of neoantigen-specific T cells against the patient-derived primary cancer cells. These may highlight the potential of using the prediction neoantigen peptides as the tumor vaccine in BCA patients.

### Supplementary Information

Below is the link to the electronic supplementary material.

Supplementary Figure S1. Workflow of a peptide-HLA binding free energy calculation (JPG 861 kb)

Supplementary Figure S2. a. The binding distance between P2 and P9 of the control peptide with the specific HLA, b. The number of contact atoms (b) and the number of hydrogen bonds (c) of a control peptide or candidate neoantigen peptide(s) with HLA class I molecules, d. The binding affinity of the control peptide with the specific HLA of PDB ID: 5GRD (SSCSSPLSK/HLA-A*11:01) · GMM/GBSA= -20.63 kcal/mol; PDB ID: 5VGE (RYRPGTVAL/HLA-C*07:02) · GMM/GBSA= -42.32 kcal/mol; PDB ID: 7JYW (TYQWIIRNW/HLA-A*24:02) · GMM/GBSA= -34.25 kcal/mol; and PDB ID: 7JYX (TYQWIIRNWET/HLA-A*24:02) · GMM/GBSA= -33.82 kcal/mol (JPG 1238 kb)

Supplementary file 3 (PDF 90 kb)

Supplementary file 4 (PDF 65 kb)

Supplementary file 5 (PDF 251 kb)

Supplementary file 6 (PDF 321 kb)

Supplementary file 7 (PDF 136 kb)

Supplementary file 8 (PDF 142 kb)

Supplementary file 9 (PDF 80 kb)

Supplementary file 10 (PDF 69 kb)

Supplementary file 11 (PDF 78 kb)

Supplementary Video S1. 5GRD (MOV 32211 kb)

Supplementary Video S2. 5VEG (MOV 23376 kb)

Supplementary Video S3. 7JYW (MOV 26779 kb)

Supplementary Video S4. 7JYX (MOV 26753 kb)

Supplementary Video S5. ADGRL1_WT/HLA-A*11:01 (MOV 23661 kb)

Supplementary Video S6. ADGRL1_E274K/HLA-A*11:01 (MOV 23839 kb)

Supplementary Video S7. PARP1_WT/HLA-A*11:01 (MOV 23014 kb)

Supplementary Video S8. PARP1_WT/HLA-A*11:01 (MOV 24788 kb)

Supplementary Video S9. SEC14L2_WT/HLA-A*11:01 (MOV 31115 kb)

Supplementary Video S10. SEC14L2_R43Q/HLA-A*11:01 (MOV 34884 kb)

Supplementary Video S11. LSR_WT/HLA-A*24:02 (MOV 33092 kb)

Supplementary Video S12. LSR_I158F/HLA-A*24:02 (MOV 25864 kb)

Supplementary Video S13. ALKBH6_WT/HLA-A*24:02 (MOV 24806 kb)

Supplementary Video S14. ALKBH6_V83M/HLA-A*24:02 (MOV 27630 kb)

Supplementary Video S15. GAA_WT/HLA-C*07:02 (MOV 26223 kb)

Supplementary Video S16. GAA_I823T/HLA-C*07:02 (MOV 24530 kb)
