# Supplementary figures and images for: Stimulating T cell responses against patient-derived breast cancer cells with neoantigen peptide-loaded peripheral blood mononuclear cells
Source: Cancer Immunol Immunother. 2024 Feb 13;73(3):43. doi: 10.1007/s00262-024-03627-3 (PMC10864427; doi:10.1007/s00262-024-03627-3)

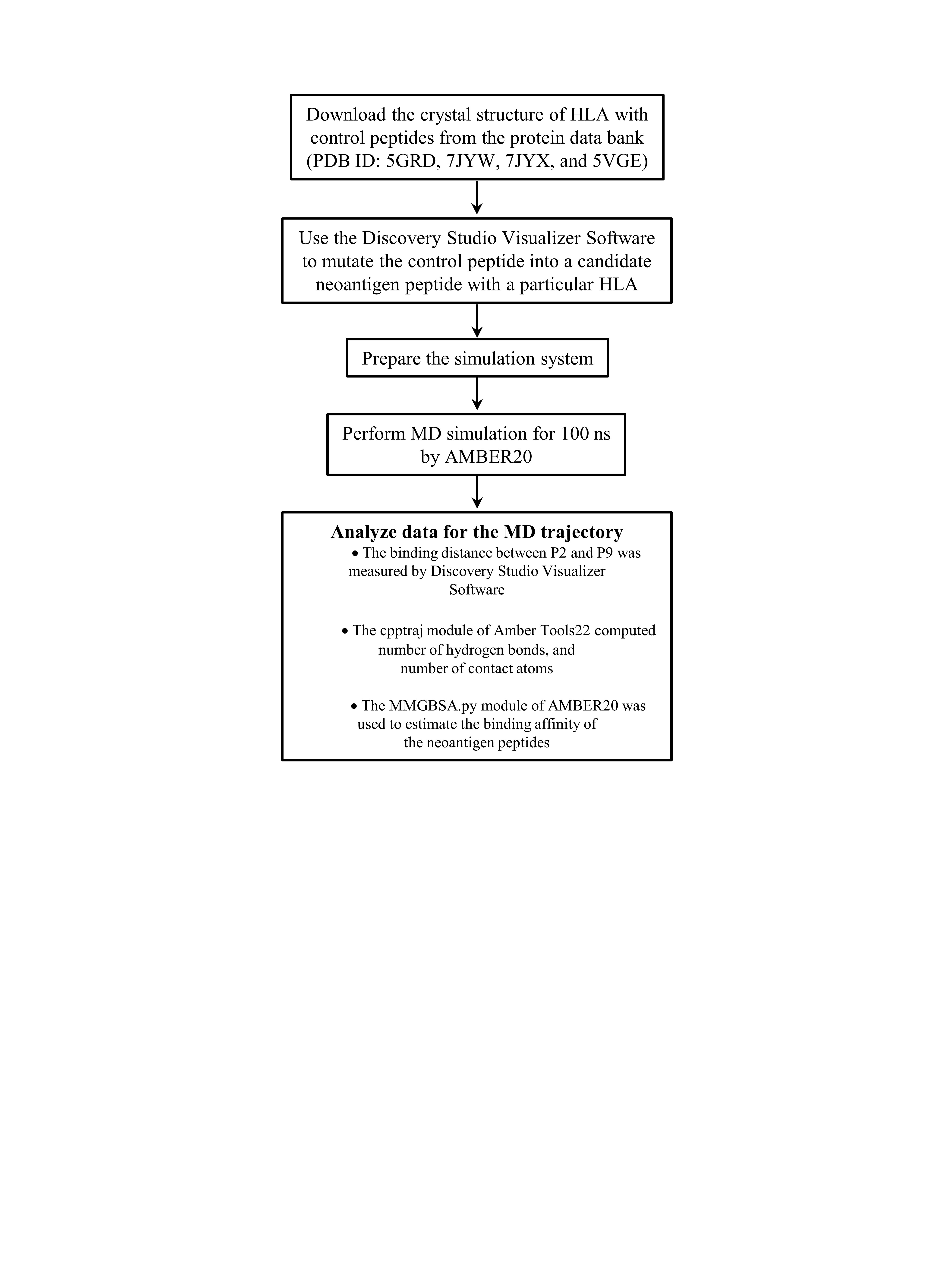

Supplement: Supplementary file 1 — Supplementary Figure S1. Workflow of a peptide-HLA binding free energy calculation (JPG 861 kb) [file 262_2024_3627_MOESM1_ESM.jpg]

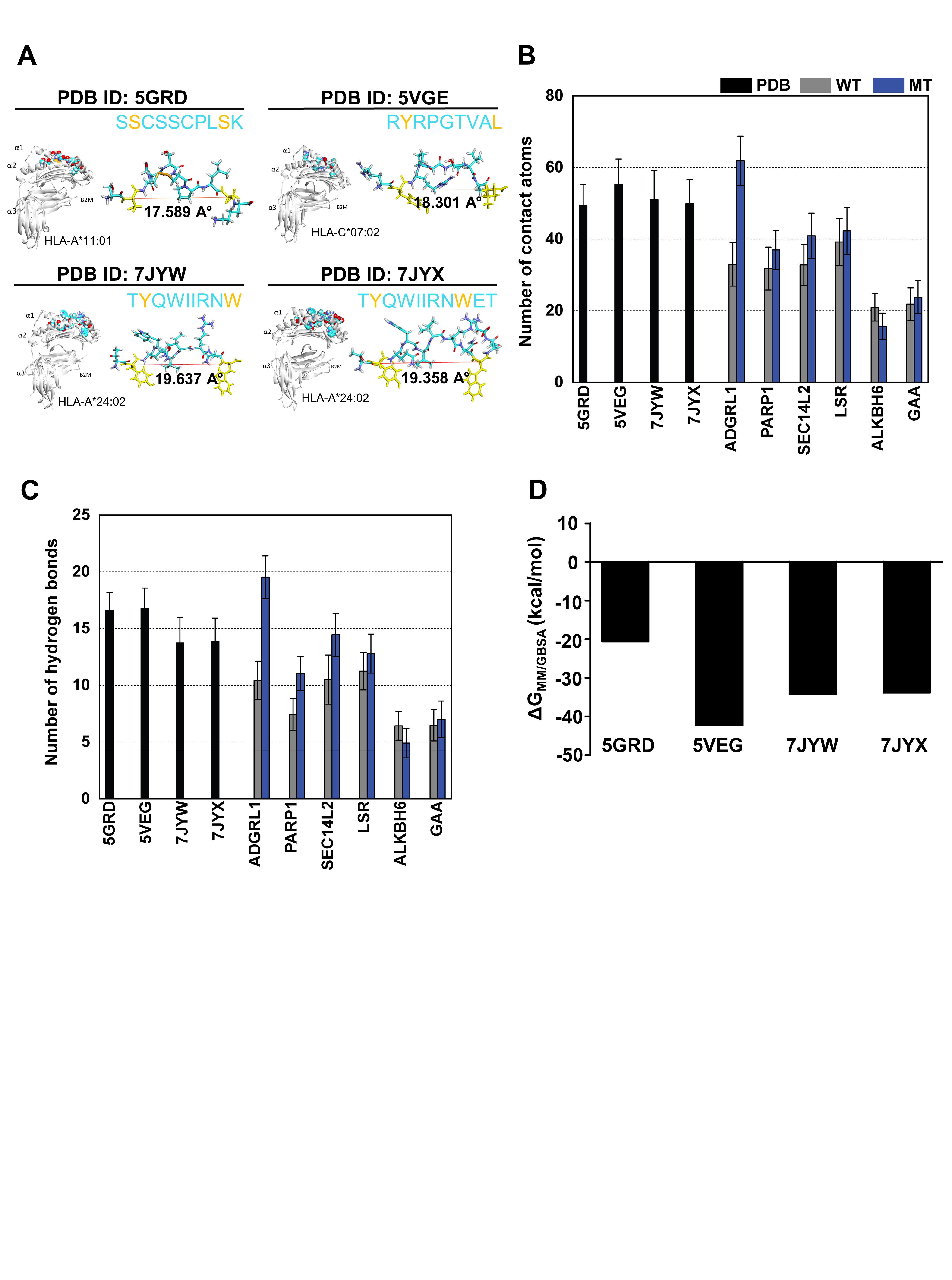

Supplement: Supplementary file 2 — Supplementary Figure S2. a. The binding distance between P2 and P9 of the control peptide with the specific HLA, b. The number of contact atoms (b) and the number of hydrogen bonds (c) of a control peptide or candidate neoantigen peptide(s) with HLA class I molecules, d. The binding affinity of the control peptide with the specific HLA of PDB ID: 5GRD (SSCSSPLSK/HLA-A*11:01) · GMM/GBSA= -20.63 kcal/mol; PDB ID: 5VGE (RYRPGTVAL/HLA-C*07:02) · GMM/GBSA= -42.32 kcal/mol; PDB ID: 7JYW (TYQWIIRNW/HLA-A*24:02) · GMM/GBSA= -34.25 kcal/mol; and PDB ID: 7JYX (TYQWIIRNWET/HLA-A*24:02) · GMM/GBSA= -33.82 kcal/mol (JPG 1238 kb) [file 262_2024_3627_MOESM2_ESM.jpg]
