# Supplementary material for: Stimulating T cell responses against patient-derived breast cancer cells with neoantigen peptide-loaded peripheral blood mononuclear cells
Source: Cancer Immunol Immunother. 2024 Feb 13;73(3):43. doi: 10.1007/s00262-024-03627-3 (PMC10864427; doi:10.1007/s00262-024-03627-3)
Supplement: Supplementary file 3 — (PDF 90 kb) [file 262_2024_3627_MOESM3_ESM.pdf]

Supplementary Table S1. Primers of neoantigens

Gene name	Ref gene No.	Forward primer (5'-3')	Reverse primer (5'-3')	Amplicon length (bp)	Tm (C°)
ADGRL1	NM_001008701	GCAGAAAGTCTTCGTGTG	CCTCAGTGGCGTAGATGA	452	53
PARP1	NM_0001618	GGAGGATGCCATTGAGCA	CCTCTTCATCCTGGCCATAG	131	55
SEC14L2	NM_033382	CCAAGTTTCGGGAGAATGT	CCTTTTGCTTTCGGAACTC	150	47
LSR	NM_205834.4	CTACGTTGAGTGCCAGGACA	CAAAGGTCAGGTCAGCATTTCC	131	59
ALKBH6	NM_001297701.2	CAGAACTGGGGTGGGCT	CACGAGGACATGGTTAGCTG	132	58
GAA	NM_000152.5	CAGACGGTGCCAGTAGAG	CTCTGTGGTTGTGAGGCC	180	57
