# Supplementary material for: Stimulating T cell responses against patient-derived breast cancer cells with neoantigen peptide-loaded peripheral blood mononuclear cells
Source: Cancer Immunol Immunother. 2024 Feb 13;73(3):43. doi: 10.1007/s00262-024-03627-3 (PMC10864427; doi:10.1007/s00262-024-03627-3)
Supplement: Supplementary file 4 — (PDF 65 kb) [file 262_2024_3627_MOESM4_ESM.pdf]

Supplementary Table S2. HLA class I phenotypes of patients and healthy donors in this study

Cancer cell lines	Donor no.	HLA class I alleles					
		HLA-A*	HLA-A*	HLA-B*	HLA-B*	HLA-C*	HLA-C*
PC-B-142CA		11:01	24:02	07:05/06	54:01	01:02	07:02
	1	11:01	24:10	18:02	40:01	07:02	07:04
	2	11:01	24:07	35:01	44:03	03:03	07:06
	3	11:01	11:01	40:01	40:01	03:03	07:02
PC-B-148CA		24:02	33:03	52:01	52:01	07:02	07:02
	4	24:02	34:01	15:35	51:01	07:02	14:02
	5	24:02	24:02	08:01	51:01	07:02	14:02
	6	02:01	24:02	07:05/06	15:02	07:02	08:01
