# Supplementary material for: Stimulating T cell responses against patient-derived breast cancer cells with neoantigen peptide-loaded peripheral blood mononuclear cells
Source: Cancer Immunol Immunother. 2024 Feb 13;73(3):43. doi: 10.1007/s00262-024-03627-3 (PMC10864427; doi:10.1007/s00262-024-03627-3)
Supplement: Supplementary file 5 — (PDF 251 kb) [file 262_2024_3627_MOESM5_ESM.pdf]

Supplementary Table S3a. List of 2,513 somatic mutations of PC-B-142CA

Number	Gene name	Chromosome	Gene_ID	Mutation	Nucleotide substitution
1	A1CF	chr10	ENSG00000148584	Silent	c.1071C>A
2	A4GALT	chr22	ENSG00000128274	3'UTR	c.*35G>C
3	AASDH	chr4	ENSG00000157426	3'UTR	c.*273A>C
4	AASDH	chr4	ENSG00000157426	Missense	c.431T>G
5	ABCA10	chr17	ENSG00000154263	Missense	c.1654G>C
6	ABCA13	chr7	ENSG00000179869	Nonsense	c.6989C>G
7	ABCA6	chr17	ENSG00000154262	Silent	c.2073G>A
8	ABCA6	chr17	ENSG00000154262	Missense	c.1975G>C
9	ABCA8	chr17	ENSG00000141338	Intron	c.4371-15G>A
10	ABCC6	chr16	ENSG00000091262	Intron	c.1943+78G>A
11	ABCG1	chr21	ENSG00000160179	Missense	c.792C>G
12	ABCG2	chr4	ENSG00000118777	3'UTR	c.*750G>A
13	ABHD11	chr7	ENSG00000106077	Missense	c.713C>T
14	ABHD17B	chr9	ENSG00000107362	3'UTR	c.*227A>T
15	ABL2	chr1	ENSG00000143322	Missense	c.2158G>C
16	AC004890.2	chr7	ENSG00000244560	Intron	n.1386-70C>T
17	AC007318.2	chr2	ENSG00000273763	Intron	n.314-690C>G
18	AC007405.3	chr2	ENSG00000239467	Intron	n.724+78G>C
19	AC008753.3	chr19	ENSG00000269877	Intron	n.283+11C>G
20	AC015910.1	chr17	ENSG00000265460	5'Flank	
21	AC015910.1	chr17	ENSG00000265460	5'Flank	
22	AC015910.1	chr17	ENSG00000265460	Intron	n.202-106G>C
23	AC015910.1	chr17	ENSG00000265460	RNA	n.207G>C
24	AC025165.3	chr12	ENSG00000257921	Intron	c.77-562G>C
25	AC048338.2	chr12	ENSG00000284934	5'UTR	c.-309G>A
26	AC053481.3	chr17	ENSG00000266365	RNA	n.34C>G
27	AC068633.1	chr3	ENSG00000243276	Intron	n.245+52C>A
28	AC079395.2	chr2	ENSG00000273634	RNA	n.2G>A
29	AC093802.1	chr2	ENSG00000220256	RNA	n.2079C>G
30	AC099489.1	chr16	ENSG00000188897	Silent	c.3819C>A
31	AC104771.1	chr4	ENSG00000271676	RNA	n.197C>A
32	AC112482.2	chr3	ENSG00000287784	3'Flank	
33	AC118758.3	chr7	ENSG00000279072	RNA	n.596C>G
34	ACADM	chr1	ENSG00000117054	5'UTR	c.-23G>C
35	ACADSB	chr10	ENSG00000196177	5'Flank	
36	ACAP3	chr1	ENSG00000131584	Missense	c.1873G>A
37	ACBD6	chr1	ENSG00000230124	5'UTR	c.-680T>A
38	ACCS	chr11	ENSG00000110455	Missense	c.1459G>A
39	ACCS	chr11	ENSG00000110455	3'UTR	c.*284G>C
40	ACOT12	chr5	ENSG00000172497	Intron	c.654-29C>T
41	ACP2	chr11	ENSG00000134575	Missense	c.940G>C
42	ACP4	chr19	ENSG00000142513	Silent	c.1044C>T
43	ACSBG1	chr15	ENSG00000103740	3'UTR	c.*3810C>T
44	ACSL6	chr5	ENSG00000164398	Missense	c.1745C>T
45	ACTL7A	chr9	ENSG00000187003	Missense	c.977C>G
46	ACTN2	chr1	ENSG00000077522	3'UTR	c.*359A>C
47	ACTR2	chr2	ENSG00000138071	Splice site	c.160-1G>A
48	ACTR3B	chr7	ENSG00000133627	Intron	c.952-53G>A
49	ACTR5	chr20	ENSG00000101442	Missense	c.1426C>G
50	ACTR8	chr3	ENSG00000113812	3'UTR	c.*363G>A
51	ACTR8	chr3	ENSG00000113812	Missense	c.619G>A

Number	Gene name	Chromosome	Gene_ID	Mutation	Nucleotide substitution
52	ACVR2A	chr2	ENSG00000121989	3'UTR	c.*832C>G
53	ADA2	chr22	ENSG00000093072	5'UTR	c.-73T>G
54	ADAM12	chr10	ENSG00000148848	3'UTR	c.*1495_*1496delinsAT
55	ADAM12	chr10	ENSG00000148848	Missense	c.1711G>A
56	ADAM23	chr2	ENSG00000114948	3'UTR	c.*1646G>C
57	ADAM23	chr2	ENSG00000114948	3'UTR	c.*2136C>T
58	ADAM28	chr8	ENSG00000042980	3'UTR	c.*4130C>G
59	ADAM28	chr8	ENSG00000042980	3'UTR	c.*4558G>A
60	ADAM29	chr4	ENSG00000168594	Missense	c.374G>A
61	ADAM8	chr10	ENSG00000151651	Missense	c.1307A>C
62	ADAMTS12	chr5	ENSG00000151388	Silent	c.1050C>T
63	ADAP1	chr7	ENSG00000105963	Intron	c.214-388G>A
64	ADARB2	chr10	ENSG00000185736	3'UTR	c.*595G>A
65	ADCY1	chr7	ENSG00000164742	3'UTR	c.*961C>G
66	ADCY10	chr1	ENSG00000143199	Missense	c.3460C>G
67	ADCY4	chr14	ENSG00000129467	Splice region	c.357+3G>A
68	ADCY5	chr3	ENSG00000173175	Silent	c.1200G>A
69	ADCY9	chr16	ENSG00000162104	Missense	c.419C>G
70	ADD2	chr2	ENSG00000075340	Intron	c.475-23G>A
71	ADGRD1	chr12	ENSG00000111452	Intron	c.104-14C>G
72	ADGRF1	chr6	ENSG00000153292	Splice region	c.552+6C>A
73	ADGRL1	chr19	ENSG00000072071	Missense	c.820G>A
74	ADGRL3	chr4	ENSG00000150471	Intron	c.587+9G>C
75	ADK	chr10	ENSG00000156110	Missense	c.1018G>C
76	ADNP	chr20	ENSG00000101126	Silent	c.1764A>T
77	ADORA1	chr1	ENSG00000163485	Nonsense	c.17C>G
78	ADRA2A	chr10	ENSG00000150594	Missense	c.1215C>A
79	AEBP1	chr7	ENSG00000106624	Silent	c.3114C>G
80	AEBP1	chr7	ENSG00000106624	3'UTR	c.*274C>T
81	AFAP1	chr4	ENSG00000196526	3'UTR	c.*611G>A
82	AFAP1L1	chr5	ENSG00000157510	Intron	c.1698+25G>C
83	AFDN	chr6	ENSG00000130396	Nonsense	c.2491G>T
84	AFF2	chrX	ENSG00000155966	3'UTR	c.*8743G>C
85	AFF4	chr5	ENSG00000072364	3'UTR	c.*3947G>C
86	AFTPH	chr2	ENSG00000119844	Intron	c.2214+27C>T
87	AGA	chr4	ENSG00000038002	3'UTR	c.*411G>C
88	AGAP1	chr2	ENSG00000157985	Missense	c.41C>A
89	AGAP2	chr12	ENSG00000135439	Silent	c.2484C>T
90	AGFG1	chr2	ENSG00000173744	Missense	c.487C>G
91	AGFG2	chr7	ENSG00000106351	3'UTR	c.*1027C>G
92	AGO4	chr1	ENSG00000134698	5'UTR	c.-123C>G
93	AGT	chr1	ENSG00000135744	3'UTR	c.*357C>G
94	AHCY	chr20	ENSG00000101444	Nonstop	c.1298G>C
95	AHCYL2	chr7	ENSG00000158467	Missense	c.1663G>A
96	AHCYL2	chr7	ENSG00000158467	3'UTR	c.*2025C>G
97	AHI1	chr6	ENSG00000135541	Missense	c.168G>C
98	AHNAK2	chr14	ENSG00000185567	Missense	c.7360G>A
99	AHNAK2	chr14	ENSG00000185567	Silent	c.7356T>C
100	AJUBA	chr14	ENSG00000129474	3'UTR	c.*274C>T
101	AKAP13	chr15	ENSG00000170776	Intron	c.34-876C>G
102	AKAP13	chr15	ENSG00000170776	Missense	c.3406C>G
103	AKAP17A	chrX	ENSG00000197976	Missense	c.953G>C
104	AKAP8	chr19	ENSG00000105127	Silent	c.564G>T

Number	Gene name	Chromosome	Gene_ID	Mutation	Nucleotide substitution
105	AKR1B15	chr7	ENSG00000227471	Intron	c.825+37C>G
106	AL445250.1	chr6	ENSG00000225096	Intron	n.185-43268C>T
107	AL583810.2	chr14	ENSG00000258811	RNA	n.430C>T
108	AL589645.1	chr9	ENSG00000287368	Intron	n.140+20843C>G
109	ALDH7A1	chr5	ENSG00000164904	Intron	c.192+749T>G
110	ALDH9A1	chr1	ENSG00000143149	Missense	c.149C>T
111	ALK	chr2	ENSG00000171094	Missense	c.977C>T
112	ALMS1	chr2	ENSG00000116127	Missense	c.6973G>C
113	ALPI	chr2	ENSG00000163295	3'UTR	c.*644G>C
114	ALPK3	chr15	ENSG00000136383	Missense	c.4228C>G
115	AMER3	chr2	ENSG00000178171	Missense	c.503G>C
116	AMER3	chr2	ENSG00000178171	Missense	c.590G>A
117	AMER3	chr2	ENSG00000178171	Silent	c.2136G>A
118	AMOTL2	chr3	ENSG00000114019	5'UTR	c.-115C>T
119	AMPD3	chr11	ENSG00000133805	Splice region	c.1430+7G>A
120	AMPH	chr7	ENSG00000078053	5'Flank	
121	ANAPC5	chr12	ENSG00000089053	Missense	c.937C>T
122	ANAPC7	chr12	ENSG00000196510	Intron	c.1235-341C>G
123	ANGPT4	chr20	ENSG00000101280	Silent	c.663G>T
124	ANGPTL3	chr1	ENSG00000132855	Missense	c.91C>G
125	ANK1	chr8	ENSG00000029534	3'UTR	c.*2191C>G
126	ANK2	chr4	ENSG00000145362	Silent	c.1249C>T
127	ANK2	chr4	ENSG00000145362	5'UTR	c.-83G>C
128	ANK2	chr4	ENSG00000145362	Missense	c.3511G>C
129	ANK3	chr10	ENSG00000151150	Missense	c.9613G>C
130	ANK3	chr10	ENSG00000151150	Missense	c.3015G>A
131	ANK3	chr10	ENSG00000151150	3'UTR	c.*164G>C
132	ANK3	chr10	ENSG00000151150	Splice site	c.997-1G>C
133	ANKEF1	chr20	ENSG00000132623	3'UTR	c.*99C>G
134	ANKH	chr5	ENSG00000154122	3'UTR	c.*598G>A
135	ANKRD26P1	chr16	ENSG00000261239	Intron	n.1543+111C>A
136	ANKRD44	chr2	ENSG00000065413	Missense	c.850C>G
137	ANKRD52	chr12	ENSG00000139645	3'UTR	c.*2971C>G
138	ANKRD6	chr6	ENSG00000135299	5'UTR	c.-124C>T
139	ANKS1A	chr6	ENSG00000064999	Intron	c.2011-14374G>C
140	ANXA1	chr9	ENSG00000135046	Silent	c.138G>C
141	ANXA11	chr10	ENSG00000122359	3'UTR	c.*1190G>A
142	AP001042.1	chr21	ENSG00000205622	RNA	n.2404C>T
143	AP2M1	chr3	ENSG00000161203	3'UTR	c.*469G>C
144	AP3B2	chr15	ENSG00000103723	Intron	c.2977+25G>A
145	AP3M1	chr10	ENSG00000185009	3'UTR	c.*1310C>G
146	AP3M2	chr8	ENSG00000070718	Missense	c.331G>C
147	AP4E1	chr15	ENSG00000081014	3'UTR	c.*1852G>A
148	APAF1	chr12	ENSG00000120868	Missense	c.784C>G
149	APC	chr5	ENSG00000134982	3'UTR	c.*597G>A
150	APLP1	chr19	ENSG00000105290	Silent	c.1758G>A
151	APP	chr21	ENSG00000142192	5'UTR	c.-158C>T
152	AQP2	chr12	ENSG00000167580	3'UTR	c.*855G>C
153	ARFGEF1	chr8	ENSG00000066777	Missense	c.4552G>C
154	ARFGEF1	chr8	ENSG00000066777	Intron	c.4339-15G>T
155	ARFGEF3	chr6	ENSG00000112379	Intron	c.352-20C>G
156	ARHGAP15	chr2	ENSG00000075884	Splice region	c.-14-3C>G
157	ARHGAP19	chr10	ENSG00000213390	3'UTR	c.*3800G>C

Number	Gene name	Chromosome	Gene_ID	Mutation	Nucleotide substitution
158	ARHGAP26	chr5	ENSG00000145819	Missense	c.82G>C
159	ARHGAP26	chr5	ENSG00000145819	3'UTR	c.*1888C>G
160	27P1-BPTFP1-	chr17	ENSG00000215769	RNA	n.1063C>G
161	ARHGAP28	chr18	ENSG00000088756	Silent	c.1338C>G
162	ARHGAP39	chr8	ENSG00000147799	Missense	c.2524A>G
163	ARHGEF11	chr1	ENSG00000132694	Silent	c.3579C>T
164	ARHGEF11	chr1	ENSG00000132694	Intron	c.1985+33C>T
165	ARHGEF33	chr2	ENSG00000214694	3'UTR	c.*730G>T
166	ARHGEF40	chr14	ENSG00000165801	Splice region	c.2035-3C>T
167	ARL6IP1	chr16	ENSG00000170540	3'UTR	c.*1136C>G
168	ARMC10P1	chr3	ENSG00000178660	3'Flank	
169	ARMC4	chr10	ENSG00000169126	Intron	c.*40+82G>C
170	ARMC9	chr2	ENSG00000135931	Intron	c.2132-9C>A
171	ARMCX4	chrX	ENSG00000196440	Silent	c.2184C>T
172	ARNT2	chr15	ENSG00000172379	Missense	c.1279G>A
173	ARNTL2	chr12	ENSG00000029153	Intron	c.183-3843G>C
174	ARNTL2	chr12	ENSG00000029153	Splice site	c.631+1G>T
175	ARPC1B	chr7	ENSG00000130429	Splice region	c.987C>G
176	ARRB1	chr11	ENSG00000137486	3'UTR	c.*1702G>C
177	ARRDC5	chr19	ENSG00000205784	Silent	c.972C>G
178	ARSH	chrX	ENSG00000205667	Splice region	c.902-4C>G
179	ASAP1	chr8	ENSG00000153317	3'UTR	c.*2487G>C
180	ASCL4	chr12	ENSG00000187855	5'UTR	c.-189G>T
181	ASPH	chr8	ENSG00000198363	Silent	c.2208G>C
182	ASPHD2	chr22	ENSG00000128203	3'UTR	c.*637C>G
183	ASPN	chr9	ENSG00000106819	Splice region	c.276T>G
184	ATAD2B	chr2	ENSG00000119778	Missense	c.2278C>T
185	ATAD3A	chr1	ENSG00000197785	5'Flank	
186	ATE1	chr10	ENSG00000107669	3'UTR	c.*2973G>C
187	ATF2	chr2	ENSG00000115966	Intron	c.978+31C>G
188	ATF6	chr1	ENSG00000118217	3'UTR	c.*661G>C
189	ATG13	chr11	ENSG00000175224	Intron	c.499+9C>G
190	ATG13	chr11	ENSG00000175224	Missense	c.1264C>T
191	ATG16L2	chr11	ENSG00000168010	Intron	c.644+17C>A
192	ATG16L2	chr11	ENSG00000168010	Missense	c.39C>T
193	ATG2A	chr11	ENSG00000110046	Silent	c.4782C>G
194	ATG2A	chr11	ENSG00000110046	Intron	c.3778-30C>G
195	ATG4B	chr2	ENSG00000168397	3'UTR	c.*229G>A
196	ATG4B	chr2	ENSG00000168397	3'UTR	c.*708G>C
197	ATG5	chr6	ENSG00000057663	3'UTR	c.*1122C>T
198	ATG9A	chr2	ENSG00000198925	5'UTR	c.-47C>T
199	ATIC	chr2	ENSG00000138363	Missense	c.1039G>A
200	ATIC	chr2	ENSG00000138363	Silent	c.1778G>A
201	ATMIN	chr16	ENSG00000166454	Missense	c.719C>G
202	ATP11C	chrX	ENSG00000101974	3'UTR	c.*1079G>A
203	ATP11C	chrX	ENSG00000101974	5'UTR	c.-748G>A
204	ATP1A4	chr1	ENSG00000132681	Silent	c.2548C>T
205	ATP2B4	chr1	ENSG00000058668	Intron	c.392-17C>T
206	ATP2C2	chr16	ENSG00000064270	Intron	c.1929+39C>T
207	ATP5F1B	chr12	ENSG00000110955	Missense	c.1342G>A
208	ATP5F1C	chr10	ENSG00000165629	Intron	c.223+10G>C
209	ATP6V0B	chr1	ENSG00000117410	Silent	c.785G>A
210	ATP6V0C	chr16	ENSG00000185883	5'UTR	c.-76C>T

Number	Gene name	Chromosome	Gene_ID	Mutation	Nucleotide substitution
211	ATP6V1A	chr3	ENSG00000114573	Silent	c.1206G>C
212	ATP6V1G1	chr9	ENSG00000136888	5'UTR	c.-46C>G
213	ATP8B3	chr19	ENSG00000130270	Silent	c.1656C>G
214	ATXN2	chr12	ENSG00000204842	5'Flank	
215	ATXN2L	chr16	ENSG00000168488	5'UTR	c.-184C>T
216	ATXN3	chr14	ENSG00000066427	3'UTR	c.*1923G>A
217	AURKC	chr19	ENSG00000105146	Missense	c.271G>A
218	AVPR1A	chr12	ENSG00000166148	Silent	c.543G>A
219	B3GALT5	chr21	ENSG00000183778	3'UTR	c.*679C>T
220	B3GNT5	chr3	ENSG00000176597	Missense	c.314C>A
221	B3GNT8	chr19	ENSG00000177191	Silent	c.828C>G
222	B4GALNT4	chr11	ENSG00000182272	Missense	c.1234G>A
223	BACE2	chr21	ENSG00000182240	Intron	.312+10976_312+10978du
224	BAHCC1	chr17	ENSG00000266074	Intron	c.178+11253C>G
225	BAHCC1	chr17	ENSG00000266074	Splice region	c.5796+3G>T
226	BAIAP3	chr16	ENSG00000007516	Intron	c.324+82C>T
227	BAZ1A	chr14	ENSG00000198604	5'UTR	c.-345C>T
228	BBC3	chr19	ENSG00000105327	Missense	c.220C>G
229	BBS10	chr12	ENSG00000179941	3'UTR	c.*464G>C
230	BCAS1	chr20	ENSG00000064787	3'UTR	c.*3658G>T
231	BCAS1	chr20	ENSG00000064787	3'UTR	c.*3652G>C
232	BCAS1	chr20	ENSG00000064787	3'UTR	c.*3274G>T
233	BCL10	chr1	ENSG00000142867	3'UTR	c.*590C>G
234	BCL2L11	chr2	ENSG00000153094	Missense	c.171C>A
235	BCL7B	chr7	ENSG00000106635	Intron	c.265+569C>G
236	BCOR	chrX	ENSG00000183337	Missense	c.1190C>G
237	BEST1	chr11	ENSG00000167995	Missense	c.1335G>T
238	BHLHE41	chr12	ENSG00000123095	Missense	c.242G>C
239	BICDL1	chr12	ENSG00000135127	Intron	c.1560+850C>T
240	BICDL2	chr16	ENSG00000162069	Intron	c.-31+298G>C
241	BICRA	chr19	ENSG00000063169	Missense	c.2821C>T
242	BLM	chr15	ENSG00000197299	Nonsense	c.311C>G
243	BLM	chr15	ENSG00000197299	Missense	c.1843C>G
244	BLZF1	chr1	ENSG00000117475	Nonsense	c.364G>T
245	BLZF1	chr1	ENSG00000117475	Missense	c.409G>A
246	BMI1	chr10	ENSG00000168283	3'UTR	c.*767C>T
247	BMPR1B	chr4	ENSG00000138696	Intron	c.73+54G>C
248	BMT2	chr7	ENSG00000164603	Missense	c.1007G>C
249	BNC1	chr15	ENSG00000169594	3'UTR	c.*470G>A
250	BNIP2	chr15	ENSG00000140299	3'UTR	c.*1801G>A
251	BNIPL	chr1	ENSG00000163141	Intron	c.792-113C>G
252	BPIFC	chr22	ENSG00000184459	3'UTR	c.*35G>C
253	BRAF	chr7	ENSG00000157764	Missense	c.1925C>G
254	BRCA1	chr17	ENSG00000012048	Missense	c.1274C>G
255	BRI3BP	chr12	ENSG00000184992	3'UTR	c.*3457C>G
256	BRI3BP	chr12	ENSG00000184992	3'UTR	c.*4685C>T
257	BRMS1	chr11	ENSG00000174744	Splice site	c.140-1G>C
258	BRWD1	chr21	ENSG00000185658	Missense	c.2830C>G
259	BRWD1	chr21	ENSG00000185658	3'UTR	c.*243G>C
260	BRWD3	chrX	ENSG00000165288	Missense	c.3586G>C
261	BTBD18	chr11	ENSG00000233436	Missense	c.306A>C
262	BTD	chr3	ENSG00000169814	3'UTR	c.*2178C>T
263	BTF3L4	chr1	ENSG00000134717	3'UTR	c.*3581G>C

Number	Gene name	Chromosome	Gene_ID	Mutation	Nucleotide substitution
264	BTG1	chr12	ENSG00000133639	3'UTR	c.*1505G>C
265	BTNL9	chr5	ENSG00000165810	Missense	c.1513C>G
266	BUB1B	chr15	ENSG00000156970	Missense	c.1990C>G
267	BUD23	chr7	ENSG00000071462	Silent	c.843C>T
268	C10orf113	chr10	ENSG00000204683	Missense	c.421G>C
269	C10orf120	chr10	ENSG00000183559	Missense	c.69A>T
270	C11orf80	chr11	ENSG00000173715	Missense	c.1031C>T
271	C11orf96	chr11	ENSG00000187479	Intron	c.300+72C>T
272	C16orf97	chr16	ENSG00000261190	Intron	n.76+24G>C
273	C1orf105	chr1	ENSG00000180999	Intron	c.199-3614G>C
274	C1orf141	chr1	ENSG00000203963	Nonsense	c.1060G>T
275	C1orf226	chr1	ENSG00000239887	3'UTR	c.*642C>T
276	C1orf56	chr1	ENSG00000143443	Missense	c.689C>T
277	C1orf61	chr1	ENSG00000125462	3'UTR	c.*219T>A
278	C1orf94	chr1	ENSG00000142698	Splice region	c.1152-6T>G
279	C1QC	chr1	ENSG00000159189	Silent	c.201G>A
280	C1QL4	chr12	ENSG00000186897	5'UTR	c.-242G>C
281	C1QTNF6	chr22	ENSG00000133466	Intron	c.51+42G>C
282	C1QTNF7	chr4	ENSG00000163145	3'UTR	c.*2283C>G
283	C1QTNF7	chr4	ENSG00000163145	3'UTR	c.*2796G>A
284	C20orf144	chr20	ENSG00000149609	Silent	c.99C>A
285	C22orf34	chr22	ENSG00000188511	RNA	n.1010C>T
286	C2orf78	chr2	ENSG00000187833	Missense	c.664C>G
287	C3orf20	chr3	ENSG00000131379	Missense	c.25G>C
288	C4orf54	chr4	ENSG00000248713	Missense	c.2758G>A
289	C4orf54	chr4	ENSG00000248713	Silent	c.222C>T
290	C5orf22	chr5	ENSG00000082213	3'UTR	c.*1337C>G
291	C7	chr5	ENSG00000112936	Nonsense	c.365C>G
292	C7orf25	chr7	ENSG00000136197	5'UTR	c.-451G>C
293	C8orf34	chr8	ENSG00000165084	Missense	c.54C>G
294	C9orf135	chr9	ENSG00000204711	Intron	c.508-3716C>G
295	C9orf72	chr9	ENSG00000147894	Missense	c.468G>T
296	CA14	chr1	ENSG00000118298	Intron	c.948-19G>C
297	CA9	chr9	ENSG00000107159	Missense	c.779C>T
298	CAAP1	chr9	ENSG00000120159	3'UTR	c.*1313T>G
299	CAAP1	chr9	ENSG00000120159	Missense	c.1026G>C
300	CACNA1A	chr19	ENSG00000141837	Splice region	c.3553+3G>C
301	CACNA1A	chr19	ENSG00000141837	Missense	c.955G>C
302	CACNA1D	chr3	ENSG00000157388	Silent	c.2706C>A
303	CACNA1E	chr1	ENSG00000198216	3'UTR	c.*1251G>T
304	CACNA1F	chrX	ENSG00000102001	Nonsense	c.73G>T
305	CACNA2D1	chr7	ENSG00000153956	Intron	c.3112+58C>T
306	CACNB1	chr17	ENSG00000067191	Intron	c.1333-42C>T
307	CACNB1	chr17	ENSG00000067191	Intron	c.629-11C>G
308	CACNB1	chr17	ENSG00000067191	5'UTR	c.-128G>A
309	CACNB4	chr2	ENSG00000182389	3'UTR	c.*4993C>G
310	CACNB4	chr2	ENSG00000182389	Intron	c.521+39G>C
311	CACNG6	chr19	ENSG00000130433	Missense	c.603G>C
312	CAD	chr2	ENSG00000084774	5'UTR	c.-26C>T
313	CADPS2	chr7	ENSG00000081803	5'UTR	c.-329C>A
314	CALB2	chr16	ENSG00000172137	Intron	c.261+14C>T
315	CALCRL	chr2	ENSG00000064989	3'UTR	c.*549G>T
316	CALD1	chr7	ENSG00000122786	Splice site	c.72-1G>A

Number	Gene name	Chromosome	Gene_ID	Mutation	Nucleotide substitution
317	CALM3	chr19	ENSG00000160014	Intron	c.422-45T>G
318	CALN1	chr7	ENSG00000183166	3'UTR	c.*1370G>A
319	CALN1	chr7	ENSG00000183166	3'UTR	c.*441C>A
320	CAMK2D	chr4	ENSG00000145349	5'UTR	c.-43C>G
321	CAMSAP1	chr9	ENSG00000130559	Intron	c.-107-92C>T
322	CAMSAP3	chr19	ENSG00000076826	Missense	c.3055C>T
323	CAMSAP3	chr19	ENSG00000076826	Missense	c.3663G>C
324	CAND1	chr12	ENSG00000111530	3'UTR	c.*649C>G
325	CAPN12	chr19	ENSG00000182472	Intron	c.730-22C>T
326	CAPN15	chr16	ENSG00000103326	Silent	c.2031C>T
327	CAPN5	chr11	ENSG00000149260	Intron	c.1288-39C>T
328	CAPRIN1	chr11	ENSG00000135387	3'UTR	c.*504_*508dup
329	CARD14	chr17	ENSG00000141527	Intron	c.2570-31C>G
330	CARF	chr2	ENSG00000138380	Missense	c.901C>T
331	CARMIL2	chr16	ENSG00000159753	Silent	c.918G>C
332	CARMIL3	chr14	ENSG00000186648	5'Flank	
333	CASTOR1	chr22	ENSG00000239282	Missense	c.332C>T
334	CASTOR2	chr7	ENSG00000274070	3'UTR	c.*3777G>C
335	CASZ1	chr1	ENSG00000130940	Missense	c.3229G>A
336	CATSPER2	chr15	ENSG00000166762	3'UTR	c.*2162G>T
337	CBFA2T2	chr20	ENSG00000078699	Intron	c.34+55C>T
338	CBFA2T2	chr20	ENSG00000078699	3'UTR	c.*126G>A
339	CBFA2T2	chr20	ENSG00000078699	3'UTR	c.*2374G>A
340	CBX6	chr22	ENSG00000183741	3'UTR	c.*3545C>G
341	CC2D1A	chr19	ENSG00000132024	Missense	c.1915G>C
342	CCDC113	chr16	ENSG00000103021	Silent	c.588C>G
343	CCDC130	chr19	ENSG00000104957	Missense	c.608G>C
344	CCDC134	chr22	ENSG00000100147	3'UTR	c.*338G>T
345	CCDC136	chr7	ENSG00000128596	Silent	c.3150G>A
346	CCDC140	chr2	ENSG00000163081	3'UTR	c.*496C>T
347	CCDC158	chr4	ENSG00000163749	Missense	c.324G>C
348	CCDC171	chr9	ENSG00000164989	Missense	c.2723C>G
349	CCDC180	chr9	ENSG00000197816	Intron	c.1793+33G>A
350	CCDC184	chr12	ENSG00000177875	3'UTR	c.*1171C>G
351	CCDC3	chr10	ENSG00000151468	3'UTR	c.*681G>A
352	CCDC30	chr1	ENSG00000186409	3'UTR	c.*91G>C
353	CCDC39	chr3	ENSG00000284862	Missense	c.241G>A
354	CCDC6	chr10	ENSG00000108091	Missense	c.92C>T
355	CCDC65	chr12	ENSG00000139537	3'UTR	c.*90G>C
356	CCDC69	chr5	ENSG00000198624	Silent	c.669T>C
357	CCDC7	chr10	ENSG00000216937	Intron	c.2123-10306G>A
358	CCDC85A	chr2	ENSG00000055813	Missense	c.583C>A
359	CCDC88A	chr2	ENSG00000115355	Missense	c.3961G>C
360	CCDC97	chr19	ENSG00000142039	5'UTR	c.-1G>C
361	CCER1	chr12	ENSG00000197651	3'UTR	c.*1069C>G
362	CCER1	chr12	ENSG00000197651	3'UTR	c.*484C>G
363	CCNB2	chr15	ENSG00000157456	Intron	c.976-35C>T
364	CCNC	chr6	ENSG00000112237	3'UTR	c.*554C>T
365	CCNL2	chr1	ENSG00000221978	Silent	c.1392G>A
366	CCNYL1	chr2	ENSG00000163249	3'UTR	c.*1364C>G
367	CCPG1	chr15	ENSG00000260916	Intron	c.176-49G>A
368	CCR6	chr6	ENSG00000112486	Missense	c.1017G>T
369	CCT6A	chr7	ENSG00000146731	Silent	c.66C>T

Number	Gene name	Chromosome	Gene_ID	Mutation	Nucleotide substitution
370	CD109	chr6	ENSG00000156535	Missense	c.2431C>T
371	CD177	chr19	ENSG00000204936	Missense	c.393G>C
372	CD44	chr11	ENSG00000026508	3'UTR	c.*317C>T
373	CD46	chr1	ENSG00000117335	Silent	c.564T>G
374	CD47	chr3	ENSG00000196776	3'UTR	c.*1794G>C
375	CD86	chr3	ENSG00000114013	3'UTR	c.*625G>A
376	CDADC1	chr13	ENSG00000102543	3'UTR	c.*1471C>G
377	CDC14A	chr1	ENSG00000079335	Missense	c.601G>C
378	CDC20B	chr5	ENSG00000164287	Intron	c.63+100C>G
379	CDH26	chr20	ENSG00000124215	Silent	c.1479C>T
380	CDH6	chr5	ENSG00000113361	3'UTR	c.*5808G>C
381	CDH8	chr16	ENSG00000150394	Missense	c.1270C>G
382	CDHR3	chr7	ENSG00000128536	5'UTR	c.-121G>C
383	CDHR3	chr7	ENSG00000128536	Missense	c.592G>C
384	CDK11B	chr1	ENSG00000248333	Missense	c.260G>A
385	CDK19	chr6	ENSG00000155111	Intron	c.25-123del
386	CDK3	chr17	ENSG00000250506	5'UTR	c.-59C>T
387	CDKL2	chr4	ENSG00000138769	Intron	c.1020+17C>T
388	CDKL3	chr5	ENSG00000006837	Missense	c.1670C>T
389	CDR1	chrX	ENSG00000184258	Missense	c.135G>C
390	CEBPB	chr20	ENSG00000172216	5'UTR	c.-1C>T
391	CEBPB	chr20	ENSG00000172216	Missense	c.1022C>A
392	CEBPZ	chr2	ENSG00000115816	5'UTR	c.-23C>G
393	CELA3A	chr1	ENSG00000142789	Intron	c.362+285C>T
394	CENPC	chr4	ENSG00000145241	Missense	c.2401C>G
395	CEP128	chr14	ENSG00000100629	3'UTR	c.*931G>T
396	CEP162	chr6	ENSG00000135315	Silent	c.336A>G
397	CEP164	chr11	ENSG00000110274	3'UTR	c.*582C>G
398	CEP57	chr11	ENSG00000166037	Missense	c.88C>T
399	CEP57L1	chr6	ENSG00000183137	3'UTR	c.*1818G>C
400	CEP78	chr9	ENSG00000148019	Intron	c.499+24C>G
401	CERCAM	chr9	ENSG00000167123	Missense	c.515A>T
402	CERS5	chr12	ENSG00000139624	Intron	c.493-26G>A
403	CERS5	chr12	ENSG00000139624	Intron	c.493-33G>T
404	CERT1	chr5	ENSG00000113163	5'UTR	c.-160G>A
405	CERT1	chr5	ENSG00000113163	5'UTR	c.-166G>C
406	CES3	chr16	ENSG00000172828	Silent	c.648C>T
407	CFAP100	chr3	ENSG00000163885	Intron	c.419-28C>T
408	CFAP100	chr3	ENSG00000163885	Splice region	c.1083-8C>G
409	CFAP47	chrX	ENSG00000165164	Missense	c.8011G>A
410	CFAP65	chr2	ENSG00000181378	Silent	c.1836C>A
411	CFAP99	chr4	ENSG00000206113	Intron	c.795+47C>G
412	CFAP99	chr4	ENSG00000206113	Missense	c.449C>T
413	CFHR3	chr1	ENSG00000116785	Intron	c.563-14G>C
414	CFL1	chr11	ENSG00000172757	5'UTR	c.-347C>G
415	CFL2	chr14	ENSG00000165410	Intron	c.4-70C>G
416	CFL2	chr14	ENSG00000165410	5'UTR	c.-54C>T
417	CFP	chrX	ENSG00000126759	Missense	c.683A>G
418	CGN	chr1	ENSG00000143375	Intron	c.2905-12C>G
419	CGN	chr1	ENSG00000143375	3'UTR	c.*1259C>G
420	CGRRF1	chr14	ENSG00000100532	3'UTR	c.*318G>C
421	CHD1L	chr1	ENSG00000131778	Missense	c.1182A>C
422	CHD2	chr15	ENSG00000173575	Intron	c.1198+33C>T

Number	Gene name	Chromosome	Gene_ID	Mutation	Nucleotide substitution
423	CHD2	chr15	ENSG00000173575	Intron	c.3414-39C>A
424	CHD5	chr1	ENSG00000116254	5'UTR	c.-90C>T
425	CHD6	chr20	ENSG00000124177	Intron	c.-88+31C>T
426	CHD7	chr8	ENSG00000171316	Missense	c.3774C>G
427	CHD9	chr16	ENSG00000177200	Missense	c.7744G>T
428	CHIC1	chrX	ENSG00000204116	5'UTR	c.-78C>A
429	CHIC1	chrX	ENSG00000204116	3'UTR	c.*373G>A
430	CHML	chr1	ENSG00000203668	3'UTR	c.*1999C>T
431	CHPF2	chr7	ENSG00000033100	Missense	c.2299G>C
432	CHRNA1	chr2	ENSG00000138435	Missense	c.1285G>C
433	CHRNA10	chr11	ENSG00000129749	3'UTR	c.*122G>C
434	CHRNA3	chr15	ENSG00000080644	5'UTR	c.-464G>C
435	CHTOP	chr1	ENSG00000160679	Intron	c.65+32C>G
436	CHURC1	chr14	ENSG00000258289	3'UTR	c.*217G>A
437	CIAO2A	chr15	ENSG00000166797	3'UTR	c.*1154C>T
438	CIITA	chr16	ENSG00000179583	Missense	c.1616G>A
439	CIP2A	chr3	ENSG00000163507	3'UTR	c.*444G>C
440	CLCA2	chr1	ENSG00000137975	Intron	c.1488+14C>A
441	CLCF1	chr11	ENSG00000175505	Intron	c.183+51G>A
442	CLCN2	chr3	ENSG00000114859	Missense	c.1423G>A
443	CLCN3	chr4	ENSG00000109572	Silent	c.1368C>T
444	CLCN7	chr16	ENSG00000103249	Intron	c.1082-10C>T
445	CLDN4	chr7	ENSG00000189143	5'UTR	c.-1791C>T
446	CLEC3A	chr16	ENSG00000166509	Missense	c.20T>C
447	CLIP1	chr12	ENSG00000130779	Splice region	c.3934-3C>T
448	CLK1	chr2	ENSG00000013441	Intron	c.1311+37G>C
449	CLLU1OS	chr12	ENSG00000205057	Intron	c.53-1365C>G
450	CLN3	chr16	ENSG00000188603	Intron	c.222+523G>A
451	CLPB	chr11	ENSG00000162129	Missense	c.994G>C
452	CLSTN2	chr3	ENSG00000158258	Intron	c.1223-58C>G
453	CLTC	chr17	ENSG00000141367	Intron	c.519+31G>C
454	CLTRN	chrX	ENSG00000147003	Nonstop	c.668G>T
455	CLUAP1	chr16	ENSG00000103351	Intron	c.928+70G>C
456	CMPK2	chr2	ENSG00000134326	Missense	c.1289C>G
457	CMTM1	chr16	ENSG00000089505	Intron	c.690+88C>G
458	CNGA3	chr2	ENSG00000144191	3'UTR	c.*658A>T
459	CNKSR2	chrX	ENSG00000149970	Intron	c.1886+14G>C
460	CNMD	chr13	ENSG00000136110	Intron	c.354+23G>C
461	CNN3	chr1	ENSG00000117519	5'UTR	c.-279C>G
462	CNNM1	chr10	ENSG00000119946	3'UTR	c.*192G>C
463	CNOT6	chr5	ENSG00000113300	3'UTR	c.*1551G>C
464	CNOT6L	chr4	ENSG00000138767	3'UTR	c.*5663G>A
465	CNPPD1	chr2	ENSG00000115649	3'UTR	c.*617C>G
466	CNRIP1	chr2	ENSG00000119865	3'UTR	c.*144C>G
467	CNTNAP5	chr2	ENSG00000155052	Missense	c.3484G>A
468	CNTRL	chr9	ENSG00000119397	Intron	c.1651-20G>A
469	COL11A1	chr1	ENSG00000060718	Intron	c.3268-24C>G
470	COL12A1	chr6	ENSG00000111799	Silent	c.3069C>G
471	COL16A1	chr1	ENSG00000084636	Silent	c.432C>T
472	COL22A1	chr8	ENSG00000169436	Intron	c.2356-64G>T
473	COL22A1	chr8	ENSG00000169436	Silent	c.1107G>A
474	COL22A1	chr8	ENSG00000169436	Silent	c.543C>G
475	COL24A1	chr1	ENSG00000171502	Missense	c.2195G>C

Number	Gene name	Chromosome	Gene_ID	Mutation	Nucleotide substitution
476	COL27A1	chr9	ENSG00000196739	Missense	c.654C>G
477	COL4A3	chr2	ENSG00000169031	Missense	c.1639G>A
478	COL4A4	chr2	ENSG00000081052	Missense	c.929G>A
479	COL5A2	chr2	ENSG00000204262	5'UTR	c.-79C>G
480	COL5A3	chr19	ENSG00000080573	Intron	c.3936+19C>G
481	COL6A3	chr2	ENSG00000163359	Missense	c.8741C>G
482	COL6A5	chr3	ENSG00000172752	Missense	c.100C>G
483	COL6A5	chr3	ENSG00000172752	Silent	c.3525A>G
484	COL6A6	chr3	ENSG00000206384	Missense	c.2481C>G
485	COL6A6	chr3	ENSG00000206384	Splice region	c.5092-3G>C
486	COL6A6	chr3	ENSG00000206384	Missense	c.5471T>G
487	COLGALT2	chr1	ENSG00000198756	3'UTR	c.*610A>C
488	COMMD2	chr3	ENSG00000114744	3'UTR	c.*2013C>G
489	COMP	chr19	ENSG00000105664	3'Flank	
490	COPG1	chr3	ENSG00000181789	Intron	c.2257-652G>C
491	COPZ2	chr17	ENSG00000005243	Silent	c.162G>A
492	COPZ2	chr17	ENSG00000005243	Missense	c.7G>A
493	COX6A2	chr16	ENSG00000156885	3'UTR	c.*42G>A
494	COX7A2L	chr2	ENSG00000115944	Intron	c.204+335G>C
495	CPS1	chr2	ENSG00000021826	Missense	c.2593G>A
496	CR1	chr1	ENSG00000203710	Intron	c.3952+94C>G
497	CRB1	chr1	ENSG00000134376	3'UTR	c.*56C>G
498	CREBRF	chr5	ENSG00000164463	Missense	c.1322T>G
499	CREG1	chr1	ENSG00000143162	Missense	c.183C>A
500	CRHR2	chr7	ENSG00000106113	3'UTR	c.*378G>C
501	CRLF3	chr17	ENSG00000176390	Missense	c.214G>A
502	CRNKL1	chr20	ENSG00000101343	Intron	c.1419+42G>A
503	CRNKL1	chr20	ENSG00000101343	Silent	c.1392G>C
504	CROCC	chr1	ENSG00000058453	Missense	c.4984G>A
505	CROCCP2	chr1	ENSG00000215908	5'Flank	
506	CROCCP5	chr1	ENSG00000186543	5'Flank	
507	CRYBG3	chr3	ENSG00000080200	Missense	c.2350G>C
508	CRYBG3	chr3	ENSG00000080200	Nonsense	c.2363C>G
509	CRYBG3	chr3	ENSG00000080200	Missense	c.6562G>C
510	CSAD	chr12	ENSG00000139631	Silent	c.600C>G
511	CSMD3	chr8	ENSG00000164796	Missense	c.10011G>C
512	CSN1S1	chr4	ENSG00000126545	Intron	c.276+89C>T
513	CSTF3	chr11	ENSG00000176102	Missense	c.1205G>A
514	CTBP2	chr10	ENSG00000175029	Silent	c.294G>A
515	CTDSP2	chr12	ENSG00000175215	3'UTR	c.*3284C>G
516	CTDSPL2	chr15	ENSG00000137770	3'UTR	c.*2975C>G
517	CTNNA1	chr5	ENSG00000044115	Silent	c.52C>T
518	CTNS	chr17	ENSG00000040531	5'Flank	
519	CTNS	chr17	ENSG00000040531	Intron	c.62-86C>T
520	CTNS	chr17	ENSG00000040531	Missense	c.343C>G
521	CTNS	chr17	ENSG00000040531	3'UTR	c.*18C>G
522	CTPS1	chr1	ENSG00000171793	Intron	c.1296+119G>A
523	CTSH	chr15	ENSG00000103811	Intron	c.124-336G>A
524	CTTN	chr11	ENSG00000085733	Intron	c.790+12C>T
525	CTTNBP2NL	chr1	ENSG00000143079	3'UTR	c.*1178G>A
526	CTXN2	chr15	ENSG00000233932	3'UTR	c.*562G>A
527	CUX2	chr12	ENSG00000111249	5'Flank	
528	CXCR5	chr11	ENSG00000160683	3'UTR	c.*78G>C

Number	Gene name	Chromosome	Gene_ID	Mutation	Nucleotide substitution
529	CXorf38	chrX	ENSG00000185753	Missense	c.455G>A
530	CXXC4	chr4	ENSG00000168772	3'Flank	
531	CYBB	chrX	ENSG00000165168	Missense	c.1486G>A
532	CYBRD1	chr2	ENSG00000071967	3'UTR	c.*1193G>C
533	CYP1A2	chr15	ENSG00000140505	Missense	c.100C>T
534	CYP1A2	chr15	ENSG00000140505	Silent	c.432C>G
535	CYP1A2	chr15	ENSG00000140505	Missense	c.481C>A
536	CYP1A2	chr15	ENSG00000140505	Missense	c.817C>G
537	CYP1A2	chr15	ENSG00000140505	Intron	c.1043-13C>A
538	CYP2A7	chr19	ENSG00000198077	Silent	c.663C>T
539	CYP2C18	chr10	ENSG00000108242	Missense	c.1135T>C
540	CYP2C18	chr10	ENSG00000108242	Nonsense	c.1137C>G
541	CYP2W1	chr7	ENSG00000073067	Missense	c.613G>A
542	CYP4V2	chr4	ENSG00000145476	Intron	c.214+258C>G
543	CYP4V2	chr4	ENSG00000145476	Missense	c.1445C>G
544	CYTH1	chr17	ENSG00000108669	3'UTR	c.*742G>A
545	CYTH4	chr22	ENSG00000100055	Missense	c.28G>A
546	CYTIP	chr2	ENSG00000115165	3'UTR	c.*606G>C
547	DAAM2	chr6	ENSG00000146122	3'UTR	c.*471G>T
548	DACH1	chr13	ENSG00000276644	3'UTR	c.*2166C>T
549	DALRD3	chr3	ENSG00000178149	Missense	c.338C>T
550	DAP3	chr1	ENSG00000132676	Silent	c.564G>A
551	DAPK1	chr9	ENSG00000196730	Intron	c.553+9C>G
552	DARS2	chr1	ENSG00000117593	Missense	c.1205G>C
553	DCAF6	chr1	ENSG00000143164	Intron	c.688+36C>A
554	DCBLD1	chr6	ENSG00000164465	Silent	c.1596C>T
555	DCBLD2	chr3	ENSG00000057019	5'UTR	c.-95G>A
556	DCD	chr12	ENSG00000161634	Intron	c.98-402G>A
557	DCHS1	chr11	ENSG00000166341	Silent	c.915C>T
558	DCLK2	chr4	ENSG00000170390	Silent	c.618G>A
559	DCLRE1C	chr10	ENSG00000152457	Missense	c.1603G>C
560	DCUN1D2	chr13	ENSG00000150401	Silent	c.75G>C
561	DDB1	chr11	ENSG00000167986	Splice region	c.666C>G
562	DDHD1	chr14	ENSG00000100523	Silent	c.252C>T
563	DDI2	chr1	ENSG00000197312	Missense	c.198G>C
564	DDIT3	chr12	ENSG00000175197	Nonsense	c.4G>T
565	DDIT4L	chr4	ENSG00000145358	3'UTR	c.*1193A>C
566	DDX11	chr12	ENSG00000013573	Missense	c.26C>G
567	DDX21	chr10	ENSG00000165732	Missense	c.1051G>A
568	DDX41	chr5	ENSG00000183258	Missense	c.518G>A
569	DDX41	chr5	ENSG00000183258	5'Flank	
570	DDX53	chrX	ENSG00000184735	Missense	c.83G>C
571	DDX56	chr7	ENSG00000136271	3'UTR	c.*526C>T
572	DECR1	chr8	ENSG00000104325	Intron	c.885+18C>G
573	DEFB115	chr20	ENSG00000215547	Nonsense	c.151G>T
574	DELE1	chr5	ENSG00000081791	3'UTR	c.*3264C>T
575	DENND1A	chr9	ENSG00000119522	3'UTR	c.*375G>A
576	DENND1B	chr1	ENSG00000213047	3'UTR	c.*3791C>G
577	DENND5A	chr11	ENSG00000184014	Missense	c.2613C>G
578	DENND5A	chr11	ENSG00000184014	Intron	c.1907-201G>A
579	DEPTOR	chr8	ENSG00000155792	3'UTR	c.*544G>T
580	DGAT2L6	chrX	ENSG00000184210	Silent	c.501G>A
581	DGKH	chr13	ENSG00000102780	3'UTR	c.*499G>C

Number	Gene name	Chromosome	Gene_ID	Mutation	Nucleotide substitution
582	DGKI	chr7	ENSG00000157680	Missense	c.910C>G
583	DGKQ	chr4	ENSG00000145214	Missense	c.860G>C
584	DGLUCY	chr14	ENSG00000133943	3'UTR	c.*272C>T
585	DGLUCY	chr14	ENSG00000133943	3'UTR	c.*447C>T
586	DHRS4	chr14	ENSG00000157326	Missense	c.463G>C
587	DIAPH1	chr5	ENSG00000131504	Missense	c.83C>T
588	DICER1	chr14	ENSG00000100697	3'UTR	c.*424G>C
589	DIDO1	chr20	ENSG00000101191	Missense	c.1155C>G
590	DIP2B	chr12	ENSG00000066084	3'UTR	c.*2933C>G
591	DIP2B	chr12	ENSG00000066084	3'UTR	c.*3037C>T
592	DIPK2A	chr3	ENSG00000181744	3'UTR	c.*1458C>G
593	DIRAS3	chr1	ENSG00000162595	Silent	c.390C>T
594	DIRAS3	chr1	ENSG00000162595	Missense	c.58C>A
595	DIS3L2	chr2	ENSG00000144535	Intron	c.1581+36628G>T
596	DLG1	chr3	ENSG00000075711	3'UTR	c.*360G>A
597	DLGAP1	chr18	ENSG00000170579	Silent	c.1533G>C
598	DLL3	chr19	ENSG00000090932	Intron	c.352-22C>G
599	DLX5	chr7	ENSG00000105880	Silent	c.381G>A
600	DMD	chrX	ENSG00000198947	Intron	c.4674+42C>A
601	DMKN	chr19	ENSG00000161249	Intron	c.1192-31C>G
602	DMKN	chr19	ENSG00000161249	Silent	c.6C>T
603	DMTF1	chr7	ENSG00000135164	Intron	c.1049+12G>A
604	DMTN	chr8	ENSG00000158856	3'UTR	c.*708G>A
605	DMXL1	chr5	ENSG00000172869	Missense	c.1219G>C
606	DMXL1	chr5	ENSG00000172869	Intron	c.2377-11A>T
607	DMXL1	chr5	ENSG00000172869	Missense	c.7706G>T
608	DNAAF1	chr16	ENSG00000154099	Missense	c.1051G>T
609	DNAH11	chr7	ENSG00000105877	Silent	c.1485C>G
610	DNAH17	chr17	ENSG00000187775	Missense	c.1474G>A
611	DNAH5	chr5	ENSG00000039139	Silent	c.12165C>T
612	DNAH5	chr5	ENSG00000039139	Intron	c.9373+49C>G
613	DNAH6	chr2	ENSG00000115423	Intron	c.1602+69C>G
614	DNAH7	chr2	ENSG00000118997	Intron	c.743+53T>A
615	DNAH8	chr6	ENSG00000124721	Silent	c.5256T>A
616	DNAH8	chr6	ENSG00000124721	Silent	c.7578C>T
617	DNAJB14	chr4	ENSG00000164031	3'UTR	c.*3091T>G
618	DNAJB6	chr7	ENSG00000105993	Silent	c.828C>G
619	DNAJC1	chr10	ENSG00000136770	Intron	c.537+182C>G
620	DNAJC10	chr2	ENSG00000077232	Missense	c.2102G>C
621	DNAJC2	chr7	ENSG00000105821	3'UTR	c.*1924G>A
622	DNAJC30	chr7	ENSG00000176410	3'UTR	c.*107G>A
623	DNAJC30	chr7	ENSG00000176410	Missense	c.265G>A
624	DNLZ	chr9	ENSG00000213221	3'UTR	c.*1995C>G
625	DNM1	chr9	ENSG00000106976	Intron	c.1422+111C>G
626	DNM3	chr1	ENSG00000197959	Intron	c.849+16C>T
627	DNM3	chr1	ENSG00000197959	Intron	c.1546-14C>G
628	DNM3	chr1	ENSG00000197959	Silent	c.1833G>A
629	DNMT3B	chr20	ENSG00000088305	3'UTR	c.*375C>G
630	DOC2A	chr16	ENSG00000149927	Missense	c.1034T>C
631	DOCK4	chr7	ENSG00000128512	Missense	c.111G>C
632	DOCK6	chr19	ENSG00000130158	Missense	c.5268C>G
633	DOP1A	chr6	ENSG00000083097	Missense	c.3686C>T
634	DPF1	chr19	ENSG00000011332	3'UTR	c.*869T>G

Number	Gene name	Chromosome	Gene_ID	Mutation	Nucleotide substitution
635	DPP3	chr11	ENSG00000254986	5'UTR	c.-49G>T
636	DPP4	chr2	ENSG00000197635	5'Flank	
637	DPP8	chr15	ENSG00000074603	3'UTR	c.*3083G>C
638	DPP8	chr15	ENSG00000074603	3'UTR	c.*1608C>G
639	DPP8	chr15	ENSG00000074603	Intron	c.2271+42G>C
640	DPY19L1	chr7	ENSG00000173852	3'UTR	c.*1602C>T
641	DPY19L1	chr7	ENSG00000173852	Missense	c.62C>T
642	DPY19L4	chr8	ENSG00000156162	3'UTR	c.*2928G>C
643	DPYD	chr1	ENSG00000188641	Intron	c.484-16dup
644	DPYSL4	chr10	ENSG00000151640	3'UTR	c.*688G>A
645	DRGX	chr10	ENSG00000165606	3'UTR	c.*947C>T
646	DROSHA	chr5	ENSG00000113360	Intron	c.1669-42C>G
647	DTD1	chr20	ENSG00000125821	Intron	c.134+18C>T
648	DTL	chr1	ENSG00000143476	Silent	c.1560G>A
649	DUSP22	chr6	ENSG00000112679	3'UTR	c.*623G>A
650	DYNLRB1	chr20	ENSG00000125971	5'UTR	c.-173C>G
651	DYRK2	chr12	ENSG00000127334	5'UTR	c.-272G>A
652	DYRK2	chr12	ENSG00000127334	3'UTR	c.*6546G>A
653	E2F3	chr6	ENSG00000112242	3'UTR	c.*395C>A
654	E2F3	chr6	ENSG00000112242	3'UTR	c.*534G>A
655	E2F8	chr11	ENSG00000129173	Missense	c.2461G>A
656	EAF2	chr3	ENSG00000145088	Missense	c.31G>C
657	EBF1	chr5	ENSG00000164330	Missense	c.277G>A
658	ECE2	chr3	ENSG00000145194	Missense	c.970C>G
659	ECI2	chr6	ENSG00000198721	Intron	c.-31+436C>G
660	ECPAS	chr9	ENSG00000136813	Intron	c.4644+45C>G
661	ECPAS	chr9	ENSG00000136813	Missense	c.819T>G
662	ECT2L	chr6	ENSG00000203734	3'UTR	c.*1217G>C
663	EDDM3A	chr14	ENSG00000181562	3'UTR	c.*340T>G
664	EEF1A1	chr6	ENSG00000156508	3'UTR	c.*1567C>G
665	EFCAB6	chr22	ENSG00000186976	Missense	c.3556G>C
666	EFHC1	chr6	ENSG00000096093	Missense	c.1029C>G
667	EFNA5	chr5	ENSG00000184349	3'UTR	c.*3118G>C
668	EGFL6	chrX	ENSG00000198759	Missense	c.242G>T
669	EHBP1	chr2	ENSG00000115504	Missense	c.3251A>G
670	EIF1AX	chrX	ENSG00000173674	3'UTR	c.*3225C>G
671	EIF4A2	chr3	ENSG00000156976	Intron	c.1080-99G>A
672	EIF4E2	chr2	ENSG00000135930	5'Flank	
673	EIF4E3	chr3	ENSG00000163412	3'UTR	c.*1810C>G
674	EIF5A2	chr3	ENSG00000163577	3'UTR	c.*4854T>G
675	ELMO1	chr7	ENSG00000155849	Intron	c.1438-24394C>T
676	ELMO3	chr16	ENSG00000102890	Intron	c.192+159C>T
677	ELMO3	chr16	ENSG00000102890	3'Flank	
678	ELOVL2	chr6	ENSG00000197977	3'UTR	c.*2259C>A
679	EMC1	chr1	ENSG00000127463	Missense	c.1045G>C
680	EML1	chr14	ENSG00000066629	Intron	c.67+16161G>A
681	EML4	chr2	ENSG00000143924	3'UTR	c.*1515C>T
682	EML5	chr14	ENSG00000165521	Missense	c.4889G>T
683	EMSY	chr11	ENSG00000158636	3'UTR	c.*412G>A
684	ENPP3	chr6	ENSG00000154269	Intron	c.78+982G>C
685	EP300	chr22	ENSG00000100393	5'Flank	
686	EPGN	chr4	ENSG00000182585	Intron	c.408-27C>G
687	EPHA10	chr1	ENSG00000183317	3'UTR	c.*700G>A

Number	Gene name	Chromosome	Gene_ID	Mutation	Nucleotide substitution
688	EPHB3	chr3	ENSG00000182580	3'UTR	c.*414G>A
689	EPHX2	chr8	ENSG00000120915	Missense	c.1144G>C
690	EPYC	chr12	ENSG00000083782	Missense	c.730C>T
691	ERAP1	chr5	ENSG00000164307	3'UTR	c.*1845G>A
692	ERBB2	chr17	ENSG00000141736	Intron	c.73+87G>A
693	ERBB2	chr17	ENSG00000141736	3'UTR	c.*51C>T
694	ERBB2	chr17	ENSG00000141736	Missense	c.3631C>G
695	ERBB2	chr17	ENSG00000141736	Silent	c.3651C>T
696	ERBB3	chr12	ENSG00000065361	Intron	c.1274+103C>G
697	ERCC4	chr16	ENSG00000175595	Splice site	c.585-1G>A
698	ERCC6	chr10	ENSG00000225830	Intron	c.543+19C>T
699	ERCC8	chr5	ENSG00000049167	5'Flank	
700	ERG	chr21	ENSG00000157554	3'UTR	c.*769G>A
701	ERG	chr21	ENSG00000157554	Missense	c.853G>T
702	ERG	chr21	ENSG00000157554	Intron	c.19-1172C>T
703	ERICH4	chr19	ENSG00000204978	Nonsense	c.103C>T
704	ERICH6	chr3	ENSG00000163645	Intron	c.1343+12C>G
705	ERICH6	chr3	ENSG00000163645	Missense	c.1309C>G
706	ERLEC1	chr2	ENSG00000068912	5'UTR	c.-113G>A
707	ERV3-1	chr7	ENSG00000213462	3'UTR	c.*145G>C
708	ESPN	chr1	ENSG00000187017	Intron	c.717+9G>C
709	ESYT1	chr12	ENSG00000139641	Intron	c.2367+49G>A
710	ESYT1	chr12	ENSG00000139641	3'UTR	c.*477C>G
711	ETFA	chr15	ENSG00000140374	Intron	c.518-13C>A
712	ETV5	chr3	ENSG00000244405	3'UTR	c.*1300_*1301delinsAT
713	ETV5	chr3	ENSG00000244405	Missense	c.625G>A
714	EVC	chr4	ENSG00000072840	Intron	c.1465-71G>C
715	EVPL	chr17	ENSG00000167880	Missense	c.4355G>A
716	EXOC7	chr17	ENSG00000182473	Intron	c.1207-44G>A
717	EXT1	chr8	ENSG00000182197	3'UTR	c.*1931C>G
718	EXT1	chr8	ENSG00000182197	3'UTR	c.*977C>G
719	EYA3	chr1	ENSG00000158161	3'UTR	c.*1049G>C
720	EYS	chr6	ENSG00000188107	Missense	c.5221G>A
721	EYS	chr6	ENSG00000188107	Missense	c.5155C>G
722	EYS	chr6	ENSG00000188107	3'UTR	c.*2177C>T
723	F13B	chr1	ENSG00000143278	Intron	c.985+18G>C
724	F13B	chr1	ENSG00000143278	Silent	c.888A>G
725	F8	chrX	ENSG00000185010	Missense	c.627G>C
726	FABP1	chr2	ENSG00000163586	3'UTR	c.*510G>C
727	FAIM2	chr12	ENSG00000135472	5'UTR	c.-538C>T
728	FAM102B	chr1	ENSG00000162636	3'UTR	c.*37A>C
729	FAM114A1	chr4	ENSG00000197712	Intron	c.437-28C>A
730	FAM114A1	chr4	ENSG00000197712	Missense	c.547G>C
731	FAM114A1	chr4	ENSG00000197712	Intron	c.550+59G>C
732	FAM118A	chr22	ENSG00000100376	Intron	c.-9-129C>G
733	FAM135B	chr8	ENSG00000147724	Silent	c.504C>G
734	FAM13A	chr4	ENSG00000138640	3'UTR	c.*2070C>T
735	FAM13A	chr4	ENSG00000138640	3'UTR	c.*463T>A
736	FAM13B	chr5	ENSG00000031003	Missense	c.2134C>T
737	FAM162A	chr3	ENSG00000114023	Missense	c.321C>A
738	FAM170A	chr5	ENSG00000164334	Intron	c.70+21C>T
739	FAM172BP	chr3	ENSG00000175841	RNA	n.92G>A
740	FAM182B	chr20	ENSG00000175170	Intron	n.646-18C>G

Number	Gene name	Chromosome	Gene_ID	Mutation	Nucleotide substitution
741	FAM184A	chr6	ENSG00000111879	3'UTR	c.*627A>G
742	FAM205A	chr9	ENSG00000205108	Missense	c.2732G>C
743	FAM214B	chr9	ENSG00000005238	3'UTR	c.*732G>A
744	FAM214B	chr9	ENSG00000005238	Intron	c.1288-19T>G
745	FAM228B	chr2	ENSG00000219626	Intron	c.932+1052G>A
746	FAM3A	chrX	ENSG00000071889	5'UTR	c.-277C>T
747	FAM3A	chrX	ENSG00000071889	5'UTR	c.-325G>T
748	FAM83A	chr8	ENSG00000147689	Missense	c.46G>A
749	FAM8A1	chr6	ENSG00000137414	5'UTR	c.-66G>C
750	FASN	chr17	ENSG00000169710	Silent	c.1362G>A
751	FASTKD5	chr20	ENSG00000215251	5'UTR	c.-158G>A
752	FAT1	chr4	ENSG00000083857	Silent	c.9339C>T
753	FBL	chr19	ENSG00000105202	Intron	c.549+12G>C
754	FBLN1	chr22	ENSG00000077942	Missense	c.1723G>A
755	FBLN1	chr22	ENSG00000077942	3'UTR	c.*118C>A
756	FBLN1	chr22	ENSG00000077942	3'UTR	c.*122C>G
757	FBN1	chr15	ENSG00000166147	Missense	c.3102G>A
758	FBRS	chr16	ENSG00000156860	Splice region	c.316-7C>T
759	FBRSL1	chr12	ENSG00000112787	Silent	c.2019C>T
760	FBXL22	chr15	ENSG00000197361	3'UTR	c.*8C>G
761	FBXO4	chr5	ENSG00000151876	Missense	c.904G>C
762	FCAMR	chr1	ENSG00000162897	3'UTR	c.*311C>T
763	FCGR3B	chr1	ENSG00000162747	Silent	c.114T>C
764	FCGR3B	chr1	ENSG00000162747	Missense	c.108C>G
765	FCHO1	chr19	ENSG00000130475	Splice region	c.790+3G>A
766	FCRL3	chr1	ENSG00000160856	3'UTR	c.*2174C>G
767	FCRL4	chr1	ENSG00000163518	3'UTR	c.*227G>C
768	FCRL5	chr1	ENSG00000143297	Intron	c.32-44T>A
769	FCSK	chr16	ENSG00000157353	Missense	c.340G>C
770	FCSK	chr16	ENSG00000157353	Missense	c.622G>C
771	FER1L5	chr2	ENSG00000249715	Silent	c.3900G>A
772	FER1L6	chr8	ENSG00000214814	Missense	c.446C>T
773	FERMT1	chr20	ENSG00000101311	3'UTR	c.*399G>C
774	FERMT2	chr14	ENSG00000073712	Intron	c.157+1831G>C
775	FES	chr15	ENSG00000182511	Missense	c.912G>C
776	FGA	chr4	ENSG00000171560	Missense	c.1141G>A
777	FGD1	chrX	ENSG00000102302	Intron	c.1636+49C>G
778	FGD2	chr6	ENSG00000146192	Missense	c.1881G>C
779	FGF3	chr11	ENSG00000186895	Missense	c.448G>A
780	FGFR1	chr8	ENSG00000077782	3'UTR	c.*2447G>T
781	FGFR2	chr10	ENSG00000066468	Intron	c.673-495G>C
782	FHAD1	chr1	ENSG00000142621	Intron	c.1827+6241G>C
783	FIBCD1	chr9	ENSG00000130720	Intron	c.-402-860C>T
784	FIG4	chr6	ENSG00000112367	Intron	c.1271+28G>C
785	FIGNL1	chr7	ENSG00000132436	Silent	c.45G>A
786	FKBP14	chr7	ENSG00000106080	Missense	c.454G>A
787	FKBP15	chr9	ENSG00000119321	3'UTR	c.*260A>C
788	FKBP15	chr9	ENSG00000119321	Missense	c.2642C>G
789	FKBP5	chr6	ENSG00000096060	3'UTR	c.*889C>G
790	FKBP7	chr2	ENSG00000079150	Intron	c.374-3348G>A
791	FKBP7	chr2	ENSG00000079150	5'UTR	c.-66C>G
792	FLG	chr1	ENSG00000143631	Frame_Shift_Ins	c.477dup
793	FLNA	chrX	ENSG00000196924	Missense	c.6166G>C

Number	Gene name	Chromosome	Gene_ID	Mutation	Nucleotide substitution
794	FLNA	chrX	ENSG00000196924	5'UTR	c.-95C>T
795	FLOT2	chr17	ENSG00000132589	Missense	c.1108G>C
796	FMN1	chr15	ENSG00000248905	Missense	c.1744G>A
797	FMN1	chr15	ENSG00000248905	Intron	c.1554+41G>C
798	FMNL2	chr2	ENSG00000157827	Missense	c.530G>A
799	FMNL2	chr2	ENSG00000157827	Intron	c.2845-46C>G
800	FMNL3	chr12	ENSG00000161791	3'UTR	c.*9C>T
801	FMO1	chr1	ENSG00000010932	Missense	c.1497C>G
802	FNDC1	chr6	ENSG00000164694	Missense	c.1299C>G
803	FNDC3B	chr3	ENSG00000075420	3'UTR	c.*223G>T
804	FNDC3B	chr3	ENSG00000075420	3'UTR	c.*1430G>C
805	FOLH1	chr11	ENSG00000086205	Missense	c.96C>G
806	FOS	chr14	ENSG00000170345	5'UTR	c.-398G>T
807	FOXK2	chr17	ENSG00000141568	3'UTR	c.*1358G>C
808	FOXL1	chr16	ENSG00000176678	Missense	c.1012C>G
809	FOXL1	chr16	ENSG00000176678	3'UTR	c.*1154C>G
810	FOXP2	chr7	ENSG00000128573	3'UTR	c.*516G>A
811	FOXP4	chr6	ENSG00000137166	Intron	c.1150-52C>T
812	FPGS	chr9	ENSG00000136877	Intron	c.430-41G>A
813	FPGT-TNNI3K	chr1	ENSG00000259030	3'UTR	c.*2050C>T
814	FRAT2	chr10	ENSG00000181274	3'UTR	c.*1245G>A
815	FREM2	chr13	ENSG00000150893	Missense	c.6063G>C
816	FRMD3	chr9	ENSG00000172159	3'UTR	c.*1532C>G
817	FRMD8P1	chrX	ENSG00000227942	RNA	n.1179C>A
818	FRMPD4	chrX	ENSG00000169933	3'UTR	c.*2841G>A
819	FRY	chr13	ENSG00000073910	Silent	c.3342C>T
820	FRY	chr13	ENSG00000073910	Intron	c.7414-118G>C
821	FRYL	chr4	ENSG00000075539	3'UTR	c.*1760G>C
822	FRYL	chr4	ENSG00000075539	Intron	c.4266+16C>G
823	FSD1L	chr9	ENSG00000106701	Intron	c.369-7240G>T
824	FSIP2	chr2	ENSG00000188738	Missense	c.10984C>G
825	FSIP2	chr2	ENSG00000188738	Missense	c.17044G>C
826	FSTL1	chr3	ENSG00000163430	3'UTR	c.*3742C>G
827	FSTL1	chr3	ENSG00000163430	3'UTR	c.*3183C>G
828	FSTL1	chr3	ENSG00000163430	3'UTR	c.*1954C>G
829	FSTL1	chr3	ENSG00000163430	3'UTR	c.*1288C>G
830	FSTL1	chr3	ENSG00000163430	3'UTR	c.*1219C>G
831	FTCDNL1	chr2	ENSG00000226124	Missense	c.398C>A
832	FTO	chr16	ENSG00000140718	3'UTR	c.*7652G>C
833	FTSJ3	chr17	ENSG00000108592	Intron	c.68-42C>A
834	FUT4	chr11	ENSG00000196371	Missense	c.680C>T
835	FUT8	chr14	ENSG00000033170	Silent	c.123C>T
836	FXR1	chr3	ENSG00000114416	3'UTR	c.*165del
837	FYCO1	chr3	ENSG00000163820	Missense	c.177G>C
838	FZD7	chr2	ENSG00000155760	Nonsense	c.626C>G
839	G3BP1	chr5	ENSG00000145907	Missense	c.372C>G
840	GAA	chr17	ENSG00000171298	Nonsense	c.118C>T
841	GAB4	chr22	ENSG00000215568	3'UTR	c.*545C>T
842	GABRA2	chr4	ENSG00000151834	5'UTR	c.-92G>A
843	GABRA3	chrX	ENSG00000011677	3'UTR	c.*360G>C
844	GABRR2	chr6	ENSG00000111886	Missense	c.961G>A
845	GABRR2	chr6	ENSG00000111886	Missense	c.789C>G
846	GABRR2	chr6	ENSG00000111886	Intron	c.737-26C>T

Number	Gene name	Chromosome	Gene_ID	Mutation	Nucleotide substitution
847	GAD1	chr2	ENSG00000128683	Missense	c.564C>A
848	GAD2	chr10	ENSG00000136750	Intron	c.286+17G>C
849	GALK2	chr15	ENSG00000156958	Intron	c.20+13966C>G
850	GALM	chr2	ENSG00000143891	Intron	c.553-30G>T
851	GALNT16	chr14	ENSG00000100626	Missense	c.931G>T
852	GALNTL6	chr4	ENSG00000174473	Intron	c.924-40C>T
853	GALR1	chr18	ENSG00000166573	Missense	c.237C>G
854	GALR2	chr17	ENSG00000182687	3'Flank	
855	GAS2L3	chr12	ENSG00000139354	Intron	c.446-72C>T
856	GASK1B	chr4	ENSG00000164125	3'UTR	c.*2918G>C
857	GATA1	chrX	ENSG00000102145	Intron	c.599-26C>A
858	GATA3	chr10	ENSG00000107485	Intron	c.-369-4007C>G
859	GATM	chr15	ENSG00000171766	Missense	c.137C>T
860	GBX2	chr2	ENSG00000168505	Silent	c.120C>T
861	GBX2	chr2	ENSG00000168505	Silent	c.84C>T
862	GBX2	chr2	ENSG00000168505	5'UTR	c.-213C>G
863	GCKR	chr2	ENSG00000084734	Silent	c.180C>A
864	GCKR	chr2	ENSG00000084734	Silent	c.348C>T
865	GCNT2	chr6	ENSG00000111846	Missense	c.994G>C
866	GCOM1	chr15	ENSG00000137878	Intron	c.1305-7845C>G
867	GDA	chr9	ENSG00000119125	3'UTR	c.*2375C>A
868	GDF5	chr20	ENSG00000125965	3'UTR	c.*269G>T
869	GDI1	chrX	ENSG00000203879	Intron	c.719+34G>A
870	GDPD1	chr17	ENSG00000153982	3'UTR	c.*31G>C
871	GET1	chr21	ENSG00000182093	Intron	c.-1+2449C>A
872	GET3	chr19	ENSG00000198356	Intron	c.717+41G>C
873	GET4	chr7	ENSG00000239857	5'Flank	
874	GFM1	chr3	ENSG00000168827	Intron	c.1658+581C>G
875	GFRA1	chr10	ENSG00000151892	Missense	c.907G>C
876	GFRA3	chr5	ENSG00000146013	Missense	c.910T>G
877	GGA3	chr17	ENSG00000125447	Missense	c.1766C>G
878	GGT1	chr22	ENSG00000286070	Intron	c.296-55C>T
879	GIGYF2	chr2	ENSG00000204120	Intron	c.492-35G>C
880	GIMAP2	chr7	ENSG00000106560	Silent	c.453C>T
881	GIMAP5	chr7	ENSG00000196329	RNA	n.428C>A
882	GIMAP6	chr7	ENSG00000133561	3'UTR	c.*2174C>G
883	GIMAP7	chr7	ENSG00000179144	Missense	c.43G>C
884	GIMAP8	chr7	ENSG00000171115	Missense	c.1615G>A
885	GINS1	chr20	ENSG00000101003	3'UTR	c.*1501C>T
886	GINS3	chr16	ENSG00000181938	Missense	c.516G>C
887	GIPC3	chr19	ENSG00000179855	Intron	c.411+59C>G
888	GJB1	chrX	ENSG00000169562	Missense	c.389C>T
889	GK2	chr4	ENSG00000196475	Missense	c.229G>A
890	GLI2	chr2	ENSG00000074047	Missense	c.4213G>T
891	GLIPR1L2	chr12	ENSG00000180481	Missense	c.604G>C
892	GLIPR2	chr9	ENSG00000122694	3'UTR	c.*648C>G
893	GLIPR2	chr9	ENSG00000122694	3'UTR	c.*932C>G
894	GLO1	chr6	ENSG00000124767	3'UTR	c.*673G>C
895	GLP2R	chr17	ENSG00000065325	Intron	c.382+88C>A
896	GLRA3	chr4	ENSG00000145451	3'UTR	c.*3609G>C
897	GLRA3	chr4	ENSG00000145451	5'UTR	c.-375C>G
898	GLRX3	chr10	ENSG00000108010	Missense	c.281C>T
899	GLUD1	chr10	ENSG00000148672	Intron	c.742-34G>A

Number	Gene name	Chromosome	Gene_ID	Mutation	Nucleotide substitution
900	GMCL1	chr2	ENSG00000087338	5'UTR	c.-112G>C
901	GMEB1	chr1	ENSG00000162419	Missense	c.572G>A
902	GMFB	chr14	ENSG00000197045	Splice site	c.4-1G>C
903	GMNC	chr3	ENSG00000205835	3'UTR	c.*2026A>G
904	GMPS	chr3	ENSG00000163655	Splice region	c.123C>T
905	GMPS	chr3	ENSG00000163655	3'UTR	c.*1882C>T
906	GMPS	chr3	ENSG00000163655	3'UTR	c.*2269C>A
907	GNB2	chr7	ENSG00000172354	Missense	c.482C>G
908	GNE	chr9	ENSG00000159921	3'UTR	c.*1994C>T
909	GNG7	chr19	ENSG00000176533	Silent	c.186G>A
910	GNGT2	chr17	ENSG00000167083	3'UTR	c.*356C>G
911	GNPDA2	chr4	ENSG00000163281	Intron	c.226+24A>C
912	GOLGA1	chr9	ENSG00000136935	Silent	c.963G>A
913	GOLGA6L2	chr15	ENSG00000174450	Missense	c.654G>C
914	GOLGB1	chr3	ENSG00000173230	3'UTR	c.*387G>C
915	GOLT1A	chr1	ENSG00000174567	Silent	c.156G>C
916	GP5	chr3	ENSG00000178732	3'UTR	c.*775C>G
917	GP5	chr3	ENSG00000178732	Missense	c.176G>C
918	GPA33	chr1	ENSG00000143167	Missense	c.695C>T
919	GPAT4	chr8	ENSG00000158669	3'UTR	c.*2615G>A
920	GPD1	chr12	ENSG00000167588	Intron	c.612+172C>G
921	GPD2	chr2	ENSG00000115159	Intron	c.2181-60C>G
922	GPHN	chr14	ENSG00000171723	Missense	c.171G>C
923	GPNMB	chr7	ENSG00000136235	5'Flank	
924	GPR160	chr3	ENSG00000173890	Silent	c.702G>C
925	GPR162	chr12	ENSG00000250510	Missense	c.779C>G
926	GPR173	chrX	ENSG00000184194	3'UTR	c.*1618G>C
927	GPR61	chr1	ENSG00000156097	3'UTR	c.*2048G>A
928	GPRC5C	chr17	ENSG00000170412	Silent	c.69G>A
929	GPRIN2	chr10	ENSG00000204175	Missense	c.927G>T
930	GRAMD4	chr22	ENSG00000075240	3'UTR	c.*1989C>T
931	GRHL1	chr2	ENSG00000134317	Intron	c.20+1150C>G
932	GRIA1	chr5	ENSG00000155511	3'UTR	c.*784G>C
933	GRIK3	chr1	ENSG00000163873	3'UTR	c.*5696G>C
934	GRIK3	chr1	ENSG00000163873	3'UTR	c.*5562G>A
935	GRIK3	chr1	ENSG00000163873	3'UTR	c.*4511G>A
936	GRIN2B	chr12	ENSG00000273079	Silent	c.1461G>A
937	GRK4	chr4	ENSG00000125388	3'UTR	c.*1309G>C
938	GRM5	chr11	ENSG00000168959	Silent	c.2460G>A
939	GSDMB	chr17	ENSG00000073605	Missense	c.348G>C
940	GSE1	chr16	ENSG00000131149	Silent	c.2523G>A
941	GSK3B	chr3	ENSG00000082701	3'UTR	c.*843G>C
942	GSS	chr20	ENSG00000100983	3'UTR	c.*676G>A
943	GTDC1	chr2	ENSG00000121964	Missense	c.667G>A
944	GTF2A1	chr14	ENSG00000165417	3'UTR	c.*2572C>G
945	GTF2F1	chr19	ENSG00000125651	3'UTR	c.*581C>A
946	GTPBP8	chr3	ENSG00000163607	Silent	c.69G>C
947	GTPBP8	chr3	ENSG00000163607	Missense	c.103G>A
948	GUCY1A1	chr4	ENSG00000164116	Missense	c.1694C>A
949	GYS2	chr12	ENSG00000111713	Intron	c.1169+41G>A
950	H1FX	chr3	ENSG00000184897	3'Flank	
951	H1FX	chr3	ENSG00000184897	3'UTR	c.*289C>T
952	HAAO	chr2	ENSG00000162882	Silent	c.531C>T

Number	Gene name	Chromosome	Gene_ID	Mutation	Nucleotide substitution
953	HAAO	chr2	ENSG00000162882	Intron	c.80+36C>T
954	HAND2-AS1	chr4	ENSG00000237125	RNA	n.641C>T
955	HAP1	chr17	ENSG00000173805	3'UTR	c.*1853C>G
956	HAPLN2	chr1	ENSG00000132702	Silent	c.483C>T
957	HBA1	chr16	ENSG00000206172	3'UTR	c.*41C>A
958	HBD	chr11	ENSG00000223609	Missense	c.152C>G
959	HBM	chr16	ENSG00000206177	Intron	c.298-21C>G
960	HCFC2	chr12	ENSG00000111727	Missense	c.370C>G
961	HDAC11	chr3	ENSG00000163517	Intron	c.490-214G>C
962	HDAC5	chr17	ENSG00000108840	Missense	c.2707C>G
963	HDC	chr15	ENSG00000140287	5'Flank	
964	HDGF	chr1	ENSG00000143321	Silent	c.66C>G
965	HEATR3	chr16	ENSG00000155393	Intron	c.139-26C>G
966	HEATR5B	chr2	ENSG00000008869	Silent	c.1302C>T
967	HECTD1	chr14	ENSG00000092148	Missense	c.5689G>C
968	HECTD4	chr12	ENSG00000173064	Splice region	c.7230+3G>C
969	HEG1	chr3	ENSG00000173706	Missense	c.2714G>T
970	HELQ	chr4	ENSG00000163312	Intron	c.1662+62T>G
971	HELQ	chr4	ENSG00000163312	Missense	c.241G>C
972	HELQ	chr4	ENSG00000163312	5'UTR	c.-130G>A
973	HELZ	chr17	ENSG00000198265	Silent	c.5226C>A
974	HERC1	chr15	ENSG00000103657	Missense	c.14380C>G
975	HERC1	chr15	ENSG00000103657	Missense	c.14271C>G
976	HERC3	chr4	ENSG00000138641	Intron	c.778-53G>C
977	HFM1	chr1	ENSG00000162669	Missense	c.3867G>C
978	HGSNAT	chr8	ENSG00000165102	Missense	c.1436C>G
979	HHEX	chr10	ENSG00000152804	Silent	c.666C>A
980	HIC2	chr22	ENSG00000169635	3'UTR	c.*2619G>C
981	HID1	chr17	ENSG00000167861	3'UTR	c.*219C>T
982	HIP1	chr7	ENSG00000127946	Missense	c.982G>C
983	HIP1R	chr12	ENSG00000130787	Nonsense	c.445C>T
984	HIP1R	chr12	ENSG00000130787	3'UTR	c.*1074C>G
985	HIPK2	chr7	ENSG00000064393	3'UTR	c.*9581G>C
986	HIPK2	chr7	ENSG00000064393	3'UTR	c.*7421G>A
987	HIPK3	chr11	ENSG00000110422	Missense	c.1568C>G
988	HIST1H1C	chr6	ENSG00000187837	5'Flank	
989	HIST1H2AG	chr6	ENSG00000196787	3'UTR	c.*1468G>C
990	HIST1H2AH	chr6	ENSG00000274997	Missense	c.89G>C
991	HIST1H3C	chr6	ENSG00000287080	Missense	c.292G>A
992	HIST1H3J	chr6	ENSG00000197153	5'Flank	
993	HIVEP2	chr6	ENSG00000010818	Missense	c.7309G>T
994	HK1	chr10	ENSG00000156515	Missense	c.533G>T
995	HKDC1	chr10	ENSG00000156510	Missense	c.2014G>A
996	HMCN1	chr1	ENSG00000143341	Silent	c.7902C>T
997	HMGA2	chr12	ENSG00000149948	3'UTR	c.*3161C>T
998	HMGCLL1	chr6	ENSG00000146151	3'UTR	c.*1648T>C
999	HMGN2	chr1	ENSG00000198830	Intron	c.91-65G>C
1000	HMX3	chr10	ENSG00000188620	Silent	c.522G>A
1001	HNF4G	chr8	ENSG00000164749	3'Flank	
1002	HNRNPLL	chr2	ENSG00000143889	Intron	c.714+15C>T
1003	HNRNPUL2	chr11	ENSG00000214753	5'UTR	c.-158G>A
1004	HOXD9	chr2	ENSG00000128709	3'UTR	c.*269G>A
1005	HRH1	chr3	ENSG00000196639	Silent	c.445C>T

Number	Gene name	Chromosome	Gene_ID	Mutation	Nucleotide substitution
1006	HRH3	chr20	ENSG00000101180	Silent	c.126G>A
1007	HS3ST1	chr4	ENSG00000002587	3'UTR	c.*5654G>T
1008	HS6ST1	chr2	ENSG00000136720	3'UTR	c.*283C>T
1009	HSD17B13	chr4	ENSG00000170509	Missense	c.670C>G
1010	HSF4	chr16	ENSG00000102878	Intron	c.123+86G>A
1011	HSPA12A	chr10	ENSG00000165868	3'UTR	c.*886C>G
1012	HSPA2	chr14	ENSG00000126803	Missense	c.319G>C
1013	HSPA4L	chr4	ENSG00000164070	Intron	c.-92+96G>C
1014	HSPB6	chr19	ENSG00000004776	Intron	c.-25-207C>G
1015	HTRA1	chr10	ENSG00000166033	Silent	c.1407C>T
1016	HTT	chr4	ENSG00000197386	3'UTR	c.*693C>T
1017	HUWE1	chrX	ENSG00000086758	Silent	c.1728C>G
1018	IDH1	chr2	ENSG00000138413	Missense	c.1039G>C
1019	IDS	chrX	ENSG00000010404	3'UTR	c.*3944C>T
1020	IFI6	chr1	ENSG00000126709	3'UTR	c.*14G>A
1021	IFIT5	chr10	ENSG00000152778	3'UTR	c.*181C>G
1022	IFITM10	chr11	ENSG00000244242	Missense	c.180C>A
1023	IFRD1	chr7	ENSG00000006652	3'UTR	c.*44dup
1024	IFT122	chr3	ENSG00000163913	Silent	c.2487C>G
1025	IFT140	chr16	ENSG00000187535	Missense	c.410G>A
1026	IFT172	chr2	ENSG00000138002	3'UTR	c.*18G>C
1027	IFT172	chr2	ENSG00000138002	3'UTR	c.*7G>A
1028	IFT172	chr2	ENSG00000138002	Missense	c.4822G>C
1029	IFT81	chr12	ENSG00000122970	Intron	c.1848+43C>A
1030	IFT88	chr13	ENSG00000032742	Missense	c.2050G>C
1031	GHV3OR16-1[illegible]	chr16	ENSG00000271178	3'Flank	
1032	IGKC	chr2	ENSG00000211592	Missense	c.299G>C
1033	IGSF10	chr3	ENSG00000152580	Missense	c.1702G>A
1034	IGSF8	chr1	ENSG00000162729	Intron	c.64+65del
1035	IKZF1	chr7	ENSG00000185811	3'UTR	c.*1295G>A
1036	IKZF3	chr17	ENSG00000161405	3'UTR	c.*5912G>A
1037	IKZF3	chr17	ENSG00000161405	Missense	c.994C>T
1038	IL13RA1	chrX	ENSG00000131724	Intron	c.1192-11del
1039	IL17D	chr13	ENSG00000172458	Missense	c.118G>C
1040	IL27	chr16	ENSG00000197272	3'Flank	
1041	IL6ST	chr5	ENSG00000134352	Splice region	c.1552+4A>C
1042	IL7R	chr5	ENSG00000168685	Missense	c.1144C>T
1043	ILVBL	chr19	ENSG00000105135	Silent	c.186G>A
1044	IMPG2	chr3	ENSG00000081148	Silent	c.507G>A
1045	INPP5E	chr9	ENSG00000148384	5'UTR	c.-32C>G
1046	INSM1	chr20	ENSG00000173404	Missense	c.693C>A
1047	INTS13	chr12	ENSG00000064102	Intron	c.979+21G>A
1048	IP6K1	chr3	ENSG00000176095	3'UTR	c.*2584C>G
1049	IP6K1	chr3	ENSG00000176095	3'UTR	c.*1419C>G
1050	IP6K1	chr3	ENSG00000176095	3'UTR	c.*623C>T
1051	IPO4	chr14	ENSG00000196497	Silent	c.3144G>A
1052	IQCB1	chr3	ENSG00000173226	Intron	c.1129+15C>G
1053	IQCG	chr3	ENSG00000114473	Missense	c.130G>C
1054	IQSEC2	chrX	ENSG00000124313	Missense	c.1064C>T
1055	IRAK3	chr12	ENSG00000090376	Missense	c.34T>A
1056	IRAK3	chr12	ENSG00000090376	3'UTR	c.*4549C>G
1057	IRS2	chr13	ENSG00000185950	Missense	c.1226G>A
1058	IRX5	chr16	ENSG00000176842	5'UTR	c.-63C>T

Number	Gene name	Chromosome	Gene_ID	Mutation	Nucleotide substitution
1059	ISL2	chr15	ENSG00000159556	3'UTR	c.*32C>G
1060	ISY1	chr3	ENSG00000240682	3'UTR	c.*1996C>T
1061	ISY1	chr3	ENSG00000240682	3'UTR	c.*1783G>T
1062	ITCH	chr20	ENSG00000078747	Missense	c.2370G>C
1063	ITGA1	chr5	ENSG00000213949	Missense	c.779A>G
1064	ITGA1	chr5	ENSG00000213949	Nonsense	c.2050C>T
1065	ITGA5	chr12	ENSG00000161638	Missense	c.2737G>C
1066	ITGA8	chr10	ENSG00000077943	3'UTR	c.*373G>A
1067	ITGA8	chr10	ENSG00000077943	3'UTR	c.*294G>C
1068	ITGA9	chr3	ENSG00000144668	3'UTR	c.*1616G>C
1069	ITGB1	chr10	ENSG00000150093	Intron	c.2332-48A>C
1070	ITGB4	chr17	ENSG00000132470	Missense	c.51G>C
1071	ITGB6	chr2	ENSG00000115221	Missense	c.1465C>T
1072	ITPKA	chr15	ENSG00000137825	3'Flank	
1073	ITPR1	chr3	ENSG00000150995	Intron	c.1961+61G>C
1074	ITPR2	chr12	ENSG00000123104	Missense	c.3831C>G
1075	ITSN1	chr21	ENSG00000205726	3'UTR	c.*3018G>C
1076	IYD	chr6	ENSG00000009765	3'UTR	c.*5363_*5372del
1077	JAK3	chr19	ENSG00000105639	Intron	c.421-25G>A
1078	JAKMIP1	chr4	ENSG00000152969	Silent	c.1515C>A
1079	JCAD	chr10	ENSG00000165757	Missense	c.1931G>C
1080	JMJD1C	chr10	ENSG00000171988	Missense	c.7359C>A
1081	JPT2	chr16	ENSG00000206053	Intron	c.45-1976C>T
1082	JUN	chr1	ENSG00000177606	3'UTR	c.*242T>G
1083	KALRN	chr3	ENSG00000160145	Intron	c.67+30G>C
1084	KALRN	chr3	ENSG00000160145	3'UTR	c.*589C>T
1085	KANK4	chr1	ENSG00000132854	Missense	c.1398G>C
1086	KAT5	chr11	ENSG00000172977	Missense	c.1422C>G
1087	KATNB1	chr16	ENSG00000140854	Intron	c.391-29C>G
1088	KCNAB1	chr3	ENSG00000169282	5'UTR	c.-828C>A
1089	KCNC1	chr11	ENSG00000129159	3'UTR	c.*319C>G
1090	KCND1	chrX	ENSG00000102057	Missense	c.1811C>A
1091	KCNE4	chr2	ENSG00000152049	Silent	c.618C>G
1092	KCNIP4	chr4	ENSG00000185774	5'UTR	c.-39G>A
1093	KCNJ13	chr2	ENSG00000115474	3'UTR	c.*562G>C
1094	KCNJ15	chr21	ENSG00000157551	Intron	c.-398-48543G>A
1095	KCNJ4	chr22	ENSG00000168135	Silent	c.45G>A
1096	KCNK10	chr14	ENSG00000100433	3'UTR	c.*537G>A
1097	KCNK9	chr8	ENSG00000169427	Silent	c.795C>A
1098	KCNMA1	chr10	ENSG00000156113	Intron	c.1335-17C>T
1099	KCNMB2	chr3	ENSG00000197584	Intron	c.-67-19C>A
1100	KCNN2	chr5	ENSG00000080709	Missense	c.521C>G
1101	KCNQ1	chr11	ENSG00000053918	Intron	c.387-6496G>A
1102	KCTD12	chr13	ENSG00000178695	Silent	c.105C>T
1103	KCTD3	chr1	ENSG00000136636	Intron	c.1021+27C>T
1104	KCTD4	chr13	ENSG00000180332	Missense	c.220G>A
1105	KCTD7	chr7	ENSG00000243335	5'UTR	c.-159G>C
1106	KDM2B	chr12	ENSG00000089094	Missense	c.25G>A
1107	KDM6A	chrX	ENSG00000147050	Intron	c.975-30G>A
1108	KDR	chr4	ENSG00000128052	Splice region	c.3848+4A>C
1109	KHDC1L	chr6	ENSG00000256980	3'UTR	c.*110C>G
1110	KHDC1L	chr6	ENSG00000256980	Silent	c.24C>T
1111	KHDRBS1	chr1	ENSG00000121774	5'Flank	

Number	Gene name	Chromosome	Gene_ID	Mutation	Nucleotide substitution
1112	KIAA0319	chr6	ENSG00000137261	Missense	c.791C>G
1113	KIAA1549	chr7	ENSG00000122778	3'UTR	c.*4897C>G
1114	KIAA1549	chr7	ENSG00000122778	3'UTR	c.*3448C>T
1115	KIAA1549	chr7	ENSG00000122778	3'UTR	c.*1002C>T
1116	KIAA1958	chr9	ENSG00000165185	3'UTR	c.*7028G>T
1117	KIAA2012	chr2	ENSG00000182329	Splice region	c.2491+7G>C
1118	KIF11	chr10	ENSG00000138160	Missense	c.1465G>C
1119	KIF11	chr10	ENSG00000138160	3'UTR	c.*713C>G
1120	KIF13B	chr8	ENSG00000197892	Missense	c.1032C>A
1121	KIF13B	chr8	ENSG00000197892	Missense	c.707C>A
1122	KIF13B	chr8	ENSG00000197892	Silent	c.687C>T
1123	KIF16B	chr20	ENSG00000089177	Missense	c.2164G>A
1124	KIF19	chr17	ENSG00000196169	Intron	c.121-1001G>C
1125	KIF1A	chr2	ENSG00000130294	Splice region	c.3729-3C>G
1126	KIF1B	chr1	ENSG00000054523	Missense	c.520C>T
1127	KIF1B	chr1	ENSG00000054523	Splice region	c.1223-7C>G
1128	KIF1B	chr1	ENSG00000054523	3'UTR	c.*2191C>G
1129	KIF3C	chr2	ENSG00000084731	Missense	c.568G>C
1130	KIFC3	chr16	ENSG00000140859	Intron	c.316-46G>A
1131	KIT	chr4	ENSG00000157404	Missense	c.2515G>A
1132	KLB	chr4	ENSG00000134962	Missense	c.173G>C
1133	KLC4	chr6	ENSG00000137171	3'UTR	c.*72C>T
1134	KLF11	chr2	ENSG00000172059	3'UTR	c.*240G>C
1135	KLF17	chr1	ENSG00000171872	Missense	c.361C>G
1136	KLF3	chr4	ENSG00000109787	3'UTR	c.*1658C>T
1137	KLHDC2	chr14	ENSG00000165516	5'Flank	
1138	KLHL22	chr22	ENSG00000099910	Intron	c.228-41C>G
1139	KLHL30	chr2	ENSG00000168427	3'UTR	c.*202G>A
1140	KLHL36	chr16	ENSG00000135686	3'UTR	c.*543G>C
1141	KLK1	chr19	ENSG00000167748	Missense	c.725C>T
1142	KLK10	chr19	ENSG00000129451	Intron	c.270-14C>G
1143	KLLN	chr10	ENSG00000227268	Missense	c.479G>C
1144	KMT2D	chr12	ENSG00000167548	Intron	c.16053-40G>C
1145	KNL1	chr15	ENSG00000137812	Missense	c.1340C>G
1146	KNTC1	chr12	ENSG00000184445	Missense	c.1733G>C
1147	KPNA1	chr3	ENSG00000114030	3'UTR	c.*691G>C
1148	KPNA1	chr3	ENSG00000114030	Intron	c.1122+25C>G
1149	KPRP	chr1	ENSG00000203786	Missense	c.772C>A
1150	KPTN	chr19	ENSG00000118162	Silent	c.153C>G
1151	KRBOX1	chr3	ENSG00000240747	5'UTR	c.-456C>T
1152	KRT17	chr17	ENSG00000128422	3'UTR	c.*6C>T
1153	KRT71	chr12	ENSG00000139648	Intron	c.657-14C>T
1154	KRTAP19-8	chr21	ENSG00000206102	3'UTR	c.*62C>T
1155	KTN1	chr14	ENSG00000126777	5'UTR	c.-243C>G
1156	KY	chr3	ENSG00000174611	Intron	c.1090+167G>A
1157	KYAT3	chr1	ENSG00000137944	Intron	c.-1-61C>G
1158	L1TD1	chr1	ENSG00000240563	Missense	c.1840G>A
1159	L2HGDH	chr14	ENSG00000087299	3'UTR	c.*1261G>A
1160	LAMC2	chr1	ENSG00000058085	Silent	c.1404C>T
1161	LAMP1	chr13	ENSG00000185896	Intron	c.877-16C>A
1162	LAT2	chr7	ENSG00000086730	Intron	c.178+52C>G
1163	LCOR	chr10	ENSG00000196233	3'UTR	c.*4508G>C
1164	LCOR	chr10	ENSG00000196233	Missense	c.1445G>T

Number	Gene name	Chromosome	Gene_ID	Mutation	Nucleotide substitution
1165	LCOR	chr10	ENSG00000196233	Missense	c.2209C>G
1166	LCOR	chr10	ENSG00000196233	Missense	c.2254G>A
1167	LCOR	chr10	ENSG00000196233	Missense	c.2565G>C
1168	LCOR	chr10	ENSG00000196233	Missense	c.2775G>C
1169	LCOR	chr10	ENSG00000196233	Nonsense	c.3673C>T
1170	LCORL	chr4	ENSG00000178177	Missense	c.5284C>G
1171	LCP2	chr5	ENSG00000043462	5'UTR	c.-12G>T
1172	LDB1	chr10	ENSG00000198728	3'Flank	
1173	LDB3	chr10	ENSG00000122367	Silent	c.1626C>T
1174	LDLR	chr19	ENSG00000130164	3'UTR	c.*719G>A
1175	LEKR1	chr3	ENSG00000197980	Intron	c.264-32103C>G
1176	LEPR	chr1	ENSG00000116678	Silent	c.3336C>T
1177	LERFS	chr9	ENSG00000234665	Intron	n.445-3148C>G
1178	LGALS3BP	chr17	ENSG00000108679	3'UTR	c.*53C>G
1179	LHPP	chr10	ENSG00000107902	3'UTR	c.*154C>T
1180	LIF	chr22	ENSG00000128342	3'UTR	c.*1966C>G
1181	LIF	chr22	ENSG00000128342	3'UTR	c.*1501G>C
1182	LIG1	chr19	ENSG00000105486	Intron	c.243+33G>A
1183	LIMK1	chr7	ENSG00000106683	Intron	c.153-3639C>G
1184	LIN54	chr4	ENSG00000189308	3'UTR	c.*2819G>C
1185	LIN7B	chr19	ENSG00000104863	Missense	c.271C>T
1186	LINC00636	chr3	ENSG00000240423	RNA	n.594G>C
1187	LINC01405	chr12	ENSG00000185847	RNA	n.185C>G
1188	LINC01804	chr2	ENSG00000231031	Intron	n.283-38G>A
1189	LINC02520	chr6	ENSG00000204110	3'Flank	
1190	LIPE	chr19	ENSG00000079435	Silent	c.2085G>A
1191	LLGL2	chr17	ENSG00000073350	Missense	c.1233G>C
1192	LNPEP	chr5	ENSG00000113441	3'UTR	c.*3608C>G
1193	LONP2	chr16	ENSG00000102910	Missense	c.1208G>A
1194	LONP2	chr16	ENSG00000102910	3'UTR	c.*2757G>C
1195	LOX	chr5	ENSG00000113083	3'UTR	c.*2991T>G
1196	LPA	chr6	ENSG00000198670	Missense	c.5075G>C
1197	LPIN3	chr20	ENSG00000132793	Missense	c.1156T>G
1198	LPP	chr3	ENSG00000145012	3'UTR	c.*2123G>A
1199	LPP	chr3	ENSG00000145012	3'UTR	c.*5255G>C
1200	LPP	chr3	ENSG00000145012	3'UTR	c.*13523C>T
1201	LPP	chr3	ENSG00000145012	3'UTR	c.*14279G>C
1202	LRCH1	chr13	ENSG00000136141	Missense	c.331G>A
1203	LRCH3	chr3	ENSG00000186001	Intron	c.2053-3038T>G
1204	LRFN5	chr14	ENSG00000165379	Missense	c.376C>T
1205	LRIT2	chr10	ENSG00000204033	Nonsense	c.430G>T
1206	LRP1	chr12	ENSG00000123384	Missense	c.6392C>T
1207	LRP10	chr14	ENSG00000197324	Intron	c.1425-58G>C
1208	LRP11	chr6	ENSG00000120256	Missense	c.535A>G
1209	LRP8	chr1	ENSG00000157193	Silent	c.1011G>A
1210	LRRC27	chr10	ENSG00000148814	Intron	c.*17-33C>A
1211	LRRC40	chr1	ENSG00000066557	Missense	c.532G>A
1212	LRRC41	chr1	ENSG00000132128	Missense	c.1048G>C
1213	LRRD1	chr7	ENSG00000240720	3'UTR	c.*916G>C
1214	LRRN4	chr20	ENSG00000125872	Silent	c.168G>T
1215	LSG1	chr3	ENSG00000041802	Intron	c.1544-29G>C
1216	LTBP1	chr2	ENSG00000049323	Missense	c.162C>G
1217	LTBP3	chr11	ENSG00000168056	Missense	c.3253G>C

Number	Gene name	Chromosome	Gene_ID	Mutation	Nucleotide substitution
1218	LTBP4	chr19	ENSG00000090006	Missense	c.198G>C
1219	LTF	chr3	ENSG00000012223	Intron	c.43+34G>C
1220	LTK	chr15	ENSG00000062524	Intron	c.1541+203C>G
1221	LUC7L	chr16	ENSG00000007392	Intron	c.157-11C>G
1222	LUC7L2	chr7	ENSG00000146963	3'UTR	c.*1006C>T
1223	LVRN	chr5	ENSG00000172901	Intron	c.2757-1352C>G
1224	LVRN	chr5	ENSG00000172901	Intron	c.2757-703C>G
1225	LY6K	chr8	ENSG00000160886	3'UTR	c.*4G>A
1226	LY9	chr1	ENSG00000122224	Splice region	c.1498+8C>A
1227	LYG1	chr2	ENSG00000144214	Missense	c.196C>G
1228	LYG1	chr2	ENSG00000144214	5'Flank	
1229	LYSMD2	chr15	ENSG00000140280	Silent	c.636C>G
1230	LYZL1	chr10	ENSG00000120563	Intron	c.437-13C>T
1231	LYZL1	chr10	ENSG00000120563	Intron	c.437-9C>G
1232	LZIC	chr1	ENSG00000162441	3'UTR	c.*786G>A
1233	LZTS2	chr10	ENSG00000107816	5'UTR	c.-2822G>C
1234	MACF1	chr1	ENSG00000127603	Missense	c.2245G>A
1235	MACF1	chr1	ENSG00000127603	Silent	c.2715G>A
1236	MAFG	chr17	ENSG00000197063	Missense	c.208G>A
1237	MAGEE1	chrX	ENSG00000198934	Missense	c.776C>T
1238	MAN1A1	chr6	ENSG00000111885	3'UTR	c.*1286G>C
1239	MAP1A	chr15	ENSG00000166963	Missense	c.633G>C
1240	MAP1LC3B	chr16	ENSG00000140941	5'UTR	c.-322G>A
1241	MAP1LC3B	chr16	ENSG00000140941	Missense	c.52G>C
1242	MAP2K5	chr15	ENSG00000137764	Missense	c.845G>C
1243	MAP4	chr3	ENSG00000047849	Silent	c.1206C>T
1244	MAP4	chr3	ENSG00000047849	Missense	c.889G>A
1245	MAP4K5	chr14	ENSG00000012983	Missense	c.2467G>C
1246	MAP6	chr11	ENSG00000171533	3'UTR	c.*55C>T
1247	MAP7D1	chr1	ENSG00000116871	Intron	c.2117-42C>G
1248	MAP7D3	chrX	ENSG00000129680	Missense	c.521C>G
1249	MAP9	chr4	ENSG00000164114	Missense	c.1115C>T
1250	MAPK8IP1	chr11	ENSG00000121653	Missense	c.1000G>A
1251	MARCH9	chr12	ENSG00000139266	5'UTR	c.-327C>T
1252	MARCO	chr2	ENSG00000019169	Intron	c.568+20G>C
1253	MARK2	chr11	ENSG00000072518	Intron	c.55-6093G>C
1254	MARK4	chr19	ENSG00000007047	Silent	c.732C>T
1255	MASP1	chr3	ENSG00000127241	Missense	c.1433C>G
1256	MAST2	chr1	ENSG00000086015	Missense	c.4318G>A
1257	MAST4	chr5	ENSG00000069020	Missense	c.1828G>A
1258	MAT2B	chr5	ENSG00000038274	3'UTR	c.*118C>G
1259	MAVS	chr20	ENSG00000088888	Silent	c.48C>T
1260	MAX	chr14	ENSG00000125952	3'UTR	c.*1350C>G
1261	MAZ	chr16	ENSG00000103495	Missense	c.541G>C
1262	MAZ	chr16	ENSG00000103495	Missense	c.1032G>C
1263	MBD3L1	chr19	ENSG00000170948	Nonsense	c.251C>G
1264	MBD6	chr12	ENSG00000166987	Silent	c.1245G>C
1265	MBD6	chr12	ENSG00000166987	Nonsense	c.2003C>G
1266	MBD6	chr12	ENSG00000166987	Silent	c.2334C>A
1267	MBOAT2	chr2	ENSG00000143797	Missense	c.1485G>C
1268	MCFD2	chr2	ENSG00000180398	3'UTR	c.*2867G>A
1269	MCIDAS	chr5	ENSG00000234602	Intron	c.309+53C>T
1270	MCM2	chr3	ENSG00000073111	Missense	c.2584G>A

Number	Gene name	Chromosome	Gene_ID	Mutation	Nucleotide substitution
1271	MCM4	chr8	ENSG00000104738	3'UTR	c.*1082C>T
1272	MCMBP	chr10	ENSG00000197771	Intron	c.1548+41G>T
1273	MCMBP	chr10	ENSG00000197771	5'UTR	c.-160G>C
1274	MDGA1	chr6	ENSG00000112139	5'UTR	c.-114C>G
1275	MDH1B	chr2	ENSG00000138400	Missense	c.280G>C
1276	MDK	chr11	ENSG00000110492	5'UTR	c.-32G>C
1277	MECOM	chr3	ENSG00000085276	3'UTR	c.*1202G>A
1278	MED13	chr17	ENSG00000108510	Splice region	c.1010-3C>T
1279	MED13	chr17	ENSG00000108510	5'UTR	c.-73C>G
1280	MED16	chr19	ENSG00000175221	Missense	c.1085C>T
1281	MED24	chr17	ENSG00000008838	Silent	c.1158G>A
1282	MED24	chr17	ENSG00000008838	Missense	c.924G>C
1283	MEF2B	chr19	ENSG00000213999	Missense	c.727C>T
1284	MEF2D	chr1	ENSG00000116604	3'UTR	c.*1440C>G
1285	MEGF10	chr5	ENSG00000145794	Silent	c.201T>C
1286	MEGF10	chr5	ENSG00000145794	Intron	c.1693+110G>A
1287	MEGF11	chr15	ENSG00000157890	3'UTR	c.*135G>C
1288	MEIOB	chr16	ENSG00000162039	Splice region	c.1308C>T
1289	MEPE	chr4	ENSG00000152595	Missense	c.1534G>A
1290	METAP1	chr4	ENSG00000164024	Missense	c.700G>A
1291	METRN	chr16	ENSG00000103260	Silent	c.648C>G
1292	METTL25	chr12	ENSG00000127720	Silent	c.1533C>G
1293	MFN1	chr3	ENSG00000171109	Silent	c.954G>A
1294	MFSD1	chr3	ENSG00000118855	Intron	c.864-406G>C
1295	MFSD1	chr3	ENSG00000118855	Intron	:.864-388_864-387delinsCC
1296	MFSD13A	chr10	ENSG00000138111	3'Flank	
1297	MFSD14A	chr1	ENSG00000156875	3'UTR	c.*1125C>T
1298	MFSD4B	chr6	ENSG00000173214	5'UTR	c.-159G>C
1299	MFSD6	chr2	ENSG00000151690	Missense	c.548C>T
1300	MFSD6	chr2	ENSG00000151690	Intron	c.2172+41C>G
1301	MFSD9	chr2	ENSG00000135953	Silent	c.882G>A
1302	MGA	chr15	ENSG00000174197	Missense	c.1844G>T
1303	MGA	chr15	ENSG00000174197	Missense	c.2701C>G
1304	MGAT4EP	chr1	ENSG00000184774	RNA	n.1497G>A
1305	MGRN1	chr16	ENSG00000102858	5'UTR	c.-22C>G
1306	MID1	chrX	ENSG00000101871	Missense	c.898G>C
1307	MIF4GD	chr17	ENSG00000125457	Silent	c.663C>G
1308	MIIP	chr1	ENSG00000116691	Missense	c.1151C>G
1309	MIR4771-1	chr2	ENSG00000264793	3'Flank	
1310	MIR5692B	chr21	ENSG00000264580	3'Flank	
1311	MLANA	chr9	ENSG00000120215	Missense	c.241C>G
1312	MLH3	chr14	ENSG00000119684	3'UTR	c.*599G>C
1313	MLLT1	chr19	ENSG00000130382	Silent	c.867C>T
1314	MLLT3	chr9	ENSG00000171843	3'UTR	c.*596C>A
1315	MLLT3	chr9	ENSG00000171843	Intron	c.194-45414C>G
1316	MLXIP	chr12	ENSG00000175727	3'UTR	c.*1519C>T
1317	MMACHC	chr1	ENSG00000132763	Missense	c.310G>C
1318	MMACHC	chr1	ENSG00000132763	3'UTR	c.*2311C>A
1319	MMP1	chr11	ENSG00000196611	Missense	c.403G>A
1320	MMP14	chr14	ENSG00000157227	3'UTR	c.*257G>A
1321	MMRN1	chr4	ENSG00000138722	3'UTR	c.*587C>G
1322	MMRN1	chr4	ENSG00000138722	3'UTR	c.*804C>T
1323	MMS22L	chr6	ENSG00000146263	3'UTR	c.*4549G>A

Number	Gene name	Chromosome	Gene_ID	Mutation	Nucleotide substitution
1324	MN1	chr22	ENSG00000169184	3'UTR	c.*1980C>T
1325	MNX1	chr7	ENSG00000130675	Silent	c.33T>C
1326	MOCOS	chr18	ENSG00000075643	3'UTR	c.*940C>T
1327	MOCS3	chr20	ENSG00000124217	Nonsense	c.419C>A
1328	MOGAT3	chr7	ENSG00000106384	Silent	c.402C>T
1329	MON2	chr12	ENSG00000061987	Intron	c.2365-48C>T
1330	MOV10L1	chr22	ENSG00000073146	Missense	c.1504G>C
1331	MOV10L1	chr22	ENSG00000073146	Missense	c.1567G>A
1332	MPEG1	chr11	ENSG00000197629	Missense	c.1939G>A
1333	MPI	chr15	ENSG00000178802	3'UTR	c.*1145C>A
1334	MR1	chr1	ENSG00000153029	3'UTR	c.*112G>T
1335	MR1	chr1	ENSG00000153029	3'UTR	c.*729G>C
1336	MRGPRX4	chr11	ENSG00000179817	5'UTR	c.-352C>T
1337	MROH1	chr8	ENSG00000179832	Missense	c.2011G>C
1338	MROH2B	chr5	ENSG00000171495	Missense	c.3325G>A
1339	MROH8	chr20	ENSG00000101353	Missense	c.445G>C
1340	MRPL11	chr11	ENSG00000174547	Silent	c.33C>T
1341	MRPL11	chr11	ENSG00000174547	5'UTR	c.-48C>A
1342	MRPL11	chr11	ENSG00000174547	5'Flank	
1343	MRPL18	chr6	ENSG00000112110	Intron	c.52+84C>G
1344	MRPL30	chr2	ENSG00000185414	Intron	c.-27-282C>T
1345	MRPS26	chr20	ENSG00000125901	Silent	c.366G>A
1346	MRPS35	chr12	ENSG00000061794	Frame_Shift_Ins	c.121dup
1347	MRTFB	chr16	ENSG00000186260	Missense	c.466G>C
1348	MT3	chr16	ENSG00000087250	Missense	c.163G>C
1349	MTA1	chr14	ENSG00000182979	Intron	c.1813+880C>G
1350	MTERF1	chr7	ENSG00000127989	Missense	c.46A>C
1351	MTFR1L	chr1	ENSG00000117640	5'UTR	c.-1211G>C
1352	MTHFR	chr1	ENSG00000177000	3'UTR	c.*3085G>A
1353	MTRF1L	chr6	ENSG00000112031	Missense	c.229G>A
1354	MUC16	chr19	ENSG00000181143	Missense	c.42778G>A
1355	MUC16	chr19	ENSG00000181143	Missense	c.36653C>A
1356	MUC16	chr19	ENSG00000181143	Missense	c.30806C>T
1357	MUC5AC	chr11	ENSG00000215182	Nonsense	c.1592C>G
1358	MUC5AC	chr11	ENSG00000215182	Silent	c.1755C>T
1359	MUC5AC	chr11	ENSG00000215182	Silent	c.15462C>G
1360	MUC5B	chr11	ENSG00000117983	Silent	c.1146C>T
1361	MUCL1	chr12	ENSG00000172551	3'UTR	c.*22C>A
1362	MVB12B	chr9	ENSG00000196814	Intron	c.874-76G>C
1363	MVD	chr16	ENSG00000167508	Splice region	c.142-3C>A
1364	MX2	chr21	ENSG00000183486	Missense	c.1832C>G
1365	MXD4	chr4	ENSG00000123933	Intron	c.65-21G>A
1366	MXRA5	chrX	ENSG00000101825	Missense	c.2932T>A
1367	MYBPC3	chr11	ENSG00000134571	Silent	c.1785C>T
1368	MYBPHL	chr1	ENSG00000221986	Intron	c.570+33G>A
1369	MYBPHL	chr1	ENSG00000221986	Silent	c.177G>A
1370	MYH14	chr19	ENSG00000105357	Silent	c.225G>A
1371	MYH15	chr3	ENSG00000144821	Missense	c.3130G>A
1372	MYL9	chr20	ENSG00000101335	3'UTR	c.*351G>T
1373	MYLK	chr3	ENSG00000065534	Intron	c.166-25T>G
1374	MYLPF	chr16	ENSG00000180209	Missense	c.193G>A
1375	MYO16	chr13	ENSG00000041515	3'UTR	c.*238C>T
1376	MYO18B	chr22	ENSG00000133454	Missense	c.4582G>C

Number	Gene name	Chromosome	Gene_ID	Mutation	Nucleotide substitution
1377	MYO1B	chr2	ENSG00000128641	3'UTR	c.*923C>T
1378	MYO9A	chr15	ENSG00000066933	Missense	c.1576C>G
1379	MYOF	chr10	ENSG00000138119	Intron	c.346-61G>C
1380	NAA15	chr4	ENSG00000164134	3'UTR	c.*808C>G
1381	NAA30	chr14	ENSG00000139977	3'UTR	c.*945C>T
1382	NAAA	chr4	ENSG00000138744	Intron	c.903-78C>T
1383	NAALADL2	chr3	ENSG00000177694	3'UTR	c.*1702G>C
1384	NAB2	chr12	ENSG00000166886	Missense	c.1115G>A
1385	NABP1	chr2	ENSG00000173559	Intron	c.-149-261C>G
1386	NACAD	chr7	ENSG00000136274	Missense	c.692C>T
1387	NAE1	chr16	ENSG00000159593	Silent	c.63C>T
1388	NAGA	chr22	ENSG00000198951	Missense	c.906G>A
1389	NANOS1	chr10	ENSG00000188613	3'UTR	c.*120C>T
1390	NAT10	chr11	ENSG00000135372	Missense	c.37C>G
1391	NBEAL1	chr2	ENSG00000144426	Missense	c.2026G>A
1392	NBEAL1	chr2	ENSG00000144426	3'UTR	c.*2195C>G
1393	NBL1	chr1	ENSG00000158747	5'UTR	c.-6G>A
1394	NCAN	chr19	ENSG00000130287	Intron	c.1661-36G>C
1395	NCF1	chr7	ENSG00000158517	Intron	c.73-44C>G
1396	NCKAP5	chr2	ENSG00000176771	Missense	c.4552C>T
1397	NCL	chr2	ENSG00000115053	Missense	c.700G>A
1398	NCLN	chr19	ENSG00000125912	Silent	c.867G>A
1399	NCMAP	chr1	ENSG00000184454	3'UTR	c.*2529C>G
1400	NCMAP	chr1	ENSG00000184454	3'UTR	c.*2603G>C
1401	NCOR1	chr17	ENSG00000141027	Intron	c.909+83G>C
1402	NCOR2	chr12	ENSG00000196498	Intron	c.312-13C>G
1403	NCS1	chr9	ENSG00000107130	Intron	c.308-22C>G
1404	NDRG3	chr20	ENSG00000101079	5'UTR	c.-14C>T
1405	NDRG4	chr16	ENSG00000103034	Intron	c.72+87G>A
1406	NDUFAF7	chr2	ENSG00000003509	Intron	c.217-46T>G
1407	NDUFV3	chr21	ENSG00000160194	Missense	c.325G>T
1408	NDUFV3	chr21	ENSG00000160194	Missense	c.583G>C
1409	NDUFV3	chr21	ENSG00000160194	Missense	c.664G>C
1410	NDUFV3	chr21	ENSG00000160194	Missense	c.769G>C
1411	NDUFV3	chr21	ENSG00000160194	Missense	c.1122G>C
1412	NEDD4	chr15	ENSG00000069869	Missense	c.2018G>T
1413	NEK1	chr4	ENSG00000137601	Missense	c.1041G>C
1414	NEK7	chr1	ENSG00000151414	3'UTR	c.*2348G>A
1415	NEK8	chr17	ENSG00000160602	Missense	c.147G>C
1416	NELL2	chr12	ENSG00000184613	Intron	c.125-145C>G
1417	NEUROD2	chr17	ENSG00000171532	Silent	c.586C>T
1418	NFASC	chr1	ENSG00000163531	Intron	c.1391+50C>T
1419	NFIA	chr1	ENSG00000162599	3'UTR	c.*2501C>T
1420	NGEF	chr2	ENSG00000066248	3'UTR	c.*598G>C
1421	NGFR	chr17	ENSG00000064300	3'UTR	c.*942C>G
1422	NIN	chr14	ENSG00000100503	3'UTR	c.*371G>C
1423	NINL	chr20	ENSG00000101004	Intron	c.518-58G>A
1424	NKX6-1	chr4	ENSG00000163623	3'UTR	c.*209C>T
1425	NLGN1	chr3	ENSG00000169760	3'UTR	c.*612G>A
1426	NLGN3	chrX	ENSG00000196338	Splice region	c.1703+8G>C
1427	NLRP5	chr19	ENSG00000171487	Intron	c.2277-18C>G
1428	NMRK1	chr9	ENSG00000106733	3'UTR	c.*294C>G
1429	NOC3L	chr10	ENSG00000173145	Splice region	c.859-3C>G

Number	Gene name	Chromosome	Gene_ID	Mutation	Nucleotide substitution
1430	NORAD	chr20	ENSG00000260032	RNA	n.410C>T
1431	NOX5	chr15	ENSG00000255346	3'UTR	c.*663G>C
1432	NPAS4	chr11	ENSG00000174576	3'UTR	c.*452C>T
1433	NPBWR1	chr8	ENSG00000183729	5'UTR	c.-501C>T
1434	NPDC1	chr9	ENSG00000107281	Silent	c.366G>T
1435	NPHP3	chr3	ENSG00000113971	Intron	c.1351-46C>G
1436	NR2F2	chr15	ENSG00000185551	3'UTR	c.*2110C>G
1437	NR3C2	chr4	ENSG00000151623	Missense	c.2930A>C
1438	NR5A2	chr1	ENSG00000116833	3'UTR	c.*2405G>C
1439	NRBP2	chr8	ENSG00000185189	Intron	c.1382-21C>T
1440	NRG2	chr5	ENSG00000158458	Missense	c.2483G>C
1441	NRG2	chr5	ENSG00000158458	Missense	c.1561G>A
1442	NRL	chr14	ENSG00000129535	3'UTR	c.*678C>T
1443	NRL	chr14	ENSG00000129535	5'UTR	c.-219G>A
1444	NSG2	chr5	ENSG00000170091	3'UTR	c.*184C>G
1445	NSUN6	chr10	ENSG00000241058	5'UTR	c.-364C>A
1446	NT5C2	chr10	ENSG00000076685	Missense	c.471C>G
1447	NT5DC4	chr2	ENSG00000144130	Intron	c.887-23C>G
1448	NT5DC4	chr2	ENSG00000144130	Missense	c.1079G>A
1449	NTMT1	chr9	ENSG00000148335	5'UTR	c.-25G>A
1450	NTRK1	chr1	ENSG00000198400	Silent	c.259C>T
1451	NUAK1	chr12	ENSG00000074590	3'UTR	c.*2945C>G
1452	NUBP1	chr16	ENSG00000103274	Intron	c.328-86C>G
1453	NUDCD3	chr7	ENSG00000015676	3'UTR	c.*3583C>G
1454	NUDCD3	chr7	ENSG00000015676	Intron	c.643-29G>A
1455	NUDT3	chr6	ENSG00000272325	3'UTR	c.*456C>G
1456	NUP155	chr5	ENSG00000113569	Splice region	c.3794-3C>G
1457	NUP188	chr9	ENSG00000095319	Intron	c.2394-25C>T
1458	NUP205	chr7	ENSG00000155561	Intron	c.4231+34C>G
1459	NUP210	chr3	ENSG00000132182	3'UTR	c.*501G>A
1460	NUP210	chr3	ENSG00000132182	Silent	c.4686C>A
1461	NUP214	chr9	ENSG00000126883	Missense	c.94G>A
1462	NUP214	chr9	ENSG00000126883	Silent	c.3225G>T
1463	NXF1	chr11	ENSG00000162231	Missense	c.10G>A
1464	NXN	chr17	ENSG00000167693	3'UTR	c.*489C>G
1465	NXPE2	chr11	ENSG00000204361	Silent	c.120A>G
1466	NYAP1	chr7	ENSG00000166924	Missense	c.811G>A
1467	NYAP1	chr7	ENSG00000166924	Missense	c.2279C>A
1468	NYAP2	chr2	ENSG00000144460	Missense	c.704C>T
1469	OAF	chr11	ENSG00000184232	3'UTR	c.*521G>A
1470	OASL	chr12	ENSG00000135114	3'UTR	c.*66C>T
1471	OBSL1	chr2	ENSG00000124006	Intron	c.2953+731G>T
1472	ODF3L1	chr15	ENSG00000182950	5'Flank	
1473	OFCC1	chr6	ENSG00000181355	RNA	n.1271C>G
1474	OLFM1	chr9	ENSG00000130558	3'UTR	c.*315C>T
1475	OMD	chr9	ENSG00000127083	Missense	c.1066C>G
1476	ONECUT3	chr19	ENSG00000205922	5'Flank	
1477	OOSP1	chr11	ENSG00000284873	Missense	c.326C>G
1478	OPTN	chr10	ENSG00000123240	Intron	c.883-733G>C
1479	OR13C8	chr9	ENSG00000186943	Missense	c.597G>A
1480	OR2L13	chr1	ENSG00000196071	Missense	c.379C>T
1481	OR51B5	chr11	ENSG00000167355	Missense	c.181C>G
1482	OR51E1	chr11	ENSG00000180785	Missense	c.500A>C

Number	Gene name	Chromosome	Gene_ID	Mutation	Nucleotide substitution
1483	OR51I2	chr11	ENSG00000187918	Missense	c.415G>A
1484	OR52L1	chr11	ENSG00000183313	Silent	c.813C>A
1485	OR5A2	chr11	ENSG00000172324	3'UTR	c.*104C>A
1486	OR5H6	chr3	ENSG00000230301	Intron	c.-18-144C>G
1487	OR6C68	chr12	ENSG00000205327	Missense	c.585G>C
1488	ORMDL1	chr2	ENSG00000128699	3'UTR	c.*279C>T
1489	OSBPL1A	chr18	ENSG00000141447	Missense	c.1580C>G
1490	OSBPL3	chr7	ENSG00000070882	Silent	c.765C>T
1491	OTUB1	chr11	ENSG00000167770	Silent	c.819C>T
1492	OTUD3	chr1	ENSG00000169914	3'UTR	c.*3352C>G
1493	OTUD4	chr4	ENSG00000164164	3'UTR	c.*3215C>G
1494	OVOL1	chr11	ENSG00000172818	5'UTR	c.-264G>A
1495	OXSR1	chr3	ENSG00000172939	3'UTR	c.*255G>C
1496	P2RY1	chr3	ENSG00000169860	5'UTR	c.-324C>T
1497	PA2G4	chr12	ENSG00000170515	Intron	c.487-35G>A
1498	PAFAH1B2	chr11	ENSG00000168092	Missense	c.262C>G
1499	PAG1	chr8	ENSG00000076641	3'UTR	c.*301G>A
1500	PAH	chr12	ENSG00000171759	3'UTR	c.*1353G>C
1501	PAIP2B	chr2	ENSG00000124374	3'UTR	c.*1809C>G
1502	PAK2	chr3	ENSG00000180370	3'UTR	c.*3750G>A
1503	PALD1	chr10	ENSG00000107719	Silent	c.2469C>T
1504	PALM	chr19	ENSG00000099864	Intron	c.421-14C>T
1505	PAM	chr5	ENSG00000145730	Missense	c.1375C>T
1506	PAMR1	chr11	ENSG00000149090	Splice region	c.259+8G>C
1507	PANX3	chr11	ENSG00000154143	Nonsense	c.1126G>T
1508	PAPOLG	chr2	ENSG00000115421	Missense	c.619G>A
1509	PAQR6	chr1	ENSG00000160781	Missense	c.41C>T
1510	PARD6G	chr18	ENSG00000178184	Intron	c.296-2820G>A
1511	PARD6G	chr18	ENSG00000178184	5'UTR	c.-41G>A
1512	PARM1	chr4	ENSG00000169116	5'UTR	c.-146C>T
1513	PARP1	chr1	ENSG00000143799	Missense	c.1855G>A
1514	PARP8	chr5	ENSG00000151883	3'UTR	c.*77C>G
1515	PASK	chr2	ENSG00000115687	Silent	c.972G>A
1516	PATL1	chr11	ENSG00000166889	Nonsense	c.13G>T
1517	PATZ1	chr22	ENSG00000100105	Missense	c.893G>A
1518	PATZ1	chr22	ENSG00000100105	5'Flank	
1519	PAWR	chr12	ENSG00000177425	3'UTR	c.*1463G>A
1520	PAX2	chr10	ENSG00000075891	3'UTR	c.*2162G>C
1521	PAX3	chr2	ENSG00000135903	3'UTR	c.*363C>G
1522	PAX5	chr9	ENSG00000196092	3'UTR	c.*5193G>C
1523	PAXIP1-AS2	chr7	ENSG00000214106	RNA	n.301G>A
1524	PBLD	chr10	ENSG00000108187	5'UTR	c.-31dup
1525	PCARE	chr2	ENSG00000179270	3'UTR	c.*1032C>G
1526	PCDH10	chr4	ENSG00000138650	Missense	c.803T>A
1527	PCDH18	chr4	ENSG00000189184	Missense	c.2032G>C
1528	PCDH19	chrX	ENSG00000165194	3'UTR	c.*1881G>T
1529	PCDHB8	chr5	ENSG00000120322	Nonsense	c.2290G>T
1530	PCDHGA5	chr5	ENSG00000253485	Missense	c.1528G>A
1531	PCDHGA5	chr5	ENSG00000253485	Missense	c.1883C>T
1532	PCDHGC4	chr5	ENSG00000242419	Missense	c.1600G>A
1533	PCDHGC5	chr5	ENSG00000240764	Nonsense	c.277G>T
1534	PCNX2	chr1	ENSG00000135749	Missense	c.5570G>A
1535	PCNX4	chr14	ENSG00000126773	Intron	c.168-41C>G

Number	Gene name	Chromosome	Gene_ID	Mutation	Nucleotide substitution
1536	PCYT1B	chrX	ENSG00000102230	3'UTR	c.*3580G>A
1537	PDE10A	chr6	ENSG00000112541	Intron	c.1986-20C>G
1538	PDE4C	chr19	ENSG00000285188	Missense	c.58C>T
1539	PDE4D	chr5	ENSG00000113448	3'UTR	c.*2258G>A
1540	PDE4D	chr5	ENSG00000113448	Intron	c.42+24717C>T
1541	PDE6B	chr4	ENSG00000133256	Silent	c.1764C>T
1542	PDE6B	chr4	ENSG00000133256	Intron	c.2269-26C>G
1543	PDE6D	chr2	ENSG00000156973	Intron	c.-22+507G>A
1544	PDE9A	chr21	ENSG00000160191	Intron	c.219-28C>G
1545	PDE9A	chr21	ENSG00000160191	Intron	c.1768+77G>A
1546	PDGFC	chr4	ENSG00000145431	3'UTR	c.*136C>T
1547	PDGFRA	chr4	ENSG00000134853	Intron	c.2156+421G>T
1548	PDGFRA	chr4	ENSG00000134853	Silent	c.2244G>A
1549	PDP1	chr8	ENSG00000164951	5'UTR	c.-118G>C
1550	PDZK1IP1	chr1	ENSG00000162366	Intron	c.176+24G>A
1551	PEAK1	chr15	ENSG00000173517	3'UTR	c.*582G>C
1552	PER1	chr17	ENSG00000179094	Intron	c.1049-11C>T
1553	PER3	chr1	ENSG00000049246	Missense	c.766G>C
1554	PEX1	chr7	ENSG00000127980	Missense	c.1450G>C
1555	PEX11A	chr15	ENSG00000166821	Missense	c.238C>G
1556	PFKM	chr12	ENSG00000152556	5'UTR	c.-32C>G
1557	PGLS	chr19	ENSG00000130313	Silent	c.426C>T
1558	PGLYRP4	chr1	ENSG00000163218	3'UTR	c.*570C>T
1559	PHACTR1	chr6	ENSG00000112137	Silent	c.702G>T
1560	PHC1P1	chr12	ENSG00000179899	3'Flank	
1561	PHC2	chr1	ENSG00000134686	3'UTR	c.*756G>C
1562	PHEX	chrX	ENSG00000102174	Missense	c.1270G>C
1563	PHF21B	chr22	ENSG00000056487	Intron	c.1038+108G>A
1564	PHF24	chr9	ENSG00000122733	Splice region	c.1107-4C>G
1565	PHKB	chr16	ENSG00000102893	Intron	c.2337-948G>C
1566	PHLDB1	chr11	ENSG00000019144	Missense	c.2917C>T
1567	PHLDB2	chr3	ENSG00000144824	Intron	c.1945-33G>A
1568	PHLDB3	chr19	ENSG00000176531	Intron	c.952-62G>C
1569	PI15	chr8	ENSG00000137558	3'UTR	c.*2326C>G
1570	PICALM	chr11	ENSG00000073921	Missense	c.1237C>G
1571	PIEZO2	chr18	ENSG00000154864	Missense	c.3310G>C
1572	PIGG	chr4	ENSG00000174227	Missense	c.437G>A
1573	PIGQ	chr16	ENSG00000007541	Silent	c.261C>T
1574	PIK3C3	chr18	ENSG00000078142	3'UTR	c.*5771C>G
1575	PIK3CA	chr3	ENSG00000121879	Missense	c.1258T>C
1576	PIM2	chrX	ENSG00000102096	3'UTR	c.*662G>C
1577	PIP5K1B	chr9	ENSG00000107242	Intron	c.1502+306G>C
1578	PIWIL4	chr11	ENSG00000134627	5'Flank	
1579	PKD1L1	chr7	ENSG00000158683	Missense	c.4441G>C
1580	PKD1L1	chr7	ENSG00000158683	Silent	c.1245C>G
1581	PKD1L2	chr16	ENSG00000166473	Intron	c.301+28G>C
1582	PKD2	chr4	ENSG00000118762	Splice region	c.2240+5G>C
1583	PKD2	chr4	ENSG00000118762	3'UTR	c.*718C>G
1584	PKHD1L1	chr8	ENSG00000205038	Splice site	c.10810_10828+13del
1585	PKN1	chr19	ENSG00000123143	Splice region	c.2294-3del
1586	PKNOX1	chr21	ENSG00000160199	3'UTR	c.*1391G>A
1587	PLA2G4E	chr15	ENSG00000188089	Intron	c.394-26G>C
1588	PLAA	chr9	ENSG00000137055	Nonsense	c.38C>A

Number	Gene name	Chromosome	Gene_ID	Mutation	Nucleotide substitution
1589	PLCB1	chr20	ENSG00000182621	Missense	c.1881G>T
1590	PLCB2	chr15	ENSG00000137841	3'UTR	c.*183C>T
1591	PLCE1	chr10	ENSG00000138193	Intron	c.5208+12G>A
1592	PLCL2	chr3	ENSG00000154822	Intron	c.2058-54G>T
1593	PLCL2	chr3	ENSG00000154822	Intron	c.2058-50C>T
1594	PLCL2	chr3	ENSG00000154822	3'UTR	c.*56G>A
1595	PLCXD1	chrX	ENSG00000182378	3'UTR	c.*3948C>T
1596	PLD1	chr3	ENSG00000075651	3'UTR	c.*1484G>C
1597	PLEKHA5	chr12	ENSG00000052126	Silent	c.513C>G
1598	PLEKHG1	chr6	ENSG00000120278	3'UTR	c.*418A>C
1599	PLEKHG6	chr12	ENSG00000008323	Intron	c.1671-18C>G
1600	PLEKHH2	chr2	ENSG00000152527	Nonsense	c.776C>A
1601	PLEKHH2	chr2	ENSG00000152527	Missense	c.920C>G
1602	PLEKHJ1	chr19	ENSG00000104886	Missense	c.145C>T
1603	PLEKHS1	chr10	ENSG00000148735	Missense	c.1291G>C
1604	PLOD3	chr7	ENSG00000106397	Intron	c.1683+83C>T
1605	PLPPR4	chr1	ENSG00000117600	3'UTR	c.*887T>G
1606	PLSCR4	chr3	ENSG00000114698	3'UTR	c.*1579G>T
1607	PLSCR4	chr3	ENSG00000114698	Missense	c.670G>A
1608	PLXDC1	chr17	ENSG00000161381	Missense	c.73G>C
1609	PLXNA4	chr7	ENSG00000221866	3'UTR	c.*6035G>C
1610	PLXNA4	chr7	ENSG00000221866	3'UTR	c.*5964C>G
1611	PM20D2	chr6	ENSG00000146281	Missense	c.106G>C
1612	PMFBP1	chr16	ENSG00000118557	Missense	c.2642G>A
1613	PMS1	chr2	ENSG00000064933	Missense	c.1429C>G
1614	PMS2	chr7	ENSG00000122512	Intron	c.23+50G>A
1615	PMS2	chr7	ENSG00000122512	5'UTR	c.-279G>C
1616	PNKP	chr19	ENSG00000039650	Splice region	c.1189-6C>T
1617	PNMA2	chr8	ENSG00000240694	3'UTR	c.*2391G>C
1618	PODXL2	chr3	ENSG00000114631	Missense	c.496G>C
1619	POF1B	chrX	ENSG00000124429	3'UTR	c.*399C>T
1620	POLA1	chrX	ENSG00000101868	Missense	c.3575C>G
1621	POLD2	chr7	ENSG00000106628	Missense	c.1167T>G
1622	POLE3	chr9	ENSG00000148229	3'UTR	c.*1077C>T
1623	POLR1B	chr2	ENSG00000125630	Intron	c.1746+55C>A
1624	POLR2D	chr2	ENSG00000144231	3'UTR	c.*1514G>A
1625	POLR3A	chr10	ENSG00000148606	3'UTR	c.*2300G>A
1626	POLR3A	chr10	ENSG00000148606	Missense	c.1537G>C
1627	POLR3H	chr22	ENSG00000100413	3'UTR	c.*2187_*2191dup
1628	POLRMTP1	chr17	ENSG00000266066	RNA	n.423G>C
1629	POU2F2	chr19	ENSG00000028277	Missense	c.547G>C
1630	PPAN	chr19	ENSG00000130810	5'UTR	c.-59G>A
1631	PPARGC1B	chr5	ENSG00000155846	Silent	c.2892G>A
1632	PPBP	chr4	ENSG00000163736	3'UTR	c.*84C>T
1633	PPBP	chr4	ENSG00000163736	3'UTR	c.*76C>T
1634	PPFIA2	chr12	ENSG00000139220	Nonsense	c.208C>T
1635	PPFIA4	chr1	ENSG00000143847	Intron	c.1267-10C>A
1636	PPFIBP1	chr12	ENSG00000110841	Silent	c.348C>T
1637	PPL	chr16	ENSG00000118898	Nonsense	c.3907C>T
1638	PPM1L	chr3	ENSG00000163590	5'UTR	c.-453C>T
1639	PPP1R12B	chr1	ENSG00000077157	Missense	c.505G>C
1640	PPP1R12C	chr19	ENSG00000125503	5'UTR	c.-23A>G
1641	PPP1R13B	chr14	ENSG00000088808	3'UTR	c.*554C>G

Number	Gene name	Chromosome	Gene_ID	Mutation	Nucleotide substitution
1642	PPP1R13B	chr14	ENSG00000088808	3'UTR	c.*451C>A
1643	PPP1R13B	chr14	ENSG00000088808	5'Flank	
1644	PPP1R17	chr7	ENSG00000106341	3'UTR	c.*637C>G
1645	PPP1R1A	chr12	ENSG00000135447	Intron	c.511-86G>C
1646	PPP1R2	chr3	ENSG00000184203	3'UTR	c.*2072C>G
1647	PPP1R35	chr7	ENSG00000160813	Silent	c.63C>T
1648	PPP1R9B	chr17	ENSG00000108819	Missense	c.646G>A
1649	PPP1R9B	chr17	ENSG00000108819	Silent	c.288G>A
1650	PPP2R2B	chr5	ENSG00000156475	3'UTR	c.*263G>C
1651	PPP2R2C	chr4	ENSG00000074211	3'UTR	c.*549G>A
1652	PPP2R2C	chr4	ENSG00000074211	Intron	c.71-165G>A
1653	PPP2R2D	chr10	ENSG00000175470	3'UTR	c.*749G>C
1654	PPP2R5B	chr11	ENSG00000068971	3'UTR	c.*193C>A
1655	PPP6R3	chr11	ENSG00000110075	Splice region	c.1126-3C>G
1656	PRAMEF1	chr1	ENSG00000116721	Missense	c.753_754delinsGC
1657	PRAMEF20	chr1	ENSG00000204478	3'UTR	c.*68C>T
1658	PRC1	chr15	ENSG00000198901	5'UTR	c.-329G>A
1659	PRDM1	chr6	ENSG00000057657	5'UTR	c.-149G>A
1660	PRDM14	chr8	ENSG00000147596	Intron	c.755-14C>T
1661	PRDM15	chr21	ENSG00000141956	3'UTR	c.*940C>T
1662	PRDM15	chr21	ENSG00000141956	Silent	c.1593G>A
1663	PRDM15	chr21	ENSG00000141956	Missense	c.1555G>A
1664	PRELID1	chr5	ENSG00000169230	3'UTR	c.*83C>T
1665	PREX2	chr8	ENSG00000046889	Intron	c.2715+3910C>G
1666	PRKAA2	chr1	ENSG00000162409	3'UTR	c.*6245C>T
1667	PRKACB	chr1	ENSG00000142875	3'UTR	c.*969G>C
1668	PRKCE	chr2	ENSG00000171132	Missense	c.2014G>C
1669	PRMT3	chr11	ENSG00000185238	Intron	c.560+43G>C
1670	PROM1	chr4	ENSG00000007062	5'UTR	c.-189C>T
1671	PROM2	chr2	ENSG00000155066	Splice site	c.2335-1G>C
1672	PROX1	chr1	ENSG00000117707	Missense	c.793G>A
1673	PROZ	chr13	ENSG00000126231	Missense	c.180G>T
1674	PRPF39	chr14	ENSG00000185246	Splice region	c.1758-3C>G
1675	PRR36	chr19	ENSG00000183248	Silent	c.1524C>G
1676	PRRT3	chr3	ENSG00000163704	Silent	c.1912C>A
1677	PRSS56	chr2	ENSG00000237412	Silent	c.1383G>C
1678	PRTFDC1	chr10	ENSG00000099256	3'UTR	c.*297A>T
1679	PRTG	chr15	ENSG00000166450	3'UTR	c.*5214C>A
1680	PSAPL1	chr4	ENSG00000178597	3'UTR	c.*1198C>G
1681	PSD4	chr2	ENSG00000125637	Missense	c.2875G>C
1682	PSKH1	chr16	ENSG00000159792	Missense	c.1228G>C
1683	PSKH2	chr8	ENSG00000147613	Missense	c.823C>G
1684	PSMB4	chr1	ENSG00000159377	3'UTR	c.*70G>A
1685	PSMB7	chr9	ENSG00000136930	Missense	c.590G>A
1686	PSMD5	chr9	ENSG00000095261	3'UTR	c.*45G>C
1687	PSMD5	chr9	ENSG00000095261	3'UTR	c.*1809G>C
1688	PSMD6	chr3	ENSG00000163636	Missense	c.253G>A
1689	PSMD7	chr16	ENSG00000103035	3'Flank	
1690	PSMD8	chr19	ENSG00000099341	Missense	c.112C>G
1691	PSMG4	chr6	ENSG00000180822	Frame_Shift_Del	c.199_212del
1692	PSTPIP1	chr15	ENSG00000140368	Intron	c.418-63C>G
1693	PTBP3	chr9	ENSG00000119314	Missense	c.568G>C
1694	PTCD3	chr2	ENSG00000132300	3'UTR	c.*4411A>C

Number	Gene name	Chromosome	Gene_ID	Mutation	Nucleotide substitution
1695	PTCH2	chr1	ENSG00000117425	3'UTR	c.*723C>T
1696	PTCH2	chr1	ENSG00000117425	Silent	c.549C>T
1697	PTCHD1	chrX	ENSG00000165186	3'UTR	c.*2487G>A
1698	PTCHD1-AS	chrX	ENSG00000233067	Intron	n.260-13556C>T
1699	PTDSS1	chr8	ENSG00000156471	3'UTR	c.*1476G>A
1700	PTGES2	chr9	ENSG00000148334	Missense	c.154C>G
1701	PTGS2	chr1	ENSG00000073756	Missense	c.1655C>G
1702	PTK2B	chr8	ENSG00000120899	Intron	c.-38+21730T>A
1703	PTMA	chr2	ENSG00000187514	5'UTR	c.-198C>T
1704	PTMA	chr2	ENSG00000187514	Missense	c.61G>A
1705	PTMA	chr2	ENSG00000187514	Intron	c.289-64G>A
1706	PTMA	chr2	ENSG00000187514	3'UTR	c.*675G>C
1707	PTPN1	chr20	ENSG00000196396	Missense	c.588C>G
1708	PTPN13	chr4	ENSG00000163629	Missense	c.1078G>A
1709	PTPN18	chr2	ENSG00000072135	Missense	c.1366G>C
1710	PTPN4	chr2	ENSG00000088179	Missense	c.1843G>C
1711	PTPRA	chr20	ENSG00000132670	3'UTR	c.*435C>T
1712	PTPRB	chr12	ENSG00000127329	Missense	c.4802C>T
1713	PTPRE	chr10	ENSG00000132334	Missense	c.1771C>T
1714	PTPRH	chr19	ENSG00000080031	Silent	c.2568C>T
1715	PTPRN	chr2	ENSG00000054356	Intron	c.1604-11C>T
1716	PTPRN2	chr7	ENSG00000155093	3'UTR	c.*1650G>A
1717	PTPRR	chr12	ENSG00000153233	Missense	c.1225G>C
1718	PTPRS	chr19	ENSG00000105426	Intron	c.1811-7185C>G
1719	PTPRU	chr1	ENSG00000060656	Missense	c.3982C>T
1720	PUM2	chr2	ENSG00000055917	Intron	c.2217+22T>C
1721	PXDN	chr2	ENSG00000130508	Silent	c.3975C>T
1722	PXDNL	chr8	ENSG00000147485	3'UTR	c.*47G>C
1723	PXMP2	chr12	ENSG00000176894	5'UTR	c.-74C>G
1724	PXN	chr12	ENSG00000089159	3'UTR	c.*188C>G
1725	PXYLP1	chr3	ENSG00000155893	5'UTR	c.-821G>C
1726	PYGL	chr14	ENSG00000100504	Intron	c.1092+53G>A
1727	PYGL	chr14	ENSG00000100504	Intron	c.773-17C>T
1728	PYGO1	chr15	ENSG00000171016	Missense	c.1102C>G
1729	QSOX1	chr1	ENSG00000116260	3'UTR	c.*4806C>G
1730	RAB14	chr9	ENSG00000119396	Missense	c.283A>G
1731	RAB21	chr12	ENSG00000080371	5'UTR	c.-143C>T
1732	RAB35	chr12	ENSG00000111737	3'UTR	c.*2059C>T
1733	RAB3B	chr1	ENSG00000169213	3'UTR	c.*6475C>G
1734	RAB3B	chr1	ENSG00000169213	3'UTR	c.*3019C>T
1735	RAB3C	chr5	ENSG00000152932	3'UTR	c.*6473G>T
1736	RAB3GAP1	chr2	ENSG00000115839	3'UTR	c.*881C>T
1737	RAB40AL	chrX	ENSG00000102128	5'Flank	
1738	RAB8B	chr15	ENSG00000166128	5'Flank	
1739	RABGAP1	chr9	ENSG00000011454	3'UTR	c.*1544C>T
1740	RABGAP1L	chr1	ENSG00000152061	Missense	c.1699C>G
1741	RABGAP1L	chr1	ENSG00000152061	Missense	c.820G>C
1742	RABGAP1L	chr1	ENSG00000152061	Missense	c.958G>A
1743	RABGEF1	chr7	ENSG00000154710	5'UTR	c.-898C>G
1744	RABIF	chr1	ENSG00000183155	Silent	c.246C>T
1745	RAD1	chr5	ENSG00000113456	3'UTR	c.*1366G>A
1746	RAD50	chr5	ENSG00000113522	Missense	c.2007C>G
1747	RAG1	chr11	ENSG00000166349	Missense	c.2134G>A

Number	Gene name	Chromosome	Gene_ID	Mutation	Nucleotide substitution
1748	RAN	chr12	ENSG00000132341	Intron	c.121+31T>G
1749	RANBP2	chr2	ENSG00000153201	Missense	c.5456C>T
1750	RANBP3	chr19	ENSG00000031823	Intron	c.78+23C>T
1751	RAP1GAP	chr1	ENSG00000076864	Intron	c.395+81C>A
1752	RAP2B	chr3	ENSG00000181467	3'UTR	c.*4478C>T
1753	RAPGEF1	chr9	ENSG00000107263	Intron	c.2580+48G>A
1754	RAPGEF2	chr4	ENSG00000109756	Missense	c.920C>G
1755	RAPGEF2	chr4	ENSG00000109756	Nonsense	c.4404G>A
1756	RASAL2	chr1	ENSG00000075391	Silent	c.2709G>C
1757	RASGRP4	chr19	ENSG00000171777	Intron	c.1244+21G>A
1758	RAX2	chr19	ENSG00000173976	3'UTR	c.*333G>A
1759	RB1	chr13	ENSG00000139687	Nonsense	c.769C>T
1760	BAK-RBAKD	chr7	ENSG00000272968	Intron	c.*32+21G>C
1761	BAK-RBAKD	chr7	ENSG00000272968	Intron	c.*32+69G>A
1762	RBCK1	chr20	ENSG00000125826	Intron	c.41+78G>C
1763	RBFOX3	chr17	ENSG00000167281	Missense	c.505G>A
1764	RBL2	chr16	ENSG00000103479	5'UTR	c.-31C>A
1765	RBM28	chr7	ENSG00000106344	5'UTR	c.-104C>T
1766	RBM46	chr4	ENSG00000151962	Intron	c.1403-7420C>G
1767	RBMX	chrX	ENSG00000147274	Missense	c.688G>C
1768	RBP3	chr10	ENSG00000265203	Missense	c.2476T>G
1769	RBP5	chr12	ENSG00000139194	Intron	c.253-68G>C
1770	RBPJ	chr4	ENSG00000168214	Splice region	c.672C>T
1771	RBPJL	chr20	ENSG00000124232	Silent	c.159G>A
1772	RBPMS2	chr15	ENSG00000166831	Intron	c.88-10836G>C
1773	RCC1L	chr7	ENSG00000274523	Silent	c.753C>G
1774	RCHY1	chr4	ENSG00000163743	Intron	c.657+69G>C
1775	RCOR2	chr11	ENSG00000167771	Intron	c.676-45C>G
1776	RCOR3	chr1	ENSG00000117625	3'UTR	c.*144G>A
1777	RECQL5	chr17	ENSG00000108469	Splice region	c.1585+3C>T
1778	REM1	chr20	ENSG00000088320	Silent	c.525C>T
1779	RERG	chr12	ENSG00000134533	5'UTR	c.-185G>C
1780	REV3L	chr6	ENSG00000009413	Missense	c.770G>T
1781	REXO1	chr19	ENSG00000079313	Splice region	c.3318-7C>T
1782	RFC2	chr7	ENSG00000049541	Missense	c.8C>A
1783	RFX8	chr2	ENSG00000196460	Intron	c.184-88G>A
1784	RFXANK	chr19	ENSG00000064490	Frame_Shift_Del	c.406_409del
1785	RGL1	chr1	ENSG00000143344	Intron	c.1336-40C>G
1786	RGL3	chr19	ENSG00000205517	Missense	c.2113C>G
1787	RGP1	chr9	ENSG00000107185	3'UTR	c.*4118G>A
1788	RGS12	chr4	ENSG00000159788	Intron	c.2283+33G>T
1789	RGS7	chr1	ENSG00000182901	Intron	c.686+11C>A
1790	RGS7BP	chr5	ENSG00000186479	5'UTR	c.-201G>A
1791	RHCE	chr1	ENSG00000188672	3'UTR	c.*32C>G
1792	RHOB	chr2	ENSG00000143878	3'UTR	c.*750T>G
1793	RHOB	chr2	ENSG00000143878	3'UTR	c.*780C>G
1794	RHOBTB3	chr5	ENSG00000164292	Missense	c.659G>C
1795	RHOD	chr11	ENSG00000173156	Intron	c.132+67G>C
1796	RIC3	chr11	ENSG00000166405	3'UTR	c.*925C>T
1797	RIF1	chr2	ENSG00000080345	Missense	c.4339G>A
1798	RILPL1	chr12	ENSG00000188026	Silent	c.633G>A
1799	RIMS1	chr6	ENSG00000079841	Missense	c.958G>C
1800	RIMS1	chr6	ENSG00000079841	Silent	c.1530G>A

Number	Gene name	Chromosome	Gene_ID	Mutation	Nucleotide substitution
1801	RINT1	chr7	ENSG00000135249	Missense	c.958C>T
1802	RIPOR2	chr6	ENSG00000111913	Missense	c.2009C>T
1803	RMC1	chr18	ENSG00000141452	Missense	c.348C>G
1804	RMDN1	chr8	ENSG00000176623	3'UTR	c.*219C>G
1805	RMDN1	chr8	ENSG00000176623	Missense	c.677G>C
1806	RNASEH2C	chr11	ENSG00000172922	3'UTR	c.*1414G>A
1807	RND1	chr12	ENSG00000172602	3'UTR	c.*780C>A
1808	RNF103	chr2	ENSG00000239305	Missense	c.1450G>A
1809	RNF14	chr5	ENSG00000013561	3'UTR	c.*2291C>G
1810	RNF152	chr18	ENSG00000176641	3'UTR	c.*2897C>G
1811	RNF157	chr17	ENSG00000141576	3'UTR	c.*554G>A
1812	RNF157	chr17	ENSG00000141576	Intron	c.673-31C>A
1813	RNF168	chr3	ENSG00000163961	3'UTR	c.*219G>A
1814	RNF182	chr6	ENSG00000180537	Nonsense	c.19G>T
1815	RNF217	chr6	ENSG00000146373	5'UTR	c.-67G>C
1816	RNF217	chr6	ENSG00000146373	Intron	c.6+107G>A
1817	RNF32	chr7	ENSG00000105982	Intron	c.450+88G>A
1818	RNF38	chr9	ENSG00000137075	3'UTR	c.*1368G>C
1819	RNF40	chr16	ENSG00000103549	Silent	c.474C>T
1820	RNF41	chr12	ENSG00000181852	3'UTR	c.*1003C>G
1821	RNF7	chr3	ENSG00000114125	3'UTR	c.*98C>T
1822	ROCK2	chr2	ENSG00000134318	Splice site	c.2550-1G>A
1823	ROCK2	chr2	ENSG00000134318	Nonsense	c.1327C>T
1824	ROCK2	chr2	ENSG00000134318	5'UTR	c.-332C>T
1825	ROPN1	chr3	ENSG00000065371	Missense	c.167C>G
1826	RP1	chr8	ENSG00000104237	Missense	c.2136G>A
1827	RPAP2	chr1	ENSG00000122484	Missense	c.1834G>C
1828	RPGR	chrX	ENSG00000156313	Intron	c.1060-30G>C
1829	RPL10	chrX	ENSG00000147403	3'UTR	c.*730T>C
1830	RPL19	chr17	ENSG00000108298	Silent	c.342G>A
1831	RPL22	chr1	ENSG00000116251	5'UTR	c.-228C>T
1832	RPL24	chr3	ENSG00000114391	5'Flank	
1833	RPL9	chr4	ENSG00000163682	Missense	c.31G>C
1834	RPN2	chr20	ENSG00000118705	Intron	c.480-13C>G
1835	RPS19BP1	chr22	ENSG00000187051	Silent	c.84G>T
1836	RPS19BP1	chr22	ENSG00000187051	Missense	c.49G>C
1837	RPS23	chr5	ENSG00000186468	3'UTR	c.*1944C>T
1838	RPS6KB1	chr17	ENSG00000108443	3'UTR	c.*2579C>G
1839	RPS6KC1	chr1	ENSG00000136643	Missense	c.2020G>C
1840	RRBP1	chr20	ENSG00000125844	Missense	c.219G>C
1841	RRM2B	chr8	ENSG00000048392	3'UTR	c.*1630C>G
1842	RSPH1	chr21	ENSG00000160188	Nonsense	c.829G>T
1843	RSPH10B2	chr7	ENSG00000169402	5'Flank	
1844	RSPH9	chr6	ENSG00000172426	Splice region	c.626-3C>A
1845	RSRC1	chr3	ENSG00000174891	Intron	c.583+76G>A
1846	RSRC2	chr12	ENSG00000111011	Intron	c.725+12_725+15del
1847	RTCB	chr22	ENSG00000100220	3'UTR	c.*252G>A
1848	RTKN2	chr10	ENSG00000182010	3'UTR	c.*236T>C
1849	RTN2	chr19	ENSG00000125744	Missense	c.1447C>G
1850	RTN4	chr2	ENSG00000115310	Silent	c.330G>C
1851	RUFY1	chr5	ENSG00000176783	Missense	c.1126G>A
1852	RUFY1	chr5	ENSG00000176783	Splice region	c.1128+3G>A
1853	RUFY2	chr10	ENSG00000204130	Missense	c.520C>G

Number	Gene name	Chromosome	Gene_ID	Mutation	Nucleotide substitution
1854	RUNX1T1	chr8	ENSG00000079102	Silent	c.1737G>A
1855	RUSC1	chr1	ENSG00000160753	Missense	c.1309G>A
1856	RWDD2A	chr6	ENSG00000013392	5'UTR	c.-75G>C
1857	RYR1	chr19	ENSG00000196218	Intron	c.2360+34C>T
1858	RYR2	chr1	ENSG00000198626	Intron	c.416-21C>A
1859	RYR3	chr15	ENSG00000198838	Missense	c.11092G>C
1860	S100A1	chr1	ENSG00000160678	5'UTR	c.-87G>C
1861	S100A7A	chr1	ENSG00000184330	3'UTR	c.*1517T>C
1862	S1PR3	chr9	ENSG00000213694	5'UTR	c.-3651C>T
1863	SAFB2	chr19	ENSG00000130254	Intron	c.1920-52C>G
1864	SAG	chr2	ENSG00000130561	Silent	c.621G>T
1865	SAT1	chrX	ENSG00000130066	Missense	c.232G>C
1866	SATB2	chr2	ENSG00000119042	3'UTR	c.*2230C>T
1867	SBF2	chr11	ENSG00000133812	Missense	c.2959G>C
1868	SBK1	chr16	ENSG00000188322	3'UTR	c.*827G>C
1869	SBK2	chr19	ENSG00000187550	Missense	c.49G>C
1870	SBNO1	chr12	ENSG00000139697	Intron	c.3218-16G>C
1871	SBNO1	chr12	ENSG00000139697	Missense	c.2853G>C
1872	SBSPON	chr8	ENSG00000164764	Missense	c.115C>T
1873	SCAMP2	chr15	ENSG00000140497	Intron	c.472+13C>G
1874	SCAND1	chr20	ENSG00000171222	3'Flank	
1875	SCAPER	chr15	ENSG00000140386	5'UTR	c.-2G>C
1876	SCGB1A1	chr11	ENSG00000149021	Missense	c.82C>G
1877	SCLY	chr2	ENSG00000132330	5'UTR	c.-90C>T
1878	SCML1	chrX	ENSG00000047634	Silent	c.525C>A
1879	SCN3B	chr11	ENSG00000166257	3'UTR	c.*1899dup
1880	SCNN1B	chr16	ENSG00000168447	Splice region	c.128-6C>T
1881	SCNN1G	chr16	ENSG00000166828	Missense	c.1042G>A
1882	SCP2D1	chr20	ENSG00000132631	3'UTR	c.*30C>G
1883	SCUBE2	chr11	ENSG00000175356	Intron	c.968-64C>A
1884	SCYL2	chr12	ENSG00000136021	3'UTR	c.*191G>A
1885	SDK1	chr7	ENSG00000146555	Silent	c.4980G>A
1886	SDK2	chr17	ENSG00000069188	Splice site	c.1000+1G>C
1887	SEC14L2	chr22	ENSG00000100003	Missense	c.128G>A
1888	SEC14L2	chr22	ENSG00000100003	Missense	c.339C>G
1889	SEC16A	chr9	ENSG00000148396	Intron	c.846+49C>A
1890	SEC16B	chr1	ENSG00000120341	Splice site	c.998+1G>C
1891	SEC23B	chr20	ENSG00000101310	Missense	c.135G>C
1892	SEC24A	chr5	ENSG00000113615	Missense	c.668C>G
1893	SEC63	chr6	ENSG00000025796	3'UTR	c.*3659G>C
1894	SEM1	chr7	ENSG00000127922	3'UTR	c.*299C>G
1895	SEMA4D	chr9	ENSG00000187764	Missense	c.1897C>T
1896	SEMA6A	chr5	ENSG00000092421	5'UTR	c.-501G>A
1897	SENP2	chr3	ENSG00000163904	3'UTR	c.*1573C>A
1898	SENP2	chr3	ENSG00000163904	3'UTR	c.*3113C>G
1899	SEPTIN10	chr2	ENSG00000186522	5'UTR	c.-18G>C
1900	SERF2	chr15	ENSG00000140264	5'UTR	c.-60C>G
1901	SERHL2	chr22	ENSG00000183569	Missense	c.695C>T
1902	SERINC2	chr1	ENSG00000168528	Intron	c.39+72C>G
1903	SERPINB13	chr18	ENSG00000197641	3'UTR	c.*1806C>G
1904	SERPINB6	chr6	ENSG00000124570	Intron	c.-10-226C>T
1905	SERPINB7	chr18	ENSG00000166396	Intron	c.455-18C>T
1906	SERPINB9	chr6	ENSG00000170542	Intron	c.168+430C>G

Number	Gene name	Chromosome	Gene_ID	Mutation	Nucleotide substitution
1907	SESN3	chr11	ENSG00000149212	Missense	c.602G>C
1908	SETD1A	chr16	ENSG00000099381	Missense	c.2950G>A
1909	SETD1A	chr16	ENSG00000099381	Missense	c.2959G>A
1910	SETD6	chr16	ENSG00000103037	Missense	c.173C>G
1911	SETX	chr9	ENSG00000107290	3'UTR	c.*693C>A
1912	SF3A2	chr19	ENSG00000104897	Intron	c.547-21C>T
1913	SF3B3	chr16	ENSG00000189091	Missense	c.3573G>C
1914	SFXN5	chr2	ENSG00000144040	Intron	c.742-65G>C
1915	SGCE	chr7	ENSG00000127990	Nonstop	c.1448G>C
1916	SH2D4A	chr8	ENSG00000104611	Missense	c.103C>G
1917	SH3BGR	chr21	ENSG00000185437	Nonstop	c.164G>C
1918	SH3BP4	chr2	ENSG00000130147	Missense	c.2099A>C
1919	SH3BP5	chr3	ENSG00000131370	Splice region	c.890-4T>G
1920	SH3D19	chr4	ENSG00000109686	Nonstop	c.2373G>C
1921	SH3D19	chr4	ENSG00000109686	Missense	c.667G>A
1922	SHANK2	chr11	ENSG00000162105	3'UTR	c.*2450G>A
1923	SHANK2	chr11	ENSG00000162105	Missense	c.2095G>T
1924	SHANK2	chr11	ENSG00000162105	Missense	c.319G>A
1925	SHC4	chr15	ENSG00000185634	3'UTR	c.*1556dup
1926	SHE	chr1	ENSG00000169291	3'UTR	c.*2381G>C
1927	SHOC1	chr9	ENSG00000165181	Missense	c.3314C>T
1928	SHOX	chrX	ENSG00000185960	3'UTR	c.*527G>A
1929	SHOX2	chr3	ENSG00000168779	Missense	c.781C>T
1930	SHPK	chr17	ENSG00000197417	Missense	c.240G>T
1931	SHPRH	chr6	ENSG00000146414	3'UTR	c.*1687G>A
1932	SHROOM2	chrX	ENSG00000146950	Intron	c.2891+29G>A
1933	SIGLEC10	chr19	ENSG00000142512	5'Flank	
1934	SIN3A	chr15	ENSG00000169375	Missense	c.259C>T
1935	SIPA1	chr11	ENSG00000213445	Intron	c.2526-24C>T
1936	SIPA1L3	chr19	ENSG00000105738	Missense	c.3431C>T
1937	SIPA1L3	chr19	ENSG00000105738	Missense	c.3806C>A
1938	SIX4	chr14	ENSG00000100625	3'UTR	c.*502C>T
1939	SIX6	chr14	ENSG00000184302	3'UTR	c.*81G>C
1940	SKOR1	chr15	ENSG00000188779	Missense	c.694C>A
1941	SKOR1	chr15	ENSG00000188779	Nonsense	c.2047G>T
1942	SLAMF6	chr1	ENSG00000162739	3'UTR	c.*280G>C
1943	SLC10A5	chr8	ENSG00000253598	3'Flank	
1944	SLC11A2	chr12	ENSG00000110911	Missense	c.1660G>A
1945	SLC12A2	chr5	ENSG00000064651	3'UTR	c.*2520C>A
1946	SLC12A3	chr16	ENSG00000070915	Silent	c.2757G>C
1947	SLC13A3	chr20	ENSG00000158296	3'UTR	c.*1968C>G
1948	SLC13A3	chr20	ENSG00000158296	3'UTR	c.*649G>A
1949	SLC13A4	chr7	ENSG00000164707	Intron	c.711+75G>C
1950	SLC15A2	chr3	ENSG00000163406	Intron	c.604+44G>A
1951	SLC1A4	chr2	ENSG00000115902	3'UTR	c.*2518C>G
1952	SLC1A6	chr19	ENSG00000105143	Silent	c.1266C>A
1953	SLC22A2	chr6	ENSG00000112499	Silent	c.1188C>T
1954	SLC22A2	chr6	ENSG00000112499	Intron	c.455+48G>C
1955	SLC23A2	chr20	ENSG00000089057	3'UTR	c.*955G>A
1956	SLC24A1	chr15	ENSG00000074621	3'UTR	c.*969C>G
1957	SLC25A52	chr18	ENSG00000141437	3'UTR	c.*8C>A
1958	SLC26A10	chr12	ENSG00000135502	Missense	c.768G>C
1959	SLC26A6	chr3	ENSG00000225697	Splice region	c.1208+3G>A

Number	Gene name	Chromosome	Gene_ID	Mutation	Nucleotide substitution
1960	SLC26A9	chr1	ENSG00000174502	3'UTR	c.*915C>G
1961	SLC27A4	chr9	ENSG00000167114	3'UTR	c.*620C>G
1962	SLC27A6	chr5	ENSG00000113396	Missense	c.1692G>T
1963	SLC27A6	chr5	ENSG00000113396	Missense	c.1694A>G
1964	SLC28A1	chr15	ENSG00000156222	Silent	c.334C>T
1965	SLC29A4	chr7	ENSG00000164638	3'UTR	c.*162G>T
1966	SLC2A2	chr3	ENSG00000163581	3'UTR	c.*1500A>C
1967	SLC2A6	chr9	ENSG00000160326	Intron	c.1036+358G>C
1968	SLC30A1	chr1	ENSG00000170385	3'UTR	c.*591G>A
1969	SLC30A3	chr2	ENSG00000115194	Intron	c.95+25C>G
1970	SLC30A8	chr8	ENSG00000164756	Missense	c.595G>A
1971	SLC34A3	chr9	ENSG00000198569	Intron	c.847-33G>A
1972	SLC35A5	chr3	ENSG00000138459	3'UTR	c.*527G>C
1973	SLC35D1	chr1	ENSG00000116704	3'UTR	c.*3399A>G
1974	SLC35D2	chr9	ENSG00000130958	Intron	c.752+113C>T
1975	SLC35G6	chr17	ENSG00000259224	Missense	c.38C>G
1976	SLC35G6	chr17	ENSG00000259224	3'UTR	c.*119C>T
1977	SLC35G6	chr17	ENSG00000259224	3'UTR	c.*303C>T
1978	SLC37A1	chr21	ENSG00000160190	Intron	c.-301+238C>G
1979	SLC37A1	chr21	ENSG00000160190	Intron	c.564-19C>T
1980	SLC37A1	chr21	ENSG00000160190	Intron	c.731-21C>G
1981	SLC37A1	chr21	ENSG00000160190	Intron	c.1135+101C>G
1982	SLC37A1	chr21	ENSG00000160190	Intron	c.1587-22C>G
1983	SLC37A3	chr7	ENSG00000157800	Intron	c.1174+61G>A
1984	SLC38A4	chr12	ENSG00000139209	3'UTR	c.*1064G>A
1985	SLC39A14	chr8	ENSG00000104635	Intron	c.1148-17C>T
1986	SLC43A3	chr11	ENSG00000134802	Silent	c.423C>G
1987	SLC44A3	chr1	ENSG00000143036	Intron	c.27+25C>T
1988	SLC45A3	chr1	ENSG00000158715	Missense	c.275T>C
1989	SLC46A1	chr17	ENSG00000076351	Missense	c.507C>A
1990	SLC4A8	chr12	ENSG00000050438	Nonsense	c.46C>T
1991	SLC5A11	chr16	ENSG00000158865	Intron	c.135+24G>T
1992	SLC5A5	chr19	ENSG00000105641	Intron	c.423+12C>T
1993	SLC66A3	chr2	ENSG00000162976	Splice region	c.226+4A>C
1994	SLC6A15	chr12	ENSG00000072041	Nonsense	c.1246G>T
1995	SLC7A11	chr4	ENSG00000151012	5'Flank	
1996	SLC7A5	chr16	ENSG00000103257	3'UTR	c.*956C>T
1997	SLC7A7	chr14	ENSG00000155465	Intron	c.-180-1830C>G
1998	SLC8A2	chr19	ENSG00000118160	Missense	c.817G>C
1999	SLC9A2	chr2	ENSG00000115616	3'UTR	c.*2526C>G
2000	SLCO1B1	chr12	ENSG00000134538	Missense	c.1643C>T
2001	SLCO2A1	chr3	ENSG00000174640	Missense	c.1671C>G
2002	SLCO5A1	chr8	ENSG00000137571	Missense	c.2054C>T
2003	SLF1	chr5	ENSG00000133302	Frame_Shift_Del	c.2929del
2004	SLFN11	chr17	ENSG00000172716	Missense	c.1747G>C
2005	SLFN14	chr17	ENSG00000236320	Missense	c.2230G>C
2006	SLFNL1	chr1	ENSG00000171790	5'UTR	c.-470C>G
2007	SLITRK2	chrX	ENSG00000185985	3'UTR	c.*3591C>T
2008	SMAD3	chr15	ENSG00000166949	3'UTR	c.*261A>C
2009	SMAD4	chr18	ENSG00000141646	Nonsense	c.670C>T
2010	SMARCAL1	chr2	ENSG00000138375	Missense	c.2002C>T
2011	SMARCAL1	chr2	ENSG00000138375	Missense	c.2714C>G
2012	SMARCD3	chr7	ENSG00000082014	5'UTR	c.-25C>T

Number	Gene name	Chromosome	Gene_ID	Mutation	Nucleotide substitution
2013	SMC2	chr9	ENSG00000136824	Missense	c.1505G>C
2014	SMC5	chr9	ENSG00000198887	3'UTR	c.*2246G>A
2015	SMCHD1	chr18	ENSG00000101596	Intron	c.1956+39G>A
2016	SMCO1	chr3	ENSG00000214097	Missense	c.397G>C
2017	SMG1	chr16	ENSG00000157106	Missense	c.8357C>T
2018	SMG7	chr1	ENSG00000116698	Missense	c.3321G>C
2019	SMIM33	chr5	ENSG00000283288	Silent	c.273C>T
2020	SNAP25	chr20	ENSG00000132639	3'UTR	c.*307C>A
2021	SNCG	chr10	ENSG00000173267	5'UTR	c.-38C>G
2022	SNRNP200	chr2	ENSG00000144028	Missense	c.2276C>T
2023	SNW1	chr14	ENSG00000100603	Silent	c.828G>A
2024	SNX20	chr16	ENSG00000167208	3'UTR	c.*2093C>T
2025	SNX21	chr20	ENSG00000124104	Missense	c.151G>A
2026	SNX24	chr5	ENSG00000064652	Intron	c.144+90G>C
2027	SNX32	chr11	ENSG00000172803	Missense	c.1135G>C
2028	SNX4	chr3	ENSG00000114520	3'UTR	c.*1046G>C
2029	SNX4	chr3	ENSG00000114520	5'Flank	
2030	SOAT1	chr1	ENSG00000057252	Intron	c.330-29T>A
2031	SOAT1	chr1	ENSG00000057252	Splice region	c.330-3C>G
2032	SORBS2	chr4	ENSG00000154556	3'UTR	c.*1188G>C
2033	SORD	chr15	ENSG00000140263	Missense	c.517G>C
2034	SOS1	chr2	ENSG00000115904	Missense	c.2671G>C
2035	SOX1	chr13	ENSG00000182968	3'UTR	c.*1864C>T
2036	SP100	chr2	ENSG00000067066	3'UTR	c.*293T>G
2037	SP3	chr2	ENSG00000172845	3'UTR	c.*2562C>G
2038	SP6	chr17	ENSG00000189120	3'UTR	c.*23C>T
2039	SPACA1	chr6	ENSG00000118434	5'Flank	
2040	SPATA2	chr20	ENSG00000158480	3'UTR	c.*1317C>G
2041	SPATA2L	chr16	ENSG00000158792	Missense	c.958G>C
2042	SPATA2L	chr16	ENSG00000158792	Missense	c.700G>A
2043	SPATA31C1	chr9	ENSG00000230246	RNA	n.768C>T
2044	SPATA31C1	chr9	ENSG00000230246	3'Flank	
2045	SPATA5	chr4	ENSG00000145375	Missense	c.1415G>C
2046	SPATA6	chr1	ENSG00000132122	3'UTR	c.*2573C>G
2047	SPATS1	chr6	ENSG00000249481	Splice region	c.288-4C>G
2048	SPCS3	chr4	ENSG00000129128	3'UTR	c.*1251G>C
2049	SPDYC	chr11	ENSG00000204710	3'UTR	c.*14G>A
2050	SPECC1L	chr22	ENSG00000100014	Missense	c.1300G>A
2051	SPEG	chr2	ENSG00000072195	Intron	c.7859-61G>A
2052	SPG11	chr15	ENSG00000104133	Missense	c.7294C>G
2053	SPG7	chr16	ENSG00000197912	Silent	c.1152G>C
2054	SPG7	chr16	ENSG00000197912	3'UTR	c.*140G>C
2055	SPICE1	chr3	ENSG00000163611	Splice region	c.385+6C>G
2056	SPIDR	chr8	ENSG00000164808	Intron	c.1098-3765C>A
2057	SPINK14	chr5	ENSG00000196800	Intron	c.111+111C>G
2058	SPINK5	chr5	ENSG00000133710	Splice region	c.282+3G>T
2059	SPO11	chr20	ENSG00000054796	Intron	c.846-10T>G
2060	SPRY4	chr5	ENSG00000187678	3'UTR	c.*3371G>C
2061	SPRYD3	chr12	ENSG00000167778	Missense	c.483C>G
2062	SPSB4	chr3	ENSG00000175093	Splice region	c.694+4A>C
2063	SPTBN1	chr2	ENSG00000115306	Missense	c.1909G>C
2064	SPTBN2	chr11	ENSG00000173898	Intron	c.2816+31G>A
2065	SPTBN4	chr19	ENSG00000160460	Intron	c.5916-63C>A

Number	Gene name	Chromosome	Gene_ID	Mutation	Nucleotide substitution
2066	SPTSSA	chr14	ENSG00000165389	Missense	c.95G>C
2067	SRA1	chr5	ENSG00000213523	Missense	c.545C>A
2068	SRCAP	chr16	ENSG00000080603	Intron	c.3707-42G>C
2069	SRCAP	chr16	ENSG00000080603	Silent	c.9594G>A
2070	SRGAP3	chr3	ENSG00000196220	3'UTR	c.*450G>C
2071	SRPK1	chr6	ENSG00000096063	3'UTR	c.*1267G>C
2072	SRPK1	chr6	ENSG00000096063	3'UTR	c.*1230G>A
2073	SRPK1	chr6	ENSG00000096063	3'UTR	c.*1037G>C
2074	SRPK1	chr6	ENSG00000096063	3'UTR	c.*643G>C
2075	SRPK1	chr6	ENSG00000096063	3'UTR	c.*409G>T
2076	SRPK1	chr6	ENSG00000096063	Missense	c.1963G>C
2077	SRRD	chr22	ENSG00000100104	3'UTR	c.*2453G>A
2078	SRSF2	chr17	ENSG00000161547	3'UTR	c.*410G>C
2079	SRSF2	chr17	ENSG00000161547	anslation_Start_S	c.3G>C
2080	SRSF3	chr6	ENSG00000112081	3'UTR	c.*1044G>A
2081	SSBP2	chr5	ENSG00000145687	Intron	c.958-52G>C
2082	SSH2	chr17	ENSG00000141298	3'UTR	c.*724G>C
2083	SSPO	chr7	ENSG00000197558	Missense	c.2170G>C
2084	ST3GAL5	chr2	ENSG00000115525	Intron	c.319-4991G>A
2085	ST3GAL5	chr2	ENSG00000115525	Intron	c.319-5018G>A
2086	STAC2	chr17	ENSG00000141750	3'UTR	c.*299G>C
2087	STAC2	chr17	ENSG00000141750	Intron	c.941+55G>C
2088	STAC2	chr17	ENSG00000141750	Silent	c.519C>T
2089	STARD9	chr15	ENSG00000159433	Missense	c.8000C>G
2090	STAT5A	chr17	ENSG00000126561	Missense	c.1984G>A
2091	STIMATE	chr3	ENSG00000213533	Intron	c.619-46G>C
2092	STK33	chr11	ENSG00000130413	Splice region	c.559-3C>G
2093	STK38L	chr12	ENSG00000211455	3'UTR	c.*997C>T
2094	STK4	chr20	ENSG00000101109	3'UTR	c.*4624G>A
2095	STOX1	chr10	ENSG00000165730	Missense	c.1726G>A
2096	STRADA	chr17	ENSG00000266173	Intron	c.580-72C>G
2097	STX12	chr1	ENSG00000117758	Silent	c.36G>A
2098	STX16	chr20	ENSG00000124222	Intron	c.398-9C>T
2099	STXBP5	chr6	ENSG00000164506	Missense	c.530C>T
2100	STXBP6	chr14	ENSG00000168952	Nonstop	c.632G>C
2101	SUB1	chr5	ENSG00000113387	3'UTR	c.*2915C>T
2102	SUCO	chr1	ENSG00000094975	Missense	c.2364G>C
2103	SUCO	chr1	ENSG00000094975	Nonsense	c.2383G>T
2104	SULT6B1	chr2	ENSG00000138068	Missense	c.736G>C
2105	SULT6B1	chr2	ENSG00000138068	Intron	c.288+12T>C
2106	SUPT20HL1	chrX	ENSG00000223731	RNA	n.45C>T
2107	SUSD1	chr9	ENSG00000106868	5'UTR	c.-35G>T
2108	SUSD4	chr1	ENSG00000143502	Intron	c.1445-9C>G
2109	SV2B	chr15	ENSG00000185518	3'UTR	c.*8461C>T
2110	SVEP1	chr9	ENSG00000165124	3'UTR	c.*1131G>A
2111	SVOPL	chr7	ENSG00000157703	Intron	c.83-32G>T
2112	SYDE2	chr1	ENSG00000097096	Nonsense	c.2542C>T
2113	SYN1	chrX	ENSG00000008056	Silent	c.156G>A
2114	SYN2	chr3	ENSG00000157152	Intron	c.981-13G>A
2115	SYNE3	chr14	ENSG00000176438	5'UTR	c.-323G>C
2116	SYNJ1	chr21	ENSG00000159082	Intron	c.1511-28C>G
2117	SYNJ2	chr6	ENSG00000078269	3'UTR	c.*2621G>C
2118	SYNPO2	chr4	ENSG00000172403	Intron	c.12+37819C>G

Number	Gene name	Chromosome	Gene_ID	Mutation	Nucleotide substitution
2119	SYT1	chr12	ENSG00000067715	Missense	c.966G>C
2120	SYT14	chr1	ENSG00000143469	3'UTR	c.*2400G>C
2121	SYTL4	chrX	ENSG00000102362	Missense	c.1702C>G
2122	SYVN1	chr11	ENSG00000162298	Missense	c.377T>C
2123	SZT2	chr1	ENSG00000198198	Missense	c.2731G>A
2124	TACR3	chr4	ENSG00000169836	3'UTR	c.*31A>G
2125	TAGLN	chr11	ENSG00000149591	5'UTR	c.-820G>C
2126	TAGLN3	chr3	ENSG00000144834	Silent	c.72G>A
2127	TAL1	chr1	ENSG00000162367	3'UTR	c.*892G>A
2128	TALDO1	chr11	ENSG00000177156	Intron	c.637+17G>A
2129	TAOK1	chr17	ENSG00000160551	Intron	c.2362-19C>T
2130	TARBP1	chr1	ENSG00000059588	Nonsense	c.2374C>T
2131	TARS2	chr1	ENSG00000143374	Intron	c.1149+72C>G
2132	TAX1BP1	chr7	ENSG00000106052	Missense	c.2170C>G
2133	TBC1D1	chr4	ENSG00000065882	Intron	c.1414-41G>C
2134	TBC1D12	chr10	ENSG00000108239	3'UTR	c.*1185C>T
2135	TBC1D12	chr10	ENSG00000108239	3'UTR	c.*2673C>T
2136	TBC1D13	chr9	ENSG00000107021	Intron	c.98-38C>G
2137	TBC1D17	chr19	ENSG00000104946	Missense	c.226G>A
2138	TBC1D19	chr4	ENSG00000109680	Missense	c.937G>C
2139	TBC1D9B	chr5	ENSG00000197226	Intron	c.2863+42C>G
2140	TBCD	chr17	ENSG00000141556	Intron	c.1318+13701C>T
2141	TBK1	chr12	ENSG00000183735	Nonsense	c.1043C>G
2142	TBL1XR1	chr3	ENSG00000177565	3'UTR	c.*3909G>C
2143	TBL2	chr7	ENSG00000106638	Intron	c.130+1794_130+1797del
2144	TBRG4	chr7	ENSG00000136270	Intron	c.908-99G>A
2145	TBX2	chr17	ENSG00000121068	3'Flank	
2146	TBX6	chr16	ENSG00000149922	5'UTR	c.-581C>A
2147	TBXA2R	chr19	ENSG00000006638	Missense	c.784C>G
2148	TBXA2R	chr19	ENSG00000006638	Missense	c.620C>T
2149	TCAP	chr17	ENSG00000173991	5'UTR	c.-687G>C
2150	TCAP	chr17	ENSG00000173991	3'UTR	c.*377G>A
2151	TCN2	chr22	ENSG00000185339	Intron	c.941-13C>A
2152	TCP10L2	chr6	ENSG00000166984	3'Flank	
2153	TCP11L1	chr11	ENSG00000176148	Intron	c.1328-14C>G
2154	TCTN2	chr12	ENSG00000168778	Intron	c.1312+49C>T
2155	TCTN3	chr10	ENSG00000119977	Intron	c.310+30T>C
2156	TDRD7	chr9	ENSG00000196116	Silent	c.426G>A
2157	TDRD7	chr9	ENSG00000196116	Missense	c.3265G>A
2158	TDRD7	chr9	ENSG00000196116	3'UTR	c.*173G>C
2159	TDRD7	chr9	ENSG00000196116	3'Flank	
2160	TDRD9	chr14	ENSG00000156414	Nonsense	c.1201G>T
2161	TEDC1	chr14	ENSG00000185347	Missense	c.69C>G
2162	TENM1	chrX	ENSG00000009694	3'UTR	c.*812C>G
2163	TENM2	chr5	ENSG00000145934	Missense	c.211G>A
2164	TEP1	chr14	ENSG00000129566	Missense	c.5472C>G
2165	TERB1	chr16	ENSG00000249961	Missense	c.172G>A
2166	TEX14	chr17	ENSG00000121101	Intron	c.4103-55G>C
2167	TEX15	chr8	ENSG00000133863	3'UTR	c.*754C>G
2168	TEX15	chr8	ENSG00000133863	Missense	c.7483G>A
2169	TEX15	chr8	ENSG00000133863	Missense	c.7267G>C
2170	TEX36	chr10	ENSG00000175018	Intron	c.51+33C>G
2171	TEX55	chr3	ENSG00000163424	Missense	c.310G>A

Number	Gene name	Chromosome	Gene_ID	Mutation	Nucleotide substitution
2172	TF	chr3	ENSG00000091513	Silent	c.1656G>A
2173	TFAP2A	chr6	ENSG00000137203	Intron	c.34-2558G>C
2174	TFE3	chrX	ENSG00000068323	Intron	c.885+18G>A
2175	TFEC	chr7	ENSG00000105967	3'UTR	c.*475C>T
2176	TFIP11	chr22	ENSG00000100109	Missense	c.2072C>T
2177	TFIP11	chr22	ENSG00000100109	Missense	c.1974C>G
2178	TFPI2	chr7	ENSG00000105825	3'UTR	c.*1084G>A
2179	TG	chr8	ENSG00000042832	Silent	c.4965C>T
2180	TG	chr8	ENSG00000042832	5'UTR	c.-169G>C
2181	TGFBR3	chr1	ENSG00000069702	Missense	c.2223G>A
2182	TGM5	chr15	ENSG00000104055	Silent	c.1965G>A
2183	THAP2	chr12	ENSG00000173451	3'UTR	c.*287A>C
2184	THBS1	chr15	ENSG00000137801	3'UTR	c.*253G>C
2185	THOC3	chr5	ENSG00000051596	Intron	c.154-63A>G
2186	THOP1	chr19	ENSG00000172009	Missense	c.1918G>C
2187	THSD7A	chr7	ENSG00000005108	Nonsense	c.1825G>T
2188	THSD7A	chr7	ENSG00000005108	Missense	c.1450G>C
2189	TIGD1	chr2	ENSG00000221944	Silent	c.1623C>T
2190	TIGD3	chr11	ENSG00000173825	Silent	c.621G>A
2191	TIGIT	chr3	ENSG00000181847	5'UTR	c.-211G>A
2192	TIMM17A	chr1	ENSG00000134375	5'UTR	c.-4C>T
2193	TIMP3	chr22	ENSG00000100234	3'UTR	c.*296C>G
2194	TK1	chr17	ENSG00000167900	3'UTR	c.*334C>G
2195	TLCD5	chr11	ENSG00000181264	3'UTR	c.*2782C>G
2196	TLE1	chr9	ENSG00000196781	Intron	c.66+31G>A
2197	TLE3	chr15	ENSG00000140332	Silent	c.1770C>T
2198	TLE4	chr9	ENSG00000106829	3'UTR	c.*28C>T
2199	TLE6	chr19	ENSG00000104953	Intron	c.1538-19G>A
2200	TLN2	chr15	ENSG00000171914	Missense	c.3436G>A
2201	TLR3	chr4	ENSG00000164342	Nonsense	c.1447C>T
2202	TLR8	chrX	ENSG00000101916	Nonsense	c.1367C>G
2203	TLX1	chr10	ENSG00000107807	5'Flank	
2204	TLX1	chr10	ENSG00000107807	5'Flank	
2205	TM2D2	chr8	ENSG00000169490	3'UTR	c.*1024G>C
2206	TM2D2	chr8	ENSG00000169490	3'UTR	c.*235C>G
2207	TM4SF18	chr3	ENSG00000163762	3'UTR	c.*1955C>G
2208	TM9SF4	chr20	ENSG00000101337	Intron	c.1118+46C>T
2209	TMC8	chr17	ENSG00000167895	Missense	c.55G>A
2210	MED7-TICAM	chr5	ENSG00000251201	3'UTR	c.*506G>A
2211	TMED8	chr14	ENSG00000100580	3'UTR	c.*5795G>C
2212	TMED8	chr14	ENSG00000100580	3'UTR	c.*2085G>C
2213	TMEFF2	chr2	ENSG00000144339	Intron	c.745+87G>A
2214	TMEM106C	chr12	ENSG00000134291	Intron	c.-28-65G>T
2215	TMEM131L	chr4	ENSG00000121210	Intron	c.1597+25G>A
2216	TMEM131L	chr4	ENSG00000121210	Splice region	c.2121-3C>T
2217	TMEM140	chr7	ENSG00000146859	5'Flank	
2218	TMEM151B	chr6	ENSG00000178233	Intron	c.136-446C>G
2219	TMEM185B	chr2	ENSG00000226479	Missense	c.318G>C
2220	TMEM185B	chr2	ENSG00000226479	5'UTR	c.-42C>T
2221	IEM189-UBE2	chr20	ENSG00000124208	Missense	c.267C>G
2222	TMEM192	chr4	ENSG00000170088	Intron	c.27+73G>A
2223	TMEM200A	chr6	ENSG00000164484	Splice site	c.-16-1G>A
2224	TMEM266	chr15	ENSG00000169758	Missense	c.112G>T

Number	Gene name	Chromosome	Gene_ID	Mutation	Nucleotide substitution
2225	TMEM33	chr4	ENSG00000109133	Silent	c.300C>G
2226	TMEM39A	chr3	ENSG00000176142	Missense	c.1054C>T
2227	TMEM44	chr3	ENSG00000145014	5'UTR	c.-184C>T
2228	TMEM81	chr1	ENSG00000174529	Silent	c.246G>C
2229	TMEM87A	chr15	ENSG00000103978	Intron	c.1626+65C>A
2230	TMOD2	chr15	ENSG00000128872	3'UTR	c.*7480C>G
2231	TMPRSS15	chr21	ENSG00000154646	Missense	c.597A>C
2232	TMPRSS3	chr21	ENSG00000160183	3'UTR	c.*871C>A
2233	TMPRSS6	chr22	ENSG00000187045	Missense	c.1283C>T
2234	TNC	chr9	ENSG00000041982	Missense	c.5539C>T
2235	TNFRSF25	chr1	ENSG00000215788	Missense	c.126G>C
2236	TNFRSF6B	chr20	ENSG00000243509	Intron	c.620-27G>C
2237	TNPO2	chr19	ENSG00000105576	5'UTR	c.-371C>A
2238	TNPO3	chr7	ENSG00000064419	Missense	c.547C>G
2239	TNRC18	chr7	ENSG00000182095	Silent	c.82C>T
2240	TNRC18P2	chr7	ENSG00000223566	3'Flank	
2241	TNS1	chr2	ENSG00000079308	Splice region	c.343-3C>A
2242	TNS3	chr7	ENSG00000136205	3'UTR	c.*2275T>G
2243	TNS3	chr7	ENSG00000136205	Intron	c.3929-19C>T
2244	TOE1	chr1	ENSG00000132773	3'UTR	c.*50C>G
2245	TOPBP1	chr3	ENSG00000163781	3'UTR	c.*55G>A
2246	TP53	chr17	ENSG00000141510	Missense	c.695T>C
2247	TP53TG5	chr20	ENSG00000124251	Missense	c.788G>A
2248	TPD52	chr8	ENSG00000076554	3'UTR	c.*1803C>G
2249	TPM3	chr1	ENSG00000143549	Intron	c.776-1371C>A
2250	TPM3	chr1	ENSG00000143549	Intron	c.776-1385C>G
2251	TPR	chr1	ENSG00000047410	Missense	c.4562T>G
2252	TPR	chr1	ENSG00000047410	Nonsense	c.4558C>T
2253	TRAF3	chr14	ENSG00000131323	3'UTR	c.*3099C>T
2254	TRAF3IP2	chr6	ENSG00000056972	Missense	c.739C>A
2255	TRAF5	chr1	ENSG00000082512	Missense	c.1504G>C
2256	TRAK2	chr2	ENSG00000115993	3'UTR	c.*2576C>T
2257	TRAK2	chr2	ENSG00000115993	3'UTR	c.*2054G>A
2258	TRAV14DV4	chr14	ENSG00000211792	Intron	c.50-56C>G
2259	TRAV22	chr14	ENSG00000211802	Nonsense	c.305C>G
2260	TRIM11	chr1	ENSG00000154370	5'UTR	c.-181G>C
2261	TRIM24	chr7	ENSG00000122779	5'UTR	c.-182G>A
2262	TRIM41	chr5	ENSG00000146063	Silent	c.1710G>C
2263	TRIM46	chr1	ENSG00000163462	Intron	c.1285+149_1285+150del
2264	TRIM6	chr11	ENSG00000121236	3'UTR	c.*72C>G
2265	TRIM6	chr11	ENSG00000121236	3'UTR	c.*476G>C
2266	TRIM66	chr11	ENSG00000166436	Intron	c.9+45C>A
2267	TRIM67	chr1	ENSG00000119283	3'UTR	c.*3431C>A
2268	TRMT13	chr1	ENSG00000122435	Intron	c.232-71C>G
2269	TRMU	chr22	ENSG00000100416	Silent	c.21C>T
2270	TRPC3	chr4	ENSG00000138741	Nonsense	c.421G>T
2271	TRPM3	chr9	ENSG00000083067	Intron	c.3350+34G>T
2272	TRPM6	chr9	ENSG00000119121	3'UTR	c.*878C>G
2273	TRPM7	chr15	ENSG00000092439	Nonsense	c.335C>G
2274	TRPS1	chr8	ENSG00000104447	Silent	c.3819G>A
2275	TRPS1	chr8	ENSG00000104447	Silent	c.1260C>T
2276	TRRAP	chr7	ENSG00000196367	Missense	c.8359G>A
2277	TSC1	chr9	ENSG00000165699	Silent	c.1260G>A

Number	Gene name	Chromosome	Gene_ID	Mutation	Nucleotide substitution
2278	TSC2	chr16	ENSG00000103197	Missense	c.4428G>C
2279	TSC22D4	chr7	ENSG00000166925	Missense	c.338G>C
2280	TSG101	chr11	ENSG00000074319	5'Flank	
2281	TSGA10	chr2	ENSG00000135951	Intron	c.-485+291G>A
2282	TSHZ2	chr20	ENSG00000182463	Missense	c.861G>C
2283	TSHZ3	chr19	ENSG00000121297	3'UTR	c.*1499C>G
2284	TSNARE1	chr8	ENSG00000171045	Intron	c.1449+2984C>T
2285	TSNARE1	chr8	ENSG00000171045	Missense	c.358C>G
2286	TSPAN32	chr11	ENSG00000064201	Intron	c.627+46C>T
2287	TSPAN33	chr7	ENSG00000158457	5'UTR	c.-200C>G
2288	TSPAN33	chr7	ENSG00000158457	3'UTR	c.*289G>C
2289	TSPEAR	chr21	ENSG00000175894	Intron	c.1566+2962C>T
2290	TSPOAP1	chr17	ENSG00000005379	Silent	c.3168C>A
2291	TSPOAP1	chr17	ENSG00000005379	Missense	c.2800G>T
2292	TTC12	chr11	ENSG00000149292	Nonsense	c.52G>T
2293	TTC13	chr1	ENSG00000143643	Splice region	c.983+3G>C
2294	TTC21B	chr2	ENSG00000123607	Missense	c.1546C>A
2295	TTC23L	chr5	ENSG00000205838	Silent	c.375G>A
2296	TTC41P	chr12	ENSG00000214198	RNA	n.748C>G
2297	TTC7B	chr14	ENSG00000165914	Intron	c.1967-8511G>C
2298	TTC9	chr14	ENSG00000133985	Missense	c.565G>C
2299	TTLL13P	chr15	ENSG00000213471	Missense	c.2100C>G
2300	TTLL5	chr14	ENSG00000119685	Intron	c.3823+1991_3823+1992del
2301	TTN	chr2	ENSG00000155657	Splice region	c.39845-3C>G
2302	TTN	chr2	ENSG00000155657	Missense	c.34591C>G
2303	TTN	chr2	ENSG00000155657	Intron	c.13859-10489C>G
2304	TTN	chr2	ENSG00000155657	Missense	c.23734C>G
2305	TTN	chr2	ENSG00000155657	Silent	c.13419C>T
2306	TTN	chr2	ENSG00000155657	Missense	c.16684G>C
2307	TTN	chr2	ENSG00000155657	Missense	c.10696G>C
2308	TTN	chr2	ENSG00000155657	Missense	c.4756G>A
2309	TUBA1B	chr12	ENSG00000123416	Intron	c.4-23G>C
2310	TUBA4A	chr2	ENSG00000127824	Splice region	c.3+5G>C
2311	TUT1	chr11	ENSG00000149016	3'Flank	
2312	TXK	chr4	ENSG00000074966	Silent	c.1425C>T
2313	TXLNG	chrX	ENSG00000086712	3'UTR	c.*1293G>A
2314	TXNRD2	chr22	ENSG00000184470	Intron	c.104-2262C>T
2315	TXNRD2	chr22	ENSG00000184470	Missense	c.92G>A
2316	UBAP2	chr9	ENSG00000137073	Nonsense	c.1655C>G
2317	UBAP2L	chr1	ENSG00000143569	Missense	c.1795C>G
2318	UBASH3A	chr21	ENSG00000160185	Silent	c.99C>T
2319	UBASH3A	chr21	ENSG00000160185	Missense	c.1193G>A
2320	UBE2J1	chr6	ENSG00000198833	3'UTR	c.*2027G>T
2321	UBE2K	chr4	ENSG00000078140	Intron	c.63+33C>T
2322	UBE2S	chr19	ENSG00000108106	Intron	c.3+99G>C
2323	UBE2V2	chr8	ENSG00000169139	3'UTR	c.*2356C>T
2324	UBE3A	chr15	ENSG00000114062	Intron	c.-112+81C>G
2325	UBE3C	chr7	ENSG00000009335	Intron	c.1810-35C>G
2326	UBR1	chr15	ENSG00000159459	Splice region	c.2379+7C>G
2327	UBR3	chr2	ENSG00000144357	Missense	c.1546G>T
2328	UBR4	chr1	ENSG00000127481	Missense	c.1912C>G
2329	UGT2A2	chr4	ENSG00000271271	Missense	c.33G>T
2330	ULK3	chr15	ENSG00000140474	Intron	c.469+22G>A

Number	Gene name	Chromosome	Gene_ID	Mutation	Nucleotide substitution
2331	UNC50	chr2	ENSG00000115446	5'UTR	c.-428G>C
2332	UNC5C	chr4	ENSG00000182168	3'UTR	c.*1281C>T
2333	Unknown	chr19		IGR	
2334	Unknown	chr16		IGR	
2335	Unknown	chr16		IGR	
2336	Unknown	chr19		IGR	
2337	UPK1B	chr3	ENSG00000114638	Intron	c.733-10dup
2338	UQCRFS1	chr19	ENSG00000169021	Missense	c.464A>C
2339	UROC1	chr3	ENSG00000159650	Missense	c.1125C>G
2340	USE1	chr19	ENSG00000053501	3'UTR	c.*153G>C
2341	USH2A	chr1	ENSG00000042781	Missense	c.2509C>T
2342	USH2A	chr1	ENSG00000042781	5'UTR	c.-26C>G
2343	USHBP1	chr19	ENSG00000130307	Missense	c.1889C>T
2344	USP19	chr3	ENSG00000172046	Missense	c.1387A>C
2345	USP28	chr11	ENSG00000048028	Missense	c.1249G>A
2346	USP3	chr15	ENSG00000140455	Intron	c.950-9C>G
2347	USP31	chr16	ENSG00000103404	Missense	c.1130C>A
2348	USP34	chr2	ENSG00000115464	3'Flank	
2349	USP34	chr2	ENSG00000115464	Missense	c.6384G>C
2350	USP35	chr11	ENSG00000118369	Missense	c.163G>C
2351	USP37	chr2	ENSG00000135913	Missense	c.333G>A
2352	USP39	chr2	ENSG00000168883	Nonsense	c.721C>T
2353	USP40	chr2	ENSG00000085982	Intron	c.2647+81G>C
2354	USP46	chr4	ENSG00000109189	Intron	c.36+2856C>T
2355	USP47	chr11	ENSG00000170242	Missense	c.2641G>A
2356	USP48	chr1	ENSG00000090686	5'UTR	c.-258G>T
2357	USP49	chr6	ENSG00000164663	Silent	c.1176C>T
2358	USP51	chrX	ENSG00000247746	Missense	c.38C>G
2359	UTP18	chr17	ENSG00000011260	Silent	c.687T>G
2360	UTP20	chr12	ENSG00000120800	Missense	c.5844G>C
2361	UTP23	chr8	ENSG00000147679	3'UTR	c.*1315G>C
2362	UTP3	chr4	ENSG00000132467	Nonsense	c.301G>T
2363	UTP3	chr4	ENSG00000132467	3'UTR	c.*176G>C
2364	UXS1	chr2	ENSG00000115652	Intron	c.95-56G>C
2365	UXT	chrX	ENSG00000126756	Intron	c.249-43G>A
2366	VCL	chr10	ENSG00000035403	Missense	c.755C>G
2367	VEGFC	chr4	ENSG00000150630	Missense	c.979C>A
2368	VILL	chr3	ENSG00000136059	Intron	c.2205+68G>C
2369	VLDLR	chr9	ENSG00000147852	5'UTR	c.-293C>G
2370	VLDLR	chr9	ENSG00000147852	Missense	c.2468G>C
2371	VMP1	chr17	ENSG00000062716	Intron	c.76+10G>C
2372	VPS13A	chr9	ENSG00000197969	Silent	c.2928G>A
2373	VPS13A	chr9	ENSG00000197969	Missense	c.4834C>G
2374	VPS13B	chr8	ENSG00000132549	Missense	c.3041C>G
2375	VPS13C	chr15	ENSG00000129003	Missense	c.10585G>C
2376	VPS13C	chr15	ENSG00000129003	Missense	c.4663G>C
2377	VPS18	chr15	ENSG00000104142	Silent	c.900G>A
2378	VPS37D	chr7	ENSG00000176428	Missense	c.268C>T
2379	VPS45	chr1	ENSG00000136631	Missense	c.992G>T
2380	VPS50	chr7	ENSG00000004766	3'UTR	c.*404C>T
2381	VPS50	chr7	ENSG00000004766	Silent	c.2088C>G
2382	VPS51	chr11	ENSG00000149823	Intron	c.61+84G>C
2383	VPS72	chr1	ENSG00000163159	Nonsense	c.152C>G

Number	Gene name	Chromosome	Gene_ID	Mutation	Nucleotide substitution
2384	VRTN	chr14	ENSG00000133980	Missense	c.1798G>C
2385	VSIG10L	chr19	ENSG00000186806	Missense	c.1295C>G
2386	VSTM2A	chr7	ENSG00000170419	3'UTR	c.*1007G>C
2387	VSTM2A	chr7	ENSG00000170419	3'UTR	c.*1409G>A
2388	VSTM2B	chr19	ENSG00000187135	Intron	c.82+77G>C
2389	VSTM4	chr10	ENSG00000165633	3'UTR	c.*6G>C
2390	VSX2	chr14	ENSG00000119614	Missense	c.850G>C
2391	VWC2	chr7	ENSG00000188730	Silent	c.522G>A
2392	VWCE	chr11	ENSG00000167992	Splice region	c.2231-6C>G
2393	VWF	chr12	ENSG00000110799	3'UTR	c.*133G>T
2394	WBP1	chr2	ENSG00000239779	Intron	c.349+80_349+81insAA
2395	WDCP	chr2	ENSG00000163026	3'UTR	c.*265A>C
2396	WDFY1	chr2	ENSG00000085449	3'UTR	c.*1932G>C
2397	WDFY1	chr2	ENSG00000085449	3'UTR	c.*1164G>A
2398	WDR13	chrX	ENSG00000101940	Intron	c.832-35G>A
2399	WDR19	chr4	ENSG00000157796	Silent	c.2505C>G
2400	WDR20	chr14	ENSG00000140153	Intron	c.250-63C>G
2401	WDR27	chr6	ENSG00000184465	Intron	c.645-382C>T
2402	WDR4	chr21	ENSG00000160193	3'UTR	c.*571C>T
2403	WDR4	chr21	ENSG00000160193	Missense	c.1234T>C
2404	WDR87	chr19	ENSG00000171804	Silent	c.1731C>G
2405	WIF1	chr12	ENSG00000156076	Intron	c.289-5992G>A
2406	WNK2	chr9	ENSG00000165238	Missense	c.1566G>C
2407	WNK4	chr17	ENSG00000126562	Missense	c.259C>G
2408	WNT2	chr7	ENSG00000105989	3'UTR	c.*1454C>T
2409	WNT2	chr7	ENSG00000105989	3'UTR	c.*1432C>T
2410	WNT2	chr7	ENSG00000105989	3'UTR	c.*304C>A
2411	WSB1	chr17	ENSG00000109046	5'UTR	c.-229G>C
2412	WSB2	chr12	ENSG00000176871	3'UTR	c.*864G>T
2413	WWC2	chr4	ENSG00000151718	3'UTR	c.*472G>A
2414	XCR1	chr3	ENSG00000173578	Silent	c.339C>T
2415	XDH	chr2	ENSG00000158125	Intron	c.1981-29G>A
2416	XPO5	chr6	ENSG00000124571	Intron	c.3066+71C>T
2417	XPO7	chr8	ENSG00000130227	Intron	c.1105-36G>A
2418	XRCC5	chr2	ENSG00000079246	3'UTR	c.*956G>C
2419	XRCC6	chr22	ENSG00000196419	5'UTR	c.-600C>T
2420	YES1	chr18	ENSG00000176105	Missense	c.414G>C
2421	YKT6	chr7	ENSG00000106636	5'Flank	
2422	YOD1	chr1	ENSG00000180667	3'UTR	c.*2036C>G
2423	YRDC	chr1	ENSG00000196449	Intron	c.625-67C>T
2424	ZBED1	chrX	ENSG00000214717	Silent	c.780C>T
2425	ZBTB20	chr3	ENSG00000181722	Silent	c.1455G>A
2426	ZBTB34	chr9	ENSG00000177125	3'UTR	c.*4694C>T
2427	ZBTB8OS	chr1	ENSG00000176261	Nonsense	c.202G>T
2428	ZC2HC1A	chr8	ENSG00000104427	Intron	c.16+59C>A
2429	ZC2HC1B	chr6	ENSG00000118491	5'Flank	
2430	ZC3H10	chr12	ENSG00000135482	Missense	c.661C>T
2431	ZC3H12C	chr11	ENSG00000149289	Missense	c.271G>A
2432	ZC3H12C	chr11	ENSG00000149289	Missense	c.289G>C
2433	ZC3H4	chr19	ENSG00000130749	Missense	c.170G>T
2434	ZC3H6	chr2	ENSG00000188177	3'UTR	c.*3392C>A
2435	ZC3H6	chr2	ENSG00000188177	3'UTR	c.*6121T>A
2436	ZC3H7B	chr22	ENSG00000100403	5'UTR	c.-1G>C

Number	Gene name	Chromosome	Gene_ID	Mutation	Nucleotide substitution
2437	ZDHHC18	chr1	ENSG00000204160	3'UTR	c.*88G>A
2438	ZDHHC19	chr3	ENSG00000163958	5'UTR	c.-68C>G
2439	ZDHHC5	chr11	ENSG00000156599	Nonsense	c.1193C>G
2440	ZFHX3	chr16	ENSG00000140836	Missense	c.6200C>T
2441	ZFHX4	chr8	ENSG00000091656	Missense	c.988G>C
2442	ZFHX4	chr8	ENSG00000091656	Missense	c.7748G>A
2443	ZFHX4	chr8	ENSG00000091656	Silent	c.9582A>G
2444	ZFP41	chr8	ENSG00000181638	Missense	c.20G>A
2445	ZFPL1	chr11	ENSG00000162300	Missense	c.406C>G
2446	ZFPM1	chr16	ENSG00000179588	Missense	c.2874G>C
2447	ZFX	chrX	ENSG00000005889	3'UTR	c.*254C>G
2448	ZIC4	chr3	ENSG00000174963	Missense	c.661C>G
2449	ZIC4	chr3	ENSG00000174963	Missense	c.379G>C
2450	ZIM2	chr19	ENSG00000269699	Missense	c.1471G>C
2451	ZKSCAN5	chr7	ENSG00000196652	Missense	c.1584G>C
2452	ZMAT1	chrX	ENSG00000166432	Intron	c.121+5816G>A
2453	ZMAT3	chr3	ENSG00000172667	3'UTR	c.*3496C>T
2454	ZMYND12	chr1	ENSG00000066185	Missense	c.811G>C
2455	ZMYND19	chr9	ENSG00000165724	5'UTR	c.-16G>C
2456	ZNF10	chr12	ENSG00000256223	5'UTR	c.-530C>G
2457	ZNF136	chr19	ENSG00000196646	Silent	c.792G>C
2458	ZNF148	chr3	ENSG00000163848	3'UTR	c.*2622G>T
2459	ZNF2	chr2	ENSG00000275111	3'UTR	c.*676G>C
2460	ZNF252P	chr8	ENSG00000196922	RNA	n.1351G>C
2461	ZNF260	chr19	ENSG00000254004	3'UTR	c.*297A>C
2462	ZNF274	chr19	ENSG00000171606	Missense	c.979G>C
2463	ZNF322	chr6	ENSG00000181315	Missense	c.100G>A
2464	ZNF326	chr1	ENSG00000162664	Missense	c.464C>T
2465	ZNF354C	chr5	ENSG00000177932	3'UTR	c.*3045G>A
2466	ZNF362	chr1	ENSG00000160094	3'UTR	c.*772G>T
2467	ZNF366	chr5	ENSG00000178175	Missense	c.255G>T
2468	ZNF37A	chr10	ENSG00000075407	3'UTR	c.*3694A>T
2469	ZNF385B	chr2	ENSG00000144331	Missense	c.634G>A
2470	ZNF391	chr6	ENSG00000124613	Intron	c.-188+45C>A
2471	ZNF407	chr18	ENSG00000215421	Nonsense	c.3394G>T
2472	ZNF41	chrX	ENSG00000147124	Missense	c.271G>A
2473	ZNF410	chr14	ENSG00000119725	Intron	c.913+140C>T
2474	ZNF410	chr14	ENSG00000119725	Intron	c.913+154C>T
2475	ZNF414	chr19	ENSG00000133250	5'UTR	c.-14G>C
2476	ZNF420	chr19	ENSG00000197050	Missense	c.224G>T
2477	ZNF432	chr19	ENSG00000256087	Silent	c.975C>A
2478	ZNF432	chr19	ENSG00000256087	Missense	c.772G>A
2479	ZNF442	chr19	ENSG00000198342	Missense	c.238C>A
2480	ZNF449	chrX	ENSG00000173275	Silent	c.621G>A
2481	ZNF467	chr7	ENSG00000181444	3'UTR	c.*25C>G
2482	ZNF467	chr7	ENSG00000181444	3'UTR	c.*2C>G
2483	ZNF467	chr7	ENSG00000181444	Silent	c.879C>A
2484	ZNF490	chr19	ENSG00000188033	3'UTR	c.*1730C>G
2485	ZNF497	chr19	ENSG00000174586	Intron	c.-111-109G>A
2486	ZNF502	chr3	ENSG00000196653	Missense	c.357G>C
2487	ZNF514	chr2	ENSG00000144026	3'UTR	c.*428C>T
2488	ZNF516	chr18	ENSG00000101493	3'UTR	c.*402G>C
2489	ZNF521	chr18	ENSG00000198795	Missense	c.2949G>C

Number	Gene name	Chromosome	Gene_ID	Mutation	Nucleotide substitution
2490	ZNF541	chr19	ENSG00000118156	Missense	c.142C>T
2491	ZNF550	chr19	ENSG00000251369	Missense	c.544C>T
2492	ZNF566	chr19	ENSG00000186017	3'UTR	c.*2212G>C
2493	ZNF568	chr19	ENSG00000198453	3'UTR	c.*390T>C
2494	ZNF568	chr19	ENSG00000198453	Missense	c.692G>C
2495	ZNF578	chr19	ENSG00000258405	Missense	c.601G>A
2496	ZNF586	chr19	ENSG00000083828	Intron	c.34+65C>T
2497	ZNF598	chr16	ENSG00000167962	Intron	c.2362-44G>T
2498	ZNF609	chr15	ENSG00000180357	Silent	c.249G>T
2499	ZNF609	chr15	ENSG00000180357	Missense	c.659G>C
2500	ZNF625	chr19	ENSG00000257591	Intron	c.3+140C>T
2501	ZNF644	chr1	ENSG00000122482	Missense	c.1870G>C
2502	ZNF649	chr19	ENSG00000198093	Missense	c.1132G>C
2503	ZNF655	chr7	ENSG00000197343	3'UTR	c.*884C>G
2504	ZNF664	chr12	ENSG00000179195	3'UTR	c.*495C>A
2505	ZNF664	chr12	ENSG00000179195	3'UTR	c.*1592C>G
2506	ZNF664	chr12	ENSG00000179195	3'UTR	c.*2265C>G
2507	ZNF692	chr1	ENSG00000171163	5'UTR	c.-197G>C
2508	ZNF7	chr8	ENSG00000147789	Missense	c.148C>G
2509	ZNF785	chr16	ENSG00000197162	3'UTR	c.*334G>A
2510	ZNF827	chr4	ENSG00000151612	Missense	c.698A>G
2511	ZNF841	chr19	ENSG00000197608	Nonsense	c.2547C>A
2512	ZPLD1	chr3	ENSG00000170044	3'UTR	c.*634T>A
2513	ZSCAN18	chr19	ENSG00000121413	Missense	c.776C>A
