# Supplementary material for: Stimulating T cell responses against patient-derived breast cancer cells with neoantigen peptide-loaded peripheral blood mononuclear cells
Source: Cancer Immunol Immunother. 2024 Feb 13;73(3):43. doi: 10.1007/s00262-024-03627-3 (PMC10864427; doi:10.1007/s00262-024-03627-3)
Supplement: Supplementary file 6 — (PDF 321 kb) [file 262_2024_3627_MOESM6_ESM.pdf]

Supplementary Table S3b. List of 3,490 somatic mutations of PC-B-148CA

Number	Gene name	Chromosome	Gene_ID	Mutation	Nucleotide substitution
1	A4GALT	chr22	ENSG00000128274	Missense	c.688A>G
2	AACS	chr12	ENSG00000081760	Intron	c.571-647T>C
3	AACS	chr12	ENSG00000081760	Intron	c.1310-1011del
4	AAK1	chr2	ENSG00000115977	3'UTR	c.*167del
5	AASDHPPT	chr11	ENSG00000149313	3'UTR	c.*1628dup
6	ABCA13	chr7	ENSG00000179869	Splice region	c.12460-8del
7	ABCA13	chr7	ENSG00000179869	Missense	c.12845G>A
8	ABCA2	chr9	ENSG00000107331	Missense	c.5224A>G
9	ABCA2	chr9	ENSG00000107331	Missense	c.572A>G
10	ABCA7	chr19	ENSG00000064687	Splice region	c.1445+8G>A
11	ABCA7	chr19	ENSG00000064687	Silent	c.4305A>C
12	ABCC5	chr3	ENSG00000114770	Missense	c.1484C>T
13	ABCD3	chr1	ENSG00000117528	Intron	c.1903-19del
14	ABCF2	chr7	ENSG00000033050	5'UTR	c.-148C>T
15	ABCF3	chr3	ENSG00000161204	Intron	c.1552-46T>C
16	ABCG4	chr11	ENSG00000172350	Missense	c.1250C>A
17	ABHD17A	chr19	ENSG00000129968	Missense	c.604G>A
18	ABHD17B	chr9	ENSG00000107362	3'UTR	c.*32T>C
19	ABHD2	chr15	ENSG00000140526	3'UTR	c.*5920C>T
20	ABI2	chr2	ENSG00000138443	3'UTR	c.*10573C>T
21	ABL2	chr1	ENSG00000143322	5'UTR	c.-191G>A
22	ABLIM1	chr10	ENSG00000099204	Intron	c.2142+41G>A
23	ABLIM2	chr4	ENSG00000163995	Missense	c.1195A>G
24	ABLIM3	chr5	ENSG00000173210	Missense	c.685T>C
25	AC005324.3	chr17	ENSG00000251537	3'UTR	c.*2125del
26	AC006305.1	chr18	ENSG00000206129	RNA	n.713C>T
27	AC008268.1	chr2	ENSG00000235584	RNA	n.211A>G
28	AC015802.6	chr17	ENSG00000284526	3'UTR	c.*3556del
29	AC018638.1	chr7	ENSG00000229413	5'Flank	
30	AC019209.3	chr12	ENSG00000256686	Intron	n.92-3115A>G
31	AC019294.1	chr15	ENSG00000187812	Intron	n.125+11C>T
32	AC019294.3	chr15	ENSG00000261043	RNA	n.92A>G
33	AC026464.4	chr16	ENSG00000260914	Intron	c.94-6937del
34	AC080125.1	chr2	ENSG00000225406	RNA	n.642T>G
35	AC093909.6	chr4	ENSG00000278974	3'Flank	
36	AC100868.1	chr8	ENSG00000258417	Silent	c.378C>T
37	AC105942.1	chr1	ENSG00000235501	RNA	n.1349G>A
38	AC187653.1	chr7	ENSG00000248767	Intron	c.107+13dup
39	ACAA2	chr18	ENSG00000167315	Intron	c.1110-57A>G
40	ACAD11	chr3	ENSG00000240303	Intron	c.1071-10T>C
41	ACADVL	chr17	ENSG00000072778	Intron	c.62+27G>T
42	ACAP3	chr1	ENSG00000131584	Missense	c.71C>T
43	ACCS	chr11	ENSG00000110455	Missense	c.343T>C
44	ACOT11	chr1	ENSG00000162390	Intron	c.1236+99del
45	ACOT11	chr1	ENSG00000162390	3'UTR	c.*3193del
46	ACP4	chr19	ENSG00000142513	Intron	c.986+63T>C
47	ACPP	chr3	ENSG00000014257	Frame shift Ins	c.846dup
48	ACPP	chr3	ENSG00000014257	3'UTR	c.*1227A>G
49	ACRV1	chr11	ENSG00000134940	3'UTR	c.*234dup
50	ACRV1	chr11	ENSG00000134940	Silent	c.201G>A
51	ACSBG1	chr15	ENSG00000103740	3'UTR	c.*3837A>G

Number	Gene name	Chromosome	Gene_ID	Mutation	Nucleotide substitution
52	ACSF2	chr17	ENSG00000167107	Intron	c.889-22A>G
53	ACSF2	chr17	ENSG00000167107	Splice site	c.1216-2A>G
54	ACSM3	chr16	ENSG00000005187	Intron	c.939+58A>C
55	ACTC1	chr15	ENSG00000159251	Intron	c.617-48T>C
56	ACTL10	chr20	ENSG00000182584	3'UTR	c.*21del
57	ACTR3	chr2	ENSG00000115091	3'UTR	c.*1158del
58	ACTR3C	chr7	ENSG00000106526	3'UTR	c.*2589T>C
59	ACTR3C	chr7	ENSG00000106526	3'UTR	c.*1284T>C
60	ACVR1B	chr12	ENSG00000135503	5'UTR	c.-25G>T
61	ACVR1B	chr12	ENSG00000135503	3'UTR	c.*1590del
62	ADA2	chr22	ENSG00000093072	Silent	c.1129C>T
63	ADAL	chr15	ENSG00000168803	5'UTR	c.-10del
64	ADAM12	chr10	ENSG00000148848	Intron	c.2114-11del
65	ADAM15	chr1	ENSG00000143537	Silent	c.2394A>G
66	ADAM21	chr14	ENSG00000139985	Missense	c.1528C>T
67	ADAM28	chr8	ENSG00000042980	Intron	c.648+15T>A
68	ADAM28	chr8	ENSG00000042980	3'UTR	c.*2418A>G
69	ADAM7	chr8	ENSG00000069206	Intron	c.389+41T>C
70	ADAMTS13	chr9	ENSG00000160323	Missense	c.863C>A
71	ADAMTS17	chr15	ENSG00000140470	Missense	c.724G>A
72	ADAMTS20	chr12	ENSG00000173157	3'UTR	c.*676T>A
73	ADAMTS3	chr4	ENSG00000156140	Missense	c.125A>G
74	ADAMTS4	chr1	ENSG00000158859	3'UTR	c.*597_*598insC
75	ADAMTS9	chr3	ENSG00000163638	Intron	c.5208+40del
76	ADAMTSL1	chr9	ENSG00000178031	3'UTR	c.*760del
77	ADAMTSL1	chr9	ENSG00000178031	Missense	c.1690A>G
78	ADAMTSL3	chr15	ENSG00000156218	3'UTR	c.*54T>C
79	ADAMTSL4	chr1	ENSG00000143382	Intron	c.435-50G>T
80	ADAT2	chr6	ENSG00000189007	Intron	c.97-5925del
81	ADCK2	chr7	ENSG00000133597	Intron	c.1741-5_1741-3del
82	ADCY1	chr7	ENSG00000164742	3'UTR	c.*3396del
83	ADCY10	chr1	ENSG00000143199	Missense	c.1190G>A
84	ADCY3	chr2	ENSG00000138031	In_Frame_Del	c.2564_2566del
85	ADCY3	chr2	ENSG00000138031	In_Frame_Del	c.2564_2566del
86	ADCY5	chr3	ENSG00000173175	Intron	c.85-18T>A
87	ADCY8	chr8	ENSG00000155897	5'Flank	
88	ADCY9	chr16	ENSG00000162104	Silent	c.2433G>A
89	ADCY9	chr16	ENSG00000162104	Missense	c.1475C>T
90	ADCYAP1	chr18	ENSG00000141433	3'UTR	c.*1745_*1746del
91	ADCYAP1R1	chr7	ENSG00000078549	3'UTR	c.*3456del
92	ADD1	chr4	ENSG00000087274	Intron	c.511-17A>G
93	ADGRA2	chr8	ENSG00000020181	Missense	c.1849G>A
94	ADGRB1	chr8	ENSG00000181790	Silent	c.339C>A
95	ADGRD2	chr9	ENSG00000180264	Intron	c.2522-22C>T
96	ADGRE2	chr19	ENSG00000127507	Intron	c.2353-26A>G
97	ADGRE4P	chr19	ENSG00000268758	Intron	n.74+2795T>C
98	ADGRG7	chr3	ENSG00000144820	Missense	c.1153T>A
99	ADGRL1	chr19	ENSG00000072071	Missense	c.232A>G
100	ADGRL3	chr4	ENSG00000150471	3'UTR	c.*741del
101	ADH5	chr4	ENSG00000197894	Intron	c.1100+66G>A
102	ADH5	chr4	ENSG00000197894	Intron	c.13-452C>G
103	ADNP	chr20	ENSG00000101126	3'UTR	c.*1424T>C
104	ADPRH	chr3	ENSG00000144843	3'UTR	c.*1363A>G

Number	Gene name	Chromosome	Gene_ID	Mutation	Nucleotide substitution
105	AEBP2	chr12	ENSG00000139154	Intron	c.879+15T>C
106	AFAP1L1	chr5	ENSG00000157510	3'UTR	c.*1749del
107	AFTPH	chr2	ENSG00000119844	Silent	c.2208A>G
108	AFTPH	chr2	ENSG00000119844	Missense	c.2377A>G
109	AGAP1	chr2	ENSG00000157985	Intron	c.396+36del
110	AGAP1	chr2	ENSG00000157985	Intron	c.673+23488del
111	AGAP3	chr7	ENSG00000133612	Missense	c.1259A>G
112	AGFG1	chr2	ENSG00000173744	Splice region	c.694+4A>C
113	AGFG2	chr7	ENSG00000106351	3'UTR	c.*849T>C
114	AGL	chr1	ENSG00000162688	Missense	c.4240A>G
115	AGPAT3	chr21	ENSG00000160216	Missense	c.96G>T
116	AGPAT3	chr21	ENSG00000160216	Intron	c.768-76A>G
117	AGRN	chr1	ENSG00000188157	Missense	c.1435T>C
118	AGRN	chr1	ENSG00000188157	Intron	c.3751+33G>A
119	AHCYL1	chr1	ENSG00000168710	3'UTR	c.*1553C>T
120	AHDC1	chr1	ENSG00000126705	Silent	c.3597C>G
121	AHNAK	chr11	ENSG00000124942	3'UTR	c.*259_*260dup
122	AHNAK	chr11	ENSG00000124942	Missense	c.12155_12156delinsGA
123	AICDA	chr12	ENSG00000111732	Missense	c.106C>T
124	AIDA	chr1	ENSG00000186063	Frame shift del	c.751del
125	AIDA	chr1	ENSG00000186063	Frame shift del	c.751del
126	AIF1L	chr9	ENSG00000126878	3'UTR	c.*2162dup
127	AIPL1	chr17	ENSG00000129221	Intron	c.96+22C>A
128	AK2	chr1	ENSG00000004455	Missense	c.394C>T
129	AK3	chr9	ENSG00000147853	3'UTR	c.*1403del
130	AKNA	chr9	ENSG00000106948	Intron	c.2448-23A>G
131	AKR1C1	chr10	ENSG00000187134	3'UTR	c.*1993del
132	AKT2	chr19	ENSG00000105221	3'UTR	c.*3155del
133	AL031590.1	chr22	ENSG00000284633	Intron	n.110-15676del
134	AL135905.2	chr6	ENSG00000285976	Intron	c.*679-98T>C
135	AL135905.2	chr6	ENSG00000285976	3'UTR	c.*2899dup
136	ALDH1A3	chr15	ENSG00000184254	5'UTR	c.-80T>C
137	ALDH1L1	chr3	ENSG00000144908	Nonsense	c.1114G>T
138	ALDH3A2	chr17	ENSG00000072210	Intron	c.1443+5665del
139	ALDH8A1	chr6	ENSG00000118514	3'UTR	c.*201dup
140	ALDH9A1	chr1	ENSG00000143149	3'UTR	c.*557del
141	ALG10	chr12	ENSG00000139133	Silent	c.897C>T
142	ALKBH4	chr7	ENSG00000160993	5'Flank	
143	ALKBH6	chr19	ENSG00000239382	Missense	c.247G>A
144	ALMS1	chr2	ENSG00000116127	Silent	c.12108A>G
145	ALPI	chr2	ENSG00000163295	3'UTR	c.*389G>A
146	ALPK3	chr15	ENSG00000136383	3'UTR	c.*4816A>G
147	ALPL	chr1	ENSG00000162551	Intron	c.61+64T>C
148	ALX4	chr11	ENSG00000052850	3'UTR	c.*3069C>T
149	AMDHD2	chr16	ENSG00000162066	Intron	c.1142-26dup
150	AMER2	chr13	ENSG00000165566	5'UTR	c.-111G>A
151	AMFR	chr16	ENSG00000159461	Silent	c.1054C>A
152	AMIGO3	chr3	ENSG00000176020	Missense	c.659C>A
153	AMT	chr3	ENSG00000145020	Missense	c.569T>C
154	AMZ1	chr7	ENSG00000174945	Intron	c.305-20C>T
155	ANAPC4	chr4	ENSG00000053900	Intron	c.877-14del
156	ANGPT1	chr8	ENSG00000154188	Nonsense	c.70C>T
157	ANGPTL5	chr11	ENSG00000187151	Splice region	c.661+5A>C

Number	Gene name	Chromosome	Gene_ID	Mutation	Nucleotide substitution
158	ANGPTL6	chr19	ENSG00000130812	Silent	c.618G>A
159	ANGPTL7	chr1	ENSG00000171819	Missense	c.31T>C
160	ANK1	chr8	ENSG00000029534	3'Flank	
161	ANK2	chr4	ENSG00000145362	Missense	c.2126A>G
162	ANK2	chr4	ENSG00000145362	Frame shift Ins	c.10268dup
163	ANK3	chr10	ENSG00000151150	Silent	c.4611A>G
164	ANKEF1	chr20	ENSG00000132623	Silent	c.288A>G
165	ANKFY1	chr17	ENSG00000185722	Frame shift del	c.2728del
166	ANKFY1	chr17	ENSG00000185722	Frame shift del	c.2728del
167	ANKFY1	chr17	ENSG00000185722	Missense	c.1613C>T
168	ANKH	chr5	ENSG00000154122	3'UTR	c.*3900del
169	ANKH	chr5	ENSG00000154122	3'UTR	c.*1278del
170	ANKIB1	chr7	ENSG00000001629	Missense	c.2261A>G
171	ANKIB1	chr7	ENSG00000001629	Silent	c.2355T>C
172	ANKK1	chr11	ENSG00000170209	Silent	c.117G>A
173	ANKLE2	chr12	ENSG00000176915	Intron	c.182-434_182-433del
174	ANKRD13A	chr12	ENSG00000076513	3'UTR	c.*524A>T
175	ANKRD13C	chr1	ENSG00000118454	5'UTR	c.-219A>C
176	ANKRD13D	chr11	ENSG00000172932	3'UTR	c.*11del
177	ANKRD29	chr18	ENSG00000154065	3'UTR	c.*1228A>G
178	ANKRD30B	chr18	ENSG00000180777	Intron	c.1330-48del
179	ANKRD33	chr12	ENSG00000167612	Missense	c.1310G>T
180	ANKRD36	chr2	ENSG00000135976	Missense	c.75A>T
181	ANKRD36	chr2	ENSG00000135976	Missense	c.79_80delinsTA
182	ANKRD36	chr2	ENSG00000135976	Intron	c.1560+14T>C
183	ANKRD36C	chr2	ENSG00000174501	Intron	c.*6+2306del
184	ANKRD40	chr17	ENSG00000154945	3'UTR	c.*13T>C
185	ANKRD45	chr1	ENSG00000183831	Intron	c.-16+2110del
186	ANKRD49	chr11	ENSG00000168876	Silent	c.348C>A
187	ANKRD52	chr12	ENSG00000139645	Missense	c.2637G>T
188	ANKS1B	chr12	ENSG00000185046	3'UTR	c.*1198A>G
189	ANKZF1	chr2	ENSG00000163516	Intron	c.820-61del
190	ANO4	chr12	ENSG00000151572	5'Flank	
191	ANO7	chr2	ENSG00000146205	Intron	c.2695-44T>C
192	ANO8	chr19	ENSG00000074855	Missense	c.1357C>A
193	ANPEP	chr15	ENSG00000166825	Missense	c.1027C>A
194	ANXA2	chr15	ENSG00000182718	Intron	c.202+7745C>A
195	AOAH	chr7	ENSG00000136250	Silent	c.1350C>T
196	AOC1	chr7	ENSG00000002726	Intron	c.-16-64A>G
197	AOC1	chr7	ENSG00000002726	Missense	c.939G>T
198	AOC3	chr17	ENSG00000131471	Silent	c.477T>C
199	AP001267.5	chr11	ENSG00000285827	Intron	c.-799+32301T>C
200	AP1M2	chr19	ENSG00000129354	Silent	c.1119G>C
201	AP2A2	chr11	ENSG00000183020	Intron	c.67+7580G>A
202	AP3M1	chr10	ENSG00000185009	3'UTR	c.*1188_*1190del
203	AP3S2	chr15	ENSG00000157823	Intron	c.274-6676del
204	AP4M1	chr7	ENSG00000221838	Intron	c.148-32del
205	APAF1	chr12	ENSG00000120868	Missense	c.1442T>C
206	APAF1	chr12	ENSG00000120868	Intron	c.956-7057T>C
207	APAF1	chr12	ENSG00000120868	3'UTR	c.*652del
208	APBB1IP	chr10	ENSG00000077420	3'UTR	c.*202T>C
209	APEX2	chrX	ENSG00000169188	3'UTR	c.*1149dup
210	APH1A	chr1	ENSG00000117362	5'UTR	c.-454dup

Number	Gene name	Chromosome	Gene_ID	Mutation	Nucleotide substitution
211	APLP2	chr11	ENSG00000084234	Intron	c.2191-45T>C
212	APOA2	chr1	ENSG00000158874	Missense	c.8T>C
213	AQP3	chr9	ENSG00000165272	Missense	c.559C>T
214	AQP8	chr16	ENSG00000103375	Silent	c.747T>C
215	ARCN1	chr11	ENSG00000095139	Silent	c.459T>C
216	AREG	chr4	ENSG00000109321	Silent	c.267C>T
217	AREL1	chr14	ENSG00000119682	3'UTR	c.*2114del
218	ARF1	chr1	ENSG00000143761	3'Flank	
219	ARFGEF1	chr8	ENSG00000066777	Frame shift Ins	c.4655dup
220	ARFGEF2	chr20	ENSG00000124198	Intron	c.3432+773C>T
221	ARFGEF2	chr20	ENSG00000124198	3'UTR	c.*2805_*2806del
222	ARFGEF3	chr6	ENSG00000112379	Intron	c.3369-30A>T
223	ARFGEF3	chr6	ENSG00000112379	Intron	c.4097-48del
224	ARFIP1	chr4	ENSG00000164144	3'UTR	c.*742del
225	ARGLU1	chr13	ENSG00000134884	3'UTR	c.*1692del
226	ARHGAP10	chr4	ENSG00000071205	Missense	c.173G>C
227	ARHGAP10	chr4	ENSG00000071205	Intron	c.1557-14dup
228	ARHGAP12	chr10	ENSG00000165322	Intron	c.1796-88A>G
229	ARHGAP12	chr10	ENSG00000165322	Frame shift del	c.1782del
230	ARHGAP12	chr10	ENSG00000165322	Frame shift del	c.1782del
231	ARHGAP18	chr6	ENSG00000146376	Intron	c.616+37del
232	ARHGAP20	chr11	ENSG00000137727	Missense	c.2105G>A
233	ARHGAP26	chr5	ENSG00000145819	3'UTR	c.*5422del
234	ARHGAP29	chr1	ENSG00000137962	3'UTR	c.*484T>C
235	ARHGAP31	chr3	ENSG00000031081	5'Flank	
236	ARHGAP31	chr3	ENSG00000031081	3'UTR	c.*3582G>T
237	ARHGAP33	chr19	ENSG00000004777	Frame shift Ins	c.3254dup
238	ARHGAP44	chr17	ENSG00000006740	3'UTR	c.*393dup
239	ARHGAP6	chrX	ENSG00000047648	3'UTR	c.*1218_*1219del
240	ARHGAP8	chr22	ENSG00000241484	Intron	c.299+851del
241	ARHGEF1	chr19	ENSG00000076928	Missense	c.1903C>T
242	ARHGEF11	chr1	ENSG00000132694	Missense	c.4385C>T
243	ARHGEF12	chr11	ENSG00000196914	Frame shift del	c.1562del
244	ARHGEF12	chr11	ENSG00000196914	Frame shift del	c.1562del
245	ARHGEF15	chr17	ENSG00000198844	Missense	c.716G>A
246	ARHGEF16	chr1	ENSG00000130762	Missense	c.70C>T
247	ARHGEF2	chr1	ENSG00000116584	Intron	c.2885-56A>G
248	ARHGEF2	chr1	ENSG00000116584	Frame shift del	c.1919del
249	ARHGEF2	chr1	ENSG00000116584	Frame shift del	c.1919del
250	ARHGEF28	chr5	ENSG00000214944	Intron	c.33+109del
251	ARHGEF5	chr7	ENSG00000050327	3'UTR	c.*238del
252	ARID1A	chr1	ENSG00000117713	Frame shift Ins	c.5548dup
253	ARID1A	chr1	ENSG00000117713	Silent	c.6426C>T
254	ARID1A	chr1	ENSG00000117713	Missense	c.6767C>T
255	ARID1A	chr1	ENSG00000117713	3'UTR	c.*577A>G
256	ARID1A	chr1	ENSG00000117713	3'UTR	c.*1253del
257	ARID4A	chr14	ENSG00000032219	Intron	c.118-1434A>G
258	ARIH1	chr15	ENSG00000166233	Intron	c.589-35A>G
259	ARL10	chr5	ENSG00000175414	3'UTR	c.*1538del
260	ARL15	chr5	ENSG00000185305	3'UTR	c.*488del
261	ARL4C	chr2	ENSG00000188042	3'UTR	c.*938dup
262	ARL5B	chr10	ENSG00000165997	3'UTR	c.*5427A>G
263	ARMC12	chr6	ENSG00000157343	Missense	c.19C>A

Number	Gene name	Chromosome	Gene_ID	Mutation	Nucleotide substitution
264	ARMC6	chr19	ENSG00000105676	Intron	c.778+62T>C
265	ARNT	chr1	ENSG00000143437	Intron	c.1506-44A>T
266	ARNT2	chr15	ENSG00000172379	5'Flank	
267	ARNT2	chr15	ENSG00000172379	Silent	c.915A>G
268	ARNTL2	chr12	ENSG00000029153	Missense	c.1357A>C
269	ARNTL2	chr12	ENSG00000029153	3'UTR	c.*2707T>A
270	ARPC5L	chr9	ENSG00000136950	5'UTR	c.-314T>C
271	ART4	chr12	ENSG00000111339	3'UTR	c.*2313del
272	ARX	chrX	ENSG00000004848	3'UTR	c.*683del
273	ASAP1	chr8	ENSG00000153317	Intron	c.166-16C>T
274	ASB13	chr10	ENSG00000196372	3'UTR	c.*254G>A
275	ASB15	chr7	ENSG00000146809	Missense	c.1588A>G
276	ASCC1	chr10	ENSG00000138303	Intron	c.311-1578T>G
277	ASIC3	chr7	ENSG00000213199	Silent	c.444A>G
278	ASPHD2	chr22	ENSG00000128203	3'UTR	c.*1034del
279	ASPM	chr1	ENSG00000066279	Missense	c.3739A>G
280	ASPM	chr1	ENSG00000066279	Silent	c.2649C>T
281	ASPN	chr9	ENSG00000106819	Frame shift del	c.201del
282	ASPN	chr9	ENSG00000106819	Frame shift del	c.201del
283	ASPSCR1	chr17	ENSG00000169696	Intron	c.1170+533_1170+534del
284	ASXL2	chr2	ENSG00000143970	Missense	c.1838G>A
285	ATAD3A	chr1	ENSG00000197785	Silent	c.381T>C
286	ATAD3B	chr1	ENSG00000160072	Missense	c.1312T>C
287	ATAD5	chr17	ENSG00000176208	Silent	c.2505T>C
288	ATF7IP2	chr16	ENSG00000166669	3'UTR	c.*384del
289	ATG7	chr3	ENSG00000197548	Intron	c.-10-38T>C
290	ATP10D	chr4	ENSG00000145246	3'UTR	c.*573A>G
291	ATP11A	chr13	ENSG00000068650	Intron	c.570+55C>A
292	ATP11A	chr13	ENSG00000068650	Silent	c.3192C>T
293	ATP11B	chr3	ENSG00000058063	Missense	c.779T>C
294	ATP13A2	chr1	ENSG00000159363	Intron	c.1040-44G>T
295	ATP13A3	chr3	ENSG00000133657	5'Flank	
296	ATP1B1	chr1	ENSG00000143153	Intron	c.98-739A>G
297	ATP2B2	chr3	ENSG00000157087	3'UTR	c.*1633del
298	ATP2B2	chr3	ENSG00000157087	Intron	c.3096+69C>G
299	ATP2B3	chrX	ENSG00000067842	Missense	c.2474T>C
300	ATP2B3	chrX	ENSG00000067842	3'UTR	c.*789T>G
301	ATP5F1A	chr18	ENSG00000152234	Intron	c.1176+56A>G
302	ATP5F1C	chr10	ENSG00000165629	Intron	c.891-775dup
303	ATP6V0D1	chr16	ENSG00000159720	3'UTR	c.*209del
304	ATP6V1B1	chr2	ENSG00000116039	Frame shift Ins	c.1155dup
305	ATP7A	chrX	ENSG00000165240	3'UTR	c.*1861dup
306	ATP8A2	chr13	ENSG00000132932	Intron	c.2601+8713del
307	ATP8A2	chr13	ENSG00000132932	Intron	c.2690+12A>G
308	ATP8B3	chr19	ENSG00000130270	Intron	c.2378+9T>C
309	ATP8B3	chr19	ENSG00000130270	Intron	c.1585-41G>A
310	ATPAF2	chr17	ENSG00000171953	Intron	c.179-51A>G
311	ATXN1	chr6	ENSG00000124788	3'UTR	c.*5657del
312	ATXN1	chr6	ENSG00000124788	Missense	c.1946C>T
313	ATXN3	chr14	ENSG00000066427	3'UTR	c.*1753A>G
314	ATXN3	chr14	ENSG00000066427	Silent	c.351C>T
315	ATXN7L1	chr7	ENSG00000146776	Frame shift del	c.1933del
316	ATXN7L1	chr7	ENSG00000146776	Frame shift del	c.1933del

Number	Gene name	Chromosome	Gene_ID	Mutation	Nucleotide substitution
317	AVIL	chr12	ENSG00000135407	Missense	c.1613A>G
318	AVL9	chr7	ENSG00000105778	Intron	c.529+52A>G
319	AVL9	chr7	ENSG00000105778	3'UTR	c.*4275del
320	AZGP1	chr7	ENSG00000160862	Intron	c.337+1504_337+1511del
321	B3GALNT2	chr1	ENSG00000162885	3'UTR	c.*197A>G
322	B3GAT3	chr11	ENSG00000149541	Splice region	c.910-5T>G
323	B3GLCT	chr13	ENSG00000187676	Intron	c.121-134C>T
324	B3GLCT	chr13	ENSG00000187676	3'UTR	c.*1246A>G
325	B4GALT1	chr9	ENSG00000086062	3'UTR	c.*705del
326	B4GALT2	chr1	ENSG00000117411	Intron	c.950+42_950+43delinsAG
327	B4GALT6	chr18	ENSG00000118276	Missense	c.497C>T
328	BAALC	chr8	ENSG00000164929	3'UTR	c.*1650_*1651del
329	BABAM2	chr2	ENSG00000158019	5'UTR	c.-138T>C
330	BACE1	chr11	ENSG00000186318	Intron	c.262-661del
331	BAHCC1	chr17	ENSG00000266074	Intron	c.179-3998G>C
332	BAIAP2L2	chr22	ENSG00000128298	5'UTR	c.-48A>G
333	BANF1	chr11	ENSG00000175334	Missense	c.110G>T
334	BAZ1B	chr7	ENSG00000009954	Intron	c.3729-11del
335	BBS7	chr4	ENSG00000138686	Missense	c.596A>G
336	BCAP29	chr7	ENSG00000075790	3'UTR	c.*303del
337	BCAS1	chr20	ENSG00000064787	Intron	c.1365-18C>T
338	BCKDHA	chr19	ENSG00000248098	Missense	c.233T>C
339	BCKDHA	chr19	ENSG00000248098	3'UTR	c.*385T>C
340	BCKDHB	chr6	ENSG00000083123	Intron	c.634-14del
341	BCL2L14	chr12	ENSG00000121380	Intron	c.*340-97G>A
342	BCL9	chr1	ENSG00000116128	Missense	c.4097C>T
343	BCL9L	chr11	ENSG00000186174	Silent	c.3234A>T
344	BCLAF1	chr6	ENSG00000029363	Missense	c.1798T>C
345	BCLAF3	chrX	ENSG00000173681	3'UTR	c.*2797del
346	BCO2	chr11	ENSG00000197580	Intron	c.736+48G>A
347	BCOR	chrX	ENSG00000183337	3'UTR	c.*378A>G
348	BCR	chr22	ENSG00000186716	Intron	c.1974+82del
349	BDNF	chr11	ENSG00000176697	Frame shift del	c.538del
350	BDNF	chr11	ENSG00000176697	Frame shift del	c.538del
351	BEND3	chr6	ENSG00000178409	3'UTR	c.*398del
352	BHLHE22	chr8	ENSG00000180828	3'UTR	c.*607G>A
353	BICRA	chr19	ENSG00000063169	Frame shift del	c.2138del
354	BID	chr22	ENSG00000015475	Silent	c.459C>T
355	BIN1	chr2	ENSG00000136717	3'UTR	c.*479del
356	BIRC2	chr11	ENSG00000110330	Intron	c.1074+14del
357	BLK	chr8	ENSG00000136573	Intron	c.1029+780C>T
358	BLOC1S2	chr10	ENSG00000196072	Silent	c.24A>G
359	BLOC1S6	chr15	ENSG00000104164	3'UTR	c.*1293T>C
360	BMF	chr15	ENSG00000104081	3'UTR	c.*2114del
361	BMP2K	chr4	ENSG00000138756	3'UTR	c.*3235T>C
362	BNC2	chr9	ENSG00000173068	3'UTR	c.*1681C>T
363	BNC2	chr9	ENSG00000173068	Nonstop_Mutatio	c.3098A>G
364	BNC2	chr9	ENSG00000173068	Missense	c.2966C>A
365	BPGM	chr7	ENSG00000172331	3'UTR	c.*318A>G
366	BPTF	chr17	ENSG00000171634	In_Frame_Del	c.589_603del
367	BPTF	chr17	ENSG00000171634	Frame shift del	c.2881del
368	BRAF	chr7	ENSG00000157764	Missense	c.1064A>T
369	BRAP	chr12	ENSG00000089234	Intron	c.1325+31A>G

Number	Gene name	Chromosome	Gene_ID	Mutation	Nucleotide substitution
370	BRCA1	chr17	ENSG00000012048	Intron	c.-19-20124A>G
371	BRCA2	chr13	ENSG00000139618	3'UTR	c.*383G>A
372	BRD1	chr22	ENSG00000100425	Silent	c.1602C>T
373	BRD3	chr9	ENSG00000169925	Missense	c.190G>T
374	BRF1	chr14	ENSG00000185024	Intron	c.-161-11del
375	BRIX1	chr5	ENSG00000113460	Intron	c.562-7del
376	BRK1	chr3	ENSG00000254999	Intron	c.202-58del
377	BTAF1	chr10	ENSG00000095564	Intron	c.1906-12del
378	BTBD8	chr1	ENSG00000189195	Frame shift del	c.3516del
379	BTN3A2	chr6	ENSG00000186470	Missense	c.715G>A
380	BUD23	chr7	ENSG00000071462	5'Flank	
381	C10orf105	chr10	ENSG00000214688	3'UTR	c.*369C>A
382	C11orf45	chr11	ENSG00000174370	3'UTR	c.*1621del
383	C12orf29	chr12	ENSG00000133641	Intron	c.91-11C>T
384	C14orf132	chr14	ENSG00000227051	3'UTR	c.*2370del
385	C15orf39	chr15	ENSG00000167173	3'UTR	c.*877T>C
386	C17orf58	chr17	ENSG00000186665	Frame shift del	c.24del
387	C17orf58	chr17	ENSG00000186665	Frame shift del	c.24del
388	C17orf67	chr17	ENSG00000214226	Missense	c.41T>C
389	C1D	chr2	ENSG00000197223	3'UTR	c.*1375C>T
390	C1orf112	chr1	ENSG00000000460	3'UTR	c.*1025C>T
391	C1orf21	chr1	ENSG00000116667	3'UTR	c.*8743C>A
392	C1orf21	chr1	ENSG00000116667	3'UTR	c.*8750A>G
393	C1orf226	chr1	ENSG00000239887	3'UTR	c.*3057del
394	C1orf229	chr1	ENSG00000221953	RNA	n.1834del
395	C1QTNF6	chr22	ENSG00000133466	3'UTR	c.*1706T>C
396	C1QTNF9	chr13	ENSG00000240654	3'UTR	c.*400A>G
397	C22orf23	chr22	ENSG00000128346	3'UTR	c.*478A>G
398	C2CD3	chr11	ENSG00000168014	Missense	c.4933A>G
399	C2CD4D	chr1	ENSG00000225556	Intron	c.-231-72T>C
400	C2CD6	chr2	ENSG00000155754	Intron	c.640-26T>C
401	C2orf42	chr2	ENSG00000115998	Missense	c.949A>C
402	C2orf68	chr2	ENSG00000168887	3'UTR	c.*313A>G
403	C2orf69	chr2	ENSG00000178074	3'UTR	c.*1271T>A
404	C3orf14	chr3	ENSG00000114405	3'UTR	c.*415del
405	C3orf14	chr3	ENSG00000114405	3'UTR	c.*858del
406	C3orf20	chr3	ENSG00000131379	Frame shift del	c.2367del
407	C3orf20	chr3	ENSG00000131379	Frame shift del	c.2367del
408	C3P1	chr19	ENSG00000167798	Intron	n.1342+42A>G
409	C4orf19	chr4	ENSG00000154274	3'UTR	c.*312C>A
410	C4orf54	chr4	ENSG00000248713	Frame shift del	c.4640del
411	C4orf54	chr4	ENSG00000248713	Frame shift del	c.4640del
412	C5	chr9	ENSG00000106804	Intron	c.4589-39T>C
413	C5orf15	chr5	ENSG00000113583	Missense	c.201T>A
414	C5orf51	chr5	ENSG00000205765	3'UTR	c.*4147del
415	C5orf67	chr5	ENSG00000225940	Intron	c.32+37A>G
416	C7	chr5	ENSG00000112936	Missense	c.2512T>C
417	C7orf31	chr7	ENSG00000153790	Intron	c.684+107G>A
418	C8orf34	chr8	ENSG00000165084	Intron	c.1277-70del
419	C8orf37	chr8	ENSG00000156172	Intron	c.244-14dup
420	C8orf82	chr8	ENSG00000213563	Missense	c.171A>G
421	C9orf153	chr9	ENSG00000187753	3'UTR	c.*316T>C
422	C9orf170	chr9	ENSG00000204446	RNA	n.1072del

Number	Gene name	Chromosome	Gene_ID	Mutation	Nucleotide substitution
423	C9orf64	chr9	ENSG00000165118	3'UTR	c.*50T>C
424	CA13	chr8	ENSG00000185015	3'UTR	c.*2359T>C
425	CA5B	chrX	ENSG00000169239	3'UTR	c.*4243T>C
426	CABIN1	chr22	ENSG00000099991	Intron	c.4747-8669A>G
427	CABIN1	chr22	ENSG00000099991	Silent	c.5145A>G
428	CABLES2	chr20	ENSG00000149679	3'UTR	c.*913del
429	CABP1	chr12	ENSG00000157782	Frame shift del	c.110_146del
430	CACNA1A	chr19	ENSG00000141837	Intron	c.4950+3644A>G
431	CACNA1C	chr12	ENSG00000151067	Intron	c.50-66G>C
432	CACNA1C	chr12	ENSG00000151067	Intron	c.1391-38C>T
433	CACNA1D	chr3	ENSG00000157388	Splice region	c.377+13del
434	CACNA1H	chr16	ENSG00000196557	Intron	c.4476+28G>T
435	CACNB1	chr17	ENSG00000067191	3'UTR	c.*629T>C
436	CACNB1	chr17	ENSG00000067191	Splice region	c.784-3dup
437	CACNB4	chr2	ENSG00000182389	Missense	c.1144A>T
438	CACNB4	chr2	ENSG00000182389	Intron	c.268-28A>C
439	CADM1	chr11	ENSG00000182985	3'UTR	c.*949del
440	CADM1	chr11	ENSG00000182985	Intron	c.1079-56del
441	CADM3	chr1	ENSG00000162706	Silent	c.63C>A
442	CADM3	chr1	ENSG00000162706	Intron	c.623-39C>G
443	CALCOCO1	chr12	ENSG00000012822	Missense	c.494A>G
444	CALCOCO1	chr12	ENSG00000012822	Silent	c.180T>C
445	CALHM1	chr10	ENSG00000185933	Silent	c.261C>T
446	CALHM3	chr10	ENSG00000183128	5'Flank	
447	CALN1	chr7	ENSG00000183166	3'UTR	c.*7077del
448	CAMK2N1	chr1	ENSG00000162545	3'UTR	c.*1115dup
449	CAMK4	chr5	ENSG00000152495	3'UTR	c.*6978del
450	CAMKV	chr3	ENSG00000164076	3'UTR	c.*1273C>T
451	CAMTA1	chr1	ENSG00000171735	Missense	c.4529A>G
452	CAPN1	chr11	ENSG00000014216	3'UTR	c.*260T>G
453	CAPN10	chr2	ENSG00000142330	Frame shift del	c.1326del
454	CAPN10	chr2	ENSG00000142330	Frame shift del	c.1326del
455	CAPN2	chr1	ENSG00000162909	5'UTR	c.-133del
456	CAPN2	chr1	ENSG00000162909	Missense	c.2050A>G
457	CAPN8	chr1	ENSG00000203697	Intron	c.1700-109C>T
458	CAPRIN2	chr12	ENSG00000110888	Intron	c.421-20T>C
459	CAPZB	chr1	ENSG00000077549	3'UTR	c.*713A>G
460	CAPZB	chr1	ENSG00000077549	Intron	c.330-23G>A
461	CARD11	chr7	ENSG00000198286	Intron	c.2607+79del
462	CARF	chr2	ENSG00000138380	3'UTR	c.*1228del
463	CARF	chr2	ENSG00000138380	Frame shift del	c.669del
464	CARF	chr2	ENSG00000138380	Frame shift del	c.669del
465	CARMIL2	chr16	ENSG00000159753	5'UTR	c.-51C>T
466	CASKIN2	chr17	ENSG00000177303	Missense	c.2651C>T
467	CASP2	chr7	ENSG00000106144	3'UTR	c.*1498_*1499del
468	CASP3	chr4	ENSG00000164305	Missense	c.590A>G
469	CASR	chr3	ENSG00000036828	5'UTR	c.-158G>T
470	CASS4	chr20	ENSG00000087589	5'UTR	c.-38A>G
471	CASTOR2	chr7	ENSG00000274070	Missense	c.383G>A
472	CASZ1	chr1	ENSG00000130940	3'UTR	c.*2124del
473	CATSPERG	chr19	ENSG00000099338	Intron	c.998-19C>T
474	CAV1	chr7	ENSG00000105974	5'Flank	
475	CAV1	chr7	ENSG00000105974	3'UTR	c.*22T>C

Number	Gene name	Chromosome	Gene_ID	Mutation	Nucleotide substitution
476	CBFA2T2	chr20	ENSG00000078699	3'UTR	c.*510C>T
477	CBLL1	chr7	ENSG00000105879	3'UTR	c.*281dup
478	CBLN4	chr20	ENSG00000054803	3'UTR	c.*856_*857del
479	CBR4	chr4	ENSG00000145439	3'UTR	c.*235del
480	CBWD1	chr9	ENSG00000172785	Missense	c.706T>C
481	CBX1	chr17	ENSG00000108468	3'UTR	c.*584del
482	CC2D1A	chr19	ENSG00000132024	Silent	c.1326A>C
483	CC2D1B	chr1	ENSG00000154222	Intron	c.2430+231A>G
484	CCAR2	chr8	ENSG00000158941	3'UTR	c.*566T>C
485	CCDC110	chr4	ENSG00000168491	Missense	c.1510T>C
486	CCDC117	chr22	ENSG00000159873	3'UTR	c.*1445A>G
487	CCDC144NL	chr17	ENSG00000205212	Missense	c.269C>T
488	CCDC148	chr2	ENSG00000153237	3'UTR	c.*938A>T
489	CCDC171	chr9	ENSG00000164989	Intron	c.353-3808del
490	CCDC25	chr8	ENSG00000147419	3'UTR	c.*1676del
491	CCDC30	chr1	ENSG00000186409	Intron	c.385-8981A>G
492	CCDC40	chr17	ENSG00000141519	Silent	c.2991A>G
493	CCDC68	chr18	ENSG00000166510	3'UTR	c.*1540del
494	CCDC71	chr3	ENSG00000177352	Missense	c.463C>T
495	CCDC73	chr11	ENSG00000186714	3'UTR	c.*1713T>C
496	CCDC85C	chr14	ENSG00000205476	3'UTR	c.*2817del
497	CCDC85C	chr14	ENSG00000205476	In_Frame_Del	c.1138_1140del
498	CCDC88A	chr2	ENSG00000115355	Intron	c.3603-9del
499	CCDC88C	chr14	ENSG00000015133	3'UTR	c.*422G>C
500	CCDC89	chr11	ENSG00000179071	3'UTR	c.*489del
501	CCKBR	chr11	ENSG00000110148	5'Flank	
502	CCKBR	chr11	ENSG00000110148	Missense	c.265A>G
503	CCL22	chr16	ENSG00000102962	3'UTR	c.*1232del
504	CCN2	chr6	ENSG00000118523	Missense	c.835T>C
505	CCNB1	chr5	ENSG00000134057	Intron	c.1194+44A>G
506	CCND2	chr12	ENSG00000118971	3'UTR	c.*3519dup
507	CCNE2	chr8	ENSG00000175305	3'UTR	c.*867del
508	CCNI2	chr5	ENSG00000205089	Missense	c.460G>A
509	CCT8	chr21	ENSG00000156261	Intron	c.60+1520del
510	CD163L1	chr12	ENSG00000177675	Intron	c.124+1934A>G
511	CD1D	chr1	ENSG00000158473	Missense	c.128G>A
512	CD300E	chr17	ENSG00000186407	3'UTR	c.*2702del
513	CD37	chr19	ENSG00000104894	Silent	c.390C>T
514	CD3G	chr11	ENSG00000160654	Intron	c.172-13C>T
515	CD44	chr11	ENSG00000026508	3'UTR	c.*331C>A
516	CD59	chr11	ENSG00000085063	Missense	c.251A>G
517	CD6	chr11	ENSG00000013725	Missense	c.521T>G
518	CD96	chr3	ENSG00000153283	Silent	c.349C>T
519	CDC123	chr10	ENSG00000151465	Intron	c.565+259T>C
520	CDC14B	chr9	ENSG00000081377	Intron	c.1344-11del
521	CDC25A	chr3	ENSG00000164045	3'UTR	c.*582del
522	CDC27	chr17	ENSG00000004897	Missense	c.1937A>G
523	CDC42	chr1	ENSG00000070831	Missense	c.169A>G
524	CDC42	chr1	ENSG00000070831	3'UTR	c.*1459G>A
525	CDC42BPB	chr14	ENSG00000198752	Silent	c.5064A>G
526	CDC42BPG	chr11	ENSG00000171219	Intron	c.4513+38del
527	CDC5L	chr6	ENSG00000096401	3'UTR	c.*3700del
528	CDC7	chr1	ENSG00000097046	3'UTR	c.*1278del

Number	Gene name	Chromosome	Gene_ID	Mutation	Nucleotide substitution
529	CDC73	chr1	ENSG00000134371	3'UTR	c.*1144del
530	CDCA5	chr11	ENSG00000146670	Intron	c.269+34_269+35del
531	CDH15	chr16	ENSG00000129910	Missense	c.1460C>T
532	CDH19	chr18	ENSG00000071991	Missense	c.2077G>A
533	CDH2	chr18	ENSG00000170558	Missense	c.79G>T
534	CDH20	chr18	ENSG00000101542	Intron	c.830-39C>T
535	CDH24	chr14	ENSG00000139880	Intron	c.1363+29A>G
536	CDH4	chr20	ENSG00000179242	Intron	c.-53-24204G>A
537	CDHR1	chr10	ENSG00000148600	Missense	c.2138A>G
538	CDHR2	chr5	ENSG00000074276	Missense	c.1637T>C
539	CDHR4	chr3	ENSG00000187492	Missense	c.421C>T
540	CDK15	chr2	ENSG00000138395	3'UTR	c.*631C>T
541	CDK16	chrX	ENSG00000102225	3'UTR	c.*507_*508del
542	CDK4	chr12	ENSG00000135446	5'UTR	c.-99del
543	CDK5RAP2	chr9	ENSG00000136861	Intron	c.508-58T>C
544	CDK6	chr7	ENSG00000105810	3'UTR	c.*9369del
545	CDK9	chr9	ENSG00000136807	3'UTR	c.*980del
546	CDKN2AIP	chr4	ENSG00000168564	Missense	c.1709C>A
547	CDKN2AIP	chr4	ENSG00000168564	3'UTR	c.*1424T>C
548	CDKN2B	chr9	ENSG00000147883	3'UTR	c.*766T>C
549	CDKN3	chr14	ENSG00000100526	Intron	c.148+639A>G
550	CDV3	chr3	ENSG00000091527	3'UTR	c.*46_*49del
551	CDV3	chr3	ENSG00000091527	3'UTR	c.*647dup
552	CDV3	chr3	ENSG00000091527	3'UTR	c.*1640C>T
553	CDX2	chr13	ENSG00000165556	5'Flank	
554	CEACAM20	chr19	ENSG00000273777	Intron	c.1428-47T>C
555	CELF2	chr10	ENSG00000048740	Intron	c.1015+3064_1015+3065del
556	CELF2	chr10	ENSG00000048740	3'UTR	c.*4914T>C
557	CELSR1	chr22	ENSG00000075275	Intron	c.5413-96T>C
558	CELSR1	chr22	ENSG00000075275	Frame shift Ins	c.175dup
559	CELSR2	chr1	ENSG00000143126	Silent	c.822A>C
560	CELSR3	chr3	ENSG00000008300	Missense	c.8501C>T
561	CELSR3	chr3	ENSG00000008300	Missense	c.6175T>C
562	CELSR3	chr3	ENSG00000008300	Missense	c.4070A>G
563	CEMIP	chr15	ENSG00000103888	Missense	c.3296G>A
564	CENPS	chr1	ENSG00000175279	Silent	c.153G>A
565	CENPV	chr17	ENSG00000166582	Intron	c.580-23A>G
566	CEP126	chr11	ENSG00000110318	3'UTR	c.*3064_*3065del
567	CEP164	chr11	ENSG00000110274	3'UTR	c.*1094del
568	CEP192	chr18	ENSG00000101639	Intron	c.3333+29A>T
569	CEP290	chr12	ENSG00000198707	Intron	c.1065+15G>A
570	CEP295	chr11	ENSG00000166004	Intron	c.5597-10del
571	CEP350	chr1	ENSG00000135837	3'UTR	c.*2094del
572	CEP41	chr7	ENSG00000106477	Splice region	c.33+4A>G
573	CEP70	chr3	ENSG00000114107	Missense	c.1324T>A
574	CEP78	chr9	ENSG00000148019	5'UTR	c.-251A>G
575	CEP78	chr9	ENSG00000148019	3'UTR	c.*8173G>T
576	CEP85L	chr6	ENSG00000111860	3'UTR	c.*3995del
577	CERS6	chr2	ENSG00000172292	3'UTR	c.*4992del
578	CES3	chr16	ENSG00000172828	3'UTR	c.*489_*492del
579	CES4A	chr16	ENSG00000172824	3'UTR	c.*560G>T
580	CETN3	chr5	ENSG00000153140	3'UTR	c.*270del
581	CFAP221	chr2	ENSG00000163075	Missense	c.893C>G

Number	Gene name	Chromosome	Gene_ID	Mutation	Nucleotide substitution
582	CFAP44	chr3	ENSG00000206530	3'UTR	c.*1367del
583	CFAP47	chrX	ENSG00000165164	3'UTR	c.*330T>C
584	CFHR3	chr1	ENSG00000116785	3'UTR	c.*22del
585	CFHR5	chr1	ENSG00000134389	Silent	c.732C>T
586	CFL2	chr14	ENSG00000165410	3'UTR	c.*2330A>G
587	CGAS	chr6	ENSG00000164430	Frame shift Ins	c.1284dup
588	CGAS	chr6	ENSG00000164430	Missense	c.1244A>G
589	CGN	chr1	ENSG00000143375	Intron	c.1045-33T>C
590	CGREF1	chr2	ENSG00000138028	Silent	c.63A>G
591	CHAD	chr17	ENSG00000136457	Silent	c.516C>T
592	CHAT	chr10	ENSG00000070748	Missense	c.349C>T
593	CHCHD1	chr10	ENSG00000172586	3'UTR	c.*268del
594	CHCHD1	chr10	ENSG00000172586	3'UTR	c.*273A>T
595	CHD1	chr5	ENSG00000153922	Missense	c.973T>C
596	CHD2	chr15	ENSG00000173575	3'UTR	c.*1684T>C
597	CHD2	chr15	ENSG00000173575	3'UTR	c.*186T>C
598	CHEK2	chr22	ENSG00000183765	Silent	c.240T>C
599	CHERP	chr19	ENSG00000085872	Missense	c.2723A>G
600	CHERP	chr19	ENSG00000085872	Silent	c.222G>A
601	CHMP7	chr8	ENSG00000147457	Frame shift del	c.968_974del
602	CHMP7	chr8	ENSG00000147457	Frame shift del	c.968_974del
603	CHN2	chr7	ENSG00000106069	Silent	c.1389C>T
604	CHRAC1	chr8	ENSG00000104472	3'UTR	c.*314C>T
605	CHRNA1	chr2	ENSG00000138435	Intron	c.853+750del
606	CHRNA1	chr2	ENSG00000138435	3'UTR	
607	CHRNA2	chr8	ENSG00000120903	Missense	c.1205T>A
608	CHST15	chr10	ENSG00000182022	3'UTR	c.*1925del
609	CHST2	chr3	ENSG00000175040	Splice region	c.-172T>C
610	CHST3	chr10	ENSG00000122863	Missense	c.239T>C
611	CHST5	chr16	ENSG00000135702	5'UTR	c.-188A>G
612	CHSY3	chr5	ENSG00000198108	5'UTR	c.-302_-300del
613	CIC	chr19	ENSG00000079432	Missense	c.1621T>C
614	CILP2	chr19	ENSG00000160161	Intron	c.65-100_65-99delinsGG
615	CILP2	chr19	ENSG00000160161	Missense	c.1964A>G
616	CIRBP	chr19	ENSG00000099622	Missense	c.665G>A
617	CIT	chr12	ENSG00000122966	Missense	c.2102G>A
618	CIZ1	chr9	ENSG00000148337	Missense	c.2066A>G
619	CKAP2L	chr2	ENSG00000169607	Intron	c.1758+9C>T
620	CKB	chr14	ENSG00000166165	Intron	c.348+25_349-35del
621	CLCA2	chr1	ENSG00000137975	Intron	c.1488+24T>C
622	CLCA4	chr1	ENSG00000016602	Intron	c.300+10G>A
623	CLCC1	chr1	ENSG00000121940	Splice site	c.-11-1G>A
624	CLCN2	chr3	ENSG00000114859	Frame shift del	c.1142del
625	CLCN2	chr3	ENSG00000114859	Frame shift del	c.1142del
626	CLCN3	chr4	ENSG00000109572	3'UTR	c.*3498del
627	CLCN4	chrX	ENSG00000073464	3'UTR	c.*3151_*3152del
628	CLCN5	chrX	ENSG00000171365	Intron	c.17-39078T>C
629	CLCN5	chrX	ENSG00000171365	3'UTR	c.*3109del
630	CLDN19	chr1	ENSG00000164007	Silent	c.30C>T
631	CLEC10A	chr17	ENSG00000132514	Missense	c.646G>T
632	CLEC3A	chr16	ENSG00000166509	3'UTR	c.*604del
633	CLIP3	chr19	ENSG00000105270	Missense	c.314G>A
634	CLMN	chr14	ENSG00000165959	Intron	c.2769+58T>C

Number	Gene name	Chromosome	Gene_ID	Mutation	Nucleotide substitution
635	CLOCK	chr4	ENSG00000134852	3'UTR	c.*1717A>G
636	CLPX	chr15	ENSG00000166855	3'UTR	c.*2089del
637	CLPX	chr15	ENSG00000166855	Missense	c.1330C>T
638	CLSPN	chr1	ENSG00000092853	Intron	c.895+66T>A
639	CLU	chr8	ENSG00000120885	3'UTR	c.*1030T>C
640	CLYBL	chr13	ENSG00000125246	Missense	c.727C>A
641	CMPK2	chr2	ENSG00000134326	Intron	c.993-7621A>G
642	CMSS1	chr3	ENSG00000184220	Intron	c.668-64del
643	CMTM6	chr3	ENSG00000091317	3'UTR	c.*2590dup
644	CMYA5	chr5	ENSG00000164309	Missense	c.613A>G
645	CNIH4	chr1	ENSG00000143771	3'UTR	c.*2397A>G
646	CNIH4	chr1	ENSG00000143771	3'UTR	c.*2539del
647	CNNM2	chr10	ENSG00000148842	Silent	c.987G>A
648	CNNM3	chr2	ENSG00000168763	3'UTR	c.*940del
649	CNOT1	chr16	ENSG00000125107	Missense	c.3418A>G
650	CNOT4	chr7	ENSG00000080802	3'UTR	c.*1238_*1239dup
651	CNOT6L	chr4	ENSG00000138767	3'UTR	c.*2835_*2838del
652	CNOT6L	chr4	ENSG00000138767	3'UTR	c.*2733A>G
653	CNOT6L	chr4	ENSG00000138767	Intron	c.1455+32T>A
654	CNOT6L	chr4	ENSG00000138767	Splice region	c.315-9_315-7del
655	CNOT9	chr2	ENSG00000144580	3'UTR	c.*1486_*1487del
656	CNST	chr1	ENSG00000162852	Missense	c.952C>A
657	CNTLN	chr9	ENSG00000044459	3'UTR	c.*208T>C
658	CNTNAP1	chr17	ENSG00000108797	Missense	c.2128C>T
659	CNTNAP5	chr2	ENSG00000155052	Silent	c.2007C>T
660	COA6	chr1	ENSG00000168275	3'UTR	c.*300A>G
661	COBL	chr7	ENSG00000106078	3'UTR	c.*1196del
662	COCH	chr14	ENSG00000100473	Missense	c.247G>T
663	COG6	chr13	ENSG00000133103	3'UTR	c.*1980T>A
664	COL10A1	chr6	ENSG00000123500	3'UTR	c.*328del
665	COL11A1	chr1	ENSG00000060718	Frame shift del	c.4543del
666	COL11A1	chr1	ENSG00000060718	Frame shift del	c.4543del
667	COL12A1	chr6	ENSG00000111799	Intron	c.9010+100_9010+101del
668	COL12A1	chr6	ENSG00000111799	Nonsense	c.2611C>T
669	COL16A1	chr1	ENSG00000084636	Missense	c.2584G>T
670	COL17A1	chr10	ENSG00000065618	Missense	c.325T>C
671	COL18A1	chr21	ENSG00000182871	Frame shift del	c.2181del
672	COL18A1	chr21	ENSG00000182871	Frame shift del	c.2181del
673	COL1A1	chr17	ENSG00000108821	3'UTR	c.*1015T>C
674	COL1A2	chr7	ENSG00000164692	3'UTR	c.*238C>T
675	COL22A1	chr8	ENSG00000169436	3'UTR	c.*668A>C
676	COL22A1	chr8	ENSG00000169436	Intron	c.3609+98T>C
677	COL27A1	chr9	ENSG00000196739	Splice region	c.4260+8A>G
678	COL27A1	chr9	ENSG00000196739	Missense	c.4811G>T
679	COL3A1	chr2	ENSG00000168542	Intron	c.1347+41del
680	COL4A4	chr2	ENSG00000081052	3'UTR	c.*2120del
681	COL4A4	chr2	ENSG00000081052	Splice region	c.976-8G>A
682	COL5A2	chr2	ENSG00000204262	Missense	c.1322G>T
683	COL6A2	chr21	ENSG00000142173	Intron	c.1179+14C>T
684	COL6A2	chr21	ENSG00000142173	Intron	c.1395+58del
685	COL6A2	chr21	ENSG00000142173	Intron	c.1734+34del
686	COL6A3	chr2	ENSG00000163359	3'UTR	c.*665del
687	COL8A1	chr3	ENSG00000144810	Intron	c.-3-81T>C

Number	Gene name	Chromosome	Gene_ID	Mutation	Nucleotide substitution
688	COL9A3	chr20	ENSG00000092758	Intron	c.79-13_79-12del
689	COMP	chr19	ENSG00000105664	Intron	c.390+52del
690	COP1	chr1	ENSG00000143207	5'UTR	c.-144del
691	COPA	chr1	ENSG00000122218	Missense	c.3568G>A
692	COPB2	chr3	ENSG00000184432	Missense	c.1888T>C
693	COPG1	chr3	ENSG00000181789	Intron	c.2257-640del
694	COPG2	chr7	ENSG00000158623	Intron	c.737+3113del
695	COQ6	chr14	ENSG00000119723	Intron	c.164-11_164-10del
696	CORO2A	chr9	ENSG00000106789	3'UTR	c.*2052del
697	CORO2A	chr9	ENSG00000106789	3'UTR	c.*1377T>C
698	COX6A1P2	chr6	ENSG00000226976	RNA	n.176T>C
699	CPA5	chr7	ENSG00000158525	Intron	c.432+76T>C
700	CPA5	chr7	ENSG00000158525	Silent	c.627T>C
701	CPAMD8	chr19	ENSG00000160111	Intron	c.505-145del
702	CPEB2	chr4	ENSG00000137449	Intron	c.1944+857T>C
703	CPEB2	chr4	ENSG00000137449	3'UTR	c.*2972del
704	CPED1	chr7	ENSG00000106034	Intron	c.249+4044G>C
705	CPNE3	chr8	ENSG00000085719	Missense	c.140G>A
706	CPNE3	chr8	ENSG00000085719	3'UTR	c.*1026del
707	CPNE7	chr16	ENSG00000178773	Missense	c.457T>C
708	CPOX	chr3	ENSG00000080819	Intron	c.1277+52C>A
709	CPSF1	chr8	ENSG00000071894	Frame shift del	c.884del
710	CPSF1	chr8	ENSG00000071894	Frame shift del	c.884del
711	CPSF7	chr11	ENSG00000149532	Intron	c.680-738A>G
712	CPTP	chr1	ENSG00000224051	Missense	c.337C>T
713	CPXM2	chr10	ENSG00000121898	3'UTR	c.*1106del
714	CR1	chr1	ENSG00000203710	Missense	c.4567G>A
715	CR1	chr1	ENSG00000203710	3'UTR	c.*727del
716	CRACR2B	chr11	ENSG00000177685	Missense	c.896A>G
717	CRAT	chr9	ENSG00000095321	Silent	c.348A>G
718	CRB1	chr1	ENSG00000134376	3'UTR	c.*2829G>A
719	CRB1	chr1	ENSG00000134376	3'UTR	c.*4460A>G
720	CRB1	chr1	ENSG00000134376	3'UTR	c.*463A>G
721	CREB3L2	chr7	ENSG00000182158	3'UTR	c.*3294del
722	CREBRF	chr5	ENSG00000164463	3'UTR	c.*4857A>G
723	CREBRF	chr5	ENSG00000164463	3'UTR	c.*4971del
724	CREBRF	chr5	ENSG00000164463	3'UTR	c.*5263_*5264del
725	CREG2	chr2	ENSG00000175874	3'UTR	c.*326_*327del
726	CRELD1	chr3	ENSG00000163703	Missense	c.610C>T
727	CRIM1	chr2	ENSG00000150938	3'UTR	c.*1365T>C
728	CRIPAK	chr4	ENSG00000288380	5'UTR	c.-1322del
729	CROCC	chr1	ENSG00000058453	5'UTR	c.-1del
730	CRY1	chr12	ENSG00000008405	Missense	c.1042C>T
731	CRYAA	chr21	ENSG00000160202	Frame shift del	c.96_97del
732	CRYBG1	chr6	ENSG00000112297	Missense	c.641T>C
733	CRYZ	chr1	ENSG00000116791	3'UTR	c.*359C>A
734	CSAD	chr12	ENSG00000139631	Intron	c.648+125G>A
735	CSAD	chr12	ENSG00000139631	Missense	c.542T>C
736	CSMD1	chr8	ENSG00000183117	Splice region	c.6819-7del
737	CSMD1	chr8	ENSG00000183117	Intron	c.4166+36T>C
738	CSMD1	chr8	ENSG00000183117	Missense	c.254G>T
739	CSMD2	chr1	ENSG00000121904	Missense	c.97G>A
740	CSMD3	chr8	ENSG00000164796	Missense	c.4721G>A

Number	Gene name	Chromosome	Gene_ID	Mutation	Nucleotide substitution
741	CSNK1A1	chr5	ENSG00000113712	3'UTR	c.*2983_*2984del
742	CSNK1G1	chr15	ENSG00000169118	3'UTR	c.*4834A>G
743	CSNK1G2	chr19	ENSG00000133275	Intron	c.448-6del
744	CSNK1G3	chr5	ENSG00000151292	3'UTR	c.*1610del
745	CSPP1	chr8	ENSG00000104218	Intron	c.207+447del
746	CSPP1	chr8	ENSG00000104218	Missense	c.3037C>G
747	CTAGE9	chr6	ENSG00000236761	Missense	c.522A>G
748	CTDSPL	chr3	ENSG00000144677	3'UTR	c.*3106del
749	CTDSPL2	chr15	ENSG00000137770	3'UTR	c.*1811dup
750	CTNNA2	chr2	ENSG00000066032	Missense	c.1422G>T
751	CTR9	chr11	ENSG00000198730	5'UTR	c.-72G>T
752	CTR9	chr11	ENSG00000198730	Missense	c.3359A>G
753	CTRL	chr16	ENSG00000141086	Intron	c.319-59A>G
754	CTTNBP2	chr7	ENSG00000077063	Intron	c.2272+11C>T
755	CTTNBP2NL	chr1	ENSG00000143079	3'UTR	c.*2165del
756	CUBN	chr10	ENSG00000107611	Silent	c.10251T>C
757	CUL1	chr7	ENSG00000055130	Intron	c.1480-39del
758	CUL7	chr6	ENSG00000044090	Intron	c.2398-25_2398-24insA
759	CUX1	chr7	ENSG00000257923	Intron	c.141+16437A>C
760	CWC22	chr2	ENSG00000163510	Intron	c.1459-10del
761	CWC22	chr2	ENSG00000163510	Splice region	c.1148-3del
762	CXCL12	chr10	ENSG00000107562	3'UTR	c.*1236del
763	CXCL5	chr4	ENSG00000163735	3'UTR	c.*758T>C
764	CXorf58	chrX	ENSG00000165182	3'UTR	c.*100del
765	CYBRD1	chr2	ENSG00000071967	5'UTR	c.-69A>G
766	CYHR1	chr8	ENSG00000187954	Intron	c.247-3852G>T
767	CYP1A2	chr15	ENSG00000140505	Missense	c.1459G>A
768	CYP2B7P	chr19	ENSG00000256612	Splice region	n.389-4G>A
769	CYP3A7	chr7	ENSG00000160870	Missense	c.539G>A
770	P3A7-CYP3A:	chr7	ENSG00000282301	Frame shift del	c.1566del
771	CYP4B1	chr1	ENSG00000142973	Silent	c.969T>C
772	CYP4F12	chr19	ENSG00000186204	3'UTR	c.*27dup
773	CYS1	chr2	ENSG00000205795	Frame shift del	c.66del
774	CYS1	chr2	ENSG00000205795	Frame shift del	c.66del
775	CYTH1	chr17	ENSG00000108669	3'UTR	c.*408del
776	CYYR1	chr21	ENSG00000166265	Intron	c.176+1417del
777	CYYR1	chr21	ENSG00000166265	Missense	c.22G>A
778	DAAM2	chr6	ENSG00000146122	3'UTR	c.*2779dup
779	DACT2	chr6	ENSG00000164488	Missense	c.1859G>A
780	DAGLA	chr11	ENSG00000134780	Missense	c.1264C>T
781	DAGLB	chr7	ENSG00000164535	Missense	c.1888C>T
782	DBF4B	chr17	ENSG00000161692	Intron	c.1190-203del
783	DBP	chr19	ENSG00000105516	3'UTR	c.*166del
784	DBX2	chr12	ENSG00000185610	3'UTR	c.*1442G>A
785	DCAF1	chr3	ENSG00000145041	Silent	c.1896T>C
786	DCAF10	chr9	ENSG00000122741	Intron	c.135-4344T>G
787	DCAF8L1	chrX	ENSG00000226372	Missense	c.945C>A
788	DCHS1	chr11	ENSG00000166341	Missense	c.2597G>A
789	DCLK1	chr13	ENSG00000133083	3'UTR	c.*1966del
790	DCP2	chr5	ENSG00000172795	3'UTR	c.*478G>A
791	DCP2	chr5	ENSG00000172795	3'UTR	c.*1888del
792	DCTN1	chr2	ENSG00000204843	3'UTR	c.*170G>A
793	DCTN1	chr2	ENSG00000204843	Intron	c.2316+48T>C

Number	Gene name	Chromosome	Gene_ID	Mutation	Nucleotide substitution
794	DCTN6	chr8	ENSG00000104671	3'UTR	c.*4del
795	DCUN1D1	chr3	ENSG00000043093	3'UTR	c.*2444G>C
796	DDAH1	chr1	ENSG00000153904	3'UTR	c.*1675del
797	DDB1	chr11	ENSG00000167986	Missense	c.3146A>G
798	DDB1	chr11	ENSG00000167986	Intron	c.922-262T>C
799	DDB1	chr11	ENSG00000167986	Intron	c.922-327dup
800	DDB2	chr11	ENSG00000134574	Intron	c.457-3058del
801	DDHD2	chr8	ENSG00000085788	5'UTR	c.-709A>G
802	DDI1	chr11	ENSG00000170967	Missense	c.352C>T
803	DDR2	chr1	ENSG00000162733	3'UTR	c.*225G>C
804	DDX1	chr2	ENSG00000079785	Missense	c.1253T>C
805	DDX20	chr1	ENSG00000064703	Silent	c.513A>G
806	DDX23	chr12	ENSG00000174243	Intron	c.1803+20C>T
807	DDX3X	chrX	ENSG00000215301	Missense	c.1124G>A
808	DDX46	chr5	ENSG00000145833	3'UTR	c.*1477_*1478del
809	DDX58	chr9	ENSG00000107201	Intron	c.767-222A>G
810	DDX60L	chr4	ENSG00000181381	Missense	c.477C>A
811	DEGS1	chr1	ENSG00000143753	3'UTR	c.*869A>G
812	DENND1B	chr1	ENSG00000213047	Intron	c.-160+49del
813	DENND5A	chr11	ENSG00000184014	Missense	c.3710T>C
814	DENND5A	chr11	ENSG00000184014	Intron	c.182-319del
815	DENND5B	chr12	ENSG00000170456	3'UTR	c.*2898A>G
816	DENND5B	chr12	ENSG00000170456	Missense	c.667A>G
817	DENND6A	chr3	ENSG00000174839	3'UTR	c.*2573del
818	DENR	chr12	ENSG00000139726	3'UTR	c.*1479A>G
819	DEPDC5	chr22	ENSG00000100150	Intron	c.2105-4368del
820	DERL1	chr8	ENSG00000136986	3'UTR	c.*486G>A
821	DERL1	chr8	ENSG00000136986	Intron	c.617+50A>G
822	DERL2	chr17	ENSG00000072849	5'Flank	
823	DESI1	chr22	ENSG00000100418	3'UTR	c.*2961A>G
824	DFFA	chr1	ENSG00000160049	3'UTR	c.*1758T>A
825	DFFA	chr1	ENSG00000160049	3'UTR	c.*1602del
826	DFFA	chr1	ENSG00000160049	3'UTR	c.*1227del
827	DFFB	chr1	ENSG00000169598	Silent	c.475C>A
828	DGAT1	chr8	ENSG00000185000	Missense	c.1078T>C
829	DGKB	chr7	ENSG00000136267	3'UTR	c.*3761G>A
830	DGKG	chr3	ENSG00000058866	Intron	c.-248-40del
831	DGKI	chr7	ENSG00000157680	Intron	c.876+32C>T
832	DGKQ	chr4	ENSG00000145214	Intron	c.2728-59G>A
833	DHDH	chr19	ENSG00000104808	Frame shift Ins	c.790dup
834	DHRS7	chr14	ENSG00000100612	Missense	c.982T>C
835	DHX30	chr3	ENSG00000132153	Frame shift del	c.3025del
836	DHX30	chr3	ENSG00000132153	Frame shift del	c.3025del
837	DHX32	chr10	ENSG00000089876	5'UTR	c.-339del
838	DHX33	chr17	ENSG00000005100	3'UTR	c.*3078del
839	DIP2C	chr10	ENSG00000151240	Intron	c.2985+76_2985+78del
840	DIPK1A	chr1	ENSG00000154511	3'UTR	c.*72del
841	DIPK1B	chr9	ENSG00000165716	5'UTR	c.-189_-188del
842	DIPK2A	chr3	ENSG00000181744	3'UTR	c.*459T>C
843	DISC1	chr1	ENSG00000162946	3'UTR	c.*888T>C
844	DISC1	chr1	ENSG00000162946	3'UTR	c.*99G>A
845	DLD	chr7	ENSG00000091140	Missense	c.109G>A
846	DLEC1	chr3	ENSG00000008226	Silent	c.708T>C

Number	Gene name	Chromosome	Gene_ID	Mutation	Nucleotide substitution
847	DLG1	chr3	ENSG00000075711	Intron	c.2005-22del
848	DLG2	chr11	ENSG00000150672	Intron	c.858+3317G>T
849	DLG2	chr11	ENSG00000150672	Intron	c.322-22019_322-22016del
850	DLG2	chr11	ENSG00000150672	5'UTR	c.-313T>C
851	DLG4	chr17	ENSG00000132535	3'UTR	c.*495del
852	DLGAP5	chr14	ENSG00000126787	Intron	c.2064-19T>C
853	DLX3	chr17	ENSG00000064195	3'UTR	c.*1224del
854	DMBT1	chr10	ENSG00000187908	3'UTR	c.*249T>C
855	DMRT1	chr9	ENSG00000137090	3'UTR	c.*242del
856	DMRT3	chr9	ENSG00000064218	Missense	c.5A>G
857	DMTN	chr8	ENSG00000158856	3'UTR	c.*855del
858	DNAAF3	chr19	ENSG00000167646	Missense	c.1264T>C
859	DNAH1	chr3	ENSG00000114841	Missense	c.2407A>G
860	DNAH10	chr12	ENSG00000197653	Missense	c.1361G>A
861	DNAH11	chr7	ENSG00000105877	Intron	c.5482-1020del
862	DNAH11	chr7	ENSG00000105877	Missense	c.5525A>G
863	DNAH17	chr17	ENSG00000187775	Frame shift Ins	c.12390dup
864	DNAH5	chr5	ENSG00000039139	Splice region	c.6062-5C>T
865	DNAI2	chr17	ENSG00000171595	Silent	c.1713G>A
866	DNAJA1	chr9	ENSG00000086061	Missense	c.101A>G
867	DNAJA1	chr9	ENSG00000086061	In_Frame_Del	c.717_719del
868	DNAJB14	chr4	ENSG00000164031	3'UTR	c.*1225del
869	DNAJB2	chr2	ENSG00000135924	3'UTR	c.*1574G>T
870	DNAJC18	chr5	ENSG00000170464	3'UTR	c.*1860del
871	DNAJC6	chr1	ENSG00000116675	Intron	c.334-68G>A
872	DNAJC8	chr1	ENSG00000126698	Intron	c.400-11del
873	DNASE1	chr16	ENSG00000213918	Missense	c.442A>G
874	DNASE1	chr16	ENSG00000213918	3'UTR	c.*4844C>T
875	DNLZ	chr9	ENSG00000213221	3'UTR	c.*1953G>A
876	DNM1	chr9	ENSG00000106976	Missense	c.1868C>T
877	DNMT1	chr19	ENSG00000130816	Splice site	c.3346+2T>C
878	DNMT3B	chr20	ENSG00000088305	Missense	c.479G>A
879	DOC2A	chr16	ENSG00000149927	Missense	c.133G>A
880	DOCK1	chr10	ENSG00000150760	Missense	c.2924T>C
881	DOCK10	chr2	ENSG00000135905	Splice region	c.243+3A>T
882	DOCK2	chr5	ENSG00000134516	Missense	c.170G>A
883	DOCK2	chr5	ENSG00000134516	Missense	c.4836G>T
884	DOCK3	chr3	ENSG00000088538	Intron	c.465-25T>G
885	DOCK5	chr8	ENSG00000147459	Intron	c.224+32del
886	DOCK5	chr8	ENSG00000147459	3'UTR	c.*3378C>T
887	DOCK6	chr19	ENSG00000130158	Intron	c.378-64A>G
888	DOCK7	chr1	ENSG00000116641	Missense	c.2525A>G
889	DOCK7	chr1	ENSG00000116641	3'UTR	c.*373del
890	DOP1B	chr21	ENSG00000142197	Splice region	c.1370+7A>T
891	DPF3	chr14	ENSG00000205683	Intron	c.526-37A>G
892	DPH5	chr1	ENSG00000117543	Missense	c.791A>G
893	DPP4	chr2	ENSG00000197635	Intron	c.286-57C>T
894	DPP6	chr7	ENSG00000130226	5'UTR	c.-182T>C
895	DPPA4	chr3	ENSG00000121570	3'UTR	c.*1402del
896	DPY19L2P2	chr7	ENSG00000170629	RNA	n.371T>C
897	DQX1	chr2	ENSG00000144045	Intron	c.1145-25_1145-23del
898	DRAM2	chr1	ENSG00000156171	3'UTR	c.*600del
899	DRAXIN	chr1	ENSG00000162490	Intron	c.758-55T>G

Number	Gene name	Chromosome	Gene_ID	Mutation	Nucleotide substitution
900	DSC1	chr18	ENSG00000134765	Intron	c.148+97G>A
901	DSE	chr6	ENSG00000111817	3'UTR	c.*91del
902	DSG3	chr18	ENSG00000134757	Missense	c.659C>A
903	DSP	chr6	ENSG00000096696	Silent	c.5494C>T
904	DST	chr6	ENSG00000151914	Frame shift Ins	c.3518dup
905	DSTN	chr20	ENSG00000125868	Frame shift del	c.213del
906	DTX2	chr7	ENSG00000091073	Intron	c.269-35T>C
907	DUSP10	chr1	ENSG00000143507	Missense	c.1088A>G
908	DUSP11	chr2	ENSG00000144048	Intron	c.451-13T>C
909	DUSP4	chr8	ENSG00000120875	3'UTR	c.*301del
910	DUSP4	chr8	ENSG00000120875	Silent	c.1041G>A
911	DUSP7	chr3	ENSG00000164086	Missense	c.279G>T
912	DUSP9	chrX	ENSG00000130829	3'UTR	c.*564del
913	DUT	chr15	ENSG00000128951	3'UTR	c.*147del
914	DUT	chr15	ENSG00000128951	3'UTR	c.*326del
915	DYNAP	chr18	ENSG00000178690	3'UTR	c.*2T>G
916	DYNC1LI1	chr3	ENSG00000144635	3'UTR	c.*813A>G
917	DYNC2H1	chr11	ENSG00000187240	Silent	c.2739A>G
918	DYNC2LI1	chr2	ENSG00000138036	Silent	c.327T>C
919	DYNLL1	chr12	ENSG00000088986	Missense	c.76A>G
920	E2F3	chr6	ENSG00000112242	Intron	c.393+1345T>C
921	EBF2	chr8	ENSG00000221818	5'UTR	c.-418del
922	EBF3	chr10	ENSG00000108001	3'UTR	c.*2080del
923	ECEL1	chr2	ENSG00000171551	Missense	c.133G>A
924	ECHDC3	chr10	ENSG00000134463	Missense	c.457G>A
925	ECPAS	chr9	ENSG00000136813	Intron	c.1383-60del
926	ECRG4	chr2	ENSG00000119147	3'UTR	c.*189T>C
927	ECT2L	chr6	ENSG00000203734	Missense	c.1968G>C
928	EDEM1	chr3	ENSG00000134109	Intron	c.1338+73del
929	EDEM3	chr1	ENSG00000116406	3'UTR	c.*1264_*1265del
930	EDEM3	chr1	ENSG00000116406	3'UTR	c.*420T>A
931	EDNRA	chr4	ENSG00000151617	3'UTR	c.*568del
932	EEF1A1	chr6	ENSG00000156508	3'UTR	c.*1991_*1992del
933	EEF1A1	chr6	ENSG00000156508	3'UTR	c.*1206T>C
934	EEF1AKMT1	chr13	ENSG00000150456	Missense	c.170A>G
935	EEF2KMT	chr16	ENSG00000118894	Missense	c.32T>A
936	EFCAB11	chr14	ENSG00000140025	3'UTR	c.*945_*946del
937	EFHB	chr3	ENSG00000163576	In_Frame_Del	c.2107_2109del
938	EFHB	chr3	ENSG00000163576	In_Frame_Del	c.2107_2109del
939	EFHC2	chrX	ENSG00000183690	3'UTR	c.*753del
940	EFNB2	chr13	ENSG00000125266	Missense	c.880A>G
941	EFTUD2	chr17	ENSG00000108883	Missense	c.1039G>A
942	EGFR	chr7	ENSG00000146648	3'UTR	c.*132A>G
943	EGLN1	chr1	ENSG00000135766	Intron	c.1217-24_1217-23del
944	EGLN3	chr14	ENSG00000129521	Missense	c.98A>G
945	EHD2	chr19	ENSG00000024422	Missense	c.1535T>C
946	EIF1	chr17	ENSG00000173812	3'Flank	
947	EIF1AX	chrX	ENSG00000173674	3'UTR	c.*374G>A
948	EIF2A	chr3	ENSG00000144895	Intron	c.1612-19T>C
949	EIF2AK2	chr2	ENSG00000055332	Splice site	c.687+2T>C
950	EIF2AK3	chr2	ENSG00000172071	Silent	c.360T>A
951	EIF2AK4	chr15	ENSG00000128829	Missense	c.3776A>G
952	EIF3C	chr16	ENSG00000184110	Intron	c.-30-11345G>A

Number	Gene name	Chromosome	Gene_ID	Mutation	Nucleotide substitution
953	EIF3F	chr11	ENSG00000175390	3'UTR	c.*4197_*4198del
954	EIF3F	chr11	ENSG00000175390	3'UTR	c.*4799del
955	EIF3L	chr22	ENSG00000100129	Missense	c.1258A>G
956	EIF3L	chr22	ENSG00000100129	3'UTR	c.*870A>C
957	EIF4A2	chr3	ENSG00000156976	Intron	c.517+23T>C
958	EIF4E	chr4	ENSG00000151247	Intron	c.281+56del
959	EIF4E3	chr3	ENSG00000163412	3'UTR	c.*1588T>C
960	EIF4E3	chr3	ENSG00000163412	3'UTR	c.*624del
961	EIF4EBP2	chr10	ENSG00000148730	3'UTR	c.*2871T>C
962	EIF4EBP2	chr10	ENSG00000148730	3'UTR	c.*6589C>A
963	EIF4G3	chr1	ENSG00000075151	Missense	c.4420G>T
964	EIF4G3	chr1	ENSG00000075151	Intron	c.2943+18_2943+19del
965	EIF4G3	chr1	ENSG00000075151	Intron	c.31-25_31-23del
966	EIF4H	chr7	ENSG00000106682	5'Flank	
967	EIF5A	chr17	ENSG00000132507	Missense	c.374A>G
968	EIF5B	chr2	ENSG00000158417	Frame shift Ins	c.374dup
969	ELK1	chrX	ENSG00000126767	3'UTR	c.*744del
970	ELMO2	chr20	ENSG00000062598	Missense	c.865C>T
971	ELMSAN1	chr14	ENSG00000156030	Silent	c.3201T>C
972	ELOA	chr1	ENSG00000011007	5'UTR	c.-270T>G
973	ELOVL2	chr6	ENSG00000197977	3'UTR	c.*1488del
974	ELP5	chr17	ENSG00000170291	Missense	c.427C>T
975	EMC1	chr1	ENSG00000127463	Missense	c.1862C>A
976	EMC3	chr3	ENSG00000125037	3'UTR	c.*53G>A
977	EML3	chr11	ENSG00000149499	Intron	c.2359+95T>C
978	EMX2	chr10	ENSG00000170370	3'UTR	c.*137del
979	EN2	chr7	ENSG00000164778	3'UTR	c.*199del
980	EN2	chr7	ENSG00000164778	3'UTR	c.*1969A>G
981	ENDOU	chr12	ENSG00000111405	Splice region	c.628+6T>C
982	ENGASE	chr17	ENSG00000167280	Splice region	c.442-6T>C
983	ENO1	chr1	ENSG00000074800	Intron	c.445-31G>T
984	ENPP1	chr6	ENSG00000197594	3'UTR	c.*495del
985	ENPP1	chr6	ENSG00000197594	3'UTR	c.*3217del
986	ENSA	chr1	ENSG00000143420	3'UTR	c.*1109dup
987	ENSA	chr1	ENSG00000143420	3'UTR	c.*981G>T
988	ENTPD6	chr20	ENSG00000197586	Intron	c.658+23_658+24del
989	ENTPD6	chr20	ENSG00000197586	Missense	c.1198C>T
990	ENTR1	chr9	ENSG00000165689	Missense	c.130A>G
991	EOMES	chr3	ENSG00000163508	Intron	c.1380-96_1380-94del
992	EOMES	chr3	ENSG00000163508	Intron	c.1380-96_1380-95del
993	EP400	chr12	ENSG00000183495	Silent	c.2850C>T
994	EPB41L2	chr6	ENSG00000079819	Missense	c.1756G>A
995	EPB41L4B	chr9	ENSG00000095203	3'UTR	c.*1509del
996	EPC1	chr10	ENSG00000120616	Missense	c.222A>G
997	EPG5	chr18	ENSG00000152223	3'UTR	c.*4562A>G
998	EPG5	chr18	ENSG00000152223	Missense	c.6286A>G
999	EPHA10	chr1	ENSG00000183317	3'UTR	c.*1514T>C
1000	EPHA4	chr2	ENSG00000116106	Missense	c.2023G>A
1001	EPHB2	chr1	ENSG00000133216	Missense	c.2249A>G
1002	EPHX2	chr8	ENSG00000120915	5'UTR	c.-148C>T
1003	EPN2	chr17	ENSG00000072134	3'UTR	c.*930del
1004	EPPK1	chr8	ENSG00000261150	Missense	c.4886A>G
1005	EPS8	chr12	ENSG00000151491	Missense	c.1192G>A

Number	Gene name	Chromosome	Gene_ID	Mutation	Nucleotide substitution
1006	EPS8L2	chr11	ENSG00000177106	Silent	c.711T>C
1007	ERAP1	chr5	ENSG00000164307	Intron	c.2818+5749C>A
1008	ERBB3	chr12	ENSG00000065361	Splice site	c.-104-2A>C
1009	ERBB4	chr2	ENSG00000178568	3'UTR	c.*4387del
1010	ERBB4	chr2	ENSG00000178568	3'UTR	c.*3252T>C
1011	ERBIN	chr5	ENSG00000112851	3'UTR	c.*3089del
1012	ERC1	chr12	ENSG00000082805	Intron	c.*9-53T>C
1013	ERCC1	chr19	ENSG00000012061	Missense	c.638A>G
1014	ERCC2	chr19	ENSG00000104884	Splice region	c.1833G>A
1015	ERCC3	chr2	ENSG00000163161	Missense	c.1495A>G
1016	ERCC6	chr10	ENSG00000225830	Missense	c.1339G>A
1017	ERG	chr21	ENSG00000157554	3'UTR	c.*3121A>G
1018	ERG28	chr14	ENSG00000133935	Missense	c.67A>G
1019	ERGIC2	chr12	ENSG00000087502	3'UTR	c.*366del
1020	ERGIC3	chr20	ENSG00000125991	3'UTR	c.*239dup
1021	ERICH5	chr8	ENSG00000177459	3'UTR	c.*144G>T
1022	ERICH6	chr3	ENSG00000163645	Intron	c.404-645_404-644del
1023	ERLIN1	chr10	ENSG00000107566	Silent	c.840G>A
1024	ERMP1	chr9	ENSG00000099219	Silent	c.1080G>A
1025	ERP27	chr12	ENSG00000139055	Intron	c.333+5717del
1026	ESPL1	chr12	ENSG00000135476	Silent	c.1782G>A
1027	ESPN	chr1	ENSG00000187017	Silent	c.1428A>G
1028	ESR1	chr6	ENSG00000091831	3'UTR	c.*2550del
1029	ESRP1	chr8	ENSG00000104413	3'UTR	c.*1386del
1030	ESYT1	chr12	ENSG00000139641	Intron	c.1381-36G>A
1031	ETHE1	chr19	ENSG00000105755	Silent	c.720T>C
1032	ETS2	chr21	ENSG00000157557	3'UTR	c.*1772T>C
1033	ETV1	chr7	ENSG00000006468	3'UTR	c.*136C>A
1034	ETV1	chr7	ENSG00000006468	Intron	c.749-70A>G
1035	ETV3	chr1	ENSG00000117036	3'UTR	c.*304G>A
1036	ETV3	chr1	ENSG00000117036	Intron	c.47-44A>G
1037	ETV5	chr3	ENSG00000244405	3'UTR	c.*1965_*1968del
1038	EVC	chr4	ENSG00000072840	3'UTR	c.*2167A>G
1039	EVI5	chr1	ENSG00000067208	3'UTR	c.*3166G>T
1040	EVX2	chr2	ENSG00000174279	3'UTR	c.*419del
1041	EWSR1	chr22	ENSG00000182944	Intron	c.1180+54A>C
1042	EXD3	chr9	ENSG00000187609	Nonsense	c.88C>T
1043	EXOC3	chr5	ENSG00000180104	Missense	c.1822C>T
1044	EXOC5	chr14	ENSG00000070367	3'UTR	c.*3729T>C
1045	EXOC5	chr14	ENSG00000070367	3'UTR	c.*2989del
1046	EXOC5	chr14	ENSG00000070367	3'UTR	c.*2226del
1047	EXOC6	chr10	ENSG00000138190	3'UTR	c.*1027del
1048	EXOC8	chr1	ENSG00000116903	Missense	c.883G>A
1049	EXT1	chr8	ENSG00000182197	3'UTR	c.*4368T>C
1050	EXTL1	chr1	ENSG00000158008	3'UTR	c.*585C>T
1051	EYA1	chr8	ENSG00000104313	3'UTR	c.*736A>G
1052	EYA1	chr8	ENSG00000104313	Missense	c.1214C>G
1053	EYA4	chr6	ENSG00000112319	Intron	c.371-12del
1054	EYA4	chr6	ENSG00000112319	Missense	c.875C>T
1055	F11R	chr1	ENSG00000158769	Missense	c.31C>A
1056	FA2H	chr16	ENSG00000103089	3'UTR	c.*876del
1057	FABP5	chr8	ENSG00000164687	Intron	c.79+15C>A
1058	FABP7	chr6	ENSG00000164434	3'UTR	c.*1512del

Number	Gene name	Chromosome	Gene_ID	Mutation	Nucleotide substitution
1059	FABP7	chr6	ENSG00000164434	3'UTR	c.*225_*226del
1060	FAF2	chr5	ENSG00000113194	3'UTR	c.*1258del
1061	FAH	chr15	ENSG00000103876	Intron	c.455+836G>A
1062	FAHD2A	chr2	ENSG00000115042	3'UTR	c.*1958dup
1063	FAM107B	chr10	ENSG00000065809	3'UTR	c.*2016A>G
1064	FAM126B	chr2	ENSG00000155744	Intron	c.-129+7301del
1065	FAM131A	chr3	ENSG00000175182	Missense	c.140T>C
1066	FAM135B	chr8	ENSG00000147724	3'UTR	c.*2056G>A
1067	FAM13B	chr5	ENSG00000031003	Missense	c.1378G>A
1068	FAM160A2	chr11	ENSG00000051009	Missense	c.2414C>A
1069	FAM160B1	chr10	ENSG00000151553	Missense	c.890A>G
1070	FAM161A	chr2	ENSG00000170264	3'UTR	c.*828del
1071	FAM161B	chr14	ENSG00000156050	Missense	c.484G>A
1072	FAM169A	chr5	ENSG00000198780	3'UTR	c.*3342del
1073	FAM169B	chr15	ENSG00000283597	3'Flank	
1074	FAM171A2	chr17	ENSG00000161682	Intron	c.778+20G>A
1075	FAM171B	chr2	ENSG00000144369	5'Flank	
1076	FAM177B	chr1	ENSG00000197520	3'UTR	c.*411G>A
1077	FAM178B	chr2	ENSG00000168754	Intron	c.74-5227G>C
1078	FAM186A	chr12	ENSG00000185958	Intron	c.6848+60del
1079	FAM192A	chr16	ENSG00000172775	Intron	c.547+38T>C
1080	FAM193A	chr4	ENSG00000125386	3'UTR	c.*323T>C
1081	FAM193B	chr5	ENSG00000146067	Intron	c.-129-257A>G
1082	FAM204A	chr10	ENSG00000165669	3'UTR	c.*3297del
1083	FAM205BP	chr9	ENSG00000257198	RNA	n.2762C>T
1084	FAM214B	chr9	ENSG00000005238	Frame shift Ins	c.124dup
1085	FAM25G	chr10	ENSG00000189090	Missense	c.125C>T
1086	FAM3A	chrX	ENSG00000071889	Missense	c.548T>C
1087	FAM43B	chr1	ENSG00000183114	5'Flank	
1088	FAM71A	chr1	ENSG00000162771	Silent	c.1155A>G
1089	FAM71E2	chr19	ENSG00000180043	Silent	c.2070C>A
1090	FAM76B	chr11	ENSG00000077458	3'UTR	c.*731A>G
1091	FAM91A1	chr8	ENSG00000176853	5'UTR	c.-55A>G
1092	FAM91A1	chr8	ENSG00000176853	3'UTR	c.*2422del
1093	FANCE	chr6	ENSG00000112039	Missense	c.716T>C
1094	FANCF	chr11	ENSG00000183161	3'Flank	
1095	FANCM	chr14	ENSG00000187790	Intron	c.2160+39del
1096	FAR1	chr11	ENSG00000197601	5'UTR	c.-1631A>G
1097	FARP1	chr13	ENSG00000152767	Missense	c.28C>A
1098	FARSA	chr19	ENSG00000179115	Silent	c.909C>T
1099	FARSA	chr19	ENSG00000179115	Intron	c.504-24T>C
1100	FAS	chr10	ENSG00000026103	Intron	c.676+47T>C
1101	FBN1	chr15	ENSG00000166147	Missense	c.8402A>G
1102	FBN1	chr15	ENSG00000166147	Missense	c.3656A>G
1103	FBN3	chr19	ENSG00000142449	Frame shift Ins	c.2259dup
1104	FBN3	chr19	ENSG00000142449	Missense	c.142C>T
1105	FBRS	chr16	ENSG00000156860	5'UTR	c.-208del
1106	FBXO11	chr2	ENSG00000138081	Missense	c.167A>G
1107	FBXO22	chr15	ENSG00000167196	Intron	c.279+72dup
1108	FBXO28	chr1	ENSG00000143756	3'UTR	c.*435del
1109	FBXO32	chr8	ENSG00000156804	3'UTR	c.*3720T>C
1110	FBXO42	chr1	ENSG00000037637	3'UTR	c.*1723A>G
1111	FBXO9	chr6	ENSG00000112146	3'UTR	c.*2359dup

Number	Gene name	Chromosome	Gene_ID	Mutation	Nucleotide substitution
1112	FBXW12	chr3	ENSG00000164049	Intron	c.986-17G>A
1113	FCER2	chr19	ENSG00000104921	Missense	c.566A>G
1114	FCHO1	chr19	ENSG00000130475	Missense	c.1994G>A
1115	FCHO2	chr5	ENSG00000157107	3'UTR	c.*2130del
1116	FCRL4	chr1	ENSG00000163518	Missense	c.539A>C
1117	FCRL5	chr1	ENSG00000143297	3'UTR	c.*2107T>A
1118	FCRL5	chr1	ENSG00000143297	3'UTR	c.*1731C>A
1119	FER1L5	chr2	ENSG00000249715	Silent	c.45A>G
1120	FER1L6	chr8	ENSG00000214814	Intron	c.1701-68A>G
1121	FES	chr15	ENSG00000182511	Missense	c.208A>G
1122	FGD3	chr9	ENSG00000127084	Missense	c.1655G>T
1123	FGD6	chr12	ENSG00000180263	3'UTR	c.*4622del
1124	FGF20	chr8	ENSG00000078579	5'UTR	c.-28dup
1125	FGF6	chr12	ENSG00000111241	Missense	c.314G>A
1126	FGF6	chr12	ENSG00000111241	Missense	c.230G>A
1127	FGF7	chr15	ENSG00000140285	Missense	c.536C>T
1128	FGFR1OP2	chr12	ENSG00000111790	3'UTR	c.*938_*939del
1129	FGFR1OP2	chr12	ENSG00000111790	3'UTR	c.*1022A>G
1130	FGFR4	chr5	ENSG00000160867	Missense	c.1151C>T
1131	FHDC1	chr4	ENSG00000137460	3'UTR	c.*1917del
1132	FHL2	chr2	ENSG00000115641	3'UTR	c.*382A>G
1133	FIGLA	chr2	ENSG00000183733	5'Flank	
1134	FIP1L1	chr4	ENSG00000145216	Splice site	c.85+2T>C
1135	FKBP14	chr7	ENSG00000106080	3'UTR	c.*3207G>A
1136	FKBP14	chr7	ENSG00000106080	Intron	c.350-1172A>G
1137	FKTN	chr9	ENSG00000106692	3'UTR	c.*3614G>T
1138	FKTN	chr9	ENSG00000106692	3'UTR	c.*4388del
1139	FLG	chr1	ENSG00000143631	Silent	c.6261A>G
1140	FLI1	chr11	ENSG00000151702	Silent	c.588G>A
1141	FLII	chr17	ENSG00000177731	Missense	c.3578A>G
1142	FLNB	chr3	ENSG00000136068	Missense	c.6887A>G
1143	FLNC	chr7	ENSG00000128591	Frame shift del	c.4275del
1144	FLNC	chr7	ENSG00000128591	Frame shift del	c.4275del
1145	FLRT2	chr14	ENSG00000185070	3'UTR	c.*2675C>T
1146	FLRT3	chr20	ENSG00000125848	3'UTR	c.*442del
1147	FLT4	chr5	ENSG00000037280	3'UTR	c.*712T>C
1148	FMNL1	chr17	ENSG00000184922	Intron	c.64-6067del
1149	FMNL1	chr17	ENSG00000184922	Frame shift del	c.1760del
1150	FMNL3	chr12	ENSG00000161791	3'UTR	c.*6197A>C
1151	FMNL3	chr12	ENSG00000161791	Missense	c.3008A>G
1152	FMO5	chr1	ENSG00000131781	Intron	c.1257-24T>C
1153	FMOD	chr1	ENSG00000122176	Missense	c.800C>T
1154	FNBP1	chr9	ENSG00000187239	Intron	c.408+50_408+51delinsTC
1155	FNDC3B	chr3	ENSG00000075420	3'UTR	c.*1849T>C
1156	FNDC5	chr1	ENSG00000160097	3'UTR	c.*442_*444del
1157	FNIP1	chr5	ENSG00000217128	3'UTR	c.*739del
1158	FOXA1	chr14	ENSG00000129514	Missense	c.1145A>C
1159	FOXA2	chr20	ENSG00000125798	3'UTR	c.*389del
1160	FOXA2	chr20	ENSG00000125798	3'UTR	c.*388A>T
1161	FOXD2	chr1	ENSG00000186564	5'UTR	c.-1557C>T
1162	FOXD2	chr1	ENSG00000186564	Missense	c.416C>A
1163	FOXJ2	chr12	ENSG00000065970	3'UTR	c.*2650del
1164	FOXL2NB	chr3	ENSG00000206262	3'UTR	c.*2421del

Number	Gene name	Chromosome	Gene_ID	Mutation	Nucleotide substitution
1165	FOXN2	chr2	ENSG00000170802	3'UTR	c.*1786del
1166	FOXP4	chr6	ENSG00000137166	Missense	c.1925C>T
1167	FOXRED2	chr22	ENSG00000100350	Missense	c.1172A>G
1168	FPGT	chr1	ENSG00000254685	3'UTR	c.*565G>A
1169	FRAT2	chr10	ENSG00000181274	3'UTR	c.*304T>G
1170	FRMD1	chr6	ENSG00000153303	Missense	c.578A>G
1171	FRMPD1	chr9	ENSG00000070601	Missense	c.3851G>A
1172	FRMPD3	chrX	ENSG00000147234	Missense	c.3454G>A
1173	FRYL	chr4	ENSG00000075539	Intron	c.121-17_121-16del
1174	FSD1	chr19	ENSG00000105255	Intron	c.490+18T>C
1175	FSD1L	chr9	ENSG00000106701	Intron	c.369-7981G>A
1176	FSD2	chr15	ENSG00000186628	Missense	c.1822T>C
1177	FSIP2	chr2	ENSG00000188738	Frame shift del	c.8038del
1178	FTCDNL1	chr2	ENSG00000226124	Frame shift del	c.441_442del
1179	FUS	chr16	ENSG00000089280	5'UTR	c.-43T>C
1180	FUT2	chr19	ENSG00000176920	3'UTR	c.*169C>T
1181	FXR1	chr3	ENSG00000114416	Intron	c.258+29T>G
1182	FZD2	chr17	ENSG00000180340	Missense	c.265C>T
1183	FZD3	chr8	ENSG00000104290	Silent	c.396T>C
1184	FZD3	chr8	ENSG00000104290	3'UTR	c.*8310T>C
1185	FZD4	chr11	ENSG00000174804	3'UTR	c.*3904dup
1186	FZD6	chr8	ENSG00000164930	Frame shift del	c.125del
1187	FZD6	chr8	ENSG00000164930	Frame shift del	c.125del
1188	G6PC	chr17	ENSG00000131482	Intron	c.447-94T>C
1189	GAA	chr17	ENSG00000171298	Missense	c.2468T>C
1190	GAB1	chr4	ENSG00000109458	Missense	c.1929A>T
1191	GABARAP	chr17	ENSG00000170296	In_Frame_Del	c.21_23del
1192	GABARAP	chr17	ENSG00000170296	In_Frame_Del	c.21_23del
1193	GABBR2	chr9	ENSG00000136928	3'UTR	c.*2046del
1194	GABBR2	chr9	ENSG00000136928	3'UTR	c.*1845del
1195	GABPA	chr21	ENSG00000154727	Missense	c.628A>G
1196	GABPB2	chr1	ENSG00000143458	Splice site	c.623-2A>G
1197	GABPB2	chr1	ENSG00000143458	Intron	c.808+3978T>C
1198	GABPB2	chr1	ENSG00000143458	3'UTR	c.*3701T>C
1199	GABPB2	chr1	ENSG00000143458	3'UTR	c.*7077A>G
1200	GABRA4	chr4	ENSG00000109158	3'UTR	c.*7984A>G
1201	GABRA4	chr4	ENSG00000109158	3'UTR	c.*2916A>G
1202	GABRB2	chr5	ENSG00000145864	3'UTR	c.*5310del
1203	GAK	chr4	ENSG00000178950	Intron	c.1205+36A>G
1204	GAL3ST3	chr11	ENSG00000175229	3'UTR	c.*134A>G
1205	GAL3ST4	chr7	ENSG00000197093	Missense	c.874G>T
1206	GALC	chr14	ENSG00000054983	Intron	c.329-477del
1207	GALNT10	chr5	ENSG00000164574	3'UTR	c.*1347_*1348del
1208	GALNT11	chr7	ENSG00000178234	3'UTR	c.*497del
1209	GALNT14	chr2	ENSG00000158089	Intron	c.946+10T>C
1210	GALNT7	chr4	ENSG00000109586	3'UTR	c.*1021A>G
1211	GALNTL6	chr4	ENSG00000174473	3'UTR	c.*651del
1212	GAN	chr16	ENSG00000261609	3'UTR	c.*1443del
1213	GAPT	chr5	ENSG00000175857	3'UTR	c.*21del
1214	GAREM1	chr18	ENSG00000141441	3'UTR	c.*1743del
1215	GAREM1	chr18	ENSG00000141441	Missense	c.2273G>T
1216	GAS7	chr17	ENSG00000007237	Intron	c.552-19G>T
1217	GASK1B	chr4	ENSG00000164125	3'UTR	c.*1996_*1998del

Number	Gene name	Chromosome	Gene_ID	Mutation	Nucleotide substitution
1218	GATA1	chrX	ENSG00000102145	Intron	c.-19-40del
1219	GATA3	chr10	ENSG00000107485	Silent	c.276A>T
1220	GATA4	chr8	ENSG00000136574	Intron	c.997+30G>T
1221	GATA5	chr20	ENSG00000130700	Missense	c.916T>C
1222	GCM1	chr6	ENSG00000137270	Frame shift del	c.88del
1223	GCM1	chr6	ENSG00000137270	Frame shift del	c.88del
1224	GCNA	chrX	ENSG00000147174	Intron	c.140+42_140+43del
1225	GCNA	chrX	ENSG00000147174	Intron	c.221+43_221+44del
1226	GCNT1	chr9	ENSG00000187210	Missense	c.508G>A
1227	GCNT2	chr6	ENSG00000111846	3'UTR	c.*2057A>G
1228	GCSAM	chr3	ENSG00000174500	3'UTR	c.*2555A>G
1229	GCSAM	chr3	ENSG00000174500	3'UTR	c.*1229dup
1230	GCSAML	chr1	ENSG00000169224	3'UTR	c.*264del
1231	GDE1	chr16	ENSG00000006007	3'UTR	c.*666del
1232	GDNF	chr5	ENSG00000168621	3'UTR	c.*540del
1233	GDPD2	chrX	ENSG00000130055	Intron	c.105+52T>G
1234	GDPD3	chr16	ENSG00000102886	Intron	c.820-39A>G
1235	GDPD4	chr11	ENSG00000178795	Splice region	c.54-4del
1236	GET4	chr7	ENSG00000239857	Missense	c.400A>G
1237	GFM1	chr3	ENSG00000168827	Silent	c.2073T>C
1238	GFPT2	chr5	ENSG00000131459	3'UTR	c.*593C>A
1239	GGA2	chr16	ENSG00000103365	Intron	c.1731+56A>G
1240	GIGYF1	chr7	ENSG00000146830	Frame shift del	c.2440del
1241	GIMAP1	chr7	ENSG00000213203	3'UTR	c.*101A>G
1242	GIMAP5	chr7	ENSG00000196329	3'UTR	c.*782G>T
1243	GINS1	chr20	ENSG00000101003	3'UTR	c.*1031del
1244	GINS4	chr8	ENSG00000147536	3'UTR	
1245	GIPC2	chr1	ENSG00000137960	3'UTR	c.*502del
1246	GIT2	chr12	ENSG00000139436	Intron	c.405+53T>C
1247	GJA4	chr1	ENSG00000187513	Missense	c.175C>T
1248	GJA5	chr1	ENSG00000265107	3'UTR	c.*1751A>G
1249	GJB5	chr1	ENSG00000189280	3'Flank	
1250	GK5	chr3	ENSG00000175066	Intron	c.816+126del
1251	GLDN	chr15	ENSG00000186417	Missense	c.223G>A
1252	GLI2	chr2	ENSG00000074047	Missense	c.2740C>T
1253	GLIPR2	chr9	ENSG00000122694	3'UTR	c.*306T>G
1254	GLMP	chr1	ENSG00000198715	Missense	c.724G>A
1255	GLS	chr2	ENSG00000115419	3'UTR	c.*437T>C
1256	GLS	chr2	ENSG00000115419	3'UTR	c.*1930T>C
1257	GLT1D1	chr12	ENSG00000151948	Intron	c.375+47G>A
1258	GLT8D2	chr12	ENSG00000120820	Intron	c.-163-10600A>G
1259	GNAI2	chr3	ENSG00000114353	Missense	c.710C>A
1260	GNAL	chr18	ENSG00000141404	Silent	c.273G>A
1261	GNAQ	chr9	ENSG00000156052	5'UTR	c.-7T>C
1262	GNAS	chr20	ENSG00000087460	3'UTR	c.*38G>A
1263	GOLPH3	chr5	ENSG00000113384	3'UTR	c.*1362del
1264	GOSR1	chr17	ENSG00000108587	Intron	c.546-11del
1265	GP2	chr16	ENSG00000169347	3'UTR	c.*783G>A
1266	GPAT2	chr2	ENSG00000186281	Intron	c.1819-31C>A
1267	GPATCH2	chr1	ENSG00000092978	3'UTR	c.*3785del
1268	GPC4	chrX	ENSG00000076716	3'UTR	c.*879del
1269	GPC6	chr13	ENSG00000183098	Missense	c.913C>T
1270	GPER1	chr7	ENSG00000164850	Missense	c.1112C>A

Number	Gene name	Chromosome	Gene_ID	Mutation	Nucleotide substitution
1271	GPM6A	chr4	ENSG00000150625	3'UTR	c.*604del
1272	GPNMB	chr7	ENSG00000136235	5'Flank	
1273	GPNMB	chr7	ENSG00000136235	Intron	c.1523+18A>G
1274	GPNMB	chr7	ENSG00000136235	3'UTR	c.*154A>G
1275	GPR137C	chr14	ENSG00000180998	Intron	c.1113-8del
1276	GPR171	chr3	ENSG00000174946	3'UTR	c.*85_*88del
1277	GPR25	chr1	ENSG00000170128	Silent	c.477G>A
1278	GPR25	chr1	ENSG00000170128	3'UTR	c.*13G>C
1279	GPR34	chrX	ENSG00000171659	Missense	c.797G>A
1280	GPRC5B	chr16	ENSG00000167191	3'UTR	c.*2296G>T
1281	GPS1	chr17	ENSG00000169727	Intron	c.622-6del
1282	GPSM1	chr9	ENSG00000160360	Silent	c.1416G>T
1283	GPSM2	chr1	ENSG00000121957	3'UTR	c.*476del
1284	GPT2	chr16	ENSG00000166123	Silent	c.975C>T
1285	GRAMD1B	chr11	ENSG00000023171	Missense	c.1493T>C
1286	GRAMD2B	chr5	ENSG00000155324	3'UTR	c.*530del
1287	GREM2	chr1	ENSG00000180875	Missense	c.268C>T
1288	GRHPR	chr9	ENSG00000137106	Intron	c.866-18T>C
1289	GRIA2	chr4	ENSG00000120251	Intron	c.2292-304del
1290	GRIN2A	chr16	ENSG00000183454	3'UTR	c.*3637A>G
1291	GRIN2A	chr16	ENSG00000183454	3'UTR	c.*1432del
1292	GRIPAP1	chrX	ENSG00000068400	Intron	c.2061+599del
1293	GRM5	chr11	ENSG00000168959	Missense	c.3359C>T
1294	GRTP1	chr13	ENSG00000139835	Missense	c.422T>C
1295	GSAP	chr7	ENSG00000186088	3'UTR	c.*432del
1296	GSDME	chr7	ENSG00000105928	Intron	c.990+854del
1297	GSE1	chr16	ENSG00000131149	3'UTR	c.*2895A>G
1298	GSTK1	chr7	ENSG00000197448	Missense	c.127C>A
1299	GTF2E1	chr3	ENSG00000153767	3'UTR	c.*191T>G
1300	GTF3C1	chr16	ENSG00000077235	Missense	c.598A>G
1301	GTF3C6	chr6	ENSG00000155115	Missense	c.314T>C
1302	GTPBP1	chr22	ENSG00000100226	Intron	c.1918-30T>C
1303	GTPBP10	chr7	ENSG00000105793	3'UTR	c.*1336A>G
1304	GTPBP10	chr7	ENSG00000105793	3'UTR	c.*4072_*4074del
1305	GTPBP2	chr6	ENSG00000172432	Missense	c.713T>C
1306	GUCY1A2	chr11	ENSG00000152402	3'UTR	c.*2392G>T
1307	GUCY2D	chr17	ENSG00000132518	Frame shift Ins	c.571dup
1308	GULP1	chr2	ENSG00000144366	3'UTR	c.*697_*698del
1309	GVINP1	chr11	ENSG00000254838	RNA	n.2723A>T
1310	GXYLT1	chr12	ENSG00000151233	3'UTR	c.*3807del
1311	H1FNT	chr12	ENSG00000187166	Missense	c.527C>T
1312	H2AFJ	chr12	ENSG00000246705	3'Flank	
1313	HAAO	chr2	ENSG00000162882	Intron	c.484+106G>A
1314	HAPLN1	chr5	ENSG00000145681	3'UTR	c.*2702A>G
1315	HARS2	chr5	ENSG00000112855	Silent	c.1221T>C
1316	HAUS6	chr9	ENSG00000147874	3'UTR	c.*832del
1317	HAUS7	chrX	ENSG00000213397	5'UTR	c.-167del
1318	HAVCR2	chr5	ENSG00000135077	3'UTR	c.*1255del
1319	HBS1L	chr6	ENSG00000112339	3'UTR	c.*1091del
1320	HCFC1	chrX	ENSG00000172534	Missense	c.2435C>T
1321	HCFC1	chrX	ENSG00000172534	5'UTR	c.-461T>C
1322	HCN1	chr5	ENSG00000164588	3'UTR	c.*1064A>G
1323	HCN2	chr19	ENSG00000099822	Missense	c.965C>T

Number	Gene name	Chromosome	Gene_ID	Mutation	Nucleotide substitution
1324	HDAC9	chr7	ENSG00000048052	Missense	c.2536T>C
1325	HDGF	chr1	ENSG00000143321	3'UTR	c.*885del
1326	HDLBP	chr2	ENSG00000115677	Splice region	c.1731+8T>C
1327	HDLBP	chr2	ENSG00000115677	Frame shift Ins	c.1456dup
1328	HEATR1	chr1	ENSG00000119285	Intron	c.3583+48C>T
1329	HECA	chr6	ENSG00000112406	Silent	c.1620C>T
1330	HECTD2	chr10	ENSG00000165338	Missense	c.2164G>A
1331	HECTD3	chr1	ENSG00000126107	Silent	c.1188C>T
1332	HECTD4	chr12	ENSG00000173064	Intron	c.13114+249A>G
1333	HEG1	chr3	ENSG00000173706	3'UTR	c.*2434A>G
1334	HELZ2	chr20	ENSG00000130589	Missense	c.1654T>C
1335	HERC1	chr15	ENSG00000103657	Missense	c.512C>T
1336	HERC4	chr10	ENSG00000148634	5'UTR	c.-378C>T
1337	HES3	chr1	ENSG00000173673	Silent	c.30T>C
1338	HEXA	chr15	ENSG00000213614	3'UTR	c.*220del
1339	HEY2	chr6	ENSG00000135547	3'UTR	c.*1403T>G
1340	HGS	chr17	ENSG00000185359	Intron	c.1566+25C>T
1341	HIC2	chr22	ENSG00000169635	3'UTR	c.*4346del
1342	HID1	chr17	ENSG00000167861	3'UTR	c.*96T>C
1343	HIF1A	chr14	ENSG00000100644	5'UTR	c.-9del
1344	HIF1A	chr14	ENSG00000100644	Intron	c.2094-41del
1345	HINFP	chr11	ENSG00000172273	5'UTR	c.-100T>C
1346	HINT2	chr9	ENSG00000137133	Intron	c.327+44A>T
1347	HIP1R	chr12	ENSG00000130787	Missense	c.2998C>T
1348	HIPK2	chr7	ENSG00000064393	3'UTR	c.*10083A>G
1349	HIPK2	chr7	ENSG00000064393	Silent	c.3348C>T
1350	HIVEP2	chr6	ENSG00000010818	Missense	c.7019C>T
1351	HJURP	chr2	ENSG00000123485	Intron	c.185-92del
1352	HLX	chr1	ENSG00000136630	5'Flank	
1353	HMCN1	chr1	ENSG00000143341	Silent	c.4752C>T
1354	HMCN1	chr1	ENSG00000143341	3'UTR	c.*464del
1355	HMGA2	chr12	ENSG00000149948	Intron	c.112-14T>C
1356	HMGA2	chr12	ENSG00000149948	3'UTR	c.*6059del
1357	HMGB1	chr13	ENSG00000189403	3'UTR	c.*2004del
1358	HMGCLL1	chr6	ENSG00000146151	3'UTR	c.*1843del
1359	HMGCR	chr5	ENSG00000113161	3'UTR	c.*984del
1360	HMHB1	chr5	ENSG00000158497	Missense	c.56A>C
1361	HNRNPA1L2	chr13	ENSG00000139675	3'UTR	
1362	HNRNPA2B1	chr7	ENSG00000122566	Frame shift del	c.986del
1363	HNRNPA2B1	chr7	ENSG00000122566	Frame shift del	c.986del
1364	HNRNPK	chr9	ENSG00000165119	3'UTR	c.*464del
1365	HOXA1	chr7	ENSG00000105991	Missense	c.368A>G
1366	HOXA1	chr7	ENSG00000105991	Silent	c.234T>C
1367	HOXA4	chr7	ENSG00000197576	Silent	c.519C>T
1368	HOXA9	chr7	ENSG00000078399	Missense	c.32A>G
1369	HOXB13	chr17	ENSG00000159184	3'UTR	c.*1520G>C
1370	HOXB3	chr17	ENSG00000120093	Missense	c.146G>A
1371	HOXB4	chr17	ENSG00000182742	Silent	c.384C>T
1372	HOXC4	chr12	ENSG00000198353	Intron	c.-124+15392_-124+15394del
1373	HPS1	chr10	ENSG00000107521	Silent	c.1701G>A
1374	HPSE2	chr10	ENSG00000172987	3'UTR	c.*293A>G
1375	HRCT1	chr9	ENSG00000196196	Missense	c.266A>T
1376	HRH3	chr20	ENSG00000101180	Missense	c.224A>G

Number	Gene name	Chromosome	Gene_ID	Mutation	Nucleotide substitution
1377	HSD17B4	chr5	ENSG00000133835	Intron	c.1842+14T>C
1378	HSF2	chr6	ENSG00000025156	Intron	c.1071-16del
1379	HSPA12B	chr20	ENSG00000132622	Intron	c.1302-15T>C
1380	HSPA8	chr11	ENSG00000109971	5'UTR	c.-286A>G
1381	HSPG2	chr1	ENSG00000142798	Missense	c.7036G>A
1382	HSPH1	chr13	ENSG00000120694	Intron	c.307-14del
1383	HTATSF1	chrX	ENSG00000102241	Intron	c.925-18del
1384	HTR1F	chr3	ENSG00000179097	Frame shift del	c.930del
1385	HTR1F	chr3	ENSG00000179097	Frame shift del	c.930del
1386	HTRA3	chr4	ENSG00000170801	3'UTR	c.*350T>C
1387	HTT	chr4	ENSG00000197386	Intron	c.8786-29A>T
1388	HUWE1	chrX	ENSG00000086758	3'UTR	c.*559del
1389	HUWE1	chrX	ENSG00000086758	Missense	c.12191A>G
1390	IAH1	chr2	ENSG00000134330	5'UTR	c.-238G>C
1391	ICA1L	chr2	ENSG00000163596	Intron	c.685-49del
1392	ICE1	chr5	ENSG00000164151	3'UTR	c.*291del
1393	ICE2	chr15	ENSG00000128915	Intron	c.409-10T>A
1394	ICK	chr6	ENSG00000112144	3'UTR	c.*168del
1395	ICMT	chr1	ENSG00000116237	3'UTR	c.*3034A>G
1396	IDH2	chr15	ENSG00000182054	Missense	c.625G>A
1397	IFFO2	chr1	ENSG00000169991	3'UTR	c.*3799del
1398	IFNA2	chr9	ENSG00000188379	3'UTR	c.*494A>G
1399	IFNB1	chr9	ENSG00000171855	3'UTR	c.*91A>G
1400	IFRD2	chr3	ENSG00000214706	Missense	c.535C>T
1401	IFT140	chr16	ENSG00000187535	Missense	c.191A>G
1402	IGF1	chr12	ENSG00000017427	3'UTR	c.*5718del
1403	IGF2	chr11	ENSG00000167244	3'UTR	c.*2409del
1404	IGF2BP1	chr17	ENSG00000159217	3'UTR	c.*3063A>G
1405	IGF2R	chr6	ENSG00000197081	Splice site	c.646+2T>C
1406	IGFBP5	chr2	ENSG00000115461	3'UTR	c.*1727dup
1407	IGFBP5	chr2	ENSG00000115461	5'UTR	c.-697del
1408	IGHMBP2	chr11	ENSG00000132740	Frame shift del	c.1165del
1409	IGHMBP2	chr11	ENSG00000132740	Frame shift del	c.1165del
1410	IGKV1-27	chr2	ENSG00000244575	Frame shift Ins	c.335dup
1411	IGLL5	chr22	ENSG00000254709	3'Flank	
1412	IGSF11	chr3	ENSG00000144847	Missense	c.619C>A
1413	IK	chr5	ENSG00000113141	Frame shift del	c.981del
1414	IK	chr5	ENSG00000113141	Frame shift del	c.981del
1415	IKZF1	chr7	ENSG00000185811	Missense	c.542C>T
1416	IKZF2	chr2	ENSG00000030419	Silent	c.519G>A
1417	IKZF3	chr17	ENSG00000161405	3'UTR	c.*4471del
1418	IL16	chr15	ENSG00000172349	Intron	c.3149+24C>G
1419	IL16	chr15	ENSG00000172349	Silent	c.3522G>T
1420	IL17RD	chr3	ENSG00000144730	3'UTR	c.*6122del
1421	IL17RD	chr3	ENSG00000144730	3'UTR	c.*3572G>A
1422	IL17RD	chr3	ENSG00000144730	Missense	c.164A>G
1423	IL17RE	chr3	ENSG00000163701	Intron	c.1073-56T>C
1424	IL18R1	chr2	ENSG00000115604	Frame shift del	c.601del
1425	IL1F10	chr2	ENSG00000136697	3'UTR	c.*105A>G
1426	IL1RAP	chr3	ENSG00000196083	3'UTR	c.*1692T>C
1427	IL1RL1	chr2	ENSG00000115602	3'UTR	c.*1315del
1428	IL1RL2	chr2	ENSG00000115598	Frame shift del	c.685del
1429	IL1RL2	chr2	ENSG00000115598	Frame shift del	c.685del

Number	Gene name	Chromosome	Gene_ID	Mutation	Nucleotide substitution
1430	IL1RN	chr2	ENSG00000136689	3'UTR	c.*490del
1431	IL36RN	chr2	ENSG00000136695	Missense	c.304C>T
1432	IL6ST	chr5	ENSG00000134352	3'UTR	c.*5981del
1433	IL6ST	chr5	ENSG00000134352	3'UTR	c.*5842del
1434	ILRUN	chr6	ENSG00000196821	3'UTR	c.*324del
1435	IMMT	chr2	ENSG00000132305	Missense	c.28G>A
1436	IMPDH1	chr7	ENSG00000106348	Intron	c.353+57G>A
1437	ING3	chr7	ENSG00000071243	3'UTR	c.*809G>A
1438	ING3	chr7	ENSG00000071243	Missense	c.377T>C
1439	INO80	chr15	ENSG00000128908	3'UTR	c.*318A>G
1440	INO80	chr15	ENSG00000128908	Frame shift del	c.685del
1441	INO80	chr15	ENSG00000128908	Frame shift del	c.685del
1442	INPP5B	chr1	ENSG00000204084	3'UTR	c.*274del
1443	INPP5K	chr17	ENSG00000132376	Missense	c.829T>C
1444	INPPL1	chr11	ENSG00000165458	Frame shift del	c.3466del
1445	INPPL1	chr11	ENSG00000165458	Frame shift del	c.3466del
1446	INSC	chr11	ENSG00000188487	3'UTR	c.*1079T>C
1447	INSM2	chr14	ENSG00000168348	Frame shift del	c.165del
1448	INSM2	chr14	ENSG00000168348	Frame shift del	c.165del
1449	INSYN2A	chr10	ENSG00000188916	3'UTR	c.*387del
1450	INTS1	chr7	ENSG00000164880	Missense	c.6448C>A
1451	INTS10	chr8	ENSG00000104613	Intron	c.1640-1076T>C
1452	INVS	chr9	ENSG00000119509	3'UTR	c.*994T>C
1453	IPO9	chr1	ENSG00000198700	3'UTR	c.*3414del
1454	IPO9	chr1	ENSG00000198700	3'UTR	c.*4609G>T
1455	IQCE	chr7	ENSG00000106012	3'UTR	c.*1685del
1456	IQCE	chr7	ENSG00000106012	3'UTR	c.*3828A>G
1457	IQCG	chr3	ENSG00000114473	Missense	c.111A>G
1458	IQSEC1	chr3	ENSG00000144711	Missense	c.2789C>T
1459	IQSEC1	chr3	ENSG00000144711	Silent	c.1407C>T
1460	IRAK3	chr12	ENSG00000090376	3'UTR	c.*895del
1461	IREB2	chr15	ENSG00000136381	Intron	c.1952-32del
1462	IRF1	chr5	ENSG00000125347	Missense	c.134G>A
1463	IRS2	chr13	ENSG00000185950	Silent	c.4002C>A
1464	IRX2	chr5	ENSG00000170561	Missense	c.416A>G
1465	ISCA1	chr9	ENSG00000135070	Intron	c.-213-29T>C
1466	ISL2	chr15	ENSG00000159556	Silent	c.282G>A
1467	ISLR2	chr15	ENSG00000167178	Intron	c.-187+800del
1468	ISM1	chr20	ENSG00000101230	3'UTR	c.*984A>G
1469	ITCH	chr20	ENSG00000078747	3'UTR	c.*1256T>C
1470	ITGA11	chr15	ENSG00000137809	Intron	c.2212-26C>T
1471	ITGA2	chr5	ENSG00000164171	Intron	c.1173+97G>T
1472	ITGA2	chr5	ENSG00000164171	3'UTR	c.*1906G>A
1473	ITGA5	chr12	ENSG00000161638	Silent	c.2331C>T
1474	ITGA5	chr12	ENSG00000161638	5'Flank	
1475	ITGA9	chr3	ENSG00000144668	Missense	c.1537T>C
1476	ITGA9	chr3	ENSG00000144668	3'UTR	c.*1377A>G
1477	ITGAV	chr2	ENSG00000138448	Missense	c.175A>G
1478	ITGB1BP2	chrX	ENSG00000147166	Missense	c.863T>C
1479	ITGB2	chr21	ENSG00000160255	Silent	c.2196C>T
1480	ITGB2	chr21	ENSG00000160255	5'Flank	
1481	ITGB4	chr17	ENSG00000132470	Intron	c.1990+38C>A
1482	ITGB6	chr2	ENSG00000115221	Missense	c.1682G>C

Number	Gene name	Chromosome	Gene_ID	Mutation	Nucleotide substitution
1483	ITIH5	chr10	ENSG00000123243	Intron	c.2032+16C>T
1484	ITM2C	chr2	ENSG00000135916	Missense	c.283C>T
1485	ITPR3	chr6	ENSG00000096433	Intron	c.4350+15G>T
1486	ITSN1	chr21	ENSG00000205726	Nonsense	c.3712C>T
1487	IVNS1ABP	chr1	ENSG00000116679	3'Flank	
1488	JADE1	chr4	ENSG00000077684	3'UTR	c.*1747del
1489	JAKMIP1	chr4	ENSG00000152969	Frame shift del	c.2462del
1490	JAKMIP1	chr4	ENSG00000152969	Frame shift del	c.2462del
1491	JAM3	chr11	ENSG00000166086	3'UTR	c.*1471del
1492	JPH2	chr20	ENSG00000149596	Missense	c.164C>A
1493	JPT2	chr16	ENSG00000206053	3'UTR	c.*2601del
1494	KAAG1	chr6	ENSG00000146049	5'UTR	c.-342C>A
1495	KALRN	chr3	ENSG00000160145	Intron	c.2341-41G>A
1496	KANK1	chr9	ENSG00000107104	3'UTR	c.*184A>G
1497	KANK2	chr19	ENSG00000197256	Silent	c.1836A>G
1498	KANSL1L	chr2	ENSG00000144445	3'UTR	c.*469_*470del
1499	KANSL3	chr2	ENSG00000114982	3'UTR	c.*1520G>T
1500	KAT6B	chr10	ENSG00000156650	3'UTR	c.*5A>G
1501	KAT6B	chr10	ENSG00000156650	Frame shift Ins	c.862dup
1502	KAT6B	chr10	ENSG00000156650	Splice region	c.1062-3T>C
1503	KATNBL1	chr15	ENSG00000134152	Missense	c.631A>G
1504	KAZN	chr1	ENSG00000189337	5'UTR	c.-213del
1505	KAZN	chr1	ENSG00000189337	Silent	c.1911C>T
1506	KCNAB1	chr3	ENSG00000169282	Missense	c.226A>T
1507	KCNB1	chr20	ENSG00000158445	Intron	c.97-35122_97-35121del
1508	KCNC3	chr19	ENSG00000131398	anslation_Start_S	c.1A>G
1509	KCNK3	chr2	ENSG00000171303	3'UTR	c.*1834C>T
1510	KCNK4	chr11	ENSG00000182450	3'UTR	c.*152T>A
1511	KCNK5	chr6	ENSG00000164626	3'UTR	c.*324G>A
1512	KCNQ5	chr6	ENSG00000185760	Splice region	c.921T>A
1513	KCNQ5	chr6	ENSG00000185760	3'UTR	c.*2207_*2225del
1514	KCNT2	chr1	ENSG00000162687	3'UTR	c.*1780T>C
1515	KCNT2	chr1	ENSG00000162687	Intron	c.95+25810C>T
1516	KCTD10	chr12	ENSG00000110906	5'UTR	c.-508del
1517	KCTD5	chr16	ENSG00000167977	5'Flank	
1518	KCTD6	chr3	ENSG00000168301	Missense	c.146C>T
1519	KCTD9	chr8	ENSG00000104756	3'UTR	c.*1101del
1520	KDM1A	chr1	ENSG00000004487	3'UTR	c.*283del
1521	KDM3B	chr5	ENSG00000120733	Intron	c.5205+20A>G
1522	KDM4A	chr1	ENSG00000066135	Intron	c.429+59del
1523	KDM4C	chr9	ENSG00000107077	Missense	c.2074A>T
1524	KDM5A	chr12	ENSG00000073614	Intron	c.1149+36T>C
1525	KDM6B	chr17	ENSG00000132510	Missense	c.961A>G
1526	KDM7A	chr7	ENSG00000006459	3'UTR	c.*860del
1527	KDM7A	chr7	ENSG00000006459	3'UTR	c.*352del
1528	KHDRBS3	chr8	ENSG00000131773	5'UTR	c.-22T>C
1529	KHNYN	chr14	ENSG00000100441	3'UTR	c.*1911del
1530	KHSRP	chr19	ENSG00000088247	3'UTR	c.*471del
1531	KIAA0232	chr4	ENSG00000170871	Silent	c.1521G>A
1532	KIAA0408	chr6	ENSG00000189367	Missense	c.929A>G
1533	KIAA1109	chr4	ENSG00000138688	Missense	c.14779T>C
1534	KIAA1217	chr10	ENSG00000120549	Intron	c.355-40A>T
1535	KIAA1217	chr10	ENSG00000120549	Intron	c.1357+36C>T

Number	Gene name	Chromosome	Gene_ID	Mutation	Nucleotide substitution
1536	KIAA1257	chr3	ENSG00000114656	3'UTR	c.*773C>A
1537	KIAA1324	chr1	ENSG00000116299	Missense	c.436A>C
1538	KIAA1324	chr1	ENSG00000116299	Missense	c.2498C>T
1539	KIAA1614	chr1	ENSG00000135835	3'UTR	c.*5870G>A
1540	KIAA1671	chr22	ENSG00000197077	Missense	c.3505C>T
1541	KIAA2012	chr2	ENSG00000182329	Intron	c.1832-2427_1832-2425del
1542	KIAA2012	chr2	ENSG00000182329	Intron	c.1832-2435A>T
1543	KIAA2013	chr1	ENSG00000116685	3'UTR	c.*27C>A
1544	KIF13A	chr6	ENSG00000137177	3'UTR	c.*444A>G
1545	KIF13B	chr8	ENSG00000197892	Silent	c.2973C>T
1546	KIF16B	chr20	ENSG00000089177	Silent	c.3573C>T
1547	KIF17	chr1	ENSG00000117245	Silent	c.2655C>T
1548	KIF19	chr17	ENSG00000196169	Missense	c.548G>A
1549	KIF1A	chr2	ENSG00000130294	Silent	c.3459C>T
1550	KIF1B	chr1	ENSG00000054523	Intron	c.-79-4432del
1551	KIF1B	chr1	ENSG00000054523	3'UTR	c.*557del
1552	KIF1C	chr17	ENSG00000129250	Intron	c.1666+17T>C
1553	KIF26A	chr14	ENSG00000066735	Missense	c.4405G>A
1554	KIF2A	chr5	ENSG00000068796	Intron	c.5-27del
1555	KIF3B	chr20	ENSG00000101350	3'UTR	c.*2708A>G
1556	KIF4A	chrX	ENSG00000090889	Intron	c.3072+16C>T
1557	KIF4B	chr5	ENSG00000226650	3'UTR	
1558	KIF6	chr6	ENSG00000164627	3'UTR	c.*1932del
1559	KIFC2	chr8	ENSG00000167702	Missense	c.1834T>A
1560	KIT	chr4	ENSG00000157404	Missense	c.1594G>A
1561	KIT	chr4	ENSG00000157404	3'UTR	c.*820T>C
1562	KLF2	chr19	ENSG00000127528	Frame shift del	c.503del
1563	KLF9	chr9	ENSG00000119138	Missense	c.245G>A
1564	KLHDC2	chr14	ENSG00000165516	Missense	c.662A>G
1565	KLHL17	chr1	ENSG00000187961	5'UTR	c.-229T>C
1566	KLHL18	chr3	ENSG00000114648	Missense	c.578T>C
1567	KLHL21	chr1	ENSG00000162413	3'UTR	c.*282T>G
1568	KLHL29	chr2	ENSG00000119771	Intron	c.-45-38264T>A
1569	KLHL35	chr11	ENSG00000149243	Silent	c.297C>T
1570	KLHL4	chrX	ENSG00000102271	Missense	c.1288G>A
1571	KLHL8	chr4	ENSG00000145332	3'UTR	c.*2972A>C
1572	KMT2A	chr11	ENSG00000118058	Missense	c.473G>A
1573	KMT2B	chr19	ENSG00000272333	Nonsense	c.3214C>T
1574	KMT2D	chr12	ENSG00000167548	In_Frame_Del	c.11217_11222del
1575	KMT2D	chr12	ENSG00000167548	Missense	c.7412G>A
1576	KNDC1	chr10	ENSG00000171798	Splice region	c.4443+7A>G
1577	KNG1	chr3	ENSG00000113889	Missense	c.1510G>A
1578	KNTC1	chr12	ENSG00000184445	Intron	c.6031-951del
1579	KPNA2	chr17	ENSG00000182481	Intron	c.1498-42T>C
1580	KPNA6	chr1	ENSG00000025800	Intron	c.912-105del
1581	KRAS	chr12	ENSG00000133703	3'UTR	c.*3845del
1582	KRAS	chr12	ENSG00000133703	3'UTR	c.*2304T>C
1583	KREMEN1	chr22	ENSG00000183762	3'UTR	c.*1642_*1643del
1584	KREMEN1	chr22	ENSG00000183762	3'UTR	c.*713C>T
1585	KRI1	chr19	ENSG00000129347	Missense	c.993_994delinsTT
1586	KRI1	chr19	ENSG00000129347	Missense	c.993_994delinsTT
1587	KRT10	chr17	ENSG00000186395	Frame shift del	c.153del
1588	KRT10	chr17	ENSG00000186395	Frame shift del	c.153del

Number	Gene name	Chromosome	Gene_ID	Mutation	Nucleotide substitution
1589	KRT24	chr17	ENSG00000167916	Missense	c.956C>T
1590	KRT35	chr17	ENSG00000197079	Missense	c.1223G>A
1591	KRT83	chr12	ENSG00000170523	3'UTR	c.*213A>G
1592	KRT86	chr12	ENSG00000170442	Intron	c.-5+6045G>A
1593	KRTAP26-1	chr21	ENSG00000197683	Missense	c.530A>G
1594	KSR1	chr17	ENSG00000141068	Missense	c.140T>G
1595	L1CAM	chrX	ENSG00000198910	3'UTR	c.*992del
1596	L3HYPDH	chr14	ENSG00000126790	Missense	c.215A>G
1597	LAMA3	chr18	ENSG00000053747	Intron	c.786+17C>T
1598	LAMA4	chr6	ENSG00000112769	Missense	c.1507G>A
1599	LAMA5	chr20	ENSG00000130702	Intron	c.1477+3515del
1600	LAMB1	chr7	ENSG00000091136	Missense	c.3605A>G
1601	LAMB2	chr3	ENSG00000172037	Splice region	c.1226-5G>A
1602	LAMC3	chr9	ENSG00000050555	5'Flank	
1603	LAMP2	chrX	ENSG00000005893	3'UTR	c.*1738_*1741del
1604	LAPTM4A	chr2	ENSG00000068697	Intron	c.433-33T>C
1605	LAPTM4B	chr8	ENSG00000104341	3'UTR	c.*1475del
1606	LAPTM4B	chr8	ENSG00000104341	3'UTR	c.*1471A>G
1607	LARP1	chr5	ENSG00000155506	3'UTR	c.*2934_*2937del
1608	LARP1	chr5	ENSG00000155506	3'UTR	c.*2936_*2937del
1609	LARP4	chr12	ENSG00000161813	Intron	c.322+12G>C
1610	LARP4	chr12	ENSG00000161813	3'UTR	c.*1158T>A
1611	LARP6	chr15	ENSG00000166173	Silent	c.715C>T
1612	LAT2	chr7	ENSG00000086730	Intron	c.-29-35A>G
1613	LATS1	chr6	ENSG00000131023	3'UTR	c.*2265dup
1614	LATS1	chr6	ENSG00000131023	Missense	c.1459A>G
1615	LBX2	chr2	ENSG00000179528	3'UTR	c.*580del
1616	LCT	chr2	ENSG00000115850	Silent	c.3360C>A
1617	LDAH	chr2	ENSG00000118961	3'UTR	c.*906A>G
1618	LDHAL6B	chr15	ENSG00000171989	Silent	c.462G>A
1619	LDLR	chr19	ENSG00000130164	3'UTR	c.*1419_*1423del
1620	LEPROTL1	chr8	ENSG00000104660	Silent	c.387A>G
1621	LETMD1	chr12	ENSG00000050426	5'UTR	c.-138C>A
1622	LGALS8	chr1	ENSG00000116977	Intron	c.-104+307G>A
1623	LGMN	chr14	ENSG00000100600	Intron	c.1192-56A>C
1624	LGR6	chr1	ENSG00000133067	Intron	c.981-76T>C
1625	LHCGR	chr2	ENSG00000138039	Missense	c.1258T>C
1626	LHX4	chr1	ENSG00000121454	3'UTR	c.*4013C>A
1627	LHX8	chr1	ENSG00000162624	5'UTR	c.-405G>C
1628	LIMCH1	chr4	ENSG00000064042	Splice region	c.238-8C>T
1629	LIMD1	chr3	ENSG00000144791	3'UTR	c.*3966del
1630	LIMS1	chr2	ENSG00000169756	3'UTR	c.*2790_*2791del
1631	LINC00479	chr21	ENSG00000236384	Intron	n.317+42T>C
1632	LINC00583	chr9	ENSG00000205636	Intron	n.92-21T>C
1633	LINC00862	chr1	ENSG00000203721	RNA	n.1437del
1634	LINC00943	chr12	ENSG00000189238	RNA	n.933T>A
1635	LINC01151	chr8	ENSG00000253819	Intron	n.211-16del
1636	LINC01341	chr1	ENSG00000227953	RNA	n.198C>T
1637	LINC01553	chr10	ENSG00000235931	RNA	n.3955del
1638	LINC01558	chr6	ENSG00000146521	RNA	n.300C>T
1639	LINC01588	chr14	ENSG00000214900	RNA	n.457C>G
1640	LINC01674	chr21	ENSG00000228159	Intron	n.166-1130C>T
1641	LINC02272	chr4	ENSG00000251283	RNA	n.393del

Number	Gene name	Chromosome	Gene_ID	Mutation	Nucleotide substitution
1642	LINC02832	chr2	ENSG00000227308	Intron	n.119-15784_119-15783del
1643	LINGO1	chr15	ENSG00000169783	Missense	c.1417C>T
1644	LINS1	chr15	ENSG00000140471	3'UTR	c.*981T>C
1645	LIPT1	chr2	ENSG00000144182	3'UTR	
1646	LLPH	chr12	ENSG00000139233	3'UTR	c.*705del
1647	LMBRD1	chr6	ENSG00000168216	Splice region	c.865-4del
1648	LMBRD2	chr5	ENSG00000164187	Frame shift del	c.2086del
1649	LMF1	chr16	ENSG00000103227	Splice region	c.*46+6T>C
1650	LMNTD1	chr12	ENSG00000152936	Missense	c.67T>C
1651	LMO4	chr1	ENSG00000143013	Intron	c.333+27_333+28insA
1652	LMO4	chr1	ENSG00000143013	3'UTR	c.*1872del
1653	LMTK2	chr7	ENSG00000164715	3'UTR	c.*740T>C
1654	LMTK3	chr19	ENSG00000142235	Missense	c.3584C>T
1655	LONP2	chr16	ENSG00000102910	3'UTR	c.*2277T>G
1656	LONP2	chr16	ENSG00000102910	3'UTR	c.*2720del
1657	LOXL3	chr2	ENSG00000115318	Frame shift del	c.824del
1658	LOXL3	chr2	ENSG00000115318	Frame shift del	c.824del
1659	LOXL3	chr2	ENSG00000115318	Silent	c.804C>T
1660	LPAR4	chrX	ENSG00000147145	3'UTR	c.*1108_*1109del
1661	LPIN2	chr18	ENSG00000101577	Intron	c.2175-72T>G
1662	LPL	chr8	ENSG00000175445	Intron	c.1323-17T>A
1663	LPP	chr3	ENSG00000145012	3'UTR	c.*112C>T
1664	LPP	chr3	ENSG00000145012	3'UTR	c.*3203del
1665	LPP	chr3	ENSG00000145012	3'UTR	c.*4078C>T
1666	LPP	chr3	ENSG00000145012	3'UTR	c.*13433A>G
1667	LPP	chr3	ENSG00000145012	3'UTR	c.*15515del
1668	LRATD1	chr2	ENSG00000162981	3'UTR	c.*3208del
1669	LRCH1	chr13	ENSG00000136141	3'UTR	c.*1615A>G
1670	LRIG2	chr1	ENSG00000198799	3'UTR	c.*570del
1671	LRIG3	chr12	ENSG00000139263	Intron	c.236+409T>C
1672	LRP1	chr12	ENSG00000123384	Intron	c.841+3488del
1673	LRP1B	chr2	ENSG00000168702	Silent	c.4839C>T
1674	LRP1B	chr2	ENSG00000168702	Missense	c.3124T>C
1675	LRP4	chr11	ENSG00000134569	Intron	c.5155+99T>G
1676	LRP5	chr11	ENSG00000162337	3'Flank	
1677	LRP6	chr12	ENSG00000070018	3'UTR	c.*1771del
1678	LRP8	chr1	ENSG00000157193	Silent	c.624C>T
1679	LRR1	chr14	ENSG00000165501	Missense	c.34C>T
1680	LRRC28	chr15	ENSG00000168904	3'UTR	c.*1370A>G
1681	LRRC31	chr3	ENSG00000114248	3'UTR	c.*205A>G
1682	LRRC36	chr16	ENSG00000159708	Silent	c.1485A>G
1683	LRRC37A3	chr17	ENSG00000176809	Missense	c.268T>C
1684	LRRC37B	chr17	ENSG00000185158	Intron	c.2205-4275T>C
1685	LRRC45	chr17	ENSG00000169683	Frame shift del	c.1436_1442del
1686	LRRC45	chr17	ENSG00000169683	Frame shift del	c.1436_1442del
1687	LRRC47	chr1	ENSG00000130764	Silent	c.561T>C
1688	LRRC49	chr15	ENSG00000137821	Frame shift Ins	c.101dup
1689	LRRC7	chr1	ENSG00000033122	Intron	c.-76-14del
1690	LRRC7	chr1	ENSG00000033122	Missense	c.358A>G
1691	LRRC72	chr7	ENSG00000205858	Intron	c.671-3del
1692	LRRC73	chr6	ENSG00000204052	5'UTR	c.-846A>T
1693	LRRC9	chr14	ENSG00000131951	Missense	c.2416A>G
1694	LRRFIP1	chr2	ENSG00000124831	3'UTR	c.*1566del

Number	Gene name	Chromosome	Gene_ID	Mutation	Nucleotide substitution
1695	LRRFIP2	chr3	ENSG00000093167	Missense	c.2012T>C
1696	LRRK2	chr12	ENSG00000188906	3'UTR	c.*936del
1697	LRSAM1	chr9	ENSG00000148356	Silent	c.126A>C
1698	LRSAM1	chr9	ENSG00000148356	Missense	c.640C>T
1699	LSP1	chr11	ENSG00000130592	Intron	c.930+27G>A
1700	LSR	chr19	ENSG00000105699	Missense	c.472A>T
1701	LTB4R	chr14	ENSG00000213903	3'UTR	c.*1263G>T
1702	LTC4S	chr5	ENSG00000213316	Missense	c.41T>C
1703	LUC7L3	chr17	ENSG00000108848	Intron	c.206+33del
1704	LUC7L3	chr17	ENSG00000108848	Missense	c.935G>A
1705	LVRN	chr5	ENSG00000172901	Intron	c.2757-579del
1706	LY6K	chr8	ENSG00000160886	5'Flank	
1707	LY6K	chr8	ENSG00000160886	5'Flank	
1708	LY75	chr2	ENSG00000054219	Missense	c.4417A>G
1709	LY75	chr2	ENSG00000054219	Frame shift del	c.4034del
1710	LY75	chr2	ENSG00000054219	Frame shift del	c.4034del
1711	LYG2	chr2	ENSG00000185674	Missense	c.191G>A
1712	LYNX1	chr8	ENSG00000180155	3'UTR	c.*249G>A
1713	LYPLA1	chr8	ENSG00000120992	3'UTR	c.*664del
1714	LYRM2	chr6	ENSG00000083099	3'UTR	c.*2836del
1715	LYRM7	chr5	ENSG00000186687	Intron	c.92-71A>C
1716	LYST	chr1	ENSG00000143669	Silent	c.6000T>C
1717	LZIC	chr1	ENSG00000162441	Frame shift del	c.570del
1718	LZIC	chr1	ENSG00000162441	5'UTR	c.-4T>A
1719	LZTR1	chr22	ENSG00000099949	3'UTR	c.*790C>T
1720	LZTS2	chr10	ENSG00000107816	Missense	c.1709G>T
1721	MACC1	chr7	ENSG00000183742	3'UTR	c.*3530G>A
1722	MACC1	chr7	ENSG00000183742	Missense	c.1826T>C
1723	MACF1	chr1	ENSG00000127603	Missense	c.2698C>T
1724	MACF1	chr1	ENSG00000127603	3'UTR	c.*830del
1725	MAFA	chr8	ENSG00000182759	3'UTR	c.*888_*889del
1726	MAFA	chr8	ENSG00000182759	3'UTR	c.*878del
1727	MAFB	chr20	ENSG00000204103	Missense	c.166T>C
1728	MAFG	chr17	ENSG00000197063	Silent	c.60T>C
1729	MAGED1	chrX	ENSG00000179222	Missense	c.1057C>G
1730	MAGI1	chr3	ENSG00000151276	Silent	c.4209C>T
1731	MALAT1	chr11	ENSG00000251562	Splice site	n.392+2T>C
1732	MALRD1	chr10	ENSG00000204740	Intron	c.996-42del
1733	MALT1	chr18	ENSG00000172175	3'UTR	c.*2380A>G
1734	MALT1	chr18	ENSG00000172175	3'UTR	c.*5351del
1735	MAN1B1	chr9	ENSG00000177239	Intron	c.1255-250T>C
1736	MAN1C1	chr1	ENSG00000117643	3'UTR	c.*1178_*1179del
1737	MAN2A1	chr5	ENSG00000112893	5'UTR	c.-381del
1738	MAN2A2	chr15	ENSG00000196547	Missense	c.1252G>A
1739	MANBA	chr4	ENSG00000109323	Silent	c.459T>C
1740	MAP2K7	chr19	ENSG00000076984	3'UTR	c.*1349del
1741	MAP3K13	chr3	ENSG00000073803	3'UTR	c.*3404A>C
1742	MAP3K2	chr2	ENSG00000169967	3'UTR	c.*4252del
1743	MAP3K20	chr2	ENSG00000091436	3'UTR	c.*4389_*4390dup
1744	MAP3K4	chr6	ENSG00000085511	Intron	c.3416-9del
1745	MAP3K6	chr1	ENSG00000142733	Silent	c.1887G>A
1746	MAP3K9	chr14	ENSG00000006432	5'UTR	c.-91dup
1747	MAP4	chr3	ENSG00000047849	Silent	c.762A>G

Number	Gene name	Chromosome	Gene_ID	Mutation	Nucleotide substitution
1748	MAP4	chr3	ENSG00000047849	Intron	c.420+1495dup
1749	MAP4	chr3	ENSG00000047849	3'UTR	c.*692_*693del
1750	MAP4K2	chr11	ENSG00000168067	Intron	c.414+40del
1751	MAP4K3	chr2	ENSG00000011566	Splice region	c.1795-7del
1752	MAP4K3	chr2	ENSG00000011566	Splice region	c.662+4A>G
1753	MAP4K5	chr14	ENSG00000012983	3'UTR	c.*270del
1754	MAP6	chr11	ENSG00000171533	Missense	c.1423G>T
1755	MAPK13	chr6	ENSG00000156711	3'UTR	c.*60A>G
1756	MAPK6	chr15	ENSG00000069956	Missense	c.1220A>G
1757	MAPK8IP1	chr11	ENSG00000121653	3'UTR	c.*537C>T
1758	MARK2	chr11	ENSG00000072518	Intron	c.1769+17G>C
1759	MARK2	chr11	ENSG00000072518	Intron	c.1769+27G>A
1760	MAST1	chr19	ENSG00000105613	Missense	c.1106C>T
1761	MAST4	chr5	ENSG00000069020	Intron	c.3386-49del
1762	MAT2B	chr5	ENSG00000038274	Intron	c.225+502del
1763	MATN2	chr8	ENSG00000132561	3'UTR	c.*84G>A
1764	MATN3	chr2	ENSG00000132031	Frame shift del	c.1159del
1765	MATN3	chr2	ENSG00000132031	Frame shift del	c.1159del
1766	MBL2	chr10	ENSG00000165471	3'UTR	c.*358G>A
1767	MBNL3	chrX	ENSG00000076770	3'UTR	c.*1857_*1858dup
1768	MBP	chr18	ENSG00000197971	Splice site	c.870+2T>C
1769	MC4R	chr18	ENSG00000166603	Missense	c.867A>G
1770	MCAT	chr22	ENSG00000100294	Missense	c.845C>T
1771	MCC	chr5	ENSG00000171444	5'UTR	c.-243T>C
1772	MCCC2	chr5	ENSG00000131844	Silent	c.711T>C
1773	MCCC2	chr5	ENSG00000131844	3'UTR	c.*1167A>G
1774	MCFD2	chr2	ENSG00000180398	3'UTR	c.*94C>G
1775	MCHR1	chr22	ENSG00000128285	3'UTR	c.*822T>G
1776	MCM3AP	chr21	ENSG00000160294	Missense	c.416T>G
1777	MCM6	chr2	ENSG00000076003	3'UTR	c.*309A>G
1778	MCM7	chr7	ENSG00000166508	Silent	c.1407T>C
1779	MCMDC2	chr8	ENSG00000178460	Intron	c.226-15del
1780	MCRIP2	chr16	ENSG00000172366	Intron	c.413-68T>A
1781	MCRS1	chr12	ENSG00000187778	Splice region	c.430-5del
1782	MCTP1	chr5	ENSG00000175471	3'UTR	c.*1851A>C
1783	MCTP2	chr15	ENSG00000140563	Intron	c.970-1053del
1784	MCTP2	chr15	ENSG00000140563	3'UTR	c.*1299_*1300del
1785	MDFIC	chr7	ENSG00000135272	5'UTR	c.-131del
1786	MDFIC	chr7	ENSG00000135272	3'UTR	c.*1488T>G
1787	MDGA1	chr6	ENSG00000112139	3'UTR	c.*24del
1788	MDGA1	chr6	ENSG00000112139	Intron	c.178-25G>C
1789	MDGA2	chr14	ENSG00000139915	Frame shift del	c.83del
1790	MDH1B	chr2	ENSG00000138400	anslation_Start_S	c.2T>C
1791	MDM2	chr12	ENSG00000135679	Intron	c.174+936del
1792	MDN1	chr6	ENSG00000112159	Intron	c.7898+10A>G
1793	MDN1	chr6	ENSG00000112159	Intron	c.5350-25del
1794	MECOM	chr3	ENSG00000085276	Missense	c.2696C>T
1795	MECP2	chrX	ENSG00000169057	3'UTR	c.*4508A>G
1796	MECP2	chrX	ENSG00000169057	Intron	c.26+15T>C
1797	MED1	chr17	ENSG00000125686	Splice region	c.852-5del
1798	MED23	chr6	ENSG00000112282	Splice region	c.285-5del
1799	MED28	chr4	ENSG00000118579	3'UTR	c.*5754G>A
1800	MEF2C	chr5	ENSG00000081189	3'UTR	c.*592del

Number	Gene name	Chromosome	Gene_ID	Mutation	Nucleotide substitution
1801	MEF2D	chr1	ENSG00000116604	3'UTR	c.*2551T>G
1802	MEGF6	chr1	ENSG00000162591	Intron	c.1738+27_1738+28del
1803	MEIS1	chr2	ENSG00000143995	3'UTR	c.*518del
1804	MEN1	chr11	ENSG00000133895	Intron	c.446-11C>A
1805	MEPE	chr4	ENSG00000152595	Frame shift del	c.694del
1806	MEPE	chr4	ENSG00000152595	Frame shift del	c.694del
1807	MESD	chr15	ENSG00000117899	Frame shift del	c.632del
1808	MESD	chr15	ENSG00000117899	Frame shift del	c.632del
1809	MESP2	chr15	ENSG00000188095	Intron	c.30+43del
1810	METAP2	chr12	ENSG00000111142	3'UTR	c.*378G>C
1811	METTL23	chr17	ENSG00000181038	Missense	c.34C>A
1812	METTL7A	chr12	ENSG00000185432	3'UTR	c.*95del
1813	MEX3B	chr15	ENSG00000183496	5'UTR	c.-292C>T
1814	MFAP1	chr15	ENSG00000140259	Missense	c.71A>G
1815	MFSD1	chr3	ENSG00000118855	Missense	c.1249T>C
1816	MFSD12	chr19	ENSG00000161091	Silent	c.483T>C
1817	MFSD4A	chr1	ENSG00000174514	3'UTR	c.*1587T>C
1818	MFSD4B	chr6	ENSG00000173214	3'UTR	c.*2412del
1819	MGAM	chr7	ENSG00000257335	Missense	c.1397G>T
1820	MGAT3	chr22	ENSG00000128268	Missense	c.1541G>A
1821	MGAT5B	chr17	ENSG00000167889	Missense	c.307G>A
1822	MGAT5B	chr17	ENSG00000167889	Missense	c.1355G>A
1823	MGAT5B	chr17	ENSG00000167889	Intron	c.2174+14del
1824	MIA2	chr14	ENSG00000150527	Intron	c.384+43del
1825	MIB1	chr18	ENSG00000101752	3'UTR	c.*5875T>C
1826	MICAL1	chr6	ENSG00000135596	Intron	c.1686+52del
1827	MICAL3	chr22	ENSG00000243156	Frame shift del	c.4495del
1828	MICALL2	chr7	ENSG00000164877	Missense	c.1151T>C
1829	MIEF1	chr22	ENSG00000100335	Splice region	c.586-8C>A
1830	MIER3	chr5	ENSG00000155545	3'UTR	c.*198A>G
1831	MINDY4	chr7	ENSG00000106125	Intron	c.2226-37T>C
1832	MINPP1	chr10	ENSG00000107789	Intron	c.835+4582del
1833	MIR200A	chr1	ENSG00000207607	RNA	n.28C>T
1834	MIR4275	chr4	ENSG00000283275	5'Flank	
1835	MIR4457	chr5	ENSG00000263670	3'Flank	
1836	MIS18A	chr21	ENSG00000159055	Silent	c.591C>T
1837	MKI67	chr10	ENSG00000148773	3'UTR	c.*2109C>T
1838	MLF2	chr12	ENSG00000089693	Intron	c.180+52A>G
1839	MLH1	chr3	ENSG00000076242	Intron	c.207+41A>G
1840	MME	chr3	ENSG00000196549	3'UTR	c.*1469del
1841	MME	chr3	ENSG00000196549	3'UTR	c.*1894C>T
1842	MMP12	chr11	ENSG00000262406	Missense	c.1205G>T
1843	MMP19	chr12	ENSG00000123342	3'UTR	c.*486A>G
1844	MNX1	chr7	ENSG00000130675	Frame shift del	c.53del
1845	MNX1	chr7	ENSG00000130675	Frame shift del	c.53del
1846	MOB1A	chr2	ENSG00000114978	Intron	c.15-451del
1847	MOB1B	chr4	ENSG00000173542	3'UTR	c.*5450del
1848	MOB1B	chr4	ENSG00000173542	3'UTR	c.*6050_*6051del
1849	MOB3B	chr9	ENSG00000120162	3'UTR	c.*4987C>A
1850	MON1B	chr16	ENSG00000103111	Intron	c.476-23dup
1851	MON2	chr12	ENSG00000061987	Intron	c.2119-11del
1852	MON2	chr12	ENSG00000061987	3'Flank	
1853	MOSPD1	chrX	ENSG00000101928	3'UTR	c.*639del

Number	Gene name	Chromosome	Gene_ID	Mutation	Nucleotide substitution
1854	MOXD1	chr6	ENSG00000079931	3'UTR	c.*257dup
1855	MOXD1	chr6	ENSG00000079931	3'UTR	c.*774A>G
1856	MPDZ	chr9	ENSG00000107186	5'UTR	c.-200T>C
1857	MPP5	chr14	ENSG00000072415	3'UTR	c.*637dup
1858	MPRIP	chr17	ENSG00000133030	Frame shift del	c.1058del
1859	MPRIP	chr17	ENSG00000133030	Frame shift del	c.1058del
1860	MPZL3	chr11	ENSG00000160588	3'UTR	c.*1664del
1861	MREG	chr2	ENSG00000118242	Intron	c.95+69C>A
1862	MRNIP	chr5	ENSG00000161010	5'Flank	
1863	MROH2A	chr2	ENSG00000185038	Missense	c.3577A>G
1864	MROH2A	chr2	ENSG00000185038	Missense	c.4578G>T
1865	MROH7	chr1	ENSG00000184313	Missense	c.1943C>T
1866	MROH8	chr20	ENSG00000101353	Intron	c.2772-36T>C
1867	MRPL12	chr17	ENSG00000262814	5'UTR	c.-18del
1868	MRPL19	chr2	ENSG00000115364	3'UTR	c.*2588del
1869	MRPL28	chr16	ENSG00000086504	3'UTR	c.*470del
1870	MRPL35	chr2	ENSG00000132313	3'UTR	c.*66_*67ins
1871	MRPL35	chr2	ENSG00000132313	3'UTR	c.*68_*69ins
1872	MRPL42	chr12	ENSG00000198015	3'UTR	c.*2343dup
1873	MRPL42	chr12	ENSG00000198015	3'UTR	c.*2477del
1874	MRPL9	chr1	ENSG00000143436	3'UTR	c.*189A>G
1875	MRPS10	chr6	ENSG00000048544	3'UTR	c.*375del
1876	MRPS10	chr6	ENSG00000048544	Splice region	c.523-3del
1877	MRPS31	chr13	ENSG00000102738	Missense	c.317T>C
1878	MRTO4	chr1	ENSG00000053372	3'UTR	c.*445_*447del
1879	MSL3P1	chr2	ENSG00000224287	RNA	n.889C>T
1880	MSRB3	chr12	ENSG00000174099	3'UTR	c.*520del
1881	MSTO1	chr1	ENSG00000125459	Missense	c.1282A>G
1882	MTA3	chr2	ENSG00000057935	Missense	c.38A>G
1883	MTAP	chr9	ENSG00000099810	Frame shift del	c.622del
1884	MTAP	chr9	ENSG00000099810	Frame shift del	c.622del
1885	MTCH1	chr6	ENSG00000137409	Intron	c.407-39A>G
1886	MTCL1	chr18	ENSG00000168502	Intron	c.4145+17A>G
1887	MTERF1	chr7	ENSG00000127989	Frame shift del	c.729del
1888	MTERF1	chr7	ENSG00000127989	Frame shift del	c.729del
1889	MTF1	chr1	ENSG00000188786	Intron	c.1068+4071del
1890	MTFMT	chr15	ENSG00000103707	3'UTR	c.*503del
1891	MTFR1	chr8	ENSG00000066855	Intron	c.281+47del
1892	MTFR2	chr6	ENSG00000146410	3'UTR	c.*169T>G
1893	MTHFD1L	chr6	ENSG00000120254	Missense	c.2068T>G
1894	MTHFD1L	chr6	ENSG00000120254	3'UTR	c.*223C>T
1895	MTHFD1L	chr6	ENSG00000120254	Missense	c.2601C>A
1896	MTO1	chr6	ENSG00000135297	3'UTR	c.*3831A>T
1897	MTR	chr1	ENSG00000116984	3'UTR	c.*2744T>C
1898	MTSS1	chr8	ENSG00000170873	Intron	c.1048-13del
1899	MTUS1	chr8	ENSG00000129422	3'UTR	c.*413del
1900	MUC17	chr7	ENSG00000169876	Missense	c.12595T>G
1901	MUC19	chr12	ENSG00000205592	Intron	n.315+47del
1902	MUC19	chr12	ENSG00000205592	RNA	n.514del
1903	MUC19	chr12	ENSG00000205592	Intron	n.4063+26del
1904	MUC19	chr12	ENSG00000205592	RNA	n.5543T>C
1905	MVB12A	chr19	ENSG00000141971	Intron	c.414-43C>A
1906	MXD1	chr2	ENSG00000059728	3'UTR	c.*800del

Number	Gene name	Chromosome	Gene_ID	Mutation	Nucleotide substitution
1907	MYADML2	chr17	ENSG00000185105	3'UTR	c.*790C>T
1908	MYBPC3	chr11	ENSG00000134571	Missense	c.3299A>G
1909	MYBPHL	chr1	ENSG00000221986	Splice region	c.145+8A>G
1910	MYH7B	chr20	ENSG00000078814	Missense	c.649A>G
1911	MYLK	chr3	ENSG00000065534	Missense	c.2350T>C
1912	MYLK4	chr6	ENSG00000145949	Intron	c.888-40C>T
1913	MYO18A	chr17	ENSG00000196535	Missense	c.5092A>G
1914	MYO18A	chr17	ENSG00000196535	Intron	c.1729-48T>C
1915	MYO18B	chr22	ENSG00000133454	Missense	c.4771C>A
1916	MYO1C	chr17	ENSG00000197879	Silent	c.771A>G
1917	MYO3A	chr10	ENSG00000095777	3'UTR	c.*1375A>C
1918	MYO3B	chr2	ENSG00000071909	Silent	c.414C>T
1919	MYO5A	chr15	ENSG00000197535	Missense	c.2708G>A
1920	MYO7A	chr11	ENSG00000137474	Missense	c.3891G>T
1921	MYOF	chr10	ENSG00000138119	Intron	c.4210+48_4210+49ins
1922	MYOT	chr5	ENSG00000120729	3'UTR	c.*272A>G
1923	MYRFL	chr12	ENSG00000166268	Intron	c.666+58del
1924	N4BP2	chr4	ENSG00000078177	Splice region	c.1820+11del
1925	N6AMT1	chr21	ENSG00000156239	3'UTR	c.*3593T>C
1926	NAA15	chr4	ENSG00000164134	Silent	c.84T>C
1927	NAA30	chr14	ENSG00000139977	3'UTR	c.*959C>T
1928	NACC2	chr9	ENSG00000148411	3'UTR	c.*4690_*4691del
1929	NACC2	chr9	ENSG00000148411	Intron	c.886+13283del
1930	NAIF1	chr9	ENSG00000171169	Missense	c.161G>T
1931	NANP	chr20	ENSG00000170191	3'UTR	c.*941_*942del
1932	NASP	chr1	ENSG00000132780	Intron	c.60-40T>C
1933	NAT10	chr11	ENSG00000135372	Missense	c.511T>C
1934	NAT16	chr7	ENSG00000167011	3'UTR	c.*711_*712del
1935	NAV1	chr1	ENSG00000134369	3'UTR	c.*6063dup
1936	NAV2	chr11	ENSG00000166833	Intron	c.268-55067_268-55066del
1937	NAV2	chr11	ENSG00000166833	Intron	c.770+4057del
1938	NAV2	chr11	ENSG00000166833	3'UTR	c.*1574del
1939	NBEAL2	chr3	ENSG00000160796	Missense	c.1060G>A
1940	NCAPD2	chr12	ENSG00000010292	Intron	c.3965-17T>C
1941	NCBP1	chr9	ENSG00000136937	3'UTR	c.*833T>C
1942	NCDN	chr1	ENSG00000020129	Silent	c.1656G>A
1943	NCF2	chr1	ENSG00000116701	Intron	c.856-85del
1944	NCOA2	chr8	ENSG00000140396	3'UTR	c.*2744del
1945	NCOA7	chr6	ENSG00000111912	Missense	c.1967A>C
1946	NCOA7	chr6	ENSG00000111912	3'UTR	c.*1359G>T
1947	NCOA7	chr6	ENSG00000111912	3'UTR	c.*1610_*1612dup
1948	NDOR1	chr9	ENSG00000188566	Silent	c.1011A>G
1949	NDRG1	chr8	ENSG00000104419	Intron	c.206-30del
1950	NDUFA11	chr19	ENSG00000174886	Missense	c.220A>G
1951	NEB	chr2	ENSG00000183091	Intron	c.18138+17G>A
1952	NEBL	chr10	ENSG00000078114	Intron	c.798+56del
1953	NEFM	chr8	ENSG00000104722	5'Flank	
1954	NEGR1	chr1	ENSG00000172260	3'UTR	c.*3281del
1955	NEGR1	chr1	ENSG00000172260	Splice region	c.556+4A>C
1956	NEK4	chr3	ENSG00000114904	In_Frame_Del	c.742_744del
1957	NEK5	chr13	ENSG00000197168	Splice region	c.1573-3del
1958	NEK6	chr9	ENSG00000119408	3'UTR	c.*1598C>A
1959	NEMP1	chr12	ENSG00000166881	3'UTR	c.*3543del

Number	Gene name	Chromosome	Gene_ID	Mutation	Nucleotide substitution
1960	NEUROD4	chr12	ENSG00000123307	3'UTR	c.*1076del
1961	NF1	chr17	ENSG00000196712	Intron	c.2251+25del
1962	NF1	chr17	ENSG00000196712	Missense	c.7602A>T
1963	NF1	chr17	ENSG00000196712	3'UTR	c.*589dup
1964	NF1P5	chr18	ENSG00000175319	Intron	n.366+45del
1965	NFAM1	chr22	ENSG00000235568	Missense	c.335T>C
1966	NFAT5	chr16	ENSG00000102908	Silent	c.2637A>G
1967	NFATC2	chr20	ENSG00000101096	3'UTR	c.*2728del
1968	NFE2L2	chr2	ENSG00000116044	Silent	c.1179T>C
1969	NFE2L3	chr7	ENSG00000050344	Frame shift del	c.458del
1970	NFIB	chr9	ENSG00000147862	3'UTR	c.*5756del
1971	NFIB	chr9	ENSG00000147862	3'UTR	c.*5749A>G
1972	NHLH1	chr1	ENSG00000171786	Missense	c.296G>A
1973	NHS	chrX	ENSG00000188158	3'UTR	c.*3461del
1974	NHSL1	chr6	ENSG00000135540	Missense	c.1714A>G
1975	NICN1	chr3	ENSG00000145029	3'UTR	c.*156T>A
1976	NINL	chr20	ENSG00000101004	Missense	c.1113C>A
1977	NIPA1	chr15	ENSG00000170113	3'UTR	c.*2322T>C
1978	NIPBL	chr5	ENSG00000164190	Frame shift del	c.1808del
1979	NIPBL	chr5	ENSG00000164190	Frame shift del	c.1808del
1980	NKD1	chr16	ENSG00000140807	Intron	c.26-26G>A
1981	NKIRAS2	chr17	ENSG00000168256	3'UTR	c.*884_*886del
1982	NKIRAS2	chr17	ENSG00000168256	3'UTR	c.*884_*885del
1983	NKRF	chrX	ENSG00000186416	3'UTR	c.*956_*957del
1984	NKTR	chr3	ENSG00000114857	3'UTR	c.*221del
1985	NKX2-1	chr14	ENSG00000136352	Intron	c.78-340del
1986	NKX2-3	chr10	ENSG00000119919	Missense	c.143C>T
1987	NKX2-4	chr20	ENSG00000125816	3'UTR	c.*20C>A
1988	NKX6-1	chr4	ENSG00000163623	3'UTR	c.*479del
1989	NLGN2	chr17	ENSG00000169992	3'UTR	c.*232del
1990	NLK	chr17	ENSG00000087095	3'UTR	c.*1538del
1991	NLRP12	chr19	ENSG00000142405	Frame shift del	c.2188del
1992	NMRK1	chr9	ENSG00000106733	Intron	c.580+538A>G
1993	NMT2	chr10	ENSG00000152465	Intron	c.207+2557T>C
1994	NNT	chr5	ENSG00000112992	3'UTR	c.*434del
1995	NNT	chr5	ENSG00000112992	3'UTR	c.*2073del
1996	NOC3L	chr10	ENSG00000173145	3'UTR	c.*584del
1997	NOD1	chr7	ENSG00000106100	Missense	c.2459G>T
1998	NOL11	chr17	ENSG00000130935	Intron	c.1843-78del
1999	NOL6	chr9	ENSG00000165271	3'UTR	c.*1085C>T
2000	NOP9	chr14	ENSG00000196943	Intron	c.697+10A>G
2001	NOS3	chr7	ENSG00000164867	Intron	c.3255+54A>G
2002	NOSIP	chr19	ENSG00000142546	Intron	c.735-39del
2003	NOTCH2	chr1	ENSG00000134250	3'UTR	c.*415C>T
2004	NOVA1	chr14	ENSG00000139910	3'UTR	c.*641C>T
2005	NPAP1	chr15	ENSG00000185823	Silent	c.1140A>G
2006	NPAP1	chr15	ENSG00000185823	3'UTR	c.*3416dup
2007	NPEPPS	chr17	ENSG00000141279	3'UTR	c.*637dup
2008	NPPC	chr2	ENSG00000163273	Intron	c.90+19A>G
2009	NPR3	chr5	ENSG00000113389	3'UTR	c.*4702T>A
2010	NPRL2	chr3	ENSG00000114388	Intron	c.78+27C>T
2011	NR1D1	chr17	ENSG00000126368	Splice site	c.605-2A>G
2012	NR1D2	chr3	ENSG00000174738	3'UTR	c.*1288_*1289dup

Number	Gene name	Chromosome	Gene_ID	Mutation	Nucleotide substitution
2013	NR1I2	chr3	ENSG00000144852	Missense	c.203T>C
2014	NR2F2	chr15	ENSG00000185551	3'UTR	c.*375del
2015	NR5A1	chr9	ENSG00000136931	3'UTR	c.*1400del
2016	NR6A1	chr9	ENSG00000148200	Intron	c.142+40913C>A
2017	NRAS	chr1	ENSG00000213281	Missense	c.34G>T
2018	NRBF2	chr10	ENSG00000148572	Missense	c.118T>C
2019	NRBP2	chr8	ENSG00000185189	Missense	c.914T>C
2020	NRCAM	chr7	ENSG00000091129	3'UTR	c.*534A>C
2021	NRF1	chr7	ENSG00000106459	Splice region	c.1405+4G>T
2022	NRG4	chr15	ENSG00000169752	Intron	c.251+2677del
2023	NRIP1	chr21	ENSG00000180530	Nonsense	c.451C>T
2024	NRIP3	chr11	ENSG00000175352	3'Flank	
2025	NRIP3	chr11	ENSG00000175352	3'Flank	
2026	NRP1	chr10	ENSG00000099250	Intron	c.1138-11del
2027	NRXN1	chr2	ENSG00000179915	3'UTR	c.*2015dup
2028	NRXN1	chr2	ENSG00000179915	Intron	c.3245-942del
2029	NRXN1	chr2	ENSG00000179915	Intron	c.2374+67G>A
2030	NRXN1	chr2	ENSG00000179915	Intron	c.772+48205G>A
2031	NRXN3	chr14	ENSG00000021645	3'UTR	c.*1564del
2032	NSMCE2	chr8	ENSG00000156831	Splice region	c.519+3A>G
2033	NSMCE4A	chr10	ENSG00000107672	Missense	c.899G>A
2034	NSMF	chr9	ENSG00000165802	Silent	c.1525T>C
2035	NSUN4	chr1	ENSG00000117481	Missense	c.731T>C
2036	NT5C1A	chr1	ENSG00000116981	Missense	c.1030G>A
2037	NT5C3A	chr7	ENSG00000122643	3'UTR	c.*222_*223insTCTT
2038	NT5DC1	chr6	ENSG00000178425	Missense	c.251T>C
2039	NTM	chr11	ENSG00000182667	3'UTR	c.*1256_*1257del
2040	NTN4	chr12	ENSG00000074527	Missense	c.475T>C
2041	NTN5	chr19	ENSG00000142233	5'Flank	
2042	NTNG1	chr1	ENSG00000162631	3'UTR	c.*2423A>G
2043	NTRK1	chr1	ENSG00000198400	5'UTR	c.-89G>A
2044	NTRK1	chr1	ENSG00000198400	Splice region	c.359+4A>G
2045	NTRK3	chr15	ENSG00000140538	3'UTR	c.*8776A>C
2046	NTRK3	chr15	ENSG00000140538	3'UTR	c.*2698A>G
2047	NTS	chr12	ENSG00000133636	Intron	c.136-13del
2048	NUAK2	chr1	ENSG00000163545	Missense	c.1133G>A
2049	NUBP1	chr16	ENSG00000103274	Intron	c.452-59A>T
2050	NUCKS1	chr1	ENSG00000069275	3'UTR	c.*2605G>T
2051	NUDT9	chr4	ENSG00000170502	3'UTR	c.*647C>A
2052	NUFIP1	chr13	ENSG00000083635	Intron	c.658-9del
2053	NUMA1	chr11	ENSG00000137497	Intron	c.-102-2486A>G
2054	NUP107	chr12	ENSG00000111581	Intron	c.1998+19del
2055	NUP205	chr7	ENSG00000155561	Intron	c.1474-1647C>T
2056	NUP210L	chr1	ENSG00000143552	Silent	c.2388A>C
2057	NUP214	chr9	ENSG00000126883	3'UTR	c.*1111del
2058	NUP85	chr17	ENSG00000125450	Missense	c.802T>C
2059	NUP85	chr17	ENSG00000125450	Silent	c.870C>T
2060	NUS1	chr6	ENSG00000153989	Splice region	c.691+12del
2061	NUS1	chr6	ENSG00000153989	3'UTR	c.*2903del
2062	NUSAP1	chr15	ENSG00000137804	Frame shift del	c.479del
2063	NUSAP1	chr15	ENSG00000137804	Frame shift del	c.479del
2064	NUTM2F	chr9	ENSG00000130950	Missense	c.1223C>A
2065	NWD1	chr19	ENSG00000188039	Intron	c.496+428A>G

Number	Gene name	Chromosome	Gene_ID	Mutation	Nucleotide substitution
2066	NXPE2	chr11	ENSG00000204361	5'Flank	
2067	NXPH3	chr17	ENSG00000182575	Missense	c.710G>T
2068	NYAP1	chr7	ENSG00000166924	Missense	c.895G>A
2069	OARD1	chr6	ENSG00000124596	3'UTR	c.*1030del
2070	OAS3	chr12	ENSG00000111331	Silent	c.189G>A
2071	OBSCN	chr1	ENSG00000154358	Silent	c.6951C>T
2072	OBSCN	chr1	ENSG00000154358	Missense	c.7222C>T
2073	OBSCN	chr1	ENSG00000154358	Splice region	c.14502+12dup
2074	OCM	chr7	ENSG00000122543	Intron	c.-3-112del
2075	OCRL	chrX	ENSG00000122126	Splice region	c.40-3C>T
2076	ODAPH	chr4	ENSG00000174792	3'UTR	c.*1379del
2077	ODF2	chr9	ENSG00000136811	Intron	c.829-20del
2078	ODF2L	chr1	ENSG00000122417	3'UTR	c.*2489del
2079	OGA	chr10	ENSG00000198408	3'UTR	c.*591del
2080	OGDH	chr7	ENSG00000105953	Silent	c.264A>G
2081	OLFM3	chr1	ENSG00000118733	3'UTR	c.*1077del
2082	OLFM3	chr1	ENSG00000118733	3'UTR	c.*43T>C
2083	OLR1	chr12	ENSG00000173391	5'UTR	c.-146A>G
2084	ONECUT1	chr15	ENSG00000169856	3'Flank	
2085	OOEP	chr6	ENSG00000203907	5'UTR	c.-131del
2086	OPCML	chr11	ENSG00000183715	3'UTR	c.*2975A>G
2087	OPCML	chr11	ENSG00000183715	Intron	c.168-43_168-42dup
2088	OPLAH	chr8	ENSG00000178814	Intron	c.3019-32T>C
2089	OR10G3	chr14	ENSG00000169208	Missense	c.359C>T
2090	OR13D1	chr9	ENSG00000179055	anslation_Start_S	c.1A>G
2091	OR4C16	chr11	ENSG00000279514	Missense	c.556_557delinsTT
2092	OR4C16	chr11	ENSG00000279514	Missense	c.556_557delinsTT
2093	OR51I2	chr11	ENSG00000187918	Missense	c.392G>A
2094	OR52A1	chr11	ENSG00000182070	Intron	c.-321-9del
2095	OR52A4P	chr11	ENSG00000205494	3'Flank	
2096	OR5B17	chr11	ENSG00000197786	Missense	c.169A>G
2097	ORC2	chr2	ENSG00000115942	3'UTR	c.*1530_*1531del
2098	OSBP2	chr22	ENSG00000184792	3'UTR	c.*135del
2099	OSBPL1A	chr18	ENSG00000141447	Intron	c.2535+30A>G
2100	OSBPL3	chr7	ENSG00000070882	3'UTR	c.*2447del
2101	OSBPL6	chr2	ENSG00000079156	Frame shift del	c.2363del
2102	OSBPL6	chr2	ENSG00000079156	Frame shift del	c.2363del
2103	OSGIN2	chr8	ENSG00000164823	Missense	c.125T>G
2104	OSTM1	chr6	ENSG00000081087	3'Flank	
2105	OSTN	chr3	ENSG00000188729	3'UTR	c.*2387C>T
2106	OTOGL	chr12	ENSG00000165899	Intron	c.1977+22G>A
2107	OTUB1	chr11	ENSG00000167770	3'UTR	c.*99_*100insA
2108	OTUD4	chr4	ENSG00000164164	3'UTR	c.*3546del
2109	OTUD7B	chr1	ENSG00000264522	Silent	c.2103G>A
2110	OTX2	chr14	ENSG00000165588	Frame shift del	c.106del
2111	OVOL1	chr11	ENSG00000172818	3'UTR	c.*590T>C
2112	OXGR1	chr13	ENSG00000165621	Frame shift del	c.764del
2113	OXGR1	chr13	ENSG00000165621	Frame shift del	c.764del
2114	P2RX1	chr17	ENSG00000108405	Missense	c.74G>A
2115	PACSIN2	chr22	ENSG00000100266	Intron	c.907-62C>T
2116	PAK6	chr15	ENSG00000137843	5'UTR	c.-105C>T
2117	PAK6	chr15	ENSG00000137843	Intron	c.1356+48T>C
2118	PAMR1	chr11	ENSG00000149090	Missense	c.1751T>G

Number	Gene name	Chromosome	Gene_ID	Mutation	Nucleotide substitution
2119	PANX2	chr22	ENSG00000073150	3'UTR	c.*245A>G
2120	PAOX	chr10	ENSG00000148832	Intron	c.1393-481dup
2121	PAPOLA	chr14	ENSG00000090060	3'UTR	c.*372dup
2122	PAPPA	chr9	ENSG00000182752	3'UTR	c.*1243T>C
2123	PAPPA	chr9	ENSG00000182752	3'UTR	c.*3652T>C
2124	PARD6B	chr20	ENSG00000124171	3'UTR	c.*2587_*2589del
2125	PARG	chr10	ENSG00000227345	Splice region	c.218-5del
2126	PARK7	chr1	ENSG00000116288	Frame shift del	c.189del
2127	PARK7	chr1	ENSG00000116288	Frame shift del	c.189del
2128	PARK7	chr1	ENSG00000116288	Intron	c.252+47A>G
2129	PARM1	chr4	ENSG00000169116	3'UTR	c.*3686G>A
2130	PARP11	chr12	ENSG00000111224	Intron	c.19-15231G>A
2131	PARP14	chr3	ENSG00000173193	3'UTR	c.*345G>A
2132	PARP2	chr14	ENSG00000129484	Missense	c.1192A>G
2133	PARP8	chr5	ENSG00000151883	3'UTR	c.*4224A>G
2134	PARP9	chr3	ENSG00000138496	3'UTR	c.*4264del
2135	PARP9	chr3	ENSG00000138496	Frame shift del	c.1844del
2136	PASK	chr2	ENSG00000115687	Intron	c.2720-23G>A
2137	PATJ	chr1	ENSG00000132849	3'UTR	c.*2571A>G
2138	PATZ1	chr22	ENSG00000100105	3'UTR	c.*66T>G
2139	PAWR	chr12	ENSG00000177425	3'UTR	c.*7126T>C
2140	PAWR	chr12	ENSG00000177425	3'UTR	c.*641A>G
2141	PAX6	chr11	ENSG00000007372	5'UTR	c.-571dup
2142	PAX7	chr1	ENSG00000009709	Silent	c.306C>T
2143	PAXBP1	chr21	ENSG00000159086	Intron	c.2191-34T>C
2144	PBXIP1	chr1	ENSG00000163346	3'UTR	c.*497T>C
2145	PCBP2	chr12	ENSG00000197111	Intron	c.790-66del
2146	PCBP2	chr12	ENSG00000197111	Nonstop_Mutation	c.994T>C
2147	PCDH15	chr10	ENSG00000150275	Intron	c.4367+37G>A
2148	PCDHA5	chr5	ENSG00000204965	Missense	c.296C>T
2149	PCDHA7	chr5	ENSG00000204963	Missense	c.547C>T
2150	PCDHB1	chr5	ENSG00000171815	Missense	c.1315G>A
2151	PCDHB13	chr5	ENSG00000187372	3'UTR	c.*25A>G
2152	PCDHB6	chr5	ENSG00000113211	3'Flank	
2153	PCDHGA2	chr5	ENSG00000081853	Missense	c.992C>T
2154	PCGF3	chr4	ENSG00000185619	3'UTR	c.*1246del
2155	PCGF6	chr10	ENSG00000156374	Intron	c.585+18del
2156	PCLO	chr7	ENSG00000186472	Splice region	c.14047-4A>G
2157	PCMT1	chr6	ENSG00000120265	Intron	c.*37+199_*37+200del
2158	PCMTD1	chr8	ENSG00000168300	3'UTR	c.*66dup
2159	PCNX1	chr14	ENSG00000100731	Intron	c.3151-73A>G
2160	PCSK5	chr9	ENSG00000099139	3'UTR	c.*195G>C
2161	PCSK7	chr11	ENSG00000160613	3'UTR	c.*726A>G
2162	PDCD11	chr10	ENSG00000148843	Silent	c.804T>A
2163	PDCD1LG2	chr9	ENSG00000197646	Intron	c.55+22G>T
2164	PDE10A	chr6	ENSG00000112541	3'UTR	c.*1967del
2165	PDE4B	chr1	ENSG00000184588	Splice region	c.281+8G>C
2166	PDGFA	chr7	ENSG00000197461	3'UTR	c.*1291_*1292del
2167	PDGFA	chr7	ENSG00000197461	Intron	c.*13+28T>C
2168	PDGFD	chr11	ENSG00000170962	5'UTR	c.-396C>T
2169	PDHX	chr11	ENSG00000110435	Intron	c.115+107A>G
2170	PDLIM5	chr4	ENSG00000163110	Intron	c.920+75A>G
2171	PDP2	chr16	ENSG00000172840	3'UTR	c.*295A>G

Number	Gene name	Chromosome	Gene_ID	Mutation	Nucleotide substitution
2172	PDPK1	chr16	ENSG00000140992	3'UTR	c.*5332del
2173	PDPN	chr1	ENSG00000162493	Intron	c.560-20T>A
2174	PDPN	chr1	ENSG00000162493	3'UTR	c.*64G>A
2175	PDS5A	chr4	ENSG00000121892	Missense	c.307C>T
2176	PDSS2	chr6	ENSG00000164494	3'UTR	c.*2032del
2177	PDXK	chr21	ENSG00000160209	3'UTR	c.*1318T>C
2178	PDXK	chr21	ENSG00000160209	3'UTR	c.*1329A>G
2179	PDZRN3	chr3	ENSG00000121440	5'Flank	
2180	PEAK1	chr15	ENSG00000173517	Missense	c.733A>G
2181	PEAR1	chr1	ENSG00000187800	Missense	c.1531T>C
2182	PELI2	chr14	ENSG00000139946	3'UTR	c.*309del
2183	PELI3	chr11	ENSG00000174516	3'UTR	c.*5C>A
2184	PER2	chr2	ENSG00000132326	Missense	c.296G>A
2185	PEX10	chr1	ENSG00000157911	3'UTR	c.*894A>G
2186	PEX5	chr12	ENSG00000139197	Missense	c.1142T>C
2187	PEX5L	chr3	ENSG00000114757	3'UTR	c.*5952del
2188	PEX5L	chr3	ENSG00000114757	3'UTR	c.*5313del
2189	PFKFB2	chr1	ENSG00000123836	3'UTR	c.*1783C>T
2190	PFKP	chr10	ENSG00000067057	Intron	c.1660-717C>T
2191	PGAP1	chr2	ENSG00000197121	Intron	c.1768-13A>G
2192	PGAP1	chr2	ENSG00000197121	Missense	c.472T>C
2193	PGBD4	chr15	ENSG00000182405	Missense	c.1385T>C
2194	PGM2	chr4	ENSG00000169299	Silent	c.345T>C
2195	PGM3	chr6	ENSG00000013375	Missense	c.701A>G
2196	PGM5	chr9	ENSG00000154330	3'UTR	c.*107T>G
2197	PHACTR1	chr6	ENSG00000112137	3'UTR	c.*73A>G
2198	PHACTR1	chr6	ENSG00000112137	3'UTR	c.*1442C>T
2199	PHACTR3	chr20	ENSG00000087495	3'UTR	c.*324G>A
2200	PHB	chr17	ENSG00000167085	Missense	c.470G>A
2201	PHETA1	chr12	ENSG00000198324	3'UTR	c.*2022del
2202	PHF13	chr1	ENSG00000116273	3'UTR	c.*462G>A
2203	PHF20L1	chr8	ENSG00000129292	Intron	c.769+33dup
2204	PHF24	chr9	ENSG00000122733	Silent	c.480C>A
2205	PHF6	chrX	ENSG00000156531	3'UTR	c.*1507del
2206	PHF6	chrX	ENSG00000156531	3'UTR	c.*2170del
2207	PHF7	chr3	ENSG00000010318	Intron	c.42-85T>C
2208	PHF7	chr3	ENSG00000010318	Missense	c.1084A>G
2209	PHLDB1	chr11	ENSG00000019144	Intron	c.3631-64T>C
2210	PHLDB2	chr3	ENSG00000144824	Intron	c.2824+61del
2211	PHLDB3	chr19	ENSG00000176531	Intron	c.1703-50C>G
2212	PIEZO1	chr16	ENSG00000103335	Intron	c.5949+51C>T
2213	PIEZO1	chr16	ENSG00000103335	Intron	c.4496-12G>A
2214	PIGT	chr20	ENSG00000124155	Missense	c.1060T>C
2215	PIK3C2B	chr1	ENSG00000133056	Missense	c.1439G>A
2216	PIK3C3	chr18	ENSG00000078142	3'UTR	c.*3093A>G
2217	PIK3CA	chr3	ENSG00000121879	3'UTR	c.*693del
2218	PIK3R1	chr5	ENSG00000145675	Intron	c.119+74A>G
2219	PIK3R3	chr1	ENSG00000117461	Frame shift del	c.883del
2220	PIKFYVE	chr2	ENSG00000115020	Silent	c.1749A>G
2221	PIM2	chrX	ENSG00000102096	3'UTR	c.*575_*576del
2222	PIP4P2	chr8	ENSG00000155099	3'UTR	c.*1277del
2223	PIP5K1C	chr19	ENSG00000186111	Frame shift del	c.2066del
2224	PIP5K1C	chr19	ENSG00000186111	Frame shift del	c.2066del

Number	Gene name	Chromosome	Gene_ID	Mutation	Nucleotide substitution
2225	PIP5K1C	chr19	ENSG00000186111	Missense	c.1481C>G
2226	PIPSL	chr10	ENSG00000180764	RNA	n.891C>T
2227	PITPNB	chr22	ENSG00000180957	3'UTR	c.*1187dup
2228	PITPNM2	chr12	ENSG00000090975	Intron	c.3709-19G>A
2229	PITPNM2	chr12	ENSG00000090975	Missense	c.3244A>G
2230	PITPNM2	chr12	ENSG00000090975	Missense	c.1948C>T
2231	PITPNM3	chr17	ENSG00000091622	3'UTR	c.*3932G>A
2232	PITX3	chr10	ENSG00000107859	3'Flank	
2233	PJA2	chr5	ENSG00000198961	Missense	c.478A>G
2234	PKD1L2	chr16	ENSG00000166473	5'Flank	
2235	PKD2	chr4	ENSG00000118762	3'UTR	c.*1355del
2236	PKIA	chr8	ENSG00000171033	3'UTR	c.*602C>A
2237	PKIA	chr8	ENSG00000171033	3'UTR	c.*1408A>G
2238	PKN1	chr19	ENSG00000123143	Missense	c.983T>C
2239	PKNOX1	chr21	ENSG00000160199	Frame shift Ins	c.879dup
2240	PKP2	chr12	ENSG00000057294	3'UTR	c.*1311del
2241	PKP3	chr11	ENSG00000184363	Intron	c.1567-27G>A
2242	PKP4	chr2	ENSG00000144283	Missense	c.1561A>G
2243	PLA2G2C	chr1	ENSG00000187980	3'UTR	c.*2733del
2244	PLA2G2C	chr1	ENSG00000187980	3'UTR	c.*632G>T
2245	PLA2G2F	chr1	ENSG00000158786	5'UTR	c.-95G>A
2246	PLA2G4C	chr19	ENSG00000105499	Missense	c.293T>C
2247	PLA2G6	chr22	ENSG00000184381	Silent	c.75G>A
2248	PLA2R1	chr2	ENSG00000153246	3'UTR	c.*1795C>G
2249	PLAA	chr9	ENSG00000137055	3'UTR	c.*355T>C
2250	PLAA	chr9	ENSG00000137055	Intron	c.1418-24C>A
2251	PLCB3	chr11	ENSG00000149782	Missense	c.929T>C
2252	PLCH1	chr3	ENSG00000114805	Missense	c.3167C>A
2253	PLCXD3	chr5	ENSG00000182836	3'UTR	c.*5922del
2254	PLEKHG3	chr14	ENSG00000126822	Missense	c.2098T>C
2255	PLEKHG4B	chr5	ENSG00000153404	Silent	c.1881C>T
2256	PLOD3	chr7	ENSG00000106397	Missense	c.541C>T
2257	PLPP3	chr1	ENSG00000162407	Missense	c.320G>A
2258	PLPP6	chr9	ENSG00000205808	3'UTR	c.*1565_*1566delinsGC
2259	PLS1	chr3	ENSG00000120756	Intron	c.1629+23del
2260	PLS1	chr3	ENSG00000120756	3'UTR	c.*602del
2261	PLSCR1	chr3	ENSG00000188313	Splice region	c.356-3dup
2262	PLSCR2	chr3	ENSG00000163746	Splice region	c.-83-3del
2263	PLSCR4	chr3	ENSG00000114698	3'UTR	c.*1597del
2264	PLSCR4	chr3	ENSG00000114698	3'UTR	c.*1270del
2265	PLXDC1	chr17	ENSG00000161381	Intron	c.990-444C>T
2266	PLXNA1	chr3	ENSG00000114554	Intron	c.4363-267T>C
2267	PLXNA4	chr7	ENSG00000221866	3'UTR	c.*2088G>A
2268	PLXNB1	chr3	ENSG00000164050	Frame shift del	c.2653del
2269	PLXNB1	chr3	ENSG00000164050	Frame shift del	c.2653del
2270	PLXNB2	chr22	ENSG00000196576	Intron	c.4647+34G>A
2271	PLXNB2	chr22	ENSG00000196576	Missense	c.3922C>T
2272	PLXNB2	chr22	ENSG00000196576	Splice region	c.1252-8G>A
2273	PLXNC1	chr12	ENSG00000136040	Missense	c.4249C>T
2274	PM20D2	chr6	ENSG00000146281	3'UTR	c.*1013G>A
2275	PMEPA1	chr20	ENSG00000124225	3'UTR	c.*736del
2276	PMF1	chr1	ENSG00000160783	Missense	c.425A>G
2277	PNISR	chr6	ENSG00000132424	Intron	c.1102+72del

Number	Gene name	Chromosome	Gene_ID	Mutation	Nucleotide substitution
2278	PNMA1	chr14	ENSG00000176903	3'UTR	c.*782T>C
2279	PNMA1	chr14	ENSG00000176903	3'UTR	c.*600del
2280	PNPLA6	chr19	ENSG00000032444	Silent	c.1557G>A
2281	PNPLA8	chr7	ENSG00000135241	Silent	c.192A>G
2282	PNPT1	chr2	ENSG00000138035	Intron	c.1177-88del
2283	PNPT1	chr2	ENSG00000138035	Missense	c.76C>T
2284	PNRC2	chr1	ENSG00000189266	3'UTR	c.*526T>C
2285	PNRC2P1	chr1	ENSG00000228217	3'Flank	
2286	POFUT1	chr20	ENSG00000101346	3'UTR	c.*1295del
2287	POFUT1	chr20	ENSG00000101346	3'UTR	c.*3870del
2288	POGZ	chr1	ENSG00000143442	Missense	c.2915A>C
2289	POLA2	chr11	ENSG00000014138	3'UTR	c.*720A>C
2290	POLA2	chr11	ENSG00000014138	3'UTR	c.*20A>G
2291	POLD2	chr7	ENSG00000106628	Missense	c.763A>G
2292	POLE	chr12	ENSG00000177084	Splice region	c.4005G>A
2293	POLE	chr12	ENSG00000177084	Missense	c.1616A>G
2294	POLL	chr10	ENSG00000166169	Silent	c.1269G>A
2295	POLQ	chr3	ENSG00000051341	Missense	c.4425T>G
2296	POLR2A	chr17	ENSG00000181222	Missense	c.2899A>G
2297	POLR2E	chr19	ENSG00000099817	Intron	c.260-40dup
2298	POLR2M	chr15	ENSG00000255529	3'UTR	c.*915del
2299	POLR3A	chr10	ENSG00000148606	Silent	c.2397G>A
2300	POLR3F	chr20	ENSG00000132664	Intron	c.180+58del
2301	POLR3G	chr5	ENSG00000113356	Intron	c.438+39dup
2302	POMT2	chr14	ENSG00000009830	Intron	c.1576+74C>T
2303	POU2F1	chr1	ENSG00000143190	Intron	c.781-2321T>A
2304	POU2F2	chr19	ENSG00000028277	3'UTR	c.*11T>C
2305	POU5F2	chr5	ENSG00000248483	Missense	c.497G>A
2306	POU6F2	chr7	ENSG00000106536	3'UTR	c.*725del
2307	PPA2	chr4	ENSG00000138777	Intron	c.784-21del
2308	PPARA	chr22	ENSG00000186951	3'UTR	c.*889del
2309	PPARA	chr22	ENSG00000186951	3'UTR	c.*1496A>G
2310	PPARGC1B	chr5	ENSG00000155846	Intron	c.1742+35T>G
2311	PPARGC1B	chr5	ENSG00000155846	Missense	c.2324T>C
2312	PPARGC1B	chr5	ENSG00000155846	3'UTR	c.*6844_*6849del
2313	PPFIA1	chr11	ENSG00000131626	Missense	c.1372T>C
2314	PPFIA3	chr19	ENSG00000177380	Missense	c.1667A>T
2315	PPFIBP1	chr12	ENSG00000110841	3'UTR	c.*1963A>G
2316	PPIA	chr7	ENSG00000196262	3'UTR	c.*754T>C
2317	PPID	chr4	ENSG00000171497	Intron	c.646-39A>G
2318	PPIH	chr1	ENSG00000171960	Missense	c.418G>C
2319	PPIL2	chr22	ENSG00000100023	Intron	c.715-81T>C
2320	PPIL6	chr6	ENSG00000185250	Intron	c.560-4890del
2321	PPM1A	chr14	ENSG00000100614	3'UTR	c.*5632del
2322	PPM1A	chr14	ENSG00000100614	3'UTR	c.*5764T>C
2323	PPM1B	chr2	ENSG00000138032	3'UTR	c.*456C>T
2324	PPM1D	chr17	ENSG00000170836	Frame shift del	c.1535del
2325	PPM1D	chr17	ENSG00000170836	Frame shift del	c.1535del
2326	PPM1L	chr3	ENSG00000163590	5'UTR	c.-546T>G
2327	PPM1M	chr3	ENSG00000164088	Missense	c.94C>G
2328	PPP1R13B	chr14	ENSG00000088808	3'UTR	c.*1154del
2329	PPP1R15B	chr1	ENSG00000158615	3'UTR	c.*183C>A
2330	PPP1R16B	chr20	ENSG00000101445	3'Flank	

Number	Gene name	Chromosome	Gene_ID	Mutation	Nucleotide substitution
2331	PPP1R27	chr17	ENSG00000182676	Missense	c.47G>A
2332	PPP2R1B	chr11	ENSG00000137713	3'UTR	c.*3080C>G
2333	PPP2R3A	chr3	ENSG00000073711	3'UTR	c.*1829del
2334	PPP2R5C	chr14	ENSG00000078304	5'UTR	c.-131C>G
2335	PPP2R5C	chr14	ENSG00000078304	Intron	c.260-20522del
2336	PPP4R1	chr18	ENSG00000154845	Missense	c.632A>G
2337	PPP4R3A	chr14	ENSG00000100796	3'UTR	c.*573_*574del
2338	PPP4R3B	chr2	ENSG00000275052	Frame shift del	c.2004del
2339	PPP6C	chr9	ENSG00000119414	Intron	c.171+42T>C
2340	PPP6R1	chr19	ENSG00000105063	Missense	c.1142A>T
2341	PRAC2	chr17	ENSG00000229637	Frame shift Ins	c.101dup
2342	PRAC2	chr17	ENSG00000229637	Frame shift del	c.102del
2343	PRDM15	chr21	ENSG00000141956	3'UTR	c.*734_*735del
2344	PRDM16	chr1	ENSG00000142611	Missense	c.49G>A
2345	PRDM16	chr1	ENSG00000142611	3'UTR	c.*1625C>A
2346	PRDM4	chr12	ENSG00000110851	Splice region	c.1610-6del
2347	PRDM6	chr5	ENSG00000061455	Missense	c.838G>A
2348	PRDX2	chr19	ENSG00000167815	Missense	c.81G>T
2349	PRDX3	chr10	ENSG00000165672	Missense	c.238G>A
2350	PRDX6	chr1	ENSG00000117592	3'UTR	c.*804G>A
2351	PRKACB	chr1	ENSG00000142875	3'UTR	c.*2867A>G
2352	PRKCB	chr16	ENSG00000166501	Silent	c.498C>A
2353	PRKCB	chr16	ENSG00000166501	3'UTR	c.*1185del
2354	PRKCZ	chr1	ENSG00000067606	Intron	c.334+14374G>A
2355	PRKCZ	chr1	ENSG00000067606	Missense	c.886T>G
2356	PRKD3	chr2	ENSG00000115825	3'UTR	c.*1296del
2357	PRKDC	chr8	ENSG00000253729	Intron	c.12182+27T>C
2358	PRKDC	chr8	ENSG00000253729	Missense	c.10238A>G
2359	PRKG1	chr10	ENSG00000185532	Intron	c.478+146279C>A
2360	PRL	chr6	ENSG00000172179	Intron	c.493-11del
2361	PRLHR	chr10	ENSG00000119973	Missense	c.946G>A
2362	PRLR	chr5	ENSG00000113494	3'UTR	c.*9097A>G
2363	PRMT6	chr1	ENSG00000198890	Missense	c.425C>G
2364	PRMT7	chr16	ENSG00000132600	Missense	c.1087C>T
2365	PRMT9	chr4	ENSG00000164169	Missense	c.923T>C
2366	PROC	chr2	ENSG00000115718	5'UTR	c.-10T>C
2367	PROCR	chr20	ENSG00000101000	Missense	c.131A>G
2368	PROM1	chr4	ENSG00000007062	Frame shift del	c.1850del
2369	PROM1	chr4	ENSG00000007062	Frame shift del	c.1850del
2370	PRORP	chr14	ENSG00000100890	3'Flank	
2371	PROS1	chr3	ENSG00000184500	Intron	c.602-23del
2372	PROSER3	chr19	ENSG00000167595	Missense	c.497C>T
2373	PRPF19	chr11	ENSG00000110107	Missense	c.1507A>G
2374	PRPF39	chr14	ENSG00000185246	Intron	c.570-705del
2375	PRPF40A	chr2	ENSG00000196504	5'UTR	c.-1del
2376	PRR14L	chr22	ENSG00000183530	3'UTR	c.*1573A>G
2377	PRR19	chr19	ENSG00000188368	Silent	c.15A>G
2378	PRR22	chr19	ENSG00000212123	Silent	c.891A>G
2379	PRR27	chr4	ENSG00000187533	Intron	n.166+83del
2380	PRRC2B	chr9	ENSG00000130723	Splice region	c.4614T>C
2381	PRRC2B	chr9	ENSG00000130723	Intron	c.5373+36del
2382	PRRC2B	chr9	ENSG00000130723	3'UTR	c.*242T>A
2383	PRRC2C	chr1	ENSG00000117523	Missense	c.485A>G

Number	Gene name	Chromosome	Gene_ID	Mutation	Nucleotide substitution
2384	PRRC2C	chr1	ENSG00000117523	Frame shift del	c.2906del
2385	PRRC2C	chr1	ENSG00000117523	Frame shift del	c.2906del
2386	PRRC2C	chr1	ENSG00000117523	3'UTR	c.*872del
2387	PRRT3	chr3	ENSG00000163704	3'UTR	c.*512del
2388	PRRT4	chr7	ENSG00000224940	Silent	c.1584G>A
2389	PRRT4	chr7	ENSG00000224940	Missense	c.386C>T
2390	PRUNE2	chr9	ENSG00000106772	3'UTR	c.*766dup
2391	PRUNE2	chr9	ENSG00000106772	3'UTR	c.*763T>C
2392	PRXL2A	chr10	ENSG00000122378	3'UTR	c.*551_*557del
2393	PSAP	chr10	ENSG00000197746	5'UTR	c.-76T>C
2394	PSD	chr10	ENSG00000059915	Missense	c.593G>C
2395	PSD3	chr8	ENSG00000156011	3'UTR	c.*6903A>G
2396	PSEN1	chr14	ENSG00000080815	Intron	c.869-87G>A
2397	PSMA1	chr11	ENSG00000129084	Silent	c.792A>G
2398	PSMA1	chr11	ENSG00000129084	5'UTR	c.-101A>G
2399	PSMA6	chr14	ENSG00000100902	Intron	c.172-62del
2400	PSMD2	chr3	ENSG00000175166	Intron	c.2298+13A>G
2401	PSMD4	chr1	ENSG00000159352	Intron	c.283-69C>T
2402	PSMD4	chr1	ENSG00000159352	Intron	c.438+65T>C
2403	PSMD5	chr9	ENSG00000095261	3'UTR	c.*763A>G
2404	PSMD7	chr16	ENSG00000103035	3'Flank	
2405	PTCD2	chr5	ENSG00000049883	Missense	c.391A>G
2406	PTCD2	chr5	ENSG00000049883	3'UTR	c.*700A>G
2407	PTCD3	chr2	ENSG00000132300	Missense	c.1363A>G
2408	PTCD3	chr2	ENSG00000132300	3'UTR	c.*4024_*4037del
2409	PTCD3	chr2	ENSG00000132300	3'UTR	c.*4290C>T
2410	PTCH2	chr1	ENSG00000117425	Frame shift Ins	c.198dup
2411	PTENP1	chr9	ENSG00000237984	RNA	n.2070del
2412	PTGER3	chr1	ENSG00000050628	Missense	c.601G>A
2413	PTPN13	chr4	ENSG00000163629	3'UTR	c.*574A>G
2414	PTPN22	chr1	ENSG00000134242	Splice site	c.828+2T>C
2415	PTPN3	chr9	ENSG00000070159	Missense	c.2509C>T
2416	PTPN4	chr2	ENSG00000088179	3'UTR	c.*2958C>G
2417	PTPRG	chr3	ENSG00000144724	Missense	c.4174A>G
2418	PTPRJ	chr11	ENSG00000149177	Missense	c.1420T>C
2419	PTPRJ	chr11	ENSG00000149177	Silent	c.3576T>C
2420	PTPRM	chr18	ENSG00000173482	Splice site	c.1753+2T>C
2421	PTPRM	chr18	ENSG00000173482	Splice region	c.3018C>A
2422	PTPRN2	chr7	ENSG00000155093	Intron	c.2257+2676A>G
2423	PTPRU	chr1	ENSG00000060656	5'Flank	
2424	PUF60	chr8	ENSG00000179950	5'Flank	
2425	PUS10	chr2	ENSG00000162927	3'UTR	c.*23T>C
2426	PWWP2A	chr5	ENSG00000170234	Missense	c.1750A>T
2427	PXDC1	chr6	ENSG00000168994	3'UTR	c.*537del
2428	PXDN	chr2	ENSG00000130508	3'UTR	c.*1732T>C
2429	PXDN	chr2	ENSG00000130508	Missense	c.2483A>G
2430	PXK	chr3	ENSG00000168297	Missense	c.1492T>C
2431	PYGO1	chr15	ENSG00000171016	3'UTR	c.*3062_*3063delinsTG
2432	QRICH2	chr17	ENSG00000129646	Frame shift del	c.4214del
2433	QRICH2	chr17	ENSG00000129646	Frame shift del	c.4214del
2434	QSER1	chr11	ENSG00000060749	3'UTR	c.*361del
2435	RAB14	chr9	ENSG00000119396	3'UTR	c.*238del
2436	RAB17	chr2	ENSG00000124839	Frame shift del	c.25del

Number	Gene name	Chromosome	Gene_ID	Mutation	Nucleotide substitution
2437	RAB17	chr2	ENSG00000124839	Frame shift del	c.25del
2438	RAB22A	chr20	ENSG00000124209	3'UTR	c.*2535dup
2439	RAB27A	chr15	ENSG00000069974	3'UTR	c.*773T>C
2440	RAB36	chr22	ENSG00000100228	3'UTR	c.*2052del
2441	RAB37	chr17	ENSG00000172794	3'UTR	c.*1066del
2442	RAB38	chr11	ENSG00000123892	Missense	c.182T>C
2443	RAB39B	chrX	ENSG00000155961	3'UTR	c.*1621del
2444	RAB3B	chr1	ENSG00000169213	3'UTR	c.*2581T>A
2445	RAB3GAP2	chr1	ENSG00000118873	Missense	c.3389C>T
2446	RAB3GAP2	chr1	ENSG00000118873	Silent	c.3045T>C
2447	RAB3GAP2	chr1	ENSG00000118873	Intron	c.2980+30T>C
2448	RAB3GAP2	chr1	ENSG00000118873	Missense	c.1408C>A
2449	RAB42	chr1	ENSG00000188060	3'UTR	c.*732G>A
2450	RAB43	chr3	ENSG00000172780	3'UTR	c.*3493T>C
2451	RAB7A	chr3	ENSG00000075785	Intron	c.-8-157A>G
2452	RABGEF1	chr7	ENSG00000154710	3'UTR	c.*2041G>A
2453	RABL2A	chr2	ENSG00000144134	3'UTR	c.*119T>C
2454	RABL6	chr9	ENSG00000196642	Frame shift del	c.1836del
2455	RABL6	chr9	ENSG00000196642	Frame shift del	c.1836del
2456	RAC2	chr22	ENSG00000128340	Intron	c.36-26G>A
2457	RAD50	chr5	ENSG00000113522	3'UTR	c.*1370del
2458	RAD51AP1	chr12	ENSG00000111247	Intron	c.210-641del
2459	RAD51D	chr17	ENSG00000185379	Intron	c.144+5444C>T
2460	RALGPS2	chr1	ENSG00000116191	3'UTR	c.*753del
2461	RALYL	chr8	ENSG00000184672	Intron	c.404+27T>C
2462	RAN	chr12	ENSG00000132341	3'UTR	c.*1003del
2463	RAP2B	chr3	ENSG00000181467	3'UTR	c.*2457del
2464	RAPGEF2	chr4	ENSG00000109756	3'UTR	c.*1017_*1018delinsGT
2465	RAPGEF3	chr12	ENSG00000079337	3'UTR	c.*2578T>C
2466	RAPGEF5	chr7	ENSG00000136237	3'UTR	c.*1363del
2467	RASAL2	chr1	ENSG00000075391	Frame shift del	c.1166del
2468	RASGRP2	chr11	ENSG00000068831	Missense	c.1303T>C
2469	RASSF2	chr20	ENSG00000101265	3'UTR	c.*2701G>A
2470	RASSF6	chr4	ENSG00000169435	3'UTR	c.*1980T>C
2471	RB1	chr13	ENSG00000139687	Frame shift del	c.1959del
2472	RB1	chr13	ENSG00000139687	Frame shift del	c.1959del
2473	RBBP6	chr16	ENSG00000122257	Frame shift del	c.4423del
2474	RBBP6	chr16	ENSG00000122257	Frame shift del	c.4423del
2475	RBBP9	chr20	ENSG00000089050	Intron	c.335-9del
2476	RBBP9	chr20	ENSG00000089050	Intron	c.142+48A>G
2477	RBL1	chr20	ENSG00000080839	Missense	c.2155A>G
2478	RBM12	chr20	ENSG00000244462	3'UTR	c.*2764del
2479	RBM28	chr7	ENSG00000106344	Splice site	c.809+2T>C
2480	RBM33	chr7	ENSG00000184863	3'UTR	c.*4525del
2481	RBM47	chr4	ENSG00000163694	3'UTR	c.*1277A>G
2482	RBP2	chr3	ENSG00000114113	3'Flank	
2483	RBPJ	chr4	ENSG00000168214	3'UTR	c.*581_*582del
2484	RBPJ	chr4	ENSG00000168214	3'UTR	c.*3867A>G
2485	RC3H1	chr1	ENSG00000135870	3'UTR	c.*6815del
2486	RCAN2	chr6	ENSG00000172348	Intron	c.226-76838del
2487	RCBTB2	chr13	ENSG00000136161	Intron	c.42+59T>C
2488	RCC1L	chr7	ENSG00000274523	3'UTR	c.*127C>T
2489	RCC2	chr1	ENSG00000179051	3'UTR	c.*2111C>G

Number	Gene name	Chromosome	Gene_ID	Mutation	Nucleotide substitution
2490	RCC2	chr1	ENSG00000179051	3'UTR	c.*1109C>T
2491	RCC2	chr1	ENSG00000179051	3'UTR	c.*177del
2492	RD3	chr1	ENSG00000198570	Missense	c.476T>C
2493	RDH10	chr8	ENSG00000121039	3'UTR	c.*1417del
2494	RECQL4	chr8	ENSG00000160957	Missense	c.837C>T
2495	RECQL5	chr17	ENSG00000108469	Missense	c.221T>C
2496	REG1B	chr2	ENSG00000172023	Splice region	c.65-3del
2497	REL	chr2	ENSG00000162924	Intron	c.11-14T>A
2498	RELB	chr19	ENSG00000104856	Intron	c.887-53A>G
2499	REST	chr4	ENSG00000084093	Missense	c.1435C>G
2500	RET	chr10	ENSG00000165731	Missense	c.539G>A
2501	REV3L	chr6	ENSG00000009413	3'UTR	c.*748A>G
2502	REV3L	chr6	ENSG00000009413	Missense	c.5467T>C
2503	REV3L	chr6	ENSG00000009413	Frame shift del	c.3435del
2504	REV3L	chr6	ENSG00000009413	Frame shift del	c.3435del
2505	REXO1	chr19	ENSG00000079313	Intron	c.158-2033T>C
2506	REXO2	chr11	ENSG00000076043	3'UTR	c.*61del
2507	RFC1	chr4	ENSG00000035928	Silent	c.2454T>C
2508	RFC4	chr3	ENSG00000163918	Intron	c.210+11A>T
2509	RFX1	chr19	ENSG00000132005	Silent	c.2055C>T
2510	RFX3	chr9	ENSG00000080298	3'UTR	c.*4671A>G
2511	RFX3	chr9	ENSG00000080298	3'UTR	c.*3912T>C
2512	RGL1	chr1	ENSG00000143344	5'UTR	c.-5T>C
2513	RGS12	chr4	ENSG00000159788	5'UTR	c.-350T>G
2514	RGS18	chr1	ENSG00000150681	3'UTR	c.*776C>T
2515	RGS21	chr1	ENSG00000253148	3'UTR	c.*490A>G
2516	RGS22	chr8	ENSG00000132554	Intron	c.2065-11del
2517	RGS5	chr1	ENSG00000143248	3'UTR	c.*4945G>A
2518	RGS5	chr1	ENSG00000143248	3'UTR	c.*2564del
2519	RGS6	chr14	ENSG00000182732	3'UTR	c.*1442del
2520	RGS9BP	chr19	ENSG00000186326	Frame shift Ins	c.613_614dup
2521	RHO	chr3	ENSG00000163914	Missense	c.89A>G
2522	RHOA	chr3	ENSG00000067560	Intron	c.409-699del
2523	RHOU	chr1	ENSG00000116574	3'UTR	c.*2171G>A
2524	RIC1	chr9	ENSG00000107036	Missense	c.235A>G
2525	RIC1	chr9	ENSG00000107036	Missense	c.2320A>G
2526	RIMBP2	chr12	ENSG00000060709	3'UTR	c.*1503A>C
2527	RIMKLB	chr12	ENSG00000166532	3'UTR	c.*311del
2528	RIMS2	chr8	ENSG00000176406	Intron	c.2851+892C>A
2529	RIN3	chr14	ENSG00000100599	3'UTR	c.*332A>G
2530	RIOK2	chr5	ENSG00000058729	3'UTR	c.*20A>G
2531	RIOK3	chr18	ENSG00000101782	Missense	c.176T>C
2532	RIPOR2	chr6	ENSG00000111913	3'UTR	c.*1136del
2533	RIPOR2	chr6	ENSG00000111913	3'UTR	c.*975del
2534	RIT2	chr18	ENSG00000152214	5'Flank	
2535	RMC1	chr18	ENSG00000141452	Intron	c.1007-52T>C
2536	RMDN1	chr8	ENSG00000176623	3'UTR	c.*232A>T
2537	RMDN1	chr8	ENSG00000176623	3'UTR	c.*1650del
2538	RNF103	chr2	ENSG00000239305	Intron	c.366+2269T>C
2539	RNF112	chr17	ENSG00000128482	Intron	c.933+31C>T
2540	RNF126	chr19	ENSG00000070423	Missense	c.743A>G
2541	RNF149	chr2	ENSG00000163162	3'UTR	c.*466del
2542	RNF17	chr13	ENSG00000132972	Missense	c.3385G>C

Number	Gene name	Chromosome	Gene_ID	Mutation	Nucleotide substitution
2543	RNF183	chr9	ENSG00000165188	3'Flank	
2544	RNF20	chr9	ENSG00000155827	Intron	c.973-26T>C
2545	RNF20	chr9	ENSG00000155827	Intron	c.2509-38T>C
2546	RNF207	chr1	ENSG00000158286	3'UTR	c.*1673A>T
2547	RNF208	chr9	ENSG00000212864	Missense	c.98T>C
2548	RNF213	chr17	ENSG00000173821	Silent	c.3960T>C
2549	RNF216	chr7	ENSG00000011275	Intron	c.1121+25del
2550	RNF216	chr7	ENSG00000011275	Intron	c.67+480del
2551	RNF217	chr6	ENSG00000146373	Frame shift del	c.387del
2552	RNF217	chr6	ENSG00000146373	Intron	c.882+14del
2553	RNF217	chr6	ENSG00000146373	3'UTR	c.*9214del
2554	RNF217-AS1	chr6	ENSG00000236548	RNA	n.2113C>T
2555	RNF26	chr11	ENSG00000173456	Missense	c.373A>G
2556	RNFT2	chr12	ENSG00000135119	3'UTR	c.*1782del
2557	RNPC3	chr1	ENSG00000185946	Intron	c.360-29A>G
2558	RNPEP	chr1	ENSG00000176393	Missense	c.925A>G
2559	RO60	chr1	ENSG00000116747	3'UTR	c.*726del
2560	ROR1	chr1	ENSG00000185483	Missense	c.434T>C
2561	ROR2	chr9	ENSG00000169071	Intron	c.1183+25del
2562	RORA	chr15	ENSG00000069667	3'UTR	c.*849del
2563	RP1L1	chr8	ENSG00000183638	3'UTR	c.*104T>C
2564	RP1L1	chr8	ENSG00000183638	Silent	c.1155G>T
2565	RPA2	chr1	ENSG00000117748	Intron	c.993-26C>T
2566	RPAIN	chr17	ENSG00000129197	Intron	c.314-618del
2567	RPAIN	chr17	ENSG00000129197	Intron	c.314-629T>C
2568	RPAP2	chr1	ENSG00000122484	Missense	c.253G>A
2569	RPH3A	chr12	ENSG00000089169	3'UTR	c.*1440del
2570	RPL22L1	chr3	ENSG00000163584	3'UTR	c.*934C>T
2571	RPL23	chr17	ENSG00000125691	3'UTR	c.*317_*318del
2572	RPL3	chr22	ENSG00000100316	Splice region	c.954T>C
2573	RPL31P57	chr17	ENSG00000256358	RNA	n.348del
2574	RPL34	chr4	ENSG00000109475	Intron	c.65+17A>T
2575	RPL37	chr5	ENSG00000145592	3'UTR	c.*4771del
2576	RPL37	chr5	ENSG00000145592	3'UTR	c.*860C>T
2577	RPP14	chr3	ENSG00000163684	3'UTR	c.*1509_*1510del
2578	RPP14	chr3	ENSG00000163684	3'UTR	c.*2149del
2579	RPS2	chr16	ENSG00000140988	In_Frame_Del	c.802_804del
2580	RPS6KA4	chr11	ENSG00000162302	Missense	c.1591C>A
2581	RPS6KB1	chr17	ENSG00000108443	Missense	c.287T>C
2582	RPS6KB1	chr17	ENSG00000108443	Missense	c.1177G>A
2583	RRBP1	chr20	ENSG00000125844	Missense	c.3666G>T
2584	RRP1B	chr21	ENSG00000160208	Intron	c.420-47C>T
2585	RSAD2	chr2	ENSG00000134321	3'UTR	c.*1212C>T
2586	RSBN1	chr1	ENSG00000081019	3'UTR	c.*2038del
2587	RSF1	chr11	ENSG00000048649	3'UTR	c.*4965A>G
2588	RSU1	chr10	ENSG00000148484	3'UTR	c.*916G>A
2589	RTKN2	chr10	ENSG00000182010	Intron	c.426+67G>C
2590	RTN4RL2	chr11	ENSG00000186907	Frame shift del	c.1118del
2591	RTN4RL2	chr11	ENSG00000186907	Frame shift del	c.1118del
2592	RUBCNL	chr13	ENSG00000102445	Missense	c.259C>G
2593	RUFY3	chr4	ENSG00000018189	Splice region	c.1557+6A>G
2594	RUFY3	chr4	ENSG00000018189	Intron	c.1650+65C>T
2595	RUFY4	chr2	ENSG00000188282	Missense	c.1511A>G

Number	Gene name	Chromosome	Gene_ID	Mutation	Nucleotide substitution
2596	RUNDC3B	chr7	ENSG00000105784	3'UTR	c.*1088del
2597	RUNX1T1	chr8	ENSG00000079102	3'UTR	c.*4770del
2598	RUNX1T1	chr8	ENSG00000079102	3'UTR	c.*1384del
2599	RUNX2	chr6	ENSG00000124813	3'UTR	c.*123T>A
2600	RUSC2	chr9	ENSG00000198853	Silent	c.3450C>T
2601	RUVBL1	chr3	ENSG00000175792	Silent	c.1023T>C
2602	RUVBL2	chr19	ENSG00000183207	3'UTR	c.*6A>G
2603	RYR1	chr19	ENSG00000196218	Missense	c.12883G>C
2604	RYR1	chr19	ENSG00000196218	Missense	c.14954T>C
2605	RYR2	chr1	ENSG00000198626	Missense	c.9205A>G
2606	S100A1	chr1	ENSG00000160678	Intron	c.77-43del
2607	S1PR3	chr9	ENSG00000213694	3'UTR	c.*3290del
2608	SAFB2	chr19	ENSG00000130254	Frame shift del	c.2471del
2609	SAFB2	chr19	ENSG00000130254	Frame shift del	c.2471del
2610	SAMD3	chr6	ENSG00000164483	3'UTR	c.*1292del
2611	SAMD5	chr6	ENSG00000203727	3'UTR	c.*1843del
2612	SAMD5	chr6	ENSG00000203727	3'UTR	c.*3840T>C
2613	SAP130	chr2	ENSG00000136715	3'UTR	c.*710A>G
2614	SAP18	chr13	ENSG00000150459	Missense	c.209T>C
2615	SASS6	chr1	ENSG00000156876	Missense	c.971T>C
2616	SASS6	chr1	ENSG00000156876	Silent	c.522T>C
2617	SAV1	chr14	ENSG00000151748	3'UTR	c.*352del
2618	SAYSD1	chr6	ENSG00000112167	3'UTR	c.*420del
2619	SBF1	chr22	ENSG00000100241	Intron	c.2843-44C>G
2620	SBSN	chr19	ENSG00000189001	Frame shift Ins	c.1260dup
2621	SCAF11	chr12	ENSG00000139218	Silent	c.1776T>C
2622	SCARA5	chr8	ENSG00000168079	Intron	c.1097-66G>T
2623	SCARB2	chr4	ENSG00000138760	3'UTR	c.*1716T>C
2624	SCD	chr10	ENSG00000099194	3'UTR	c.*824del
2625	SCFD1	chr14	ENSG00000092108	Intron	c.-55-3476del
2626	SCHIP1	chr3	ENSG00000151967	5'UTR	c.-32A>G
2627	SCIN	chr7	ENSG00000006747	Intron	c.1759+68del
2628	SCML1	chrX	ENSG00000047634	3'UTR	c.*301del
2629	SCN11A	chr3	ENSG00000168356	Missense	c.1245G>T
2630	SCN2A	chr2	ENSG00000136531	Missense	c.2050C>T
2631	SCN4A	chr17	ENSG00000007314	Missense	c.1375G>A
2632	SCNN1A	chr12	ENSG00000111319	3'UTR	c.*294A>G
2633	SCP2	chr1	ENSG00000116171	Intron	c.949+4803del
2634	SCPEP1	chr17	ENSG00000121064	Intron	c.658-56A>G
2635	SCUBE2	chr11	ENSG00000175356	Missense	c.2753A>G
2636	SCYL2	chr12	ENSG00000136021	Intron	c.969+10T>C
2637	SDC3	chr1	ENSG00000162512	3'UTR	c.*537C>A
2638	SDF4	chr1	ENSG00000078808	3'UTR	c.*526C>T
2639	SDHB	chr1	ENSG00000117118	Silent	c.180T>C
2640	SEC14L3	chr22	ENSG00000100012	3'UTR	c.*591A>G
2641	SEC16A	chr9	ENSG00000148396	Splice site	c.1341+2T>C
2642	SEC24A	chr5	ENSG00000113615	3'UTR	c.*2067del
2643	SECISBP2L	chr15	ENSG00000138593	Frame shift del	c.1545del
2644	SECISBP2L	chr15	ENSG00000138593	Frame shift del	c.1545del
2645	SEL1L	chr14	ENSG00000071537	Missense	c.895T>A
2646	SELENOS	chr15	ENSG00000131871	Missense	c.134A>G
2647	SELPLG	chr12	ENSG00000110876	Missense	c.808A>G
2648	SELPLG	chr12	ENSG00000110876	Missense	c.199T>C

Number	Gene name	Chromosome	Gene_ID	Mutation	Nucleotide substitution
2649	SEMA3C	chr7	ENSG00000075223	Silent	c.1152A>G
2650	SEMA3D	chr7	ENSG00000153993	Silent	c.1710T>C
2651	SEMA3D	chr7	ENSG00000153993	Splice region	c.1047-5del
2652	SEMA3E	chr7	ENSG00000170381	3'UTR	c.*2056C>T
2653	SEMA3E	chr7	ENSG00000170381	Frame shift del	c.761del
2654	SEMA3E	chr7	ENSG00000170381	Frame shift del	c.761del
2655	SEMA6A	chr5	ENSG00000092421	3'UTR	c.*423G>A
2656	SEMA6A	chr5	ENSG00000092421	Silent	c.2577G>A
2657	SEMA6C	chr1	ENSG00000143434	Missense	c.307T>C
2658	SENP1	chr12	ENSG00000079387	3'UTR	c.*2336del
2659	SENP6	chr6	ENSG00000112701	Intron	c.1406-57del
2660	SEPHS1	chr10	ENSG00000086475	Missense	c.1120G>A
2661	SEPTIN7	chr7	ENSG00000122545	Intron	c.61+10883del
2662	SEPTIN7	chr7	ENSG00000122545	Missense	c.1202G>A
2663	SERINC3	chr20	ENSG00000132824	3'UTR	c.*1415del
2664	SERPINA9	chr14	ENSG00000170054	Silent	c.933C>T
2665	SERPINC1	chr1	ENSG00000117601	Missense	c.847A>G
2666	SERTAD2	chr2	ENSG00000179833	Missense	c.440A>G
2667	SESTD1	chr2	ENSG00000187231	3'UTR	c.*1300del
2668	SET	chr9	ENSG00000119335	Splice region	c.378+3A>G
2669	SET	chr9	ENSG00000119335	3'UTR	c.*1069T>C
2670	SETBP1	chr18	ENSG00000152217	Nonsense	c.2836C>T
2671	SETD4	chr21	ENSG00000185917	Splice region	c.1+6T>C
2672	SETD7	chr4	ENSG00000145391	3'UTR	c.*823T>C
2673	SETDB1	chr1	ENSG00000143379	3'UTR	c.*327A>G
2674	SEZ6L	chr22	ENSG00000100095	Missense	c.575C>T
2675	SF3B3	chr16	ENSG00000189091	Splice region	c.2134-5del
2676	SF3B3	chr16	ENSG00000189091	Splice region	c.2134-8C>T
2677	SFMBT1	chr3	ENSG00000163935	Silent	c.777A>G
2678	SFN	chr1	ENSG00000175793	3'UTR	c.*405_*408del
2679	SFN	chr1	ENSG00000175793	3'UTR	c.*414_*415del
2680	SGK1	chr6	ENSG00000118515	Silent	c.111C>T
2681	SH2D3C	chr9	ENSG00000095370	5'UTR	c.-177A>G
2682	SH2D4A	chr8	ENSG00000104611	Intron	c.1273-19C>T
2683	SH3BP5	chr3	ENSG00000131370	3'UTR	c.*510del
2684	SH3D21	chr1	ENSG00000214193	Intron	c.162+35A>G
2685	SH3KBP1	chrX	ENSG00000147010	3'UTR	c.*2303del
2686	SH3PXD2B	chr5	ENSG00000174705	3'UTR	c.*962C>A
2687	SHC2	chr19	ENSG00000129946	Intron	c.774+18_774+19insT
2688	SHC4	chr15	ENSG00000185634	Missense	c.1294A>G
2689	SHC4	chr15	ENSG00000185634	Frame shift del	c.1043del
2690	SHC4	chr15	ENSG00000185634	Frame shift del	c.1043del
2691	SHC4	chr15	ENSG00000185634	Silent	c.690C>T
2692	SHE	chr1	ENSG00000169291	3'UTR	c.*445A>G
2693	SHISA5	chr3	ENSG00000164054	5'UTR	c.-310del
2694	SHISA6	chr17	ENSG00000188803	Missense	c.1366G>A
2695	SHISA7	chr19	ENSG00000187902	Frame shift del	c.1076del
2696	SHKBP1	chr19	ENSG00000160410	Missense	c.928G>A
2697	SHOC1	chr9	ENSG00000165181	Intron	c.2663+47A>G
2698	SHOC2	chr10	ENSG00000108061	3'UTR	c.*1775del
2699	SHROOM3	chr4	ENSG00000138771	Intron	c.323+19859A>G
2700	SHROOM3	chr4	ENSG00000138771	Silent	c.3681A>G
2701	SHROOM4	chrX	ENSG00000158352	Nonsense	c.3178G>T

Number	Gene name	Chromosome	Gene_ID	Mutation	Nucleotide substitution
2702	SHTN1	chr10	ENSG00000187164	3'UTR	c.*14del
2703	SI	chr3	ENSG00000090402	Splice region	c.4180-4del
2704	SIAE	chr11	ENSG00000110013	3'UTR	c.*1007del
2705	SIGLEC15	chr18	ENSG00000197046	Intron	c.906-52T>C
2706	SIGLEC7	chr19	ENSG00000168995	Frame shift del	c.979del
2707	SIGLEC7	chr19	ENSG00000168995	Frame shift del	c.979del
2708	SIK2	chr11	ENSG00000170145	Silent	c.780A>G
2709	SIK2	chr11	ENSG00000170145	3'UTR	c.*262_*263del
2710	SIPA1	chr11	ENSG00000213445	Intron	c.2637+32G>A
2711	SIRPB2	chr20	ENSG00000196209	Intron	c.859+414dup
2712	SIRT5	chr6	ENSG00000124523	3'UTR	c.*174A>T
2713	SIX1	chr14	ENSG00000126778	3'UTR	
2714	SIX5	chr19	ENSG00000177045	3'UTR	c.*259T>C
2715	SKP1	chr5	ENSG00000113558	Intron	c.97+2943del
2716	SLAMF1	chr1	ENSG00000117090	3'UTR	c.*2205A>G
2717	SLAMF8	chr1	ENSG00000158714	3'UTR	c.*762G>T
2718	SLC11A2	chr12	ENSG00000110911	3'UTR	c.*337A>T
2719	SLC12A2	chr5	ENSG00000064651	3'UTR	c.*2307_*2308del
2720	SLC12A4	chr16	ENSG00000124067	Missense	c.1673T>C
2721	SLC12A6	chr15	ENSG00000140199	Splice site	c.2889+1G>A
2722	SLC14A2	chr18	ENSG00000132874	Silent	c.1182C>T
2723	SLC15A2	chr3	ENSG00000163406	Intron	c.1571+51del
2724	SLC15A2	chr3	ENSG00000163406	3'UTR	c.*2268_*2269del
2725	SLC16A1	chr1	ENSG00000155380	Silent	c.1065A>G
2726	SLC16A2	chrX	ENSG00000147100	5'UTR	c.-29G>A
2727	SLC16A4	chr1	ENSG00000168679	Missense	c.533T>A
2728	SLC16A7	chr12	ENSG00000118596	3'UTR	c.*533_*534del
2729	SLC17A4	chr6	ENSG00000146039	Frame shift Ins	c.37dup
2730	SLC17A5	chr6	ENSG00000119899	3'UTR	c.*1366A>G
2731	SLC17A6	chr11	ENSG00000091664	3'UTR	c.*827del
2732	SLC18A2	chr10	ENSG00000165646	Intron	c.121+46G>A
2733	SLC18A2	chr10	ENSG00000165646	Intron	c.465-44del
2734	SLC19A1	chr21	ENSG00000173638	3'UTR	c.*2491dup
2735	SLC19A2	chr1	ENSG00000117479	5'Flank	
2736	SLC20A2	chr8	ENSG00000168575	Frame shift del	c.687del
2737	SLC20A2	chr8	ENSG00000168575	Frame shift del	c.687del
2738	SLC22A4	chr5	ENSG00000197208	Missense	c.928G>A
2739	SLC22A5	chr5	ENSG00000197375	Missense	c.1652T>C
2740	SLC23A3	chr2	ENSG00000213901	Splice site	c.418+2_418+3del
2741	SLC25A21	chr14	ENSG00000183032	3'UTR	c.*587del
2742	SLC25A27	chr6	ENSG00000153291	3'UTR	c.*518A>G
2743	SLC25A3	chr12	ENSG00000075415	Missense	c.714A>C
2744	SLC25A46	chr5	ENSG00000164209	3'UTR	c.*900A>T
2745	SLC26A10	chr12	ENSG00000135502	5'UTR	c.-207A>G
2746	SLC26A5	chr7	ENSG00000170615	Intron	c.2041+7388A>T
2747	SLC26A6	chr3	ENSG00000225697	Missense	c.1687C>A
2748	SLC26A9	chr1	ENSG00000174502	Missense	c.1763T>C
2749	SLC29A1	chr6	ENSG00000112759	Splice site	c.-51-2A>G
2750	SLC2A4RG	chr20	ENSG00000125520	Missense	c.1087G>A
2751	SLC30A10	chr1	ENSG00000196660	Missense	c.854T>C
2752	SLC30A7	chr1	ENSG00000162695	Missense	c.710T>C
2753	SLC35A3	chr1	ENSG00000117620	3'UTR	c.*1551del
2754	SLC35D1	chr1	ENSG00000116704	3'UTR	c.*4459T>C

Number	Gene name	Chromosome	Gene_ID	Mutation	Nucleotide substitution
2755	SLC35E1	chr19	ENSG00000127526	Intron	c.559-1742del
2756	SLC35E3	chr12	ENSG00000175782	3'UTR	c.*456dup
2757	SLC35F6	chr2	ENSG00000213699	Intron	c.323-23G>A
2758	SLC38A1	chr12	ENSG00000111371	Intron	c.388+91A>G
2759	SLC38A11	chr2	ENSG00000169507	3'UTR	c.*2122T>C
2760	SLC38A7	chr16	ENSG00000103042	Missense	c.284C>T
2761	SLC45A1	chr1	ENSG00000162426	Silent	c.1155C>T
2762	SLC45A4	chr8	ENSG00000022567	3'UTR	c.*3680del
2763	SLC4A2	chr7	ENSG00000164889	Intron	c.3619-36del
2764	SLC4A4	chr4	ENSG00000080493	Intron	c.2281-21del
2765	SLC4A9	chr5	ENSG00000113073	Intron	c.562-56T>C
2766	SLC50A1	chr1	ENSG00000169241	5'UTR	c.-118G>A
2767	SLC5A1	chr22	ENSG00000100170	Silent	c.1752T>C
2768	SLC5A10	chr17	ENSG00000154025	Intron	c.598-1145del
2769	SLC5A12	chr11	ENSG00000148942	3'UTR	c.*621del
2770	SLC5A9	chr1	ENSG00000117834	3'UTR	c.*589G>C
2771	SLC66A1L	chr3	ENSG00000174899	3'UTR	c.*442G>A
2772	SLC6A15	chr12	ENSG00000072041	3'UTR	c.*2075T>C
2773	SLC6A4	chr17	ENSG00000108576	3'UTR	c.*2172del
2774	SLC6A7	chr5	ENSG00000011083	3'UTR	c.*888C>T
2775	SLC7A1	chr13	ENSG00000139514	3'UTR	c.*1313G>A
2776	SLC7A11	chr4	ENSG00000151012	3'UTR	c.*2900T>A
2777	SLC7A13	chr8	ENSG00000164893	Frame shift del	c.372del
2778	SLC7A13	chr8	ENSG00000164893	Frame shift del	c.372del
2779	SLC7A9	chr19	ENSG00000021488	Nonsense	c.79C>T
2780	SLC8A1	chr2	ENSG00000183023	Intron	c.1954+26A>G
2781	SLC8A3	chr14	ENSG00000100678	3'UTR	c.*694A>G
2782	SLC8A3	chr14	ENSG00000100678	Intron	c.-85-16_-85-15del
2783	SLC9A1	chr1	ENSG00000090020	3'UTR	c.*827T>C
2784	SLC9A1	chr1	ENSG00000090020	Missense	c.359A>G
2785	SLC9A2	chr2	ENSG00000115616	3'UTR	c.*1320del
2786	SLC9B2	chr4	ENSG00000164038	3'UTR	c.*205del
2787	SLCO4A1	chr20	ENSG00000101187	5'Flank	
2788	SLFN13	chr17	ENSG00000154760	3'UTR	c.*2864del
2789	SLIT1	chr10	ENSG00000187122	3'UTR	c.*211del
2790	SLIT2	chr4	ENSG00000145147	Intron	c.2429+49del
2791	SLITRK1	chr13	ENSG00000178235	5'UTR	c.-246_-245del
2792	SLITRK2	chrX	ENSG00000185985	5'UTR	c.-430_-429del
2793	SLMAP	chr3	ENSG00000163681	Intron	c.457-34T>C
2794	SMAD2	chr18	ENSG00000175387	3'UTR	c.*8896del
2795	SMAD7	chr18	ENSG00000101665	Frame shift del	c.626del
2796	SMAD7	chr18	ENSG00000101665	Frame shift del	c.626del
2797	SMAD9	chr13	ENSG00000120693	3'UTR	c.*3046del
2798	SMARCA2	chr9	ENSG00000080503	Intron	c.9+545del
2799	SMARCA4	chr19	ENSG00000127616	Silent	c.2934A>G
2800	SMARCA5	chr4	ENSG00000153147	3'UTR	c.*1873C>T
2801	SMARCA5	chr4	ENSG00000153147	3'UTR	c.*3307del
2802	SMARCC1	chr3	ENSG00000173473	3'UTR	c.*2808C>T
2803	SMARCC1	chr3	ENSG00000173473	3'UTR	c.*977del
2804	SMARCC2	chr12	ENSG00000139613	Missense	c.2735T>C
2805	SMARCD3	chr7	ENSG00000082014	Intron	c.778-9T>C
2806	SMARCE1	chr17	ENSG00000073584	3'UTR	c.*1142A>G
2807	SMC5	chr9	ENSG00000198887	Silent	c.1797A>G

Number	Gene name	Chromosome	Gene_ID	Mutation	Nucleotide substitution
2808	SMC6	chr2	ENSG00000163029	Nonsense	c.865C>T
2809	SMCHD1	chr18	ENSG00000101596	Silent	c.3567T>C
2810	SMCHD1	chr18	ENSG00000101596	Intron	c.4434+17del
2811	SMCR8	chr17	ENSG00000176994	Silent	c.1539C>T
2812	SMG1	chr16	ENSG00000157106	3'UTR	c.*1820del
2813	SMG5	chr1	ENSG00000198952	3'UTR	c.*1355del
2814	SMG7	chr1	ENSG00000116698	Splice site	c.1415+2T>C
2815	SMG8	chr17	ENSG00000167447	Missense	c.1426T>A
2816	SMIM13	chr6	ENSG00000224531	3'UTR	c.*3699T>A
2817	SMIM29	chr6	ENSG00000186577	Missense	c.131A>G
2818	SMOC2	chr6	ENSG00000112562	Intron	c.1357-10dup
2819	SMPD2	chr6	ENSG00000135587	Intron	c.625-30C>T
2820	SMU1	chr9	ENSG00000122692	3'UTR	c.*2452A>G
2821	SMU1	chr9	ENSG00000122692	3'UTR	c.*924A>G
2822	SMYD3	chr1	ENSG00000185420	Intron	c.1185+47del
2823	SMYD4	chr17	ENSG00000186532	Intron	c.1537+26T>C
2824	SNAPC1	chr14	ENSG00000023608	3'UTR	c.*743del
2825	SNCB	chr5	ENSG00000074317	3'UTR	c.*693C>T
2826	SNRNP200	chr2	ENSG00000144028	Intron	c.2421+52T>C
2827	SNRNP48	chr6	ENSG00000168566	3'UTR	c.*984C>T
2828	SNRPF	chr12	ENSG00000139343	Intron	c.195-54A>G
2829	SNTA1	chr20	ENSG00000101400	Missense	c.657G>A
2830	SNTG1	chr8	ENSG00000147481	Intron	c.550-29del
2831	SNTN	chr3	ENSG00000188817	3'UTR	c.*568_*569dup
2832	SNUPN	chr15	ENSG00000169371	Intron	c.-6+1000del
2833	SOBP	chr6	ENSG00000112320	Missense	c.2057A>G
2834	SOCS1	chr16	ENSG00000185338	5'UTR	c.-28C>T
2835	SOD2	chr6	ENSG00000112096	3'UTR	c.*1312T>C
2836	SOD2	chr6	ENSG00000285441	5'UTR	c.-996dup
2837	SOGA1	chr20	ENSG00000149639	Intron	c.1135-37T>C
2838	SOGA3	chr6	ENSG00000214338	3'UTR	c.*209A>G
2839	SOGA3	chr6	ENSG00000214338	Missense	c.1178A>G
2840	SON	chr21	ENSG00000159140	Missense	c.5957G>A
2841	SON	chr21	ENSG00000159140	3'UTR	c.*78T>C
2842	SORBS1	chr10	ENSG00000095637	Silent	c.870C>A
2843	SORBS2	chr4	ENSG00000154556	Intron	c.379+77T>C
2844	SORCS1	chr10	ENSG00000108018	Nonsense	c.959G>A
2845	SORCS2	chr4	ENSG00000184985	3'UTR	c.*1186del
2846	SORCS3	chr10	ENSG00000156395	Missense	c.2033G>A
2847	SORL1	chr11	ENSG00000137642	Silent	c.951C>T
2848	SORL1	chr11	ENSG00000137642	3'UTR	c.*1071del
2849	SOX13	chr1	ENSG00000143842	3'UTR	c.*1564A>G
2850	SOX2	chr3	ENSG00000181449	Silent	c.789G>A
2851	SP6	chr17	ENSG00000189120	Frame shift del	c.461del
2852	SP6	chr17	ENSG00000189120	Frame shift del	c.461del
2853	SP8	chr7	ENSG00000164651	Frame shift del	c.1443del
2854	SP8	chr7	ENSG00000164651	Frame shift del	c.1443del
2855	SP9	chr2	ENSG00000217236	Intron	c.22-16A>C
2856	SPAG5	chr17	ENSG00000076382	Missense	c.1933T>C
2857	SPATA1	chr1	ENSG00000122432	Missense	c.865A>G
2858	SPATA6	chr1	ENSG00000132122	3'UTR	c.*876del
2859	SPCS2P4	chr1	ENSG00000228589	RNA	n.12A>G
2860	SPCS3	chr4	ENSG00000129128	3'UTR	c.*730del

Number	Gene name	Chromosome	Gene_ID	Mutation	Nucleotide substitution
2861	SPDYE1	chr7	ENSG00000136206	Missense	c.956A>G
2862	SPIDR	chr8	ENSG00000164808	Intron	c.1545-148C>T
2863	SPIN1	chr9	ENSG00000106723	3'UTR	c.*332G>A
2864	SPIN3	chrX	ENSG00000204271	5'Flank	
2865	SPIN4	chrX	ENSG00000186767	3'UTR	c.*1460del
2866	SPIN4	chrX	ENSG00000186767	3'UTR	c.*1121del
2867	SPIN4	chrX	ENSG00000186767	5'UTR	c.-478A>G
2868	SPOCK1	chr5	ENSG00000152377	Intron	c.404-29del
2869	SPOCK1	chr5	ENSG00000152377	Intron	c.187-11del
2870	SPOP	chr17	ENSG00000121067	Missense	c.146G>A
2871	SPPL2A	chr15	ENSG00000138600	3'UTR	c.*296del
2872	SPPL2A	chr15	ENSG00000138600	Missense	c.35C>T
2873	SPRED1	chr15	ENSG00000166068	3'UTR	c.*1483del
2874	SPRED2	chr2	ENSG00000198369	5'UTR	c.-110del
2875	SPSB1	chr1	ENSG00000171621	Missense	c.107G>A
2876	SPSB2	chr12	ENSG00000111671	Missense	c.377G>A
2877	SPSB4	chr3	ENSG00000175093	Intron	c.695-14957del
2878	SPTA1	chr1	ENSG00000163554	Missense	c.1016C>A
2879	SPTAN1	chr9	ENSG00000197694	Missense	c.2963T>C
2880	SPTBN1	chr2	ENSG00000115306	3'UTR	c.*2179A>G
2881	SPTBN4	chr19	ENSG00000160460	Intron	c.4360-27C>T
2882	SPTBN5	chr15	ENSG00000137877	Splice region	c.10836-8_10836-7insT
2883	SPTBN5	chr15	ENSG00000137877	Intron	c.10836-34A>G
2884	SPTBN5	chr15	ENSG00000137877	Missense	c.6599C>T
2885	SPTY2D1	chr11	ENSG00000179119	3'UTR	c.*2992del
2886	SPZ1	chr5	ENSG00000164299	Silent	c.1266A>G
2887	SRCAP	chr16	ENSG00000080603	Intron	c.857-51C>A
2888	SRD5A3	chr4	ENSG00000128039	3'UTR	c.*2328del
2889	SRFBP1	chr5	ENSG00000151304	3'UTR	c.*52del
2890	SRGAP1	chr12	ENSG00000196935	3'UTR	c.*3715del
2891	SRGAP2	chr1	ENSG00000266028	Silent	c.2172C>T
2892	SRP14	chr15	ENSG00000140319	Intron	c.211-258dup
2893	SRP19	chr5	ENSG00000153037	3'UTR	c.*30A>G
2894	SRPK2	chr7	ENSG00000135250	Intron	c.1883-10del
2895	SRPRA	chr11	ENSG00000182934	Missense	c.1202G>A
2896	SRRM1	chr1	ENSG00000133226	Silent	c.568A>C
2897	SRRM1	chr1	ENSG00000133226	3'UTR	c.*343dup
2898	SRRM4	chr12	ENSG00000139767	3'UTR	c.*5127del
2899	SRSF11	chr1	ENSG00000116754	3'UTR	c.*1058C>T
2900	SRSF12	chr6	ENSG00000154548	3'UTR	c.*1498T>G
2901	SSBP4	chr19	ENSG00000130511	Intron	c.1020+43del
2902	SSC5D	chr19	ENSG00000179954	Missense	c.4186T>C
2903	SSH1	chr12	ENSG00000084112	3'UTR	c.*4090A>G
2904	SSH2	chr17	ENSG00000141298	Missense	c.1232A>G
2905	SSPO	chr7	ENSG00000197558	Intron	c.7279+39del
2906	SSPO	chr7	ENSG00000197558	Missense	c.8770G>A
2907	SSR1	chr6	ENSG00000124783	3'UTR	c.*7983dup
2908	SSRP1	chr11	ENSG00000149136	3'UTR	c.*14T>C
2909	SSX2IP	chr1	ENSG00000117155	3'UTR	c.*560T>C
2910	SSX3	chrX	ENSG00000165584	Missense	c.464C>G
2911	ST3GAL1	chr8	ENSG00000008513	3'UTR	c.*2518G>A
2912	ST5	chr11	ENSG00000166444	5'UTR	c.-132T>C
2913	ST6GALNAC3	chr1	ENSG00000184005	Intron	c.18+67950del

Number	Gene name	Chromosome	Gene_ID	Mutation	Nucleotide substitution
2914	ST7L	chr1	ENSG00000007341	Missense	c.7C>A
2915	ST8SIA4	chr5	ENSG00000113532	3'UTR	c.*1955del
2916	STAB2	chr12	ENSG00000136011	Intron	c.2259+11A>G
2917	STAM	chr10	ENSG00000136738	5'UTR	c.-174T>C
2918	STARD8	chrX	ENSG00000130052	Intron	c.-7-1142C>A
2919	STAT3	chr17	ENSG00000168610	3'UTR	c.*1421C>T
2920	STEAP1B	chr7	ENSG00000105889	3'UTR	c.*66A>G
2921	STEAP3	chr2	ENSG00000115107	Missense	c.554T>C
2922	STIM2	chr4	ENSG00000109689	3'UTR	c.*1270del
2923	STIP1	chr11	ENSG00000168439	5'UTR	c.-92T>C
2924	STK26	chrX	ENSG00000134602	Splice region	c.330+7A>G
2925	STK26	chrX	ENSG00000134602	3'UTR	c.*409del
2926	STK32A	chr5	ENSG00000169302	3'UTR	c.*2857del
2927	STK4	chr20	ENSG00000101109	Intron	c.245+10T>C
2928	STK40	chr1	ENSG00000196182	Intron	c.624-30A>G
2929	STRADA	chr17	ENSG00000266173	Intron	c.-51-91G>A
2930	STRBP	chr9	ENSG00000165209	anslation_Start_S	c.1A>G
2931	STUM	chr1	ENSG00000203685	3'UTR	c.*2013A>G
2932	STX12	chr1	ENSG00000117758	3'UTR	c.*1745del
2933	STX1B	chr16	ENSG00000099365	3'UTR	c.*1684T>C
2934	STXBP4	chr17	ENSG00000166263	3'UTR	c.*2567T>C
2935	STXBP5L	chr3	ENSG00000145087	Intron	c.287+26C>A
2936	SUCO	chr1	ENSG00000094975	Missense	c.2999T>C
2937	SUFU	chr10	ENSG00000107882	Intron	c.756+43A>G
2938	SUMO1	chr2	ENSG00000116030	3'UTR	c.*363del
2939	SUPT3H	chr6	ENSG00000196284	Intron	c.101+36922del
2940	SUPT6H	chr17	ENSG00000109111	Intron	c.2838+17A>C
2941	SUPT6H	chr17	ENSG00000109111	Missense	c.4216G>A
2942	SUPV3L1	chr10	ENSG00000156502	Missense	c.812A>G
2943	SURF4	chr9	ENSG00000148248	3'UTR	c.*401T>A
2944	SUSD1	chr9	ENSG00000106868	Missense	c.687G>A
2945	SUSD3	chr9	ENSG00000157303	5'Flank	
2946	SUSD6	chr14	ENSG00000100647	3'UTR	c.*1840T>C
2947	SUZ12	chr17	ENSG00000178691	3'UTR	c.*738T>C
2948	SV2A	chr1	ENSG00000159164	Missense	c.1958C>A
2949	SV2C	chr5	ENSG00000122012	3'UTR	c.*443_*444del
2950	SV2C	chr5	ENSG00000122012	3'UTR	c.*8388del
2951	SWAP70	chr11	ENSG00000133789	3'UTR	c.*1342A>G
2952	SYAP1	chrX	ENSG00000169895	3'UTR	c.*4347del
2953	SYBU	chr8	ENSG00000147642	Intron	c.-128-26055del
2954	SYCE1	chr10	ENSG00000171772	3'UTR	c.*363del
2955	SYCP3	chr12	ENSG00000139351	Splice site	c.658-1G>A
2956	SYMPK	chr19	ENSG00000125755	3'UTR	c.*137_*138del
2957	SYNCRIP	chr6	ENSG00000135316	3'UTR	c.*1684A>G
2958	SYNE1	chr6	ENSG00000131018	Missense	c.23389C>T
2959	SYNPO	chr5	ENSG00000171992	Frame shift Ins	c.2178_2181dup
2960	SYNPO2	chr4	ENSG00000172403	3'UTR	c.*3050del
2961	SYPL1	chr7	ENSG00000008282	Intron	c.645+176A>G
2962	SYPL2	chr1	ENSG00000143028	Intron	c.54+19G>T
2963	SYPL2	chr1	ENSG00000143028	3'UTR	c.*1379A>G
2964	SYT1	chr12	ENSG00000067715	3'UTR	c.*1704T>C
2965	SYT13	chr11	ENSG00000019505	3'UTR	c.*3745A>G
2966	SYT13	chr11	ENSG00000019505	Splice region	c.410-7T>A

Number	Gene name	Chromosome	Gene_ID	Mutation	Nucleotide substitution
2967	SYT17	chr16	ENSG00000103528	3'UTR	c.*894_*902del
2968	TAB1	chr22	ENSG00000100324	Silent	c.1398C>T
2969	TAB3	chrX	ENSG00000157625	Frame shift del	c.423del
2970	TAF1B	chr2	ENSG00000115750	Frame shift del	c.1411del
2971	TAF1B	chr2	ENSG00000115750	Frame shift del	c.1411del
2972	TAF2	chr8	ENSG00000064313	3'UTR	c.*645C>T
2973	TAF2	chr8	ENSG00000064313	Silent	c.1401A>G
2974	TAF6L	chr11	ENSG00000162227	Nonsense	c.313C>T
2975	TAF6L	chr11	ENSG00000162227	Missense	c.1798A>G
2976	TAF9B	chrX	ENSG00000187325	3'UTR	c.*427del
2977	TAGLN	chr11	ENSG00000149591	3'UTR	c.*417_*419del
2978	TANC1	chr2	ENSG00000115183	3'UTR	c.*106del
2979	TANC2	chr17	ENSG00000170921	Missense	c.3890G>A
2980	TANK	chr2	ENSG00000136560	Intron	c.328-4256del
2981	TAPT1	chr4	ENSG00000169762	3'UTR	c.*881del
2982	TARS	chr5	ENSG00000113407	3'UTR	c.*370del
2983	TARSL2	chr15	ENSG00000185418	Intron	c.1074+20del
2984	TATDN2	chr3	ENSG00000157014	Missense	c.88T>A
2985	TAX1BP3	chr17	ENSG00000213977	3'UTR	c.*317T>C
2986	TBC1D12	chr10	ENSG00000108239	3'UTR	c.*1555del
2987	TBC1D14	chr4	ENSG00000132405	3'UTR	c.*1020T>C
2988	TBC1D22B	chr6	ENSG00000065491	3'UTR	c.*363del
2989	TBC1D5	chr3	ENSG00000131374	Intron	c.752+34del
2990	TBC1D9B	chr5	ENSG00000197226	Missense	c.2570A>G
2991	TBCCD1	chr3	ENSG00000113838	5'UTR	c.-155A>G
2992	TBCK	chr4	ENSG00000145348	3'UTR	c.*1551del
2993	TBKBP1	chr17	ENSG00000198933	Missense	c.890C>T
2994	TBL1X	chrX	ENSG00000101849	3'UTR	c.*3188del
2995	TBL3	chr16	ENSG00000183751	Missense	c.1820G>A
2996	TBX2	chr17	ENSG00000121068	3'Flank	
2997	TCF19	chr6	ENSG00000137310	3'UTR	c.*789A>G
2998	TCF21	chr6	ENSG00000118526	3'UTR	c.*1370A>G
2999	TCF23	chr2	ENSG00000163792	Splice site	c.465+2T>C
3000	TCF25	chr16	ENSG00000141002	Missense	c.1637T>C
3001	TCF7	chr5	ENSG00000081059	Frame shift Ins	c.463dup
3002	TCF7L2	chr10	ENSG00000148737	3'UTR	c.*860del
3003	TCL1A	chr14	ENSG00000100721	Intron	c.121-386del
3004	TCL1B	chr14	ENSG00000213231	Intron	c.*15+46T>C
3005	TCTA	chr3	ENSG00000145022	3'UTR	c.*662A>T
3006	TDGF1P3	chrX	ENSG00000225366	RNA	n.397del
3007	TDRD7	chr9	ENSG00000196116	Intron	c.1629+24A>G
3008	TDRD7	chr9	ENSG00000196116	Missense	c.2488C>T
3009	TEAD2	chr19	ENSG00000074219	Frame shift del	c.883del
3010	TEAD2	chr19	ENSG00000074219	Frame shift del	c.883del
3011	TEAD3	chr6	ENSG00000007866	Missense	c.253C>T
3012	TENM3	chr4	ENSG00000218336	Silent	c.6A>G
3013	TENT5A	chr6	ENSG00000112773	Frame shift del	c.517del
3014	TERF2	chr16	ENSG00000132604	3'UTR	c.*1056del
3015	TESK2	chr1	ENSG00000070759	3'UTR	c.*564T>C
3016	TESK2	chr1	ENSG00000070759	Missense	c.1343C>T
3017	TET1	chr10	ENSG00000138336	Silent	c.477A>G
3018	TET2	chr4	ENSG00000168769	Silent	c.1548A>G
3019	TET2	chr4	ENSG00000168769	3'UTR	c.*4512_*4514del

Number	Gene name	Chromosome	Gene_ID	Mutation	Nucleotide substitution
3020	TET3	chr2	ENSG00000187605	Missense	c.4412A>G
3021	TFAP2B	chr6	ENSG00000008196	3'UTR	c.*135_*136inv
3022	TFCP2L1	chr2	ENSG00000115112	3'UTR	c.*5599G>A
3023	TFDP2	chr3	ENSG00000114126	Intron	c.-203-777T>A
3024	TFE3	chrX	ENSG00000068323	5'UTR	c.-79del
3025	TFEC	chr7	ENSG00000105967	Splice site	c.382+2T>C
3026	TFRC	chr3	ENSG00000072274	Intron	c.1677+91C>A
3027	TGFBI	chr5	ENSG00000120708	5'Flank	
3028	TGFBR1	chr9	ENSG00000106799	3'UTR	c.*4804del
3029	TGIF1	chr18	ENSG00000177426	3'UTR	c.*561del
3030	TGIF2	chr20	ENSG00000118707	3'UTR	c.*730del
3031	TGIF2	chr20	ENSG00000118707	3'UTR	c.*917G>T
3032	TGIF2	chr20	ENSG00000118707	3'UTR	c.*2510del
3033	TGM2	chr20	ENSG00000198959	Missense	c.1952G>A
3034	TGM2	chr20	ENSG00000198959	Missense	c.1810G>A
3035	TGM5	chr15	ENSG00000104055	Missense	c.1829T>A
3036	THAP1	chr8	ENSG00000131931	3'UTR	c.*280del
3037	THAP10	chr15	ENSG00000129028	3'UTR	c.*219del
3038	THBS1	chr15	ENSG00000137801	3'UTR	c.*3429C>T
3039	THBS1	chr15	ENSG00000137801	3'UTR	c.*3464del
3040	THBS3	chr1	ENSG00000169231	Nonsense	c.778C>T
3041	THEM5	chr1	ENSG00000196407	Missense	c.584C>A
3042	THOC1	chr18	ENSG00000079134	Missense	c.268G>A
3043	THOP1	chr19	ENSG00000172009	5'UTR	c.-5C>A
3044	THSD4	chr15	ENSG00000187720	Missense	c.256C>T
3045	THUMPD1	chr16	ENSG00000066654	Intron	c.407-356_407-355del
3046	TIA1	chr2	ENSG00000116001	3'UTR	c.*1200G>T
3047	TIA1	chr2	ENSG00000116001	3'UTR	c.*402del
3048	TICAM1	chr19	ENSG00000127666	Silent	c.1041G>A
3049	TICRR	chr15	ENSG00000140534	Missense	c.5412G>T
3050	TIGD6	chr5	ENSG00000164296	Missense	c.127A>G
3051	TIMM44	chr19	ENSG00000104980	Missense	c.714C>A
3052	TINAG	chr6	ENSG00000137251	anslation_Start_S	c.2T>C
3053	TIPARP	chr3	ENSG00000163659	3'UTR	c.*1596del
3054	TIPIN	chr15	ENSG00000075131	Intron	c.212+79del
3055	TJAP1	chr6	ENSG00000137221	Frame shift Ins	c.1598dup
3056	TJP2	chr9	ENSG00000119139	Missense	c.2065C>T
3057	TKT	chr3	ENSG00000163931	3'UTR	c.*444A>G
3058	TLCD5	chr11	ENSG00000181264	3'UTR	c.*2546del
3059	TLK1	chr2	ENSG00000198586	3'UTR	c.*2747del
3060	TLL1	chr4	ENSG00000038295	Intron	c.169+45780A>G
3061	TLN2	chr15	ENSG00000171914	Intron	c.-36-9071G>C
3062	TLN2	chr15	ENSG00000171914	Missense	c.676G>A
3063	TLR2	chr4	ENSG00000137462	5'UTR	c.-827A>G
3064	TM2D1	chr1	ENSG00000162604	Intron	c.439+635A>G
3065	TM9SF2	chr13	ENSG00000125304	Intron	c.717-16del
3066	TMCC1	chr3	ENSG00000172765	Silent	c.1611C>T
3067	TMCC3	chr12	ENSG00000057704	5'UTR	c.-5C>T
3068	TMCO4	chr1	ENSG00000162542	Splice region	c.383-3G>A
3069	TMCO4	chr1	ENSG00000162542	Missense	c.89C>T
3070	TMED10	chr14	ENSG00000170348	3'UTR	c.*2315C>T
3071	TMEFF1	chr9	ENSG00000241697	Intron	c.464-28T>C
3072	TMEFF2	chr2	ENSG00000144339	3'UTR	c.*1978del

Number	Gene name	Chromosome	Gene_ID	Mutation	Nucleotide substitution
3073	TMEM123	chr11	ENSG00000152558	3'UTR	c.*1027A>G
3074	TMEM127	chr2	ENSG00000135956	5'Flank	
3075	TMEM135	chr11	ENSG00000166575	Intron	c.551+54del
3076	TMEM135	chr11	ENSG00000166575	3'UTR	c.*622del
3077	TMEM145	chr19	ENSG00000167619	Missense	c.938C>T
3078	TMEM145	chr19	ENSG00000167619	3'UTR	c.*13del
3079	TMEM154	chr4	ENSG00000170006	3'UTR	c.*5267del
3080	TMEM154	chr4	ENSG00000170006	3'UTR	c.*1551T>C
3081	TMEM171	chr5	ENSG00000157111	Missense	c.91G>A
3082	TMEM18	chr2	ENSG00000151353	3'UTR	c.*1268_*1269del
3083	TMEM184B	chr22	ENSG00000198792	3'UTR	c.*1940del
3084	TMEM184B	chr22	ENSG00000198792	3'UTR	c.*1939T>A
3085	TMEM192	chr4	ENSG00000170088	3'UTR	c.*1811del
3086	TMEM199	chr17	ENSG00000244045	3'UTR	c.*2298A>G
3087	TMEM201	chr1	ENSG00000188807	Missense	c.68C>T
3088	TMEM201	chr1	ENSG00000188807	Intron	c.1903+14G>T
3089	TMEM202	chr15	ENSG00000187806	Silent	c.276C>T
3090	TMEM214	chr2	ENSG00000119777	Intron	c.637+51del
3091	TMEM222	chr1	ENSG00000186501	3'UTR	c.*11del
3092	TMEM245	chr9	ENSG00000106771	3'UTR	c.*1720del
3093	TMEM247	chr2	ENSG00000284701	Missense	c.571A>G
3094	TMEM259	chr19	ENSG00000182087	Missense	c.625T>C
3095	TMEM26	chr10	ENSG00000196932	Missense	c.106T>C
3096	TMEM35B	chr1	ENSG00000243749	3'UTR	c.*345del
3097	TMEM38B	chr9	ENSG00000095209	3'UTR	c.*1418del
3098	TMEM38B	chr9	ENSG00000095209	3'UTR	c.*2383del
3099	TMEM51	chr1	ENSG00000171729	3'UTR	c.*537C>T
3100	TMEM62	chr15	ENSG00000137842	Intron	c.431-25T>A
3101	TMEM62	chr15	ENSG00000137842	Intron	c.1605+38C>T
3102	TMEM64	chr8	ENSG00000180694	3'UTR	c.*82T>G
3103	TMEM87B	chr2	ENSG00000153214	3'UTR	c.*1530del
3104	TMEM8A	chr16	ENSG00000129925	Splice region	c.860+3A>G
3105	TMEM94	chr17	ENSG00000177728	Silent	c.3348G>A
3106	TMEM9B	chr11	ENSG00000175348	3'UTR	c.*501A>G
3107	TMPO	chr12	ENSG00000120802	3'UTR	c.*1661del
3108	TMPRSS12	chr12	ENSG00000186452	Missense	c.316G>A
3109	TMPRSS13	chr11	ENSG00000137747	Intron	c.1524+11C>A
3110	TMX2	chr11	ENSG00000213593	Missense	c.785A>G
3111	TNFAIP1	chr17	ENSG00000109079	3'UTR	c.*708del
3112	TNFAIP2	chr14	ENSG00000185215	Missense	c.1339C>A
3113	TNFRSF10B	chr8	ENSG00000120889	anslation_Start_S	c.2T>C
3114	TNFRSF11A	chr18	ENSG00000141655	3'UTR	c.*197C>T
3115	TNFRSF17	chr16	ENSG00000048462	Silent	c.327T>C
3116	TNFSF10	chr3	ENSG00000121858	5'UTR	c.-7C>T
3117	TNFSF4	chr1	ENSG00000117586	3'UTR	c.*784G>T
3118	TNKS2	chr10	ENSG00000107854	Intron	c.729-67T>C
3119	TNN	chr1	ENSG00000120332	Missense	c.587A>G
3120	TNNT2	chr1	ENSG00000118194	Intron	c.97+537G>T
3121	TNRC6A	chr16	ENSG00000090905	Missense	c.1339A>G
3122	TNRC6A	chr16	ENSG00000090905	3'UTR	c.*833del
3123	TNRC6B	chr22	ENSG00000100354	3'UTR	c.*4619del
3124	TNS1	chr2	ENSG00000079308	3'UTR	c.*872C>T
3125	TNS3	chr7	ENSG00000136205	3'UTR	c.*15C>A

Number	Gene name	Chromosome	Gene_ID	Mutation	Nucleotide substitution
3126	TOMM20	chr1	ENSG00000173726	3'UTR	c.*351del
3127	TOMM40L	chr1	ENSG00000158882	Missense	c.80C>A
3128	TOMM40L	chr1	ENSG00000158882	3'UTR	c.*1609T>C
3129	TOP1	chr20	ENSG00000198900	Splice region	c.508-8C>T
3130	TOP3A	chr17	ENSG00000177302	Splice region	c.391-7A>G
3131	TOPBP1	chr3	ENSG00000163781	Missense	c.2875A>G
3132	TOPBP1	chr3	ENSG00000163781	Frame shift del	c.72del
3133	TOPBP1	chr3	ENSG00000163781	Frame shift del	c.72del
3134	TOPORS	chr9	ENSG00000197579	3'UTR	c.*638A>T
3135	TOR1AIP1	chr1	ENSG00000143337	3'UTR	
3136	TP53	chr17	ENSG00000141510	Nonsense	c.586C>T
3137	TP53BP1	chr15	ENSG00000067369	Silent	c.3165C>A
3138	TP53I3	chr2	ENSG00000115129	5'UTR	c.-174G>T
3139	TP53INP1	chr8	ENSG00000164938	3'UTR	c.*1638T>C
3140	TP63	chr3	ENSG00000073282	Missense	c.415G>A
3141	TPD52	chr8	ENSG00000076554	5'UTR	c.-153G>C
3142	TPMT	chr6	ENSG00000137364	Intron	c.580+14del
3143	TPRG1	chr3	ENSG00000188001	3'UTR	c.*3871A>G
3144	TPTE	chr21	ENSG00000274391	Intron	c.681+49G>A
3145	TRAF1	chr9	ENSG00000056558	5'UTR	c.-167T>C
3146	TRAJ61	chr14	ENSG00000211831	3'Flank	
3147	TRAK1	chr3	ENSG00000182606	3'UTR	c.*1040del
3148	TRANK1	chr3	ENSG00000168016	Missense	c.4930T>C
3149	TRAPPC10	chr21	ENSG00000160218	3'UTR	c.*315T>C
3150	TRAPPC10	chr21	ENSG00000160218	Frame shift del	c.858del
3151	TRAPPC10	chr21	ENSG00000160218	Frame shift del	c.858del
3152	TRAPPC2	chrX	ENSG00000196459	3'UTR	c.*594del
3153	TRAPPC6B	chr14	ENSG00000182400	3'UTR	c.*2331T>C
3154	TRAPPC9	chr8	ENSG00000167632	Intron	c.3105-22465del
3155	TRDN	chr6	ENSG00000186439	3'UTR	c.*1112G>A
3156	TRHDE	chr12	ENSG00000072657	Missense	c.2309G>A
3157	TRHDE	chr12	ENSG00000072657	3'UTR	c.*2347del
3158	TRIM13	chr13	ENSG00000204977	Intron	c.-218+503del
3159	TRIM14	chr9	ENSG00000106785	Missense	c.1022A>G
3160	TRIM2	chr4	ENSG00000109654	3'UTR	c.*3998_*3999del
3161	TRIM38	chr6	ENSG00000112343	3'UTR	c.*1166del
3162	TRIM54	chr2	ENSG00000138100	Intron	c.513+41del
3163	TRIP11	chr14	ENSG00000100815	3'UTR	c.*1339del
3164	TRIP13	chr5	ENSG00000071539	3'UTR	c.*527del
3165	TRIQK	chr8	ENSG00000205133	3'UTR	c.*338T>C
3166	TRIQK	chr8	ENSG00000205133	3'UTR	c.*261G>T
3167	TRMO	chr9	ENSG00000136932	Intron	c.73+75G>T
3168	TRMT10B	chr9	ENSG00000165275	Intron	c.652+88_652+90del
3169	TRMT11	chr6	ENSG00000066651	Intron	c.522+41dup
3170	TRMT44	chr4	ENSG00000155275	Missense	c.133G>C
3171	TRMT61A	chr14	ENSG00000166166	3'UTR	c.*819C>G
3172	TRNAU1AP	chr1	ENSG00000180098	Missense	c.23G>T
3173	TROAP	chr12	ENSG00000135451	Frame shift Ins	c.2054dup
3174	TRPM2	chr21	ENSG00000142185	Silent	c.2991G>A
3175	TRPM3	chr9	ENSG00000083067	3'UTR	c.*867A>G
3176	TRPM6	chr9	ENSG00000119121	Missense	c.5540T>A
3177	TRPM7	chr15	ENSG00000092439	5'UTR	c.-138T>C
3178	TRPS1	chr8	ENSG00000104447	3'UTR	c.*1843del

Number	Gene name	Chromosome	Gene_ID	Mutation	Nucleotide substitution
3179	TRPS1	chr8	ENSG00000104447	3'UTR	c.*1504T>C
3180	TRPV1	chr17	ENSG00000196689	Missense	c.863T>C
3181	TRPV6	chr7	ENSG00000165125	Silent	c.1098G>A
3182	TSC1	chr9	ENSG00000165699	3'UTR	c.*988C>A
3183	TSGA10IP	chr11	ENSG00000175513	anslation_Start_S	c.1A>G
3184	TSHZ2	chr20	ENSG00000182463	5'UTR	c.-458del
3185	TSPAN13	chr7	ENSG00000106537	3'UTR	c.*631A>G
3186	TSPAN14	chr10	ENSG00000108219	3'UTR	c.*4067T>C
3187	TSPAN16	chr19	ENSG00000130167	Splice site	c.69+2del
3188	TSPAN17	chr5	ENSG00000048140	Missense	c.493G>C
3189	TSPAN9	chr12	ENSG00000011105	Splice site	c.565-2A>G
3190	TSPEAR	chr21	ENSG00000175894	Intron	c.82+11G>A
3191	TSSK6	chr19	ENSG00000178093	Silent	c.537A>G
3192	TTC13	chr1	ENSG00000143643	Missense	c.1319A>G
3193	TTC26	chr7	ENSG00000105948	Intron	c.257-40T>C
3194	TTC28	chr22	ENSG00000100154	3'UTR	c.*1869C>T
3195	TTC30A	chr2	ENSG00000197557	Missense	c.1918C>T
3196	TTF1	chr9	ENSG00000125482	Intron	c.1857-72T>A
3197	TTLL7	chr1	ENSG00000137941	Missense	c.2287T>G
3198	TTYH3	chr7	ENSG00000136295	3'UTR	c.*978del
3199	TUBA1B	chr12	ENSG00000123416	3'Flank	
3200	TUBGCP3	chr13	ENSG00000126216	Splice site	c.1555+2T>C
3201	TULP3	chr12	ENSG00000078246	Intron	c.41+126A>G
3202	TULP3	chr12	ENSG00000078246	3'UTR	c.*894A>G
3203	TXLNA	chr1	ENSG00000084652	Frame shift Ins	c.1204dup
3204	TXNL4B	chr16	ENSG00000140830	Intron	c.132+342del
3205	TYW1	chr7	ENSG00000198874	5'UTR	c.-124C>A
3206	TYW1	chr7	ENSG00000198874	Splice site	c.984+2T>C
3207	TYW1	chr7	ENSG00000198874	3'UTR	c.*101A>G
3208	UBA6	chr4	ENSG00000033178	3'UTR	c.*2837del
3209	UBA6	chr4	ENSG00000033178	3'UTR	c.*911del
3210	UBA6	chr4	ENSG00000033178	Intron	c.897+37del
3211	UBALD1	chr16	ENSG00000153443	5'UTR	c.-60A>G
3212	UBE2A	chrX	ENSG00000077721	3'UTR	c.*1122del
3213	UBE2C	chr20	ENSG00000175063	Splice region	c.217-2del
3214	UBE2E1	chr3	ENSG00000170142	Intron	c.203+58T>G
3215	UBE2F	chr2	ENSG00000184182	Intron	c.214+8566C>T
3216	UBE2G2	chr21	ENSG00000184787	3'UTR	c.*1110T>A
3217	UBE2H	chr7	ENSG00000186591	5'UTR	c.-1T>A
3218	UBE2J1	chr6	ENSG00000198833	Intron	c.105+87A>G
3219	UBE2L3	chr22	ENSG00000185651	3'UTR	c.*545A>G
3220	UBE2V2	chr8	ENSG00000169139	5'UTR	c.-899T>C
3221	UBE3B	chr12	ENSG00000151148	Intron	c.2077-1380A>G
3222	UBE4A	chr11	ENSG00000110344	Intron	c.562-13A>G
3223	UBFD1	chr16	ENSG00000103353	3'UTR	c.*454del
3224	UBP1	chr3	ENSG00000153560	Intron	c.448+42T>C
3225	UBQLN4	chr1	ENSG00000160803	Missense	c.34C>T
3226	UBR4	chr1	ENSG00000127481	Intron	c.3900-24T>A
3227	UBR5	chr8	ENSG00000104517	Missense	c.4193T>C
3228	UBXN10	chr1	ENSG00000162543	3'UTR	c.*1508T>C
3229	UBXN2A	chr2	ENSG00000173960	3'UTR	c.*1594T>A
3230	UBXN2A	chr2	ENSG00000173960	3'UTR	c.*2104del
3231	UBXN2A	chr2	ENSG00000173960	3'UTR	c.*3489A>G

Number	Gene name	Chromosome	Gene_ID	Mutation	Nucleotide substitution
3232	UFC1	chr1	ENSG00000143222	5'Flank	
3233	UGCG	chr9	ENSG00000148154	3'UTR	c.*835C>T
3234	UGDH	chr4	ENSG00000109814	Intron	c.1263+63A>T
3235	UHMK1	chr1	ENSG00000152332	Intron	c.754-4del
3236	UHRF1	chr19	ENSG00000276043	Silent	c.390G>A
3237	ULBP1	chr6	ENSG00000111981	Missense	c.152A>G
3238	UMODL1	chr21	ENSG00000177398	Missense	c.451G>A
3239	UNC13B	chr9	ENSG00000198722	Missense	c.2675C>T
3240	UNC5B	chr10	ENSG00000107731	Missense	c.727G>A
3241	UNC5D	chr8	ENSG00000156687	Silent	c.177G>A
3242	UNCX	chr7	ENSG00000164853	Missense	c.527A>G
3243	Unknown	chr13		IGR	
3244	Unknown	chr20		IGR	
3245	Unknown	chr1		IGR	
3246	Unknown	chr1		IGR	
3247	UQCC3	chr11	ENSG00000204922	3'UTR	c.*122dup
3248	UQCRB	chr8	ENSG00000156467	3'UTR	c.*434del
3249	UQCRC2	chr16	ENSG00000140740	Missense	c.929A>G
3250	UQCRHL	chr1	ENSG00000233954	Missense	c.146G>A
3251	USH2A	chr1	ENSG00000042781	Missense	c.1247C>T
3252	USP14	chr18	ENSG00000101557	Missense	c.265G>A
3253	USP15	chr12	ENSG00000135655	Frame shift del	c.454del
3254	USP18	chr22	ENSG00000184979	Missense	c.355C>T
3255	USP19	chr3	ENSG00000172046	Missense	c.2045T>C
3256	USP2	chr11	ENSG00000036672	3'UTR	c.*787del
3257	USP24	chr1	ENSG00000162402	Missense	c.4432T>C
3258	USP34	chr2	ENSG00000115464	Intron	c.44-1180T>C
3259	USP35	chr11	ENSG00000118369	3'UTR	c.*565del
3260	USP36	chr17	ENSG00000055483	Missense	c.812T>C
3261	USP40	chr2	ENSG00000085982	Intron	c.2648-25del
3262	USP47	chr11	ENSG00000170242	Intron	c.3559-17A>T
3263	USP48	chr1	ENSG00000090686	3'UTR	c.*45dup
3264	USP8	chr15	ENSG00000138592	3'UTR	c.*618del
3265	USPL1	chr13	ENSG00000132952	Intron	c.1113-5del
3266	UST	chr6	ENSG00000111962	Missense	c.479A>C
3267	UTP18	chr17	ENSG00000011260	Intron	c.455+35T>C
3268	UTP20	chr12	ENSG00000120800	5'UTR	c.-155A>G
3269	UTP4	chr16	ENSG00000141076	Splice region	c.-2-4del
3270	UTP6	chr17	ENSG00000108651	Intron	c.621+40T>C
3271	UTS2R	chr17	ENSG00000181408	5'UTR	c.-24T>C
3272	VAMP1	chr12	ENSG00000139190	Frame shift del	c.351del
3273	VAMP4	chr1	ENSG00000117533	3'UTR	c.*1501del
3274	VANGL1	chr1	ENSG00000173218	Frame shift del	c.515del
3275	VANGL1	chr1	ENSG00000173218	Frame shift del	c.515del
3276	VASP	chr19	ENSG00000125753	Missense	c.92A>T
3277	VAV1	chr19	ENSG00000141968	Intron	c.928-69T>G
3278	VCP	chr9	ENSG00000165280	Intron	c.2315+32A>G
3279	VDAC2	chr10	ENSG00000165637	5'UTR	c.-64T>G
3280	VGLL2	chr6	ENSG00000170162	Missense	c.920G>A
3281	VGLL4	chr3	ENSG00000144560	3'UTR	c.*1941del
3282	VIPR1	chr3	ENSG00000114812	3'UTR	c.*169del
3283	VIRMA	chr8	ENSG00000164944	Silent	c.4281A>G
3284	VIT	chr2	ENSG00000205221	3'UTR	c.*294T>G

Number	Gene name	Chromosome	Gene_ID	Mutation	Nucleotide substitution
3285	VMA21	chrX	ENSG00000160131	3'UTR	c.*4291del
3286	VPS13A	chr9	ENSG00000197969	Missense	c.5705C>A
3287	VPS13A	chr9	ENSG00000197969	Intron	c.6379-22_6379-21del
3288	VPS13A	chr9	ENSG00000197969	3'UTR	c.*3675A>T
3289	VPS13C	chr15	ENSG00000129003	3'UTR	c.*308A>G
3290	VPS13D	chr1	ENSG00000048707	Intron	c.7274-10dup
3291	VPS13D	chr1	ENSG00000048707	Intron	c.8495+28del
3292	VPS26B	chr11	ENSG00000151502	3'UTR	c.*766C>T
3293	VPS35L	chr16	ENSG00000103544	Intron	c.701-666del
3294	VPS37B	chr12	ENSG00000139722	5'UTR	c.-1102_-1099del
3295	VPS41	chr7	ENSG00000006715	3'UTR	c.*1142A>G
3296	VPS41	chr7	ENSG00000006715	3'UTR	c.*824A>G
3297	VPS8	chr3	ENSG00000156931	Missense	c.1364T>C
3298	VPS8	chr3	ENSG00000156931	Nonsense	c.2683C>T
3299	VPS8	chr3	ENSG00000156931	Intron	c.3586-9del
3300	VPS9D1	chr16	ENSG00000075399	Intron	c.1697+19A>G
3301	VRK2	chr2	ENSG00000028116	Frame shift del	c.1089del
3302	VRTN	chr14	ENSG00000133980	3'UTR	c.*639del
3303	VSIG10	chr12	ENSG00000176834	Missense	c.752A>G
3304	VSIG10	chr12	ENSG00000176834	Frame shift del	c.671del
3305	VSTM5	chr11	ENSG00000214376	3'UTR	c.*2300del
3306	VTI1B	chr14	ENSG00000100568	3'UTR	c.*1126A>C
3307	VWA1	chr1	ENSG00000179403	3'UTR	c.*1623del
3308	VWC2	chr7	ENSG00000188730	Intron	c.827-36C>T
3309	VWC2L	chr2	ENSG00000174453	3'UTR	c.*473del
3310	VWDE	chr7	ENSG00000146530	Missense	c.2081T>C
3311	VXN	chr8	ENSG00000169085	Missense	c.236C>T
3312	VXN	chr8	ENSG00000169085	3'UTR	c.*1254A>G
3313	WBP1	chr2	ENSG00000239779	Frame shift del	c.552del
3314	WBP1	chr2	ENSG00000239779	Frame shift del	c.552del
3315	WDCP	chr2	ENSG00000163026	Intron	c.1819-219T>C
3316	WDFY2	chr13	ENSG00000139668	Missense	c.1103A>G
3317	WDFY3	chr4	ENSG00000163625	Silent	c.1749T>G
3318	WDHD1	chr14	ENSG00000198554	Intron	c.1153+29A>G
3319	WDPCP	chr2	ENSG00000143951	Intron	c.1915+29414_1915+29415de
3320	WDR1	chr4	ENSG00000071127	3'UTR	c.*630del
3321	WDR12	chr2	ENSG00000138442	5'UTR	c.-599del
3322	WDR18	chr19	ENSG00000065268	Missense	c.402T>C
3323	WDR20	chr14	ENSG00000140153	3'UTR	c.*1695G>A
3324	WDR24	chr16	ENSG00000127580	Missense	c.2444A>G
3325	WDR36	chr5	ENSG00000134987	3'UTR	c.*1802A>G
3326	WDR41	chr5	ENSG00000164253	Frame shift del	c.1101del
3327	WDR41	chr5	ENSG00000164253	Frame shift del	c.1101del
3328	WDR44	chrX	ENSG00000131725	Intron	c.2648-1846_2648-1845del
3329	WDR44	chrX	ENSG00000131725	3'UTR	c.*418del
3330	WDR55	chr5	ENSG00000120314	Frame shift del	c.1022del
3331	WDR60	chr7	ENSG00000126870	3'UTR	c.*235G>A
3332	WDR7	chr18	ENSG00000091157	3'UTR	c.*3229G>A
3333	WDR70	chr5	ENSG00000082068	5'Flank	
3334	WDR70	chr5	ENSG00000082068	3'UTR	c.*274A>G
3335	WDR73	chr15	ENSG00000177082	3'Flank	
3336	WDR81	chr17	ENSG00000167716	Missense	c.2476C>A
3337	WDR83	chr19	ENSG00000123154	Missense	c.635A>G

Number	Gene name	Chromosome	Gene_ID	Mutation	Nucleotide substitution
3338	WDR87	chr19	ENSG00000171804	Frame shift del	c.6187del
3339	WDR90	chr16	ENSG00000161996	Silent	c.3594C>T
3340	WIF1	chr12	ENSG00000156076	Intron	c.288+18T>C
3341	WIPF3	chr7	ENSG00000122574	Frame shift del	c.23del
3342	WIPI2	chr7	ENSG00000157954	Missense	c.521C>A
3343	WNK1	chr12	ENSG00000060237	Intron	c.5584-6del
3344	WNK2	chr9	ENSG00000165238	3'UTR	c.*120del
3345	WRAP53	chr17	ENSG00000141499	Frame shift del	c.1565del
3346	WRAP53	chr17	ENSG00000141499	Frame shift del	c.1565del
3347	WRN	chr8	ENSG00000165392	3'UTR	c.*537A>G
3348	WTAP	chr6	ENSG00000146457	Splice region	c.607+6G>T
3349	WTAP	chr6	ENSG00000146457	3'UTR	c.*103T>C
3350	WWC2	chr4	ENSG00000151718	3'UTR	c.*1455del
3351	XDH	chr2	ENSG00000158125	Missense	c.3526C>T
3352	XIAP	chrX	ENSG00000101966	Missense	c.695G>A
3353	XIAP	chrX	ENSG00000101966	3'UTR	c.*5505_*5506del
3354	XIAP	chrX	ENSG00000101966	3'UTR	c.*5507T>A
3355	XIST	chrX	ENSG00000229807	RNA	n.2319T>C
3356	XK	chrX	ENSG00000047597	3'UTR	c.*1500A>G
3357	XKR6	chr8	ENSG00000171044	3'UTR	c.*359del
3358	XPA	chr9	ENSG00000136936	3'UTR	c.*134A>G
3359	XPNPEP1	chr10	ENSG00000108039	Missense	c.125G>A
3360	XPOT	chr12	ENSG00000184575	3'UTR	c.*517del
3361	XRCC2	chr7	ENSG00000196584	3'UTR	c.*1812del
3362	XYLB	chr3	ENSG00000093217	3'UTR	c.*584T>C
3363	YAF2	chr12	ENSG00000015153	3'UTR	c.*858A>G
3364	YARS2	chr12	ENSG00000139131	Intron	c.947+1009C>T
3365	YBX1	chr1	ENSG00000065978	Missense	c.217C>T
3366	YEATS2	chr3	ENSG00000163872	Intron	c.3583-4del
3367	YKT6	chr7	ENSG00000106636	Intron	c.460-180T>C
3368	YWHAZ	chr8	ENSG00000164924	Intron	c.-11-12del
3369	ZBED1	chrX	ENSG00000214717	5'UTR	c.-230dup
3370	ZBTB10	chr8	ENSG00000205189	Nonsense	c.490C>T
3371	ZBTB21	chr21	ENSG00000173276	Missense	c.2317A>G
3372	ZBTB21	chr21	ENSG00000173276	Silent	c.555T>C
3373	ZBTB34	chr9	ENSG00000177125	Missense	c.403G>A
3374	ZBTB37	chr1	ENSG00000185278	5'Flank	
3375	ZBTB4	chr17	ENSG00000174282	Frame shift del	c.1479del
3376	ZBTB4	chr17	ENSG00000174282	Frame shift del	c.1479del
3377	ZBTB40	chr1	ENSG00000184677	3'UTR	c.*2013T>C
3378	ZBTB44	chr11	ENSG00000196323	3'UTR	c.*4923del
3379	ZBTB44	chr11	ENSG00000196323	3'UTR	c.*1247del
3380	ZC3H18	chr16	ENSG00000158545	Frame shift Ins	c.2037dup
3381	ZCCHC7	chr9	ENSG00000147905	3'UTR	c.*328del
3382	ZCCHC7	chr9	ENSG00000147905	Missense	c.1376A>C
3383	ZCWPW2	chr3	ENSG00000206559	Frame shift del	c.392del
3384	ZDHHC18	chr1	ENSG00000204160	Intron	c.532-27T>C
3385	ZDHHC3	chr3	ENSG00000163812	3'UTR	c.*2381C>A
3386	ZEB2	chr2	ENSG00000169554	3'UTR	c.*560del
3387	ZFAND5	chr9	ENSG00000107372	3'UTR	c.*4359_*4360insGT
3388	ZFAND5	chr9	ENSG00000107372	3'UTR	c.*2222G>T
3389	ZFHX4	chr8	ENSG00000091656	Frame shift del	c.4248del
3390	ZFP1	chr16	ENSG00000184517	3'UTR	c.*1698dup

Number	Gene name	Chromosome	Gene_ID	Mutation	Nucleotide substitution
3391	ZFP14	chr19	ENSG00000142065	Missense	c.1232T>A
3392	ZFP28	chr19	ENSG00000196867	Missense	c.2279G>A
3393	ZFP36L2	chr2	ENSG00000152518	3'UTR	c.*1544del
3394	ZFPM2	chr8	ENSG00000169946	RNA	n.264A>G
3395	ZFR	chr5	ENSG00000056097	Intron	c.2948-50del
3396	ZFYVE9	chr1	ENSG00000157077	Silent	c.2421A>G
3397	ZGLP1	chr19	ENSG00000220201	Missense	c.130C>T
3398	ZGRF1	chr4	ENSG00000138658	3'UTR	c.*175del
3399	ZGRF1	chr4	ENSG00000138658	Intron	c.102+1904del
3400	ZHX1	chr8	ENSG00000165156	Frame shift del	c.1692del
3401	ZHX1	chr8	ENSG00000165156	Frame shift del	c.1692del
3402	ZKSCAN1	chr7	ENSG00000106261	3'UTR	c.*1233del
3403	ZKSCAN8	chr6	ENSG00000198315	3'UTR	c.*1150A>G
3404	ZMAT3	chr3	ENSG00000172667	3'UTR	c.*6568del
3405	ZMAT3	chr3	ENSG00000172667	3'UTR	c.*6353del
3406	ZMYM3	chrX	ENSG00000147130	Frame shift del	c.143del
3407	ZMYM3	chrX	ENSG00000147130	Frame shift del	c.143del
3408	ZMYM4	chr1	ENSG00000146463	Intron	c.840+20del
3409	ZMYM6	chr1	ENSG00000163867	Intron	c.2147-27T>C
3410	ZMYM6	chr1	ENSG00000163867	Missense	c.1205G>A
3411	ZMYM6	chr1	ENSG00000163867	Missense	c.655G>A
3412	ZMYND12	chr1	ENSG00000066185	Splice region	c.110+6C>T
3413	ZMYND8	chr20	ENSG00000101040	3'UTR	c.*332del
3414	ZMYND8	chr20	ENSG00000101040	Silent	c.2634T>C
3415	ZNF131	chr5	ENSG00000172262	5'UTR	c.-94del
3416	ZNF157	chrX	ENSG00000147117	3'UTR	c.*321del
3417	ZNF175	chr19	ENSG00000105497	3'UTR	c.*83A>G
3418	ZNF204P	chr6	ENSG00000204789	RNA	n.550T>C
3419	ZNF207	chr17	ENSG00000010244	3'UTR	c.*11937del
3420	ZNF211	chr19	ENSG00000121417	5'UTR	c.-62C>T
3421	ZNF213	chr16	ENSG00000085644	Missense	c.701A>G
3422	ZNF236	chr18	ENSG00000130856	Missense	c.781G>A
3423	ZNF250	chr8	ENSG00000196150	3'UTR	c.*3983del
3424	ZNF286A	chr17	ENSG00000187607	3'UTR	c.*3139del
3425	ZNF292	chr6	ENSG00000188994	3'UTR	c.*462A>G
3426	ZNF331	chr19	ENSG00000130844	3'UTR	c.*1401T>A
3427	ZNF331	chr19	ENSG00000130844	3'UTR	c.*1407G>C
3428	ZNF354A	chr5	ENSG00000169131	5'UTR	c.-150C>T
3429	ZNF365	chr10	ENSG00000138311	3'UTR	c.*1900del
3430	ZNF391	chr6	ENSG00000124613	3'UTR	c.*1849C>A
3431	ZNF410	chr14	ENSG00000119725	5'UTR	c.-233del
3432	ZNF420	chr19	ENSG00000197050	3'UTR	c.*661_*662del
3433	ZNF420	chr19	ENSG00000197050	3'UTR	c.*702del
3434	ZNF436	chr1	ENSG00000125945	3'UTR	c.*1361T>C
3435	ZNF438	chr10	ENSG00000183621	Silent	c.1275C>T
3436	ZNF438	chr10	ENSG00000183621	Missense	c.4G>T
3437	ZNF445	chr3	ENSG00000185219	3'UTR	c.*2818_*2819del
3438	ZNF462	chr9	ENSG00000148143	3'UTR	c.*2101del
3439	ZNF469	chr16	ENSG00000225614	Missense	c.2114T>C
3440	ZNF488	chr10	ENSG00000265763	Silent	c.861G>A
3441	ZNF500	chr16	ENSG00000103199	Missense	c.131C>A
3442	ZNF502	chr3	ENSG00000196653	3'UTR	c.*1295_*1298del
3443	ZNF512	chr2	ENSG00000243943	5'Flank	

Number	Gene name	Chromosome	Gene_ID	Mutation	Nucleotide substitution
3444	ZNF513	chr2	ENSG00000163795	Silent	c.780G>A
3445	ZNF516	chr18	ENSG00000101493	3'UTR	c.*4006G>A
3446	ZNF516	chr18	ENSG00000101493	3'UTR	c.*469C>T
3447	ZNF527	chr19	ENSG00000189164	3'UTR	c.*644G>A
3448	ZNF548	chr19	ENSG00000188785	5'UTR	c.-91A>G
3449	ZNF572	chr8	ENSG00000180938	3'UTR	c.*1448T>C
3450	ZNF575	chr19	ENSG00000176472	Intron	c.136-5del
3451	ZNF576	chr19	ENSG00000124444	3'UTR	c.*766A>G
3452	ZNF583	chr19	ENSG00000198440	3'UTR	c.*2693T>C
3453	ZNF587	chr19	ENSG00000198466	Splice region	c.33+5T>G
3454	ZNF606	chr19	ENSG00000166704	Intron	c.32-32T>C
3455	ZNF610	chr19	ENSG00000167554	Intron	c.190+12T>C
3456	ZNF611	chr19	ENSG00000213020	Missense	c.1460G>A
3457	ZNF621	chr3	ENSG00000172888	3'UTR	c.*851del
3458	ZNF628	chr19	ENSG00000197483	Missense	c.2057C>T
3459	ZNF639	chr3	ENSG00000121864	3'UTR	c.*508T>C
3460	ZNF652	chr17	ENSG00000198740	3'UTR	c.*3076del
3461	ZNF655	chr7	ENSG00000197343	Intron	c.137-933G>A
3462	ZNF664	chr12	ENSG00000179195	3'UTR	c.*1339G>A
3463	ZNF682	chr19	ENSG00000197124	Silent	c.492T>C
3464	ZNF697	chr1	ENSG00000143067	Silent	c.1497C>T
3465	ZNF697	chr1	ENSG00000143067	Missense	c.1043C>T
3466	ZNF7	chr8	ENSG00000147789	Missense	c.128T>C
3467	ZNF704	chr8	ENSG00000164684	3'UTR	c.*11958del
3468	ZNF708	chr19	ENSG00000182141	Intron	c.4-15_4-14del
3469	ZNF711	chrX	ENSG00000147180	Intron	c.917-859dup
3470	ZNF738	chr19	ENSG00000172687	3'UTR	c.*1141_*1142del
3471	ZNF740	chr12	ENSG00000139651	3'UTR	c.*1533del
3472	ZNF772	chr19	ENSG00000197128	3'UTR	c.*2911del
3473	ZNF780A	chr19	ENSG00000197782	Missense	c.14C>T
3474	ZNF791	chr19	ENSG00000173875	Missense	c.332G>A
3475	ZNF81	chrX	ENSG00000197779	Intron	c.278-9294del
3476	ZNF836	chr19	ENSG00000196267	Missense	c.2584T>C
3477	ZNF839	chr14	ENSG00000022976	3'UTR	c.*425T>C
3478	ZNF883	chr9	ENSG00000285447	Frame shift Ins	c.571dup
3479	ZNF90	chr19	ENSG00000213988	Intron	c.130+61del
3480	ZNF90	chr19	ENSG00000213988	3'Flank	
3481	ZNRF3	chr22	ENSG00000183579	Missense	c.853A>G
3482	ZNRF4	chr19	ENSG00000105428	Missense	c.530G>A
3483	ZP1	chr11	ENSG00000149506	Silent	c.276A>C
3484	ZPLD1	chr3	ENSG00000170044	Intron	c.1120+95A>G
3485	ZSCAN21	chr7	ENSG00000166529	3'UTR	c.*45C>T
3486	ZSCAN29	chr15	ENSG00000140265	3'UTR	c.*2710_*2711del
3487	ZSWIM8	chr10	ENSG00000214655	Missense	c.4151G>A
3488	ZSWIM9	chr19	ENSG00000185453	Intron	c.589-5068del
3489	ZSWIM9	chr19	ENSG00000185453	Missense	c.934G>A
3490	ZZEF1	chr17	ENSG00000074755	Missense	c.485G>A

1
