# Supplementary material for: Stimulating T cell responses against patient-derived breast cancer cells with neoantigen peptide-loaded peripheral blood mononuclear cells
Source: Cancer Immunol Immunother. 2024 Feb 13;73(3):43. doi: 10.1007/s00262-024-03627-3 (PMC10864427; doi:10.1007/s00262-024-03627-3)
Supplement: Supplementary file 7 — (PDF 136 kb) [file 262_2024_3627_MOESM7_ESM.pdf]

**Supplementary Table S4a. List of 646 nonsynonymous mutations of PC-B-142CA**

| Number | HUGO Symbol | Chromosome | Transcript ID | HGVSp | MT Epitope Seq | WT Epitope Seq |
|---|---|---|---|---|---|---|
| 1 | AASDH | chr4 | ENST00000205214 | p.L144R | LVLFRRHWK | LVLFRLHWK |
| 2 | ABCA10 | chr17 | ENST00000269081 | p.D552H | LLHEPTAGL | LLDEPTAGL |
| 3 | ABCA6 | chr17 | ENST00000284425 | p.E659Q | LLRQRRADHVIL | LLRERRADHVIL |
| 4 | ABCG1 | chr21 | ENST00000343687 | p.F264L | SASCLQVVSLMK | SASCFQVVSLMK |
| 5 | ABHD11 | chr7 | ENST00000222800 | p.A238V | DVLTQHLDK | DALTQHLDK |
| 6 | ABL2 | chr1 | ENST00000344730 | p.E720Q | LPKKSQESAA | LPKKSEESAA |
| 7 | ACAP3 | chr1 | ENST00000353662 | p.E625K | GAEVNWADAK | GAEVNWADAE |
| 8 | ACCS | chr11 | ENST00000263776 | p.D487N | KSQVAENPR | KSQVAEDPR |
| 9 | ACP2 | chr11 | ENST00000256997 | p.E314Q | PYASCHIFQL | PYASCHIFEL |
| 10 | ACSL6 | chr5 | ENST00000379246 | p.A580V | AQGEYVVPEK | AQGEYVAPEK |
| 11 | ACSL6 | chr5 | ENST00000651250 | p.A541V | LPGEYVVPE | LPGEYVAPE |
| 12 | ACTL7A | chr9 | ENST00000333999 | p.S326C | KPCLIKSM | KPSLIKSM |
| 13 | ACTR5 | chr20 | ENST00000243903 | p.L476V | LQYIVDRYPK | LQYILDRYPK |
| 14 | ADAM12 | chr10 | ENST00000368676 | p.A571T | KVSKSSFTK | KVSKSSFAK |
| 15 | ADAM29 | chr4 | ENST00000404450 | p.G125E | CFGGFQEI | CFGGFQGI |
| 16 | ADAM8 | chr10 | ENST00000415217 | p.N436T | RNRCCTSTTCQL | RNRCCNSTTCQL |
| 17 | ADCY10 | chr1 | ENST00000367848 | p.Q1154E | ETENCFQIIK | ETQNCFQIIK |
| 18 | ADCY9 | chr16 | ENST00000294016 | p.S140C | IYFAVHMRCRLI | IYFAVHMRSRLI |
| 19 | ADGRL1 | chr19 | ENST00000340736 | p.E274K | KTDIDLAVDK | KTDIDLAVDE |
| 20 | ADK | chr10 | ENST00000372734 | p.E340Q | RTGCTFPQK | RTGCTFPEK |
| 21 | ADRA2A | chr10 | ENST00000280155 | p.F405L | FPLFFTYTLTAV | FPFFFTYTLTAV |
| 22 | AGFG1 | chr2 | ENST00000310078 | p.L163V | KPLKSVLGDSA | KPLKSLLGDSA |
| 23 | AHCYL2 | chr7 | ENST00000325006 | p.E555K | ALIELYNAPK | ALIELYNAPE |
| 24 | AHI1 | chr6 | ENST00000265602 | p.M56I | TIRSNLHYIK | TIRSNLHYMK |
| 25 | AKAP13 | chr15 | ENST00000361243 | p.L1136V | LPVAVQDKA | LPVQDKA |
| 26 | AKAP17A | chrX | ENST00000313871 | p.R318T | ERKTEEKLR | ERKREEKLR |
| 27 | ALDH9A1 | chr1 | ENST00000354775 | p.S50F | ARVEPADAF | ARVEPADAS |
| 28 | ALK | chr2 | ENST00000389048 | p.S326L | LLLNTLADSK | LLLNTSADSK |
| 29 | ALMS1 | chr2 | ENST00000423048 | p.E644Q | RSPLQEAQSK | RSPLQEAESK |
| 30 | ALPK3 | chr15 | ENST00000258888 | p.P1410A | VPGSPGTAG | VPGSPGTPG |
| 31 | AMER3 | chr2 | ENST00000321420 | p.R168T | NLFHIRTNK | NLFHIRRNK |
| 32 | AMER3 | chr2 | ENST00000321420 | p.R197Q | DPGGRQSKAF | DPGGRRSKAF |
| 33 | ANAPC5 | chr12 | ENST00000261819 | p.R313C | CCFGHYQQA | CRFGHYQQA |
| 34 | ANGPTL3 | chr1 | ENST00000371129 | p.P31A | SSFDSLSAEPK | SSFDSLSPEPK |
| 35 | ANK2 | chr4 | ENST00000357077 | p.E1204Q | AQPMHSQLVK | AQPMHSELVK |
| 36 | ANK3 | chr10 | ENST00000280772 | p.M1005I | SRHHGIRII | SRHHGMRII |
| 37 | ANK3 | chr10 | ENST00000280772 | p.E3205Q | KPSPIPEVSQ | KPSPIPEVSE |
| 38 | ANKRD44 | chr2 | ENST00000409919 | p.H284D | FAAASTDGA | FAAASTHGA |
| 39 | AP3M2 | chr8 | ENST00000396926 | p.E111Q | VYQVLEEML | VYEVLEEML |
| 40 | APAF1 | chr12 | ENST00000359972 | p.L251V | CQILVTTRDK | CQILLTTRDK |
| 41 | ARFGEF1 | chr8 | ENST00000262215 | p.E1518Q | FTLQIWDKT | FTLEIWDKT |
| 42 | ARHGAP26 | chr5 | ENST00000274498 | p.E28Q | AQLDKTNKFIK | AELDKTNKFIK |
| 43 | ARNT2 | chr15 | ENST00000303329 | p.E427K | FTFQNPYSDK | FTFQNPYSDE |
| 44 | ATAD2B | chr2 | ENST00000381024 | p.H41Y | YYQPTSYRPRLL | YHQPTSYRPRLL |
| 45 | ATG13 | chr11 | ENST00000359513 | p.P422S | SPETESSL | SPETESPL |
| 46 | ATIC | chr2 | ENST00000236959 | p.E347K | VSDGIIAPGYK | VSDGIIAPGYE |
| 47 | ATMIN | chr16 | ENST00000299575 | p.S240C | AQNQKLCNK | AQNQKLSNK |
| 48 | ATP5F1B | chr12 | ENST00000262030 | p.E448K | AILGMDELSK | AILGMDELSE |
| 49 | AURKC | chr19 | ENST00000302804 | p.E91K | RRKIEIQAH | RREIEIQAH |
| 50 | B3GNT5 | chr3 | ENST00000326505 | p.S105Y | RRYGIRRTW | RRSGIRRTW |
| 51 | B4GALNT4 | chr11 | ENST00000329962 | p.E412K | KYMKMDKEKG | KYMKMDKEEG |

| Number | HUGO Symbol | Chromosome | Transcript ID | HGVSp | MT Epitope Seq | WT Epitope Seq |
|---|---|---|---|---|---|---|
| 52 | BBC3 | chr19 | ENST00000449228 | p.L74V | SVLRPVRAR | SVLRPLRAR |
| 53 | BCL2L11 | chr2 | ENST00000393256 | p.H57Q | CPQGSPQGPL | CPHGSPQGPL |
| 54 | BCOR | chrX | ENST00000378444 | p.P397R | YPKAREGGEGA | YPKAPEGGEGA |
| 55 | BEST1 | chr11 | ENST00000378043 | p.W445C | KACKLKAVDAFK | KAWKLKAVDAFK |
| 56 | BHLHE41 | chr12 | ENST00000242728 | p.G81A | KLTTLAHLEK | KLTTLGHLEK |
| 57 | BLM | chr15 | ENST00000355112 | p.Q615E | LPVSSTAENI | LPVSSTAQNI |
| 58 | BLZF1 | chr1 | ENST00000329281 | p.E137K | LKNSKRRLL | LKNSRLL |
| 59 | BMT2 | chr7 | ENST00000297145 | p.G336A | NYPAMLYIPQDF | NYPGMLYIPQDF |
| 60 | BRAF | chr7 | ENST00000288602 | p.S642C | CRWSGSHQF | SRWSGSHQF |
| 61 | BRCA1 | chr17 | ENST00000354071 | p.S425C | EYSGCSEKI | EYSGSSEKI |
| 62 | BRWD1 | chr21 | ENST00000333229 | p.P944A | MEHLYEFHPA | MEHLYEFHPP |
| 63 | BRWD3 | chrX | ENST00000373275 | p.E1196Q | LQNRFYRRISAL | LENRFYRRISAL |
| 64 | BTBD18 | chr11 | ENST00000436147 | p.Q102H | SHEEAQDVL | SQEEAQDVL |
| 65 | BTNL9 | chr5 | ENST00000327705 | p.L505V | HPDPLTICPV | HPDPLTICPL |
| 66 | BUB1B | chr15 | ENST00000287598 | p.L664V | GTIYSQTVSIKK | GTIYSQTLSIKK |
| 67 | C10orf113 | chr10 | ENST00000534331 | p.E141Q | GIKQKFNVSK | GIKEKFNVSK |
| 68 | C10orf120 | chr10 | ENST00000329446 | p.Q23H | ASDTMVHERK | ASDTMVQERK |
| 69 | C11orf80 | chr11 | ENST00000525449 | p.S189L | RISLVSIFLY | RISSVSIFLY |
| 70 | C1orf56 | chr1 | ENST00000368926 | p.S230F | FGRLRVGAL | SGRLRVGAL |
| 71 | C3orf20 | chr3 | ENST00000253697 | p.E9Q | QLYQQYTAMAPK | ELYQQYTAMAPK |
| 72 | C4orf54 | chr4 | ENST00000511828 | p.E920K | HTKVCEIKK | HTEVCEIKK |
| 73 | C8orf34 | chr8 | ENST00000348340 | p.I18M | AYLEKNKMGPLF | AYLEKNKIGPLF |
| 74 | C9orf72 | chr9 | ENST00000380003 | p.K156N | ERQENVQNI | ERQENVQKI |
| 75 | CA9 | chr9 | ENST00000378357 | p.A260V | VVHLSTAFVR | VVHLSTAFAR |
| 76 | CAAP1 | chr9 | ENST00000333916 | p.E342D | LELDMRARA | LELEMRARA |
| 77 | CACNA1A | chr19 | ENST00000360228 | p.G319R | TVFQCITMER | TVFQCITMEG |
| 78 | CACNG6 | chr19 | ENST00000252729 | p.Q201H | VRALLHRVS | VRALLQRVS |
| 79 | CAMSAP3 | chr19 | ENST00000446248 | p.M1248I | IPAKTMSISVDA | IPAKTMSMSVDA |
| 80 | CAMSAP3 | chr19 | ENST00000446248 | p.R1046C | GRRAWCS | GRRAWRS |
| 81 | CARF | chr2 | ENST00000402905 | p.H377Y | AYQYHELETPCL | AHQYHELETPCL |
| 82 | CASTOR1 | chr22 | ENST00000407689 | p.S111F | APLAEHHVF | APLAEHHVS |
| 83 | CASZ1 | chr1 | ENST00000377022 | p.E1077K | AAFPASAAK | AAFPASAAE |
| 84 | CC2D1A | chr19 | ENST00000318003 | p.E639Q | RFQQRTFSVI | RFEQRTFSVI |
| 85 | CCDC130 | chr19 | ENST00000221554 | p.R203T | ETDQALQAK | ERDQALQAK |
| 86 | CCDC158 | chr4 | ENST00000434846 | p.L108F | YFRQSVIDL | YLRQSVIDL |
| 87 | CCDC171 | chr9 | ENST00000380701 | p.S908C | GAAKNCFAK | GAAKNSFAK |
| 88 | CCDC39 | chr3 | ENST00000651046 | p.E81K | SLCKARERETK | SLCKARERETE |
| 89 | CCDC6 | chr10 | ENST00000263102 | p.S31L | AAMQSSCSSTL | AAMQSSCSSTS |
| 90 | CCDC85A | chr2 | ENST00000407595 | p.Q195K | SIDSQASLCK | SIDSQASLCQ |
| 91 | CCDC88A | chr2 | ENST00000263630 | p.E1321Q | NLQEENRHL | NLEEENRHL |
| 92 | CCR6 | chr6 | ENST00000341935 | p.R339S | KILKDLWCVRSK | KILKDLWCVRRK |
| 93 | CD109 | chr6 | ENST00000287097 | p.P811S | LVSSEDGATVL | LVPSEDGATVL |
| 94 | CD177 | chr19 | ENST00000618265 | p.L131F | FRCPVCLSM | LRCPVCLSM |
| 95 | CDC14A | chr1 | ENST00000336454 | p.E201Q | HPKSKIQNGYPL | HPKSKIENGYPL |
| 96 | CDH8 | chr16 | ENST00000577390 | p.P424A | SSAIRFSIDR | SSPIRFSIDR |
| 97 | CDHR3 | chr7 | ENST00000317716 | p.E198Q | DFQAGHRSF | DFEAGHRSF |
| 98 | CDKL3 | chr5 | ENST00000265334 | p.S557F | RESKKTESFK | RESKKTESSK |
| 99 | CDR1 | chrX | ENST00000370532 | p.L45F | SRLEDINFM | SRLEDINLM |
| 100 | CEBPB | chr20 | ENST00000303004 | p.S341Y | LPEPLLAYSG | LPEPLLASSG |
| 101 | CENPC | chr4 | ENST00000273853 | p.L801V | RICVDNDERK | RICLDNDERK |
| 102 | CENPC | chr4 | ENST00000515140 | p.L87V | CVDNDERSM | CLDNDERSM |
| 103 | CEP57 | chr11 | ENST00000325486 | p.R30W | SMVWHSSSPY | SMVRHSSSPY |
| 104 | CERCAM | chr9 | ENST00000372838 | p.D172V | LPVVAPMLV | LPVVAPMLD |

| Number | HUGO Symbol | Chromosome | Transcript ID | HGVSp | MT Epitope Seq | WT Epitope Seq |
|---|---|---|---|---|---|---|
| 105 | CFP | chrX | ENST00000377005 | p.E228G | CSAPGPSQK | CSAPEPSQK |
| 106 | CHD1L | chr1 | ENST00000369258 | p.K546N | ILGETNDGQ | ILGETKDGQ |
| 107 | CHD9 | chr16 | ENST00000398510 | p.D2582Y | GAFAPPLKYLCR | GAFAPPLKDLCR |
| 108 | CHPF2 | chr7 | ENST00000035307 | p.E767Q | MALFEQQQA | MALFEQEQA |
| 109 | CHRNA1 | chr2 | ENST00000348749 | p.E404Q | GIKYIAQTMK | GIKYIAETMK |
| 110 | CIITA | chr16 | ENST00000324288 | p.R539Q | KKLLQGCTL | KKLLRGCTL |
| 111 | CLCN2 | chr3 | ENST00000265593 | p.E475K | AAFGRLVGK | AAFGRLVGE |
| 112 | CLEC3A | chr16 | ENST00000651443 | p.V16A | CPRAMAKNGLAI | CPRAMAKNGLVI |
| 113 | CLEC3A | chr16 | ENST00000299642 | p.V7A | LAICILVIT | LVICILVIT |
| 114 | CLPB | chr11 | ENST00000294053 | p.E332Q | KREAQERRR | KREAEERRR |
| 115 | CMPK2 | chr2 | ENST00000256722 | p.S430C | VVDDCREK | VVDDSREK |
| 116 | CNTNAP5 | chr2 | ENST00000431078 | p.A1162T | NAMGFTGCM | NAMGFAGCM |
| 117 | COL24A1 | chr1 | ENST00000370571 | p.G732A | YPADKGAV | YPGDKGAV |
| 118 | COL27A1 | chr9 | ENST00000356083 | p.I218M | FSMYPVTQVA | FSIYPVTQVA |
| 119 | COL4A3 | chr2 | ENST00000396578 | p.E547K | QPKGQVGVPG | QPEGQVGVPG |
| 120 | COL6A3 | chr2 | ENST00000295550 | p.P2914R | KPARAKPVA | KPAPAKPVA |
| 121 | COL6A5 | chr3 | ENST00000312481 | p.L34V | VYADVVFVV | VYADVVFLV |
| 122 | COPZ2 | chr17 | ENST00000621465 | p.E89K | NVFNKTSRTK | NVFNKTSRTE |
| 123 | CPS1 | chr2 | ENST00000233072 | p.D865N | NMSLNEIEK | NMSLDEIEK |
| 124 | CREBRF | chr5 | ENST00000296953 | p.L441R | RTPSQQERMLR | LTPSQQERMLR |
| 125 | CREG1 | chr1 | ENST00000370509 | p.F61L | ARVARLVTH | ARVARFVTH |
| 126 | CRLF3 | chr17 | ENST00000324238 | p.E72K | GTLGKLLDK | GTLGKLLDE |
| 127 | CSMD3 | chr8 | ENST00000297405 | p.W3337C | GTCSGSSPH | GTWSGSSPH |
| 128 | CSTF3 | chr11 | ENST00000323959 | p.R402K | KSGRMIFKKAK | KSGRMIFKKAR |
| 129 | CXorf38 | chrX | ENST00000327877 | p.R152Q | NSCDHFVVDQK | NSCDHFVVDRK |
| 130 | CYBB | chrX | ENST00000378588 | p.D496N | FAVHHNEEK | FAVHHDEEK |
| 131 | CYP1A2 | chr15 | ENST00000343932 | p.L161M | SSSCYMEEHVSK | SSSCYLEEHVSK |
| 132 | CYP1A2 | chr15 | ENST00000343932 | p.Q273E | TVQEHYEDFDK | TVQEHYQDFDK |
| 133 | CYP1A2 | chr15 | ENST00000343932 | p.R34W | RPWVPKGL | RPRVPKGL |
| 134 | CYP4V2 | chr4 | ENST00000378802 | p.S482W | KTILWCILR | KTILSCILR |
| 135 | CYTH4 | chr22 | ENST00000248901 | p.E10K | LCHPEPAKL | LCHPEPAEL |
| 136 | DALRD3 | chr3 | ENST00000341949 | p.S113L | SPALLGQRVL | SPASLGQRVL |
| 137 | DARS2 | chr1 | ENST00000647645 | p.R402T | IMESQTLEL | IMESQRLEL |
| 138 | DCD | chr12 | ENST00000546807 | p.E47K | KPPSKTAKF | KPPSETAKF |
| 139 | DCLRE1C | chr10 | ENST00000357717 | p.D535H | STNADSQSSSH | STNADSQSSSD |
| 140 | DDX21 | chr10 | ENST00000354185 | p.D351N | FANQVEEIL | FADQVEEIL |
| 141 | DDX41 | chr5 | ENST00000330503 | p.G173E | LVEEDGIPPPIK | LVEGDGIPPPIK |
| 142 | DDX53 | chrX | ENST00000327968 | p.R28T | RGSTGSGWSGPF | RGSRGSGWSGPF |
| 143 | DENND5A | chr11 | ENST00000328194 | p.I871M | HMQNIGEIK | HIQNIGEIK |
| 144 | DGKI | chr7 | ENST00000453654 | p.H4D | HDIEEPCSL | HHIEEPCSL |
| 145 | DGKQ | chr4 | ENST00000273814 | p.R287T | GTETQATPESGK | GRETQATPESGK |
| 146 | DHRS4 | chr14 | ENST00000313250 | p.E155Q | MTKAVVPQMEK | MTKAVVPEMEK |
| 147 | DIAPH1 | chr5 | ENST00000647433 | p.S28L | GRSPDELPL | GRSPDELPS |
| 148 | DIDO1 | chr20 | ENST00000354665 | p.F385L | LQPVIEAPGA | FQPVIEAPGA |
| 149 | DIRAS3 | chr1 | ENST00000646789 | p.L20M | MPALLILRA | LPALLILRA |
| 150 | DMXL1 | chr5 | ENST00000311085 | p.R2569L | SAGPAILLHK | SAGPAILRHK |
| 151 | DMXL1 | chr5 | ENST00000311085 | p.E407Q | QVFLQQLRK | EVFLQQLRK |
| 152 | DNAAF1 | chr16 | ENST00000378553 | p.D351Y | DYGENVPAS | DDGENVPAS |
| 153 | DNAJC10 | chr2 | ENST00000264065 | p.G701A | FYAPWCAPCQNF | FYAPWCGPCQNF |
| 154 | DNAJC30 | chr7 | ENST00000395176 | p.E89K | GSAEAAKRFTR | GSAEAAERFTR |
| 155 | DOC2A | chr16 | ENST00000350119 | p.I345T | TVWDYDTGK | TVWDYDIGK |
| 156 | DOCK4 | chr7 | ENST00000423057 | p.D38H | LTQNGDMLHLLK | LTQNGDMLDLLK |
| 157 | DOCK6 | chr19 | ENST00000294618 | p.F1756L | TYFRVGGAHF | TYFRVGFYGAHF |

| Number | HUGO Symbol | Chromosome | Transcript ID | HGVSp | MT Epitope Seq | WT Epitope Seq |
|---|---|---|---|---|---|---|
| 158 | DOP1A | chr6 | ENST00000349129 | p.S1238L | LPCISGTTHTL | SPCISGTTHTL |
| 159 | DPY19L1 | chr7 | ENST00000310974 | p.P21L | TGRALRGRRRAV | TGRAPRGRRRAV |
| 160 | E2F8 | chr11 | ENST00000250024 | p.E821K | SQLVAKSFFR | SQLVAESFFR |
| 161 | EAF2 | chr3 | ENST00000273668 | p.D11H | HRRERVLKL | DRRERVLKL |
| 162 | EBF1 | chr5 | ENST00000313708 | p.V93M | RTAFVGFMEK | RTAFVGFVEK |
| 163 | ECE2 | chr3 | ENST00000357474 | p.L324V | AYVDYMEEL | AYLDYMEEL |
| 164 | ECPAS | chr9 | ENST00000259335 | p.I273M | VTNFTIMYVK | VTNFTIIYVK |
| 165 | EFCAB6 | chr22 | ENST00000396231 | p.E1034Q | HYHAITQEFQNF | HYHAITQEFENF |
| 166 | EFHC1 | chr6 | ENST00000371068 | p.F343L | TILGRTFLIY | TILGRTFFIY |
| 167 | EGFL6 | chrX | ENST00000361306 | p.R81I | CICFPGYTGK | CRCFPGYTGK |
| 168 | EHBP1 | chr2 | ENST00000263991 | p.E1084G | NGQKQIDTRAAL | NEQKQIDTRAAL |
| 169 | EMC1 | chr1 | ENST00000375199 | p.D349H | HGSMGSFSEK | DGSMGSFSEK |
| 170 | EPHX2 | chr8 | ENST00000521400 | p.E435Q | SPEQPSLSRM | SPEEPSLSRM |
| 171 | EPYC | chr12 | ENST00000261172 | p.L244F | MYDLHHLYF | MYDLHHLYL |
| 172 | ERBB2 | chr17 | ENST00000269571 | p.P1211A | HPPAAFSPA | HPPPAFSPA |
| 173 | ERG | chr21 | ENST00000288319 | p.D285Y | STVPKTEYQR | STVPKTEDQR |
| 174 | ERICH6 | chr3 | ENST00000295910 | p.L437V | HYKHGSKFVTSF | HYKHGSKFLTSF |
| 175 | ETV5 | chr3 | ENST00000306376 | p.E209K | LQMPKMMPK | LQMPKMMPE |
| 176 | EVPL | chr17 | ENST00000301607 | p.R1452Q | KQPPTVQEK | KRPPTVQEK |
| 177 | EYS | chr6 | ENST00000370621 | p.D1741N | HPSNSSLDF | HPSDSSLDF |
| 178 | EYS | chr6 | ENST00000370621 | p.Q1719E | VLNEESLLDMEK | VLNQESLLDMEK |
| 179 | F8 | chrX | ENST00000360256 | p.Q209H | SLAKEKTHTLHK | SLAKEKTQTLHK |
| 180 | FAM114A1 | chr4 | ENST00000358869 | p.E183Q | VPQITDAAT | VPEITDAAT |
| 181 | FAM13B | chr5 | ENST00000033079 | p.L712F | ASFQKSLLYY | ASLQKSLLYY |
| 182 | FAM162A | chr3 | ENST00000477892 | p.F117L | CILMVIEGK | CIFMVIEGK |
| 183 | FAM83A | chr8 | ENST00000276699 | p.D16N | KRLENVKSQ | KRLEDVKSQ |
| 184 | FBLN1 | chr22 | ENST00000262722 | p.E575K | ATRCERLPCHK | ATRCERLPCHE |
| 185 | FBN1 | chr15 | ENST00000316623 | p.M1034I | KIIPSLCTHGK | KMIPSLCTHGK |
| 186 | FBXO4 | chr5 | ENST00000281623 | p.E302Q | KRHQWQDEF | KRHEWQDEF |
| 187 | FCSK | chr16 | ENST00000288078 | p.E114Q | LPVQNPEAPVEA | LPVENPEAPVEA |
| 188 | FCSK | chr16 | ENST00000288078 | p.E208Q | YYQGTEAQI | YYQGTEAEI |
| 189 | FER1L6 | chr8 | ENST00000522917 | p.S149F | IIKIFVFHHK | IIKISVFHHK |
| 190 | FES | chr15 | ENST00000328850 | p.E304D | TVDSVQHTLTSV | TVESVQHTLTSV |
| 191 | FGA | chr4 | ENST00000403106 | p.G381S | SVSSSTGQWHS | SVSGSTGQWHS |
| 192 | FGD2 | chr6 | ENST00000274963 | p.E627D | YTFKAETEDLK | YTFKAETEELK |
| 193 | FGF3 | chr11 | ENST00000334134 | p.E150K | RRQPSAKRL | RRQPSAERL |
| 194 | FKBP14 | chr7 | ENST00000222803 | p.D152N | DLNDNWKLSK | DLNDDWKLSK |
| 195 | FKBP15 | chr9 | ENST00000238256 | p.S881C | AACDPSEKVKK | AASDPSEKVKK |
| 196 | FLNA | chrX | ENST00000360319 | p.E2056Q | TFQPAEFII | TFEPAEFII |
| 197 | FLOT2 | chr17 | ENST00000394908 | p.E315Q | AQAQAEKIRK | AQAEAEKIRK |
| 198 | FMN1 | chr15 | ENST00000334528 | p.V582I | IQTDRETFLK | VQTDRETFLK |
| 199 | FMNL2 | chr2 | ENST00000288670 | p.R177K | RSIEDLHK | RSIEDLHR |
| 200 | FMO1 | chr1 | ENST00000354841 | p.I499M | MTQWDRTFKVMK | MTQWDRTFKVIK |
| 201 | FNDC1 | chr6 | ENST00000297267 | p.F433L | LQPGERYLLK | LQPGERYLFK |
| 202 | FOLH1 | chr11 | ENST00000256999 | p.F32L | LFLLGFLF | FFLLGFLF |
| 203 | FOXL1 | chr16 | ENST00000320241 | p.P338A | FPLQVADTV | FPLQVPDTV |
| 204 | FREM2 | chr13 | ENST00000280481 | p.E2021D | YSVDDSAGY | YSVDESAGY |
| 205 | FUT4 | chr11 | ENST00000358752 | p.A227V | RVSYGEAQAVLF | RASYGEAQAVLF |
| 206 | FYCO1 | chr3 | ENST00000296137 | p.E59D | QFDQKDKATLLG | QFDQKEKATLLG |
| 207 | G3BP1 | chr5 | ENST00000356245 | p.F124L | KLYVHNDIFR | KFYVHNDIFR |
| 208 | GABRR2 | chr6 | ENST00000402938 | p.V321I | ASMPRVSYIK | ASMPRVSYVK |
| 209 | GABRR2 | chr6 | ENST00000402938 | p.F263L | RRHIFLFLL | RRHIFFFLL |
| 210 | GAD1 | chr2 | ENST00000358196 | p.F188L | VRTGHPRFL | VRTGHPRFF |

| Number | HUGO Symbol | Chromosome | Transcript ID | HGVSp | MT Epitope Seq | WT Epitope Seq |
|---|---|---|---|---|---|---|
| 211 | GALNT16 | chr14 | ENST00000337827 | p.D311Y | FNHLGKYYA | FNHLGKYDA |
| 212 | GALR1 | chr18 | ENST00000299727 | p.I79M | SMADLAYLLF | SIADLAYLLF |
| 213 | GATM | chr15 | ENST00000396659 | p.S46F | STQAATASFR | STQAATASSR |
| 214 | GCNT2 | chr6 | ENST00000265012 | p.E332Q | IKWSDMQDR | IKWSDMEDR |
| 215 | GFRA1 | chr10 | ENST00000369236 | p.D298H | TPNYIHSSSL | TPNYIDSSSL |
| 216 | GFRA3 | chr5 | ENST00000274721 | p.F304V | TPNVVSNV | TPNFVSNV |
| 217 | GIMAP7 | chr7 | ENST00000313543 | p.G15R | RSLRIVLVRK | RSLRIVLVGK |
| 218 | GIMAP8 | chr7 | ENST00000307271 | p.E539K | LVFQLGRFTK | LVFQLGRFTE |
| 219 | GINS3 | chr16 | ENST00000318129 | p.E172D | ARLDEMDRG | ARLDEMERG |
| 220 | GJB1 | chrX | ENST00000361726 | p.T130I | KVHGILWWTY | KVHGTLWWTY |
| 221 | GK2 | chr4 | ENST00000358842 | p.E77K | KLNIDISNIK | ELNIDISNIK |
| 222 | GLI2 | chr2 | ENST00000361492 | p.A1405S | YARATGHAMASM | YARATGHAMAAM |
| 223 | GLIPR1L2 | chr12 | ENST00000378692 | p.E202Q | KKQKEEMEM | KKEKEEMEM |
| 224 | GLRX3 | chr10 | ENST00000331244 | p.S94F | FLFFKNFQK | FLFFKNSQK |
| 225 | GMEB1 | chr1 | ENST00000294409 | p.R191K | FDLLISSAKA | FDLLISSARA |
| 226 | GNB2 | chr7 | ENST00000303210 | p.S161C | SCGDTTCAL | SSGDTTCAL |
| 227 | GP5 | chr3 | ENST00000401815 | p.G59A | HILLFAMGR | HILLFGMGR |
| 228 | GPA33 | chr1 | ENST00000367868 | p.S232F | SPFMNV | SPSMNV |
| 229 | GPR162 | chr12 | ENST00000311268 | p.S260W | SSLDGWESAK | SSLDGSESAK |
| 230 | GPRIN2 | chr10 | ENST00000374314 | p.R309S | LVPEPGSSTK | LVPEPGSRTK |
| 231 | GSDMB | chr17 | ENST00000360317 | p.Q116H | GSFQGFHHHK | GSFQGFHHQK |
| 232 | GTDC1 | chr2 | ENST00000409214 | p.D223N | CNTHCGLDTA | CDTHCGLDTA |
| 233 | GTPBP8 | chr3 | ENST00000295864 | p.E35K | SQAFAKVLRLPK | SQAFAEVLRLPK |
| 234 | GUCY1A1 | chr4 | ENST00000296518 | p.S565Y | YPHGEPIKM | SPHGEPIKM |
| 235 | HBD | chr11 | ENST00000643122 | p.S51C | LSCPDAVMGNPK | LSSPDAVMGNPK |
| 236 | HCFC2 | chr12 | ENST00000229330 | p.P124A | PACPRLGHSFSL | PPCPRLGHSFSL |
| 237 | HDAC5 | chr17 | ENST00000336057 | p.P817A | RYDNGNFFAG | RYDNGNFFPG |
| 238 | HELQ | chr4 | ENST00000295488 | p.D81H | SPECLVLGGGH | SPECLVLGGGD |
| 239 | HERC1 | chr15 | ENST00000443617 | p.Q4794E | ISQRFEIMK | ISQRFQIMK |
| 240 | HERC1 | chr15 | ENST00000443617 | p.F4757L | QWLWHTLEEF | QWFWHTLEEF |
| 241 | HFM1 | chr1 | ENST00000370425 | p.E1289D | VTSFSTDTDK | VTSFSTDTEK |
| 242 | HIP1 | chr7 | ENST00000336926 | p.E328Q | ASSPDSEPVLQK | ASSPDSEPVLEK |
| 243 | HIPK3 | chr11 | ENST00000379016 | p.P523R | TLNHRFVNMK | TLNHPFVNMK |
| 244 | HT1H2AH | chr6 | ENST00000377459 | p.R30P | FPVGPVHRL | FPVGRVHRL |
| 245 | HT1H3C | chr6 | ENST00000612966 | p.E98K | AVMALQEACK | AVMALQEACE |
| 246 | HIVEP2 | chr6 | ENST00000367603 | p.D2437Y | YPSSEKSQL | DPSSEKSQL |
| 247 | HK1 | chr10 | ENST00000359426 | p.G179V | SVVEGADVVK | SGVEGADVVK |
| 248 | HKDC1 | chr10 | ENST00000354624 | p.E672K | MTCGYEDPNCK | MTCGYEDPNCE |
| 249 | HSD17B13 | chr4 | ENST00000302219 | p.P224A | ASYINIFLR | PSYINIFLR |
| 250 | HSPA2 | chr14 | ENST00000247207 | p.E107Q | QYKGETKTFF | EYKGETKTFF |
| 251 | IDH1 | chr2 | ENST00000345146 | p.D347H | RAKLHNNKELAF | RAKLDNNKELAF |
| 252 | IFT172 | chr2 | ENST00000260570 | p.E1608Q | RFLDLTDAIQE | RFLDLTDAIEE |
| 253 | IFT88 | chr13 | ENST00000319980 | p.D684H | HTYKDTK | DTYKDTK |
| 254 | IGKC | chr2 | ENST00000390237 | p.K100N | SSPVTNSFNR | SSPVTKSFNR |
| 255 | IGSF10 | chr3 | ENST00000282466 | p.V568M | YRITMVEPL | YRITVVEPL |
| 256 | IKZF3 | chr17 | ENST00000346872 | p.R410W | LSWARNGMPLLK | LSRARNGMPLLK |
| 257 | IL17D | chr13 | ENST00000304920 | p.E40Q | RPEELLQQL | RPEELLEQL |
| 258 | IL7R | chr5 | ENST00000303115 | p.L382F | APIFSSSRSL | APILSSSRSL |
| 259 | INSM1 | chr20 | ENST00000310227 | p.F231L | KPKAIRKLHL | KPKAIRKLHF |
| 260 | IQCG | chr3 | ENST00000265239 | p.E44Q | IPKETDIQI | IPKETDIEI |
| 261 | IQSEC2 | chrX | ENST00000375365 | p.S355L | LPPVPPPVPL | LPPVPPPVPS |
| 262 | IRAK3 | chr12 | ENST00000261233 | p.S12T | GRALTAHTLL | GRALSAHTLL |
| 263 | IRS2 | chr13 | ENST00000375856 | p.C409Y | TLSGGYGGRGSK | TLSGGCGGRGSK |

| Number | HUGO Symbol | Chromosome | Transcript ID | HGVSp | MT Epitope Seq | WT Epitope Seq |
|---|---|---|---|---|---|---|
| 264 | ITCH | chr20 | ENST00000262650 | p.L790F | DFNDWQRHA | DLNDWQRHA |
| 265 | ITGA1 | chr5 | ENST00000650673 | p.R259G | GIDTRHSRK | GIDTARRHSRK |
| 266 | ITGA1 | chr5 | ENST00000282588 | p.E260G | GAFTERARR | EAFTERARR |
| 267 | ITGA5 | chr12 | ENST00000293379 | p.E913Q | CPQAECFRL | CPEAECFRL |
| 268 | ITGB4 | chr17 | ENST00000200181 | p.L17F | PWARLLLAAF | PWARLLLAAL |
| 269 | ITGB6 | chr2 | ENST00000283249 | p.R489C | HPGHMGPC | HPGHMGPR |
| 270 | ITPR2 | chr12 | ENST00000381340 | p.I1277M | MRHMFMNNY | MRHIFMNNY |
| 271 | JMJD1C | chr10 | ENST00000542921 | p.F2271L | VRTCTLIQL | VRTCTLIQF |
| 272 | KANK4 | chr1 | ENST00000371153 | p.Q466H | NHSPAERVL | NQSPAERVL |
| 273 | KAT5 | chr11 | ENST00000341318 | p.I474M | RPQITMNEI | RPQITINEI |
| 274 | KCND1 | chrX | ENST00000218176 | p.P604H | IPTHPANTPD | IPTPPANTPD |
| 275 | KCTD4 | chr13 | ENST00000379108 | p.D74N | CPFDANGHYF | CPFDADGHYF |
| 276 | KIAA0319 | chr6 | ENST00000378214 | p.S264C | QLQEQSSNSCGK | QLQEQSSNSSGK |
| 277 | KIF11 | chr10 | ENST00000260731 | p.E489Q | STQEKLHDAASK | STEEKLHDAASK |
| 278 | KIF13B | chr8 | ENST00000524189 | p.F1752L | GSIGGKQYLR | GSIGGKQYFR |
| 279 | KIF16B | chr20 | ENST00000354981 | p.E722K | KKFQIFQEL | EKFQIFQEL |
| 280 | KIF1B | chr1 | ENST00000263934 | p.L174F | LRVREHPLF | LRVREHPLL |
| 281 | KIF3C | chr2 | ENST00000264712 | p.E190Q | NVKQIEHVM | NVKEIEHVM |
| 282 | KIT | chr4 | ENST00000288135 | p.E839K | KWMAPKSIF | KWMAPESIF |
| 283 | KLB | chr4 | ENST00000257408 | p.R58T | FSGDGTAIWSK | FSGDGRAIWSK |
| 284 | KLF17 | chr1 | ENST00000372299 | p.Q121E | RMSPPEQEMTIF | RMSPPQQEMTIF |
| 285 | KLK1 | chr19 | ENST00000301420 | p.S242F | KPFVAVRVL | KPSVAVRVL |
| 286 | KLLN | chr10 | ENST00000445946 | p.R160T | LTERGETVPK | LTERGERVPK |
| 287 | KNL1 | chr15 | ENST00000346991 | p.S447C | SIYSNPCIQGCK | SIYSNPSIQGCK |
| 288 | KNTC1 | chr12 | ENST00000333479 | p.R578T | HRANFESTF | HRANFESRF |
| 289 | KPRP | chr1 | ENST00000606109 | p.P258T | LPTPRRLQL | LPPPRRLQL |
| 290 | L1TD1 | chr1 | ENST00000498273 | p.E614K | KEADLTEETK | KEADLTEETE |
| 291 | LCOR | chr10 | ENST00000286067 | p.L925F | SSLQAERFKK | SSLQAERLKK |
| 292 | LCOR | chr10 | ENST00000286067 | p.E752K | KAAQVNPIMPK | EAAQVNPIMPK |
| 293 | LCOR | chr10 | ENST00000286067 | p.S482I | TIIDSLEENLDK | TSIDSLEENLDK |
| 294 | LCOR | chr10 | ENST00000286067 | p.Q855H | HKCSPVQML | QKCSPVQML |
| 295 | LCOR | chr10 | ENST00000286067 | p.H737D | YNLRHADSLGSL | YNLRHAHSLGSL |
| 296 | LLGL2 | chr17 | ENST00000167462 | p.Q411H | RHNAHFSTM | RQNAHFSTM |
| 297 | LONP2 | chr16 | ENST00000285737 | p.G403E | FHRIALGEV | FHRIALGGV |
| 298 | LPA | chr6 | ENST00000316300 | p.R1692P | TPCSDTEGTVVA | TRCSDTEGTVVA |
| 299 | LPIN3 | chr20 | ENST00000632009 | p.L387V | VPSLDSENAA | LPSLDSENAA |
| 300 | LRCH1 | chr13 | ENST00000311191 | p.E111K | QADLSKNRLVK | QADLSKNRLVE |
| 301 | LRFN5 | chr14 | ENST00000298119 | p.H126Y | MFSGLSNLYHLI | MFSGLSNLHHLI |
| 302 | LRP1 | chr12 | ENST00000243077 | p.S2131F | FVPLRTGIGV | SVPLRTGIGV |
| 303 | LRP11 | chr6 | ENST00000239367 | p.S179G | GGYSSYSLSR | SGYSSYSLSR |
| 304 | LRRC40 | chr1 | ENST00000370952 | p.D178N | LSNLENLDL | LSNLEDLDL |
| 305 | LRRC41 | chr1 | ENST00000343304 | p.E350Q | ATSHQAPGTK | ATSHEAPGTK |
| 306 | LTBP4 | chr19 | ENST00000204005 | p.K66N | SPSVRNRQVSL | SPSVRKRQVSL |
| 307 | LYG1 | chr2 | ENST00000308528 | p.L66V | MPYLVKYQP | MPYLLKYQP |
| 308 | MACF1 | chr1 | ENST00000671089 | p.E698K | LLSLKNHPAK | LLSLENHPAK |
| 309 | MAFG | chr17 | ENST00000357736 | p.V70M | ASCRMKRVTQK | ASCRVKRVTQK |
| 310 | MAGEE1 | chrX | ENST00000361470 | p.S259F | TRDEGPSTF | TRDEGPSTS |
| 311 | MAP1LC3B | chr16 | ENST00000268607 | p.E18Q | RVQDVRLIR | RVEDVRLIR |
| 312 | MAP2K5 | chr15 | ENST00000178640 | p.R282T | TYLWSLKILHTD | TYLWSLKILHRD |
| 313 | MAP4 | chr3 | ENST00000360240 | p.D931N | ATNTSAPNLK | ATNTSAPDLK |
| 314 | MAP4K5 | chr14 | ENST00000013125 | p.E823Q | GSDRVVVLQSR | GSDRVVVLESR |
| 315 | MAP7D3 | chrX | ENST00000316077 | p.S174C | SAMANCESK | SAMANSESK |
| 316 | MAP9 | chr4 | ENST00000311277 | p.S372F | STNNRASSAFAR | STNNRASSASAR |

| Number | HUGO Symbol | Chromosome | Transcript ID | HGVSp | MT Epitope Seq | WT Epitope Seq |
|---|---|---|---|---|---|---|
| 317 | MAPK8IP1 | chr11 | ENST00000241014 | p.E334K | SISEEEKGF | SISEEEEGF |
| 318 | MD1 | chr3 | ENST00000337774 | p.S478C | QPFCGGCLL | QPFCGGSLL |
| 319 | MAST2 | chr1 | ENST00000361297 | p.E1440K | KKKLPPREV | KKELPPREV |
| 320 | MAST4 | chr5 | ENST00000403666 | p.E615K | VISDEINWPK | VISDEINWPE |
| 321 | MAZ | chr16 | ENST00000219782 | p.E181Q | AVAPVASALQK | AVAPVASALEK |
| 322 | MAZ | chr16 | ENST00000219782 | p.K344N | KPYNCSHCGNSF | KPYNCSHCGKSF |
| 323 | MBOAT2 | chr2 | ENST00000305997 | p.Q495H | GHNSFSTTN | GQNSFSTTN |
| 324 | MCM2 | chr3 | ENST00000265056 | p.E862K | AQQDTIEVPK | AQQDTIEVPE |
| 325 | MDH1B | chr2 | ENST00000374412 | p.D94H | LYYHVTSSM | LYYDVTSSM |
| 326 | MED16 | chr19 | ENST00000312090 | p.S362L | ILLTNTDLK | ISLTNTDLK |
| 327 | MED24 | chr17 | ENST00000394126 | p.K346N | KLNKYSHGDK | KLKKYSHGDK |
| 328 | MED24 | chr17 | ENST00000578901 | p.E48Q | GFGEVEQVL | GFGEVEEVL |
| 329 | MEPE | chr4 | ENST00000361056 | p.E512K | RRDDSSKSS | RRDDSSESS |
| 330 | MAP1 | chr4 | ENST00000296411 | p.E234K | GYHGDLNKTF | GYHGDLNETF |
| 331 | MFSD6 | chr2 | ENST00000281416 | p.S183F | LPTNFSFTSF | LPTNSSFTSF |
| 332 | MGA | chr15 | ENST00000566586 | p.G615V | RVRPRKLKLCK | RGRPRKLKLCK |
| 333 | MGA | chr15 | ENST00000566586 | p.Q901E | SRKAKSENR | SRKAKSQNR |
| 334 | MID1 | chrX | ENST00000616003 | p.D300H | RRERKLTLH | RRERKLTLD |
| 335 | MIIP | chr1 | ENST00000235332 | p.P384R | SVPQVPRPHVRR | SVPQVPRPHVPR |
| 336 | MLANA | chr9 | ENST00000381477 | p.H81D | DRDSKVSLQ | HRDSKVSLQ |
| 337 | MMP1 | chr11 | ENST00000315274 | p.E135K | RADVDHAIKK | RADVDHAIEK |
| 338 | MOV10L1 | chr22 | ENST00000262794 | p.E523K | LTFQPLLAK | LTFQPLLAE |
| 339 | MOV10L1 | chr22 | ENST00000262794 | p.D502H | IPHRLRKCV | IPDRLRKCV |
| 340 | MPEG1 | chr11 | ENST00000361050 | p.G647R | RRAMNVIHR | RRAMNVIHG |
| 341 | MROH1 | chr8 | ENST00000528919 | p.E671Q | VRKHLQQLL | VRKHLQELL |
| 342 | MROH2B | chr5 | ENST00000399564 | p.E1109K | KTLWKAK | KTLWKAE |
| 343 | MROH8 | chr20 | ENST00000343811 | p.S149T | KKGSSTIEL | KKGSSSIEL |
| 344 | MRTFB | chr16 | ENST00000573051 | p.E105Q | GPMELVQKNIL | GPMELVEKNIL |
| 345 | MT3 | chr16 | ENST00000200691 | p.E55Q | GQAAEAEAEK | GEAAEAEAEK |
| 346 | MTERF1 | chr7 | ENST00000351870 | p.N16H | QTSISKGLHY | QTSISKQY |
| 347 | MTRF1L | chr6 | ENST00000367230 | p.E77K | KLLNEKEKRK | KLLNEKEKRE |
| 348 | MX2 | chr21 | ENST00000330714 | p.S611C | MKLNCHFPS | MKLNSHFPS |
| 349 | MYH15 | chr3 | ENST00000273353 | p.D1044N | QVNELEGAL | QVDELEGAL |
| 350 | MYLPF | chr16 | ENST00000322861 | p.E65K | AAMGRLNVKNK | AAMGRLNVKNE |
| 351 | MYO18B | chr22 | ENST00000335473 | p.E1528Q | AQMQNEFLRK | AQMENEFLRK |
| 352 | MYO9A | chr15 | ENST00000356056 | p.H526D | LLNSKDLEDNTK | LLNSKDLEHNTK |
| 353 | NAB2 | chr12 | ENST00000300131 | p.G372D | HPEELGDPPL | HPEELGGPPL |
| 354 | NAGA | chr22 | ENST00000396398 | p.M302I | ILQNPLIIK | ILQNPLMIK |
| 355 | NAT10 | chr11 | ENST00000257829 | p.L13V | IVIENGVAER | ILIENGVAER |
| 356 | NBEAL1 | chr2 | ENST00000434469 | p.E189K | VGKPAKMRSGQL | VGKPAEMRSGQL |
| 357 | NDUFV3 | chr21 | ENST00000354250 | p.D109Y | SVLFTYEGVPK | SVLFTDEGVPK |
| 358 | NDUFV3 | chr21 | ENST00000354250 | p.E257Q | SQVDEQFLK | SQVDEEFLK |
| 359 | NDUFV3 | chr21 | ENST00000354250 | p.E195Q | HSFQNRAPR | HSFENRAPR |
| 360 | NDUFV3 | chr21 | ENST00000354250 | p.E222Q | ITDPQKPHQPK | ITDPEKPHQPK |
| 361 | NDUFV3 | chr21 | ENST00000354250 | p.Q374H | EDHIPPSNL | EDQIPPSNL |
| 362 | NEDD4 | chr15 | ENST00000338963 | p.R673I | RIGSLQAYTF | RRGSLQAYTF |
| 363 | NEK1 | chr4 | ENST00000439128 | p.K347N | NRVNTGEER | KRVNTGEER |
| 364 | NEK8 | chr17 | ENST00000268766 | p.Q49H | AAHNECQVLK | AAQNECQVLK |
| 365 | NR3C2 | chr4 | ENST00000358102 | p.K977T | ATPLYFHRK | AKPLYFHRK |
| 366 | NRG2 | chr5 | ENST00000358522 | p.R836T | STHSRGPPPRAK | SRHSRGPPPRAK |
| 367 | NRG2 | chr5 | ENST00000358522 | p.E529K | HTWSLERSK | HTWSLERSE |
| 368 | NT5C2 | chr10 | ENST00000343289 | p.F157L | FYILNTLLNL | FYILNTLFNL |
| 369 | NT5DC4 | chr2 | ENST00000327581 | p.R360K | ERLEELKKL | ERLEELKRL |

| Number | HUGO Symbol | Chromosome | Transcript ID | HGVSp | MT Epitope Seq | WT Epitope Seq |
|---|---|---|---|---|---|---|
| 370 | NUP214 | chr9 | ENST00000359428 | p.E32K | RIFDSPKELPK | RIFDSPEELPK |
| 371 | NXF1 | chr11 | ENST00000530875 | p.E4K | MADKGKSYSV | MADEGKSYSV |
| 372 | NXF1 | chr11 | ENST00000294172 | p.E4K | KGKSYSEHD | EGKSYSEHD |
| 373 | NYAP2 | chr2 | ENST00000272907 | p.A235V | SPVGDPEEEEPV | SPAGDPEEEEPV |
| 374 | OMD | chr9 | ENST00000375550 | p.H356D | SYIFFCFPDI | SYIFFCFPHI |
| 375 | OOSP1 | chr11 | ENST00000644529 | p.S109C | SRDSTVRCE | SRDSTVRSE |
| 376 | OR13C8 | chr9 | ENST00000335040 | p.M199I | ISITGSNLI | ISMTGSNLI |
| 377 | OR2L13 | chr1 | ENST00000358120 | p.H127Y | RYLAICL | RYLAICHSL |
| 378 | OR51B5 | chr11 | ENST00000300773 | p.L61V | MYFFVAMLAA | MYFFLAMLAA |
| 379 | OR51E1 | chr11 | ENST00000396952 | p.K167T | LPVFITQLPF | LPVFIKQLPF |
| 380 | OR51I2 | chr11 | ENST00000341449 | p.E139K | RYATVLTTKVI | RYATVLTTEVI |
| 381 | OR6C68 | chr12 | ENST00000548615 | p.Q195H | EHMVVASAVLTF | EQMVVASAVLTF |
| 382 | OSBPL1A | chr18 | ENST00000319481 | p.S527C | ALCNGIKK | ALSNGIKK |
| 383 | PAFAH1B2 | chr11 | ENST00000304808 | p.R88G | LPGGEKPNPL | LPRGEKPNPL |
| 384 | PAM | chr5 | ENST00000304400 | p.H459Y | AILVRDRIYK | AILVRDRIHK |
| 385 | PAPOLG | chr2 | ENST00000238714 | p.D207N | VTNEILHLVPNK | VTDEILHLVPNK |
| 386 | PAQR6 | chr1 | ENST00000623241 | p.S14F | ASFSPPTCLK | ASSSPPTCLK |
| 387 | PARP1 | chr1 | ENST00000366794 | p.E619K | AIEHFMKLYK | AIEHFMKLYE |
| 388 | PATZ1 | chr22 | ENST00000215919 | p.G298D | GILPCGLCDK | GILPCGLCGK |
| 389 | PCDH10 | chr4 | ENST00000264360 | p.L268H | SPPGTHVIQL | SPPGTLVIQL |
| 390 | PCDH18 | chr4 | ENST00000344876 | p.E678Q | LKCMIFQYA | LKCMIFEYA |
| 391 | PCDHGA5 | chr5 | ENST00000518069 | p.D510N | SINSNTGVLY | SINSDTGVLY |
| 392 | PCDHGA5 | chr5 | ENST00000518069 | p.T628I | RIARALLDR | RTARALLDR |
| 393 | PCDHGC4 | chr5 | ENST00000306593 | p.E534K | QTQTLQFK | QTQTLQFE |
| 394 | PDE4C | chr19 | ENST00000594465 | p.R20C | RLSRSCGRHSMT | RLSRSRGRHSMT |
| 395 | PER3 | chr1 | ENST00000361923 | p.E256Q | TVVQKIHSGY | TVVEKIHSGY |
| 396 | PEX1 | chr7 | ENST00000248633 | p.E484Q | LVISQEEFIK | LVISEEEFIK |
| 397 | PEX11A | chr15 | ENST00000300056 | p.L80V | SIHATDVVPR | SIHATDLVPR |
| 398 | FX | chrX | ENST00000379374 | p.D424H | LPYVVGKMFVHV | LPYVVGKMFVDV |
| 399 | PHLDB1 | chr11 | ENST00000361417 | p.R1020C | LPCNLAATL | LPRNLAATL |
| 400 | PICALM | chr11 | ENST00000393346 | p.Q413E | HPMSTASEVA | HPMSTASQVA |
| 401 | PIEZO2 | chr18 | ENST00000503781 | p.D1104H | TRLHLHDGL | TRLHLDDGL |
| 402 | PIGG | chr4 | ENST00000453061 | p.S146N | SPALLEDNV | SPALLEDSV |
| 403 | PIK3CA | chr3 | ENST00000643187 | p.C420R | RPLAWGNINL | CPLAWGNINL |
| 404 | PKD1L1 | chr7 | ENST00000289672 | p.V1481L | LHYNLQSSL | LHYNLQSSV |
| 405 | PLCB1 | chr20 | ENST00000338037 | p.Q627H | NFHTMDLAM | NFQTMDLAM |
| 406 | PLEKHH2 | chr2 | ENST00000282406 | p.S307C | SRCTSTLCS | SRCTSTLSS |
| 407 | PLEKHJ1 | chr19 | ENST00000326631 | p.R49W | YFWTDEAEPVGA | YFRTDEAEPVGA |
| 408 | PLEKHS1 | chr10 | ENST00000369310 | p.D431H | MESHWCRDSKTA | MESDWCRDSKTA |
| 409 | PLSCR4 | chr3 | ENST00000354952 | p.E224K | GVTIGFVAK | GVTIGFVAE |
| 410 | PM20D2 | chr6 | ENST00000275072 | p.E36Q | AAQRLGALSRAI | AAERLGALSRAI |
| 411 | PMFBP1 | chr16 | ENST00000237353 | p.R881Q | LYQGNDQIM | LYRGNDQIM |
| 412 | PMS1 | chr2 | ENST00000409593 | p.L477V | IVNAILNRNAK | ILNAILNRNAK |
| 413 | PODXL2 | chr3 | ENST00000342480 | p.E166Q | PREEEEQEE | PREEEEEEE |
| 414 | POLA1 | chrX | ENST00000379059 | p.A1192G | RGYAPEQLQK | RAYAPEQLQK |
| 415 | POLD2 | chr7 | ENST00000452185 | p.F403L | VYLCGNTPSF | VYFCGNTPSF |
| 416 | POLR3A | chr10 | ENST00000372371 | p.E513Q | LPQTQEAKA | LPQTEEAKA |
| 417 | POU2F2 | chr19 | ENST00000389341 | p.D183H | HPEEPSHL | HPEEPSDL |
| 418 | PPP1R12B | chr1 | ENST00000290419 | p.E169Q | ERRALQRKM | ERRALERKM |
| 419 | PPP1R9B | chr17 | ENST00000612501 | p.E216K | TVSQLSAVFK | TVSQLSAVFE |
| 420 | PRAMEF1 | chr1 | ENST00000332296 | p.E252Q | YTSDNELQGR | YTSDNELEGR |
| 421 | PRDM15 | chr21 | ENST00000269844 | p.E519K | TTSRDIPPGTK | TTSRDIPPGTE |
| 422 | PRKCE | chr2 | ENST00000306156 | p.D672H | FFKEIHWVL | FFKEIDWVL |

| Number | HUGO Symbol | Chromosome | Transcript ID | HGVSp | MT Epitope Seq | WT Epitope Seq |
|---|---|---|---|---|---|---|
| 423 | PX1 | chr1 | ENST00000261454 | p.D265N | FYQIYDSTN | FYQIYDSTD |
| 424 | PZ | chr13 | ENST00000342783 | p.W60C | VLVRCKRAGSYL | VLVRWKRAGSYL |
| 425 | PSD4 | chr2 | ENST00000245796 | p.E959Q | RRGRGRELQ | RRGRGRELE |
| 426 | PSKH1 | chr16 | ENST00000291041 | p.E410Q | VRERQLREL | VRERELREL |
| 427 | PSKH2 | chr8 | ENST00000276616 | p.L275V | QTRLYRKIVK | QTRLYRKILK |
| 428 | PSMD6 | chr3 | ENST00000295901 | p.D85N | KRLNEELED | KRLDEELED |
| 429 | PSMD8 | chr19 | ENST00000215071 | p.R38G | STSGPHFRR | STSRPHFRR |
| 430 | PTBP3 | chr9 | ENST00000334318 | p.E190Q | RIIIQNLFY | RIIIENLFY |
| 431 | PTGES2 | chr9 | ENST00000338961 | p.R52G | AAAGKGSPR | AAARKGSPR |
| 432 | PTGS2 | chr1 | ENST00000367468 | p.S552C | ASIQCLICNNVK | ASIQSLICNNVK |
| 433 | PTPN1 | chr20 | ENST00000371621 | p.F196L | ASFLNFLLK | ASFLNFLFK |
| 434 | PTPN13 | chr4 | ENST00000316707 | p.D360N | NPIYHTREL | DPIYHTREL |
| 435 | PTPN18 | chr2 | ENST00000175756 | p.E456Q | GPRDPPAQWTRV | GPRDPPAEWTRV |
| 436 | PTPN4 | chr2 | ENST00000263708 | p.E615Q | EKLQNEPDF | EKLENEPDF |
| 437 | PTPRB | chr12 | ENST00000261266 | p.S1601L | LYLDTFFSL | KDTFFSL |
| 438 | PTPRE | chr10 | ENST00000254667 | p.H591Y | FYFHGWPEI | FHFHGWPEI |
| 439 | PTPRR | chr12 | ENST00000283228 | p.E409Q | QIDIPRHGTK | EIDIPRHGTK |
| 440 | PTPRU | chr1 | ENST00000345512 | p.R1328W | FRVQNISWL | FRVQNISRL |
| 441 | PTPRU | chr1 | ENST00000460170 | p.R1324W | ISWEGHLLVR | ISREGHLLVR |
| 442 | PYGO1 | chr15 | ENST00000302000 | p.H368D | ASCQKWFDR | ASCQKWFHR |
| 443 | RAB14 | chr9 | ENST00000373840 | p.R95G | ALMVYDITGR | ALMVYDITRR |
| 444 | RABGAP1L | chr1 | ENST00000325589 | p.E320K | ALMNKIQAAK | ALMNEIQAAK |
| 445 | RABGAP1L | chr1 | ENST00000357444 | p.L530V | AMLDRYRIVITK | AMLDRYRILITK |
| 446 | RABGAP1L | chr1 | ENST00000325589 | p.E274Q | QTDDEKDSLKK | ETDDEKDSLKK |
| 447 | RAD50 | chr5 | ENST00000378823 | p.F669L | VYSQLITQL | VYSQFITQL |
| 448 | RAG1 | chr11 | ENST00000299440 | p.E712K | FIFRGTGYDK | FIFRGTGYDE |
| 449 | RANBP2 | chr2 | ENST00000283195 | p.S1819L | APLAFTLGSEM | APSAFTLGSEM |
| 450 | RAPGEF2 | chr4 | ENST00000264431 | p.S307C | RTFLCSPMEVGK | RTFLSSPMEVGK |
| 451 | RBFOX3 | chr17 | ENST00000648001 | p.E124K | GTIVEGRKIK | GTIVEGRKIE |
| 452 | RBMX | chrX | ENST00000320676 | p.D230H | YPSSRHTRDYA | YPSSRDTRDYA |
| 453 | RBP3 | chr10 | ENST00000584701 | p.L826V | TVPQVAGQR | TLPQVAGQR |
| 454 | REV3L | chr6 | ENST00000368802 | p.G257V | IGVNPGLQA | IGGNPGLQA |
| 455 | RGL3 | chr19 | ENST00000380456 | p.L705V | MLRRKEGTRNTV | MLRRKEGTRNTL |
| 456 | RHOBTB3 | chr5 | ENST00000379982 | p.R220T | FHGITPPQL | FHGIRPPQL |
| 457 | RIF1 | chr2 | ENST00000243326 | p.E1447K | RRKEKEKPL | RRKEEEKPL |
| 458 | RIMS1 | chr6 | ENST00000521978 | p.E320Q | ERRESRRLQ | ERRESRRLE |
| 459 | RINT1 | chr7 | ENST00000257700 | p.H320Y | YYFRGNRQTNVL | YHFRGNRQTNVL |
| 460 | RIPOR2 | chr6 | ENST00000259698 | p.S670F | SFDFLNTFDF | SFDFLNTSDF |
| 461 | RMC1 | chr18 | ENST00000269221 | p.F116L | TKNANILGL | TKNANILGF |
| 462 | RMDN1 | chr8 | ENST00000406452 | p.R226T | MPWYQTRIA | MPWYQRRIA |
| 463 | RNF103 | chr2 | ENST00000237455 | p.E484K | FPVESDWDKD | FPVESDWDED |
| 464 | ROPN1 | chr3 | ENST00000184183 | p.S56C | VRERCERVA | VRERSVA |
| 465 | RP1 | chr8 | ENST00000220676 | p.M712I | IIVQDSDSPLK | MIVQDSDSPLK |
| 466 | RPAP2 | chr1 | ENST00000610020 | p.E612Q | IFRTSCLPQ | IFRTSCLPE |
| 467 | RPL9 | chr4 | ENST00000295955 | p.D11H | HIPENVDITLK | DIPENVDITLK |
| 468 | RPS19BP1 | chr22 | ENST00000334678 | p.E17Q | SQAPRDPPGQAK | SEAPRDPPGQAK |
| 469 | RPS6KC1 | chr1 | ENST00000366960 | p.E686Q | FDDVSGTDQGRP | FDDVSGTDEGRP |
| 470 | RRBP1 | chr20 | ENST00000246043 | p.K73N | KTVEKKGKTNK | KTVEKKGKTKK |
| 471 | RTN2 | chr19 | ENST00000245923 | p.L483V | IVGVIGLFTIPL | ILGVIGLFTIPL |
| 472 | RUFY2 | chr10 | ENST00000399200 | p.H105D | LPQEFYEYDA | LPQEFYEYHA |
| 473 | RUFY2 | chr10 | ENST00000466493 | p.H153D | FYEYDALMM | FYEYHALMM |
| 474 | RYR3 | chr15 | ENST00000634891 | p.E3699Q | SVLDLNAFQR | SVLDLNAFER |
| 475 | SAT1 | chrX | ENST00000379270 | p.E48Q | FGQHPFYHCLVA | FGEHPFYHCLVA |

| Number | HUGO Symbol | Chromosome | Transcript ID | HGVSp | MT Epitope Seq | WT Epitope Seq |
|---|---|---|---|---|---|---|
| 476 | SBF2 | chr11 | ENST00000256190 | p.E987Q | QVSPEVVEIFK | EVSPEVVEIFK |
| 477 | SBNO1 | chr12 | ENST00000420886 | p.R952S | SRAKNQRRR | RRAKNQRRR |
| 478 | SBSPON | chr8 | ENST00000297354 | p.R39C | ACFACGWRL | ACFRWRL |
| 479 | SCGB1A1 | chr11 | ENST00000278282 | p.Q28E | SASAEICPSFER | SASAEICPSFQR |
| 480 | SCNN1G | chr16 | ENST00000300061 | p.E348K | FVEDVGTEIK | FVEDVGTEIE |
| 481 | SEC14L2 | chr22 | ENST00000405717 | p.R43Q | LQARSFDLQK | LRARSFDLQK |
| 482 | SEC14L2 | chr22 | ENST00000405717 | p.F113L | GPLDAKGLLL | GPLDAKGLLF |
| 483 | SEC23B | chr20 | ENST00000336714 | p.L45F | VVPLACLLTPFK | VVPLACLLTPLK |
| 484 | SEC24A | chr5 | ENST00000322887 | p.A223G | GGPPPVRGLTPL | GGPPPVRALTPL |
| 485 | SEMA4D | chr9 | ENST00000420101 | p.R18C | ADICFVWEK | ADIRFVWEK |
| 486 | SHL2 | chr22 | ENST00000327678 | p.S232F | RELCAHFIRK | RELCAHSIRK |
| 487 | SF3B3 | chr16 | ENST00000302516 | p.K1191N | KQNNVSEELDR | KQKNVSEELDR |
| 488 | SGCE | chr7 | ENST00000643193 | p.E435Q | TTFQRFQASIMK | TTFQRFEASIMK |
| 489 | SGCE | chr7 | ENST00000428696 | p.E428Q | QVNGIPEERK | EVNGIPEERK |
| 490 | SH2D4A | chr8 | ENST00000519207 | p.Q35E | MREEEIRRW | MREEQIRRW |
| 491 | SH3BP4 | chr2 | ENST00000344528 | p.K700T | TTEWYIGYY | TKEWYIGYY |
| 492 | SH3D19 | chr4 | ENST00000409598 | p.E223K | PTPSGNLAEK | PTPSGNLAEE |
| 493 | SHANK2 | chr11 | ENST00000645599 | p.E174K | SLNRLGGAGK | SLNRLGGAGE |
| 494 | SHANK2 | chr11 | ENST00000409161 | p.G691C | CRDRKGDDK | GRDRKGDDK |
| 495 | SHOC1 | chr9 | ENST00000318737 | p.S1105F | SLFKIGSFSITK | SLFKIGSSSITK |
| 496 | SHOX2 | chr3 | ENST00000389589 | p.H261Y | SYCNVTPLSF | SHCNVTPLSF |
| 497 | SHPK | chr17 | ENST00000225519 | p.Q80H | LPRPHLRSV | LPRPQLRSV |
| 498 | SIN3A | chr15 | ENST00000360439 | p.H87Y | HYPTAVQPH | HHPTAVQPH |
| 499 | SIPA1L3 | chr19 | ENST00000222345 | p.S1144L | TPYTVLPAGA | TPYTVSPAGA |
| 500 | SIPA1L3 | chr19 | ENST00000222345 | p.S1269Y | QYSSHYSSNTL | QYSSHSSSNTL |
| 501 | SKOR1 | chr15 | ENST00000341418 | p.Q232K | GGMEALTTK | GGMEALTTQ |
| 502 | SLC11A2 | chr12 | ENST00000262052 | p.E554K | SISKGLLTEK | SISKGLLTEE |
| 503 | SLC26A10 | chr12 | ENST00000320442 | p.Q256H | RYQVHIVGLL | RYQVQIVGLL |
| 504 | SLC30A8 | chr8 | ENST00000456015 | p.D248N | KPEYKIANPI | KPEYKIADPI |
| 505 | SLC35G6 | chr17 | ENST00000412468 | p.S13C | YLNPPDCTH | YLNPPDSTH |
| 506 | SLC45A3 | chr1 | ENST00000367145 | p.L92P | RPFIWAPSL | RPFIWALSL |
| 507 | SLC46A1 | chr17 | ENST00000618626 | p.F243L | KSTRLLTFR | KSTRLFTFR |
| 508 | SLC8A2 | chr19 | ENST00000236877 | p.E273Q | IIGAQGDPPK | IIGAEGDPPK |
| 509 | SLCO1B1 | chr12 | ENST00000256958 | p.S548F | IQVLNLFFF | IQVLNLFFS |
| 510 | SLCO2A1 | chr3 | ENST00000310926 | p.F557L | AIGVQLLLMR | AIGVQFLLMR |
| 511 | SLCO5A1 | chr8 | ENST00000260126 | p.A685V | RPFVLGMQF | RPFALGMQF |
| 512 | SLFN11 | chr17 | ENST00000308377 | p.E583Q | QIFSRSLRK | EIFSRSLRK |
| 513 | SLFN14 | chr17 | ENST00000415846 | p.E744Q | KVMKQEMKR | KVMKEEMKR |
| 514 | SMARCAL1 | chr2 | ENST00000358207 | p.R668W | PGWINARTRAAL | PGRINARTRAAL |
| 515 | SMARCAL1 | chr2 | ENST00000358207 | p.S905C | ELLEAAECF | ELLEAAESF |
| 516 | SMC2 | chr9 | ENST00000286398 | p.R502T | ATFPNLRFAYK | ARFPNLRFAYK |
| 517 | SMCO1 | chr3 | ENST00000397537 | p.E133Q | LQQGDITAL | LQEGDITAL |
| 518 | SMG1 | chr16 | ENST00000446231 | p.A2786V | MMEGAVSSA | MMEGAASSA |
| 519 | SMG7 | chr1 | ENST00000347615 | p.M1107I | IMHPGPSAL | MMHPGPSAL |
| 520 | SNRNP200 | chr2 | ENST00000323853 | p.T759I | GSASIEVLR | GSASTEVLR |
| 521 | SNX21 | chr20 | ENST00000342644 | p.E51K | AKGLSSRLS | AEGLSSRLS |
| 522 | SNX32 | chr11 | ENST00000308342 | p.E379Q | AQLELKHAK | AELELKHAK |
| 523 | SORD | chr15 | ENST00000267814 | p.G173R | LRHKVLVCGA | LGHKVLVCGA |
| 524 | SOS1 | chr2 | ENST00000402219 | p.E891Q | HTFQQIPSRQK | HTFEQIPSRQK |
| 525 | SPATA2L | chr16 | ENST00000289805 | p.G320R | RPRDLATPESSA | RPGDLATPESSA |
| 526 | SPATA2L | chr16 | ENST00000289805 | p.D234N | YRDLQENEG | YRDLQEDEG |
| 527 | SPATA5 | chr4 | ENST00000274008 | p.R472T | EVEKTVVASL | EVEKRVVASL |
| 528 | SPECC1L | chr22 | ENST00000314328 | p.E434K | QQITQELNSK | QQITQELNSE |

| Number | HUGO Symbol | Chromosome | Transcript ID | HGVSp | MT Epitope Seq | WT Epitope Seq |
|---|---|---|---|---|---|---|
| 529 | SPG11 | chr15 | ENST00000261866 | p.Q2432E | LLKDPETGCCLK | LLKDPQTGCCLK |
| 530 | SPG7 | chr16 | ENST00000643370 | p.G116A | SALPVCAASLRK | SALPVCGASLRK |
| 531 | SPRYD3 | chr12 | ENST00000301463 | p.I161M | QTAQMFFTK | QTAQIFFTK |
| 532 | SPTBN1 | chr2 | ENST00000333896 | p.E637Q | FFWEMAEQE | FFWEMAEEE |
| 533 | SPTSSA | chr14 | ENST00000298130 | p.W32S | LYMLEPSTVF | LYMLEPWERTVF |
| 534 | SRA1 | chr5 | ENST00000336283 | p.S182Y | HRYLMVDHV | HRSLMVDHV |
| 535 | SRPK1 | chr6 | ENST00000361690 | p.E655Q | LPMLELIPQKRA | LPMLELIPEKRA |
| 536 | STAC2 | chr17 | ENST00000584501 | p.S106L | ATLVPLSWCMSR | ATSVPLSWCMSR |
| 537 | STAT5A | chr17 | ENST00000345506 | p.D662N | RSLADRLGNLSY | RSLADRLGDLSY |
| 538 | STOX1 | chr10 | ENST00000298596 | p.D576N | KPINNDFRGHL | KPINDDFRGHL |
| 539 | STXBP5 | chr6 | ENST00000367481 | p.S177L | FTLLGYVIMWNK | FTLSGYVIMWNK |
| 540 | SUCO | chr1 | ENST00000263688 | p.E788D | KSESFSSIDK | KSESFSSIEK |
| 541 | SULT6B1 | chr2 | ENST00000535679 | p.E284Q | EKFKQCLAGTSL | EKFKECLAGTSL |
| 542 | SYT1 | chr12 | ENST00000261205 | p.K322N | MQNGNRLKK | MQNGKRLKK |
| 543 | SYTL4 | chrX | ENST00000276141 | p.P568A | ASKRKTAVMKK | ASKRKTPVMKK |
| 544 | SYVN1 | chr11 | ENST00000307289 | p.F126S | SYAILMTMVL | FYAILMTMVL |
| 545 | SYVN1 | chr11 | ENST00000294256 | p.F126S | LAEDRVDSM | LAEDRVDFM |
| 546 | TAX1BP1 | chr7 | ENST00000265393 | p.P724A | CPMCSEQFPA | CPMCSEQFPP |
| 547 | TBC1D17 | chr19 | ENST00000221543 | p.E76K | SSGGDSCASK | SSGGDSCASE |
| 548 | TBC1D19 | chr4 | ENST00000264866 | p.E313Q | YYFVFQDYL | YYFVFEDYL |
| 549 | TBXA2R | chr19 | ENST00000375190 | p.L262V | LPLVVFIAQT | LPLLVFIAQT |
| 550 | TBXA2R | chr19 | ENST00000375190 | p.S207L | FSMLGGLLV | FSMLGGLSV |
| 551 | TDRD7 | chr9 | ENST00000355295 | p.E1089K | MSKYLIELSK | MSEYLIELSK |
| 552 | TEDC1 | chr14 | ENST00000354560 | p.I23M | LPEAMAAL | LPEAIAAL |
| 553 | TEP1 | chr14 | ENST00000262715 | p.I1824M | GSMSFFQVDGLK | GSISFFQVDGLK |
| 554 | TEX15 | chr8 | ENST00000256246 | p.D2423H | HHFSGQQEN | DHFSGQQEN |
| 555 | TEX15 | chr8 | ENST00000256246 | p.D2495N | CLSNINPET | CLSDINPET |
| 556 | TEX55 | chr3 | ENST00000295622 | p.D104N | LRADNQVNQ | LRADDQVNQ |
| 557 | TFIP11 | chr22 | ENST00000407690 | p.F658L | GLLEKHLFPK | GLLEKHFFPK |
| 558 | TFIP11 | chr22 | ENST00000407690 | p.S691L | WYLGWKSMFL | WYLGWKSMFS |
| 559 | TGFBR3 | chr1 | ENST00000212355 | p.M741I | SIIWAIMQNK | SIIWAMMQNK |
| 560 | THOP1 | chr19 | ENST00000307741 | p.D640H | GVLNSKVGMHYR | GVLNSKVGMDYR |
| 561 | TMC8 | chr17 | ENST00000318430 | p.E19K | VPEPKELWEA | VPEPEELWEA |
| 562 | TMEM185B | chr2 | ENST00000426077 | p.E106D | DRVDRGTHF | DRVERGTHF |
| 563 | MEM189-UBE2V | chr20 | ENST00000341698 | p.F89L | VAGALIADL | VAGALIADF |
| 564 | TMEM266 | chr15 | ENST00000561302 | p.A38S | SPVQLVNFA | APVQLVNFA |
| 565 | TMEM39A | chr3 | ENST00000319172 | p.H352Y | AAYLGKWQK | AAHLGKWQK |
| 566 | TMPRSS15 | chr21 | ENST00000284885 | p.K199N | SPCTDALTCINA | SPCTDALTCIKA |
| 567 | TMPRSS6 | chr22 | ENST00000442782 | p.S428F | LPFSIVTPSLEA | LPSSIVTPSLEA |
| 568 | TNC | chr9 | ENST00000350763 | p.R2029C | LRCKNGREN | LRRKNGREN |
| 569 | TNFRSF25 | chr1 | ENST00000348333 | p.K42N | DFHKNIGLF | DFHKKIGLF |
| 570 | TNPO3 | chr7 | ENST00000265388 | p.L183V | SVLMTCVEK | SLLMTCVEK |
| 571 | TP53 | chr17 | ENST00000269305 | p.I232T | GSDCTTTHYNY | GSDCTTIHYNY |
| 572 | TP53TG5 | chr20 | ENST00000372726 | p.R263K | HPYKVDVTWTKA | HPYKVDVTWTRA |
| 573 | TRAF3IP2 | chr6 | ENST00000340026 | p.Q247K | FPKFEPQRYPA | FPQFEPQRYPA |
| 574 | TRAF5 | chr1 | ENST00000261464 | p.D502H | KPHPNSSSF | KPDPNSSSF |
| 575 | TRRAP | chr7 | ENST00000355540 | p.E2787K | KAMDKAKKEHK | KAMDKAKKEHE |
| 576 | TSC2 | chr16 | ENST00000350773 | p.E1453D | APSRRGKRVDRD | APSRRGKRVERD |
| 577 | TSC22D4 | chr7 | ENST00000300181 | p.R113P | IPGASGGAG | IRGASGGAG |
| 578 | TSHZ2 | chr20 | ENST00000371497 | p.L290F | SLQDFSVHMIK | SLQDLSVHMIK |
| 579 | TSNARE1 | chr8 | ENST00000520166 | p.R120G | MAGPSTTGA | MAGPSTTRA |
| 580 | TSPOAP1 | chr17 | ENST00000268893 | p.G934W | SPWILIISWL | SPGILIISWL |
| 581 | TTC21B | chr2 | ENST00000243344 | p.Q516K | DIEAAFNNLK | DIEAAFNNLQ |

| Number | HUGO Symbol | Chromosome | Transcript ID | HGVSp | MT Epitope Seq | WT Epitope Seq |
|---|---|---|---|---|---|---|
| 582 | TTC9 | chr14 | ENST00000256367 | p.E189Q | YYLKQARTQ | YYLKEARTQ |
| 583 | TTLL13P | chr15 | ENST00000641754 | p.F636L | RPKNLNWTGEPA | RPKNFNWTGEPA |
| 584 | TXNRD2 | chr22 | ENST00000334363 | p.R31Q | GVRGAAQGAAA[illegible] | GVRGARAAAG |
| 585 | TXNRD2 | chr22 | ENST00000400519 | p.R31Q | VRGAAQGAA | VRGARAA |
| 586 | UBAP2L | chr1 | ENST00000343815 | p.Q588E | YTSQNNAEGPL | YTSQNNAQGPL |
| 587 | UBASH3A | chr21 | ENST00000291535 | p.S398N | FPCNLPRRS | FPCSLPRRS |
| 588 | UBR4 | chr1 | ENST00000375254 | p.L638V | APGEKGNIV | APGEKGNIL |
| 589 | UGT2A2 | chr4 | ENST00000604021 | p.K11N | SIRDFTMPNK | SIRDFTMPKK |
| 590 | UQCRFS1 | chr19 | ENST00000304863 | p.K155T | ITLSDIPEGK | IKLSDIPEGK |
| 591 | UROC1 | chr3 | ENST00000290868 | p.F375L | MNPAVLK | MNPAVFK |
| 592 | USH2A | chr1 | ENST00000307340 | p.R837W | FYLWQNNSF | FYLRQNNSF |
| 593 | USHBP1 | chr19 | ENST00000252597 | p.A630V | SVELNRDLCK | SAELNRDLCK |
| 594 | USP19 | chr3 | ENST00000398888 | p.S377R | LRKRQRQRWGGL | LRKRQSQRWGGL |
| 595 | USP28 | chr11 | ENST00000003302 | p.E417K | IRKLKKEIK | IRKLKEEIK |
| 596 | USP31 | chr16 | ENST00000219689 | p.S377Y | YYDGFHRYF | YYDGFHRSF |
| 597 | USP34 | chr2 | ENST00000453734 | p.E406D | FLMGKSDRK | FLMGKSK |
| 598 | USP37 | chr2 | ENST00000258399 | p.M111I | AVHQNRLPAAIK | AVHQNRLPAAMK |
| 599 | USP47 | chr11 | ENST00000527733 | p.D949N | HSSNTLCNA | HSSDTLCNA |
| 600 | USP51 | chrX | ENST00000500968 | p.S13C | LPSGCGVRWI | LPSGSGVRWI |
| 601 | UTP20 | chr12 | ENST00000261637 | p.Q1948H | VKHILSKVM | VKQILSKVM |
| 602 | VCL | chr10 | ENST00000211998 | p.S252C | LTCWDEDAWASK | LTSWDEDAWASK |
| 603 | VEGFC | chr4 | ENST00000618562 | p.Q327K | FPSKCGANREF | FPSQCGANREF |
| 604 | VLDLR | chr9 | ENST00000382099 | p.R823T | LSIDIGTHSA | LSIDIGRHSA |
| 605 | VPS13A | chr9 | ENST00000360280 | p.P1612A | RACPFLAVK | RACPFLPVK |
| 606 | VPS13B | chr8 | ENST00000357162 | p.S1014C | QACEYASSPVK | QASEYASSPVK |
| 607 | VPS13C | chr15 | ENST00000249837 | p.D1555H | SQKDVFHLK | SQKDVFDLK |
| 608 | VPS13C | chr15 | ENST00000249837 | p.D3529H | RPTGGIVHM | RPTGGIVDM |
| 609 | VPS37D | chr7 | ENST00000324941 | p.R90C | KYQELCEVA | KYQELREVA |
| 610 | VPS45 | chr1 | ENST00000643970 | p.G400V | RVSDLFSPK | RGSDLFSPK |
| 611 | VRTN | chr14 | ENST00000256362 | p.E600Q | ASSEDVQGGPSR | ASSEDVEGGPSR |
| 612 | VSX2 | chr14 | ENST00000261980 | p.E284Q | LPKLDKMQQ | LPKLDKMEQ |
| 613 | WDR4 | chr21 | ENST00000330317 | p.C412R | RPGEATLSR | RPGEATLSC |
| 614 | WNK2 | chr9 | ENST00000297954 | p.E522D | SGFFHDSDVK | SGFFHESDVK |
| 615 | WNK4 | chr17 | ENST00000246914 | p.P87A | SPAPDPPDPA | SPAPDPPDPP |
| 616 | YES1 | chr18 | ENST00000314574 | p.K138N | RSIATGNNGY | RSIATGKNGY |
| 617 | ZC3H10 | chr12 | ENST00000257940 | p.H221Y | YFESYEYSL | HFESYEYSL |
| 618 | ZC3H4 | chr19 | ENST00000253048 | p.G57V | LPDDREDVEL | LPDDREDGEL |
| 619 | ZFHX3 | chr16 | ENST00000268489 | p.S2067L | IPAPPI | IPASAPPI |
| 620 | ZFHX4 | chr8 | ENST00000651372 | p.E330Q | QPLISFLEPK | EPLISFLEPK |
| 621 | ZFHX4 | chr8 | ENST00000651372 | p.R2609K | HRDKRLKTT | HRDKRLRTT |
| 622 | ZFP41 | chr8 | ENST00000520584 | p.R7K | KPAGKKKKTPT | KPAGRKKKTPT |
| 623 | ZFPL1 | chr11 | ENST00000294258 | p.L136V | NWARAGLGLPVI | NWARAGLGLPLI |
| 624 | ZFPM1 | chr16 | ENST00000319555 | p.Q958H | SYSDKGVHTP | SYSDKGVQTP |
| 625 | ZIC4 | chr3 | ENST00000383075 | p.E127Q | RYMRQPIKQQLI | RYMRQPIKQELI |
| 626 | ZIC4 | chr3 | ENST00000383075 | p.L221V | KVFARSENVK | KVFARSENLK |
| 627 | ZIM2 | chr19 | ENST00000593711 | p.E491Q | YRTHTQQRP | YRTHTQERP |
| 628 | ZKSCAN5 | chr7 | ENST00000326775 | p.K528N | SSNRMNYSEVPY | SSKRMNYSEVPY |
| 629 | ZMYND12 | chr1 | ENST00000372565 | p.E271Q | FQNDTGLDEA | FENDTGLDEA |
| 630 | ZNF274 | chr19 | ENST00000326804 | p.D327H | LSSTLEHTL | LSSTLEDTL |
| 631 | ZNF322 | chr6 | ENST00000415922 | p.E34K | KIFIHMHK | KIFIHMHE |
| 632 | ZNF326 | chr1 | ENST00000340281 | p.S155F | NYFSYSSF | NYSSYSSF |
| 633 | ZNF366 | chr5 | ENST00000318442 | p.K85N | MPTNMPYNHPA | MPTKMPYNHPA |
| 634 | ZNF385B | chr2 | ENST00000410066 | p.E329K | GSKHKTMVK | GSKHKTMVE |

| Number | HUGO Symbol | Chromosome | Transcript ID | HGVSp | MT Epitope Seq | WT Epitope Seq |
|---|---|---|---|---|---|---|
| 635 | ZNF41 | chrX | ENST00000313116 | p.E91K | GPWMLEGKA | GPWMLEGEA |
| 636 | ZNF420 | chr19 | ENST00000337995 | p.R75I | SQWEMSDIL | SQWEMSDRL |
| 637 | ZNF432 | chr19 | ENST00000221315 | p.E258K | RIHKRKKSF | RIHKREKSF |
| 638 | ZNF502 | chr3 | ENST00000296091 | p.R119S | LSVSTEESL | LRVSTEESL |
| 639 | ZNF521 | chr18 | ENST00000361524 | p.K983N | HKVTHSNSL | HKVTHSKSL |
| 640 | ZNF541 | chr19 | ENST00000263351 | p.S48L | GPGPSSGSPL | GPGPSSGSPS |
| 641 | ZNF550 | chr19 | ENST00000325134 | p.L182F | FHECDSQQP | LHECDSQQP |
| 642 | ZNF568 | chr19 | ENST00000444991 | p.G363A | SINTAEKPHK | SINTGEKPHK |
| 643 | ZNF644 | chr1 | ENST00000337393 | p.D624H | SFGSPLGLHK | SFGSPLGLDK |
| 644 | ZNF649 | chr19 | ENST00000354957 | p.E378Q | CPQCGQPC | CPECGQPC |
| 645 | ZNF827 | chr4 | ENST00000508784 | p.E233G | TRTEGTMRF | TRTEETMRF |
| 646 | ZSCAN18 | chr19 | ENST00000240727 | p.S259Y | YRLDTEELR | SRLDTEELR |

HUGO: Human Genome Organisation

HGVSp:Human Genome Variation Society (HGVS) nomenclature of protein
