# Supplementary material for: Stimulating T cell responses against patient-derived breast cancer cells with neoantigen peptide-loaded peripheral blood mononuclear cells
Source: Cancer Immunol Immunother. 2024 Feb 13;73(3):43. doi: 10.1007/s00262-024-03627-3 (PMC10864427; doi:10.1007/s00262-024-03627-3)
Supplement: Supplementary file 8 — (PDF 142 kb) [file 262_2024_3627_MOESM8_ESM.pdf]

Supplementary Table S4b. List of 652 nonsynonymous mutations of PC-B-148CA

Number	HUGO Symbol	Chromosome	Transcript ID	HGVSp	MT Epitope Seq	WT Epitope Seq
1	A4GALT	chr22	ENST00000249005	p.M230V	FERRHEFVCM	FERRHEFMALCM
2	ABCA13	chr7	ENST00000435803	p.R4282H	LDLTHVLLRKFR	LDLTRVLLRKFR
3	ABCA2	chr9	ENST00000479446	p.T1092A	YNNKGYHSMPAY	YNNKGYHSMPTY
4	ABCA2	chr9	ENST00000487109	p.Y220C	ARVDPPEVC	ARVDPPEVY
5	ABCC5	chr3	ENST00000334444	p.A495V	PHIKIEMKNVTL	PHIKIEMKNATL
6	ABCG4	chr11	ENST00000619701	p.A417D	LYLHIGDDDSKV	LYLHIGDDASKV
7	ABHD17A	chr19	ENST00000250974	p.G202S	GYSASSGRPS	GYGASSGRPS
8	ABLIM2	chr4	ENST00000341937	p.S399G	SESGRSTPGLSV	SESGRSTPSLSV
9	ABLIM3	chr5	ENST00000309868	p.Y229H	AGGKHHHPTCAR	AGGKHYHPTCAR
10	ACAP3	chr1	ENST00000354700	p.T24M	FRATIDEVEMDV	FRATIDEVETDV
11	ACCS	chr11	ENST00000263776	p.W115R	SRRLSQRDMQRV	SWRLSQRDMQRV
12	ADAMTS13	chr9	ENST00000355699	p.P288H	VWDPPRHQPGSA	VWDPPRPQPGSA
13	ADAMTS17	chr15	ENST00000268070	p.A242T	VETLVVADTDMV	VETLVVADADMV
14	ADAMTS3	chr4	ENST00000286657	p.Y42C	YRECELVTPVST	YREYELVTPVST
15	ADCY10	chr1	ENST00000367848	p.R397H	LLGHNKEINYFM	LLGRNKEINYFM
16	ADCY9	chr16	ENST00000294016	p.T492M	VNMRVGVHMGTV	VNMRVGVHTGTV
17	ADGRA2	chr8	ENST00000412232	p.A617T	FHIKNSVTSI	FHIKNSVASI
18	ADGRG7	chr3	ENST00000273352	p.Y385N	KYACVNWNLS	KYACVYWNLS
19	ADGRL1	chr19	ENST00000340736	p.M78V	DPFQVENVQCYL	DPFQMENVQCYL
20	AFTPH	chr2	ENST00000238856	p.T793A	MNTCASDQFQES	MNTCTSDQFQES
21	AGAP3	chr7	ENST00000397238	p.K420R	LNREWKKKYVTL	LNKEWKKKYVTL
22	AGL	chr1	ENST00000294724	p.M1414V	KKLLGPLGVKTL	KKLLGPLGMKTL
23	AGPAT3	chr21	ENST00000291572	p.Q32H	VINFVHLCTLAL	VINFVQLCTLAL
24	AGRN	chr1	ENST00000379370	p.C479R	FGATRAVKNGQA	FGATCAVKNGQA
25	AICDA	chr12	ENST00000229335	p.R36C	RCDSATSFSLDF	RRDSATSFSLDF
26	AIPL1	chr17	ENST00000381128	p.Q40K	RVSGAPPEK	RVSGAPPEQ
27	AK2	chr1	ENST00000354858	p.R132C	ITGEPLICR	ITGEPLIRR
28	ALKBH6	chr19	ENST00000252984	p.V83M	RYMDKVSNLSLF	RYVDKVSNLSLF
29	AMIGO3	chr3	ENST00000320431	p.P220H	HNNHLPCDCRLY	HNNPLPCDCRLY
30	AMT	chr3	ENST00000273588	p.V190A	QALQAGVADDLR	QVLQAGVADDLR
31	ANGPTL7	chr1	ENST00000376819	p.W11R	MLKKPLSAVTRL	MLKKPLSAVTWL
32	ANK2	chr4	ENST00000357077	p.N709S	LHLAAQEDKVSV	LHLAAQEDKVNV
33	ANKFY1	chr17	ENST00000570535	p.A580V	EAASLTSLVDSV	EAASLTSLADSV
34	ANKIB1	chr7	ENST00000265742	p.Y754C	TWDWECLGF	TWDWEYLGF
35	ANKRD33	chr12	ENST00000340970	p.Q245H	PSSLAIPGAHDR	PSSLAIPGAQDR
36	ANKRD52	chr12	ENST00000267116	p.Q879H	GLRMLLHHQAEV	GLRMLLQHQAEV
37	ANO8	chr19	ENST00000159087	p.L453I	SYLSIFYIGF	SYLSLFYIGF
38	ANPEP	chr15	ENST00000300060	p.Q343K	LPKSDKIGLPDF	LPKSDQIGLPDF
39	AOC1	chr7	ENST00000360937	p.E313D	FRLDGNAVL	FRLEGNAVL
40	APAF1	chr12	ENST00000359972	p.M470T	CTYWYNFLAYHM	CMYWYNFLAYHM
41	APOA2	chr1	ENST00000367990	p.L3P	MKPLAATVLLLT	MKLLAATVLLLT
42	AQP3	chr9	ENST00000645858	p.R187C	LCVPADDRL	LRVPADDRL
43	ARHGAP10	chr4	ENST00000336498	p.R58P	SVAQPKFAHSLR	SVAQRKFAHSLR
44	ARHGAP20	chr11	ENST00000260283	p.R702H	LRRHRHCSEPSI	LRRHRRCSEPSI
45	ARHGEF1	chr19	ENST00000337665	p.R635W	LRLKDYQWRLDL	LRLKDYQRRLDL
46	ARHGEF11	chr1	ENST00000361409	p.T1462M	GTMPVGSFH	GTTPVGSFH
47	ARHGEF15	chr17	ENST00000361926	p.R239H	HRDLRTS	RRDLRTS
48	ARID1A	chr1	ENST00000324856	p.S2256L	FTLYELRLLDIS	FTLYESRLLDIS
49	ARMC12	chr6	ENST00000288065	p.Q7K	KYLGQLDIRKSV	QYLGQLDIRKSV
50	ARNTL2	chr12	ENST00000261178	p.S453R	SEESSRQSCMRV	SEESSRQSCMSV
51	DM	chr1	ENST00000294732	p.K1247E	NTIPDEEVVITY	NTIPDEKVVITY

Number	HUGO Symbol	Chromosome	Transcript ID	HGVSp	MT Epitope Seq	WT Epitope Seq
52	ASXL2	chr2	ENST00000336112	p.R613H	AHFPVSITSPNR	ARFPVSITSPNR
53	ASXL2	chr2	ENST00000672666	p.R84H	SPRAHFPVSITS	SPRARFPVSITS
54	ATAD3B	chr1	ENST00000673477	p.Y438H	LHHMGQHSNKFM	LYHMGQHSNKFM
55	ATP2B3	chrX	ENST00000263519	p.I825T	EASDTILTDDNF	EASDIILTDDNF
56	ATXN1	chr6	ENST00000244769	p.P649L	VLVEYLFFVF	VLVEYPFFVF
57	ATXN3	chr14	ENST00000553570	p.A88V	ICQIVKLTNSCR	ICQIAKLTNSCR
58	AVIL	chr12	ENST00000257861	p.N538S	FASSLSSNDVFL	FASSLNSNDVFL
59	B4GALT6	chr18	ENST00000237019	p.A166V	FNRVMLFNVGFK	FNRAMLFNVGFK
60	BANF1	chr11	ENST00000312175	p.R37M	LEEMGFDKAYVV	LEERGFDKAYVV
61	BBS7	chr4	ENST00000264499	p.N199S	HNGSGGDSGEDL	HNGNGGDSGEDL
62	BCKDHA	chr19	ENST00000269980	p.I78T	QPNVISGIPY	QPNVISGIPIYR
63	BCL9	chr1	ENST00000234739	p.A1366V	MIPGKDRGPVGL	MIPGKDRGPAGL
64	BCLAF1	chr6	ENST00000527613	p.S602P	TPESFIQHIVSL	TSESFIQHIVSL
65	BRAF	chr7	ENST00000288602	p.N355I	FRPADEDHRIQF	FRPADEDHRNQF
66	BRD3	chr9	ENST00000303407	p.D64Y	FYQPVYAIKLNL	FYQPVDAIKLNL
67	BTN3A2	chr6	ENST00000377708	p.D239N	TASISIANPFFR	TASISIADPFFR
68	C17orf67	chr17	ENST00000397861	p.L14P	VLSLTLPTVF	VLSLTLLTVF
69	C2CD3	chr11	ENST00000313663	p.M1645V	SILVERAVHLSL	SILVERAMHLSL
70	C2orf42	chr2	ENST00000264434	p.S317R	MNRSLLPQDAVS	MNSSLLPQDAVS
71	C5orf15	chr5	ENST00000231512	p.N67K	NSHTPKVNAL	NSHTPNVNAL
72	C7	chr5	ENST00000313164	p.C838R	GQSISVTSIRPR	GQSISVTSIRPC
73	CACNB4	chr2	ENST00000534999	p.I396F	ALSPYPTAF	ALSPYPTAI
74	CALCOCO1	chr12	ENST00000548263	p.Q165R	ERNDLMRLKLQL	ERNDLMQLKLQL
75	CAMTA1	chr1	ENST00000303635	p.E1510G	EKVENGFAQLTL	EKVENEFAQLTL
76	CAPN2	chr1	ENST00000295006	p.T684A	FKQLDPENAGTI	FKQLDPENTGTI
77	CASKIN2	chr17	ENST00000321617	p.P884L	LSSVSGPSL	LSSVSGPSP
78	CD3	chr4	ENST00000308394	p.Y197C	ACSTAPGYYSWR	AYSTAPGYYSWR
79	CASTOR2	chr7	ENST00000616305	p.R128H	FILVHERDLPFV	FILVRERDLPFV
80	CBWD1	chr9	ENST00000377447	p.Y236H	YRSMLHTQNIFY	YRSMLYTQNIFY
81	CCDC110	chr4	ENST00000307588	p.C504R	TEEYSKERLKEF	TEEYSKECLKEF
82	CCDC144NL	chr17	ENST00000650066	p.S90L	HRAARLGDVPGV	HRAARSGDVPGV
83	CCDC71	chr3	ENST00000321895	p.R155W	AWGAAVGF	RAAVGF
84	CCKBR	chr11	ENST00000334619	p.T89A	RRLRTVANAFLL	RRLRTVTNAFLL
85	CCN2	chr6	ENST00000367976	p.Y279H	TAKFCGVCTD	YAKFCGVCTD
86	CCNI2	chr5	ENST00000378731	p.V154M	DAFEEVMLWLLR	DAFEEVVLWLLR
87	CD1D	chr1	ENST00000368171	p.R43H	FANSSWTHTDGL	FANSSWTRTDGL
88	CD59	chr11	ENST00000652086	p.H84R	SVVFQALRGIGV	SVVFQALHGIGV
89	CDC27	chr17	ENST00000066544	p.Y646C	YCKQEKFSLAEM	YYKQEKFSLAEM
90	CDC42	chr1	ENST00000652582	p.N57D	FARSAGRQDSSR	FARSAGRQNSSR
91	CDH15	chr16	ENST00000289746	p.P487L	HAPVLAPPL	HAPVLAPPP
92	CDH19	chr18	ENST00000262150	p.A693T	SLQVGPDSTIFR	SLQVGPDSAIFR
93	CDHR1	chr10	ENST00000332904	p.Q713R	KKPHSSRGL	KKPHSSQGL
94	CDHR2	chr5	ENST00000261944	p.L546P	NGELPDRESQAV	NGELLDRESQAV
95	CDHR4	chr3	ENST00000343366	p.R141W	SWQGGGRHGLSR	SRQGGGRHGLSR
96	CDKN2AIP	chr4	ENST00000504169	p.P570H	ESRPVNLHPALK	ESRPVNLPPALK
97	CELSR3	chr3	ENST00000164024	p.C2059R	NGQRHCKEFHYR	NGQCHCKEFHYR
98	CELSR3	chr3	ENST00000164024	p.Y1357C	FSSEELQEQLCV	FSSEELQEQLYV
99	CELSR3	chr3	ENST00000164024	p.A2834V	TVSSVSSVRSGR	TVSSVSSARSGR
100	CEMIP	chr15	ENST00000220244	p.R1099H	FSILSDVHNHLL	FSILSDVHNRLL
101	CEP70	chr3	ENST00000264982	p.F442I	KVEDLLIIVDTM	KVEDLLFIVDTM
102	CGAS	chr6	ENST00000370315	p.Y415C	MKCLLEQLKERF	MKYLLEQLKERF
103	CHAT	chr10	ENST00000337653	p.R117C	KTPIKVPCKM	KTPIKVPRKM
104	CHD1	chr5	ENST00000284049	p.W325R	QYLIKWKGRSHI	QYLIKWKGWSHI

Number	HUGO Symbol	Chromosome	Transcript ID	HGVSp	MT Epitope Seq	WT Epitope Seq
105	CHERP	chr19	ENST00000546361	p.Y897C	YENCRRNKSYSF	YENYRRNKSYSF
106	CHST3	chr10	ENST00000373115	p.L80P	AENASLLSPSEL	AENASLLSLSEL
107	CIC	chr19	ENST00000575354	p.S541P	SASSPAPSS	SASSPASSS
108	CILP2	chr19	ENST00000291495	p.Q655R	LRAPGSAERLQV	LRAPGSAEQLQV
109	CIT	chr12	ENST00000261833	p.R701Q	LEEKHQEAQVSA	LEEKHREAQVSA
110	CIZ1	chr9	ENST00000372938	p.K717R	LRSLEKEIAGQD	LKSLEKEIAGQD
111	CLEC10A	chr17	ENST00000576617	p.V216F	FREMFLKLGRTL	FREMVLKLGRTL
112	CLIP3	chr19	ENST00000360535	p.R105H	KIDVIGNEILHR	KIDVIGNEILRR
113	CLPX	chr15	ENST00000300107	p.P444S	EKYLGFGTSSNL	EKYLGFGTPSNL
114	CLYBL	chr13	ENST00000339105	p.L243M	AKAFGLQAIDMV	AKAFGLQAIDLV
115	CNOT1	chr16	ENST00000317147	p.S1140G	KRVGIEPNF	KRVSIEPNF
116	CNST	chr1	ENST00000366512	p.L318I	SESKTCIGTESS	SESKTCLGTESS
117	CNTNAP1	chr17	ENST00000264638	p.P710S	YWGGSQSGIQRC	YWGGSQPGIQRC
118	COCH	chr14	ENST00000216361	p.D83Y	AYLLLLTCF	ADLLLLTCF
119	COL17A1	chr10	ENST00000650263	p.Y109H	HAHEASSSTRGR	HAYEASSSTRGR
120	COL17A1	chr10	ENST00000393211	p.Y109H	KTHVTRHAH	KTHVTRHAY
121	COL27A1	chr9	ENST00000356083	p.G1604V	GLQGPRVPPGPR	GLQGPRGPPGPR
122	COL5A2	chr2	ENST00000374866	p.G441V	SVPPGSAGPPGS	SGPPGSAGPPGS
123	COPA	chr1	ENST00000241704	p.A1190T	GTCPEFK	GACPEFK
124	COPB2	chr3	ENST00000333188	p.F630L	EKQGLKQQALTV	EKQGFKQQALTV
125	CPNE3	chr8	ENST00000517490	p.R47H	YEVEHTERIKNC	YEVERTERIKNC
126	CPNE7	chr16	ENST00000268720	p.S153P	FLCCTEPSHLAR	FLCCTESSHLAR
127	CR1	chr1	ENST00000400960	p.A1073T	TITNGDFISTNR	TIANGDFISTNR
128	CRACR2B	chr11	ENST00000525077	p.Q299R	AREQIRRLESEA	AQEQIRRLESEA
129	CRELD1	chr3	ENST00000326434	p.R204C	YFEAECNASHLV	YFEAERNASHLV
130	CRY1	chr12	ENST00000008527	p.R348C	LCQEGWIHHLAR	LRQEGWIHHLAR
131	CRYBG1	chr6	ENST00000369066	p.L214S	GSKPRNHFGVGR	GLKPRNHFGVGR
132	CSAD	chr12	ENST00000267085	p.I181T	STSNMYAVNLAR	SISNMYAVNLAR
133	CSMD1	chr8	ENST00000335551	p.G86W	EYWNNMNCVWL	EYGNNMNCVWLI
134	CSMD2	chr1	ENST00000373381	p.G33R	ALVPGARSRWGR	ALVPGAGSRWGR
135	CSMD3	chr8	ENST00000297405	p.R1574Q	ERITCIQVENQY	ERITCIQVENRY
136	CSPP1	chr8	ENST00000262210	p.L1013V	HTLEIQQQAVLR	HTLEIQQQALLR
137	CTAGE9	chr6	ENST00000314099	p.I174M	LMADISKSMQSL	LMADISKSIQSL
138	CTNNA2	chr2	ENST00000402739	p.M858I	SDSSILDSATSL	SDSSMLDSATSL
139	CTR9	chr11	ENST00000361367	p.D1120G	RSHSGVSENGSR	RSHSGVSENDSR
140	CYP1A2	chr15	ENST00000343932	p.V487M	EFSVPPGMKVDL	EFSVPPGVKVDL
141	CYP3A7	chr7	ENST00000336374	p.S180N	LKHVFGAYNMDV	LKHVFGAYSMDV
142	CYYR1	chr21	ENST00000299340	p.V8M	MDAPRLPMRPGV	MDAPRLPVRPGV
143	DACT2	chr6	ENST00000607983	p.R212H	QSTVEISARAHL	QSTVEISARARL
144	DAGLA	chr11	ENST00000257215	p.H422Y	GYHGTWLGHKGM	GHHGTWLGHKGM
145	DAGLB	chr7	ENST00000297056	p.L630F	AEFSKIFIGPKM	AEFSKILIGPKM
146	DCAF8L1	chrX	ENST00000441525	p.D315E	IELRQDRPASKV	IDLRQDRPASKV
147	DCHS1	chr11	ENST00000299441	p.R866Q	LEVQAGSGV	LEVRAGSGV
148	DDB1	chr11	ENST00000301764	p.N1049S	LSESWYSLLLDM	LSESWYNLLLDM
149	DDI1	chr11	ENST00000302259	p.R118C	RPQHPGQQQCT	RPQHPGQQQRT
150	DDX1	chr2	ENST00000233084	p.L418P	SFDVKKPSEKIM	SFDVKKLSEKIM
151	DDX3X	chrX	ENST00000399959	p.R375H	VHHTMMFSATF	VRHTMMFSATF
152	DENND5A	chr11	ENST00000328194	p.L1237P	WIAPLADCPITA	WIALLADCPITA
153	DENND5B	chr12	ENST00000354285	p.M223V	FVQACKKFLIQL	FMQACKKFLIQL
154	DHRS7	chr14	ENST00000536410	p.S278P	FKSGVDADPSYF	FKSGVDADSSYF
155	DLD	chr7	ENST00000205402	p.D37N	YANQPIDADVTV	YADQPIDADVTV
156	DMRT3	chr9	ENST00000190165	p.N2S	MSGYGSPYL	MNGYGSPYL
157	DNAH1	chr3	ENST00000420323	p.I803V	IVGPFYINTDNV	IIGPFYINTDNV

Number	HUGO Symbol	Chromosome	Transcript ID	HGVSp	MT Epitope Seq	WT Epitope Seq
158	DNAJA1	chr9	ENST00000330899	p.H34R	YRPDKNPNE	YHPDKNPNE
159	DNASE1	chr16	ENST00000246949	p.R148G	RFFSRFTEVGEF	RFFSRFTEVREF
160	DNM1	chr9	ENST00000341179	p.A623V	SWKASFLRVGVY	SWKASFLRAGVY
161	DNMT3B	chr20	ENST00000201963	p.R160Q	TRSLRQRATASA	TRSLRRRATASA
162	DOC2A	chr16	ENST00000350119	p.G45S	YFPRGPGPEGSG	YFPRGPGPEGGG
163	DOCK1	chr10	ENST00000280333	p.L975P	FKNPIGKNVYPF	FKNLIGKNVYPF
164	DOCK2	chr5	ENST00000524185	p.G57D	GYLIKHKMLQDI	GYLIKHKMLQGI
165	DOCK7	chr1	ENST00000454575	p.N842S	GNHDQHGRSSLL	GNHDQHGRNSLL
166	DPH5	chr1	ENST00000342173	p.E264G	IHPMEMGMLSLF	IHPMEMEMLSLF
167	DSG3	chr18	ENST00000257189	p.T220N	LSRNTGEVRNLT	LSRNTGEVRTLT
168	DUSP10	chr1	ENST00000366899	p.Y363C	YHCEKGLFNYKR	YHYEKGLFNYKR
169	DUSP7	chr3	ENST00000495880	p.K160N	ASVLGLLLQNLR	ASVLGLLLQKLR
170	DYNLL1	chr12	ENST00000242577	p.T26A	AAQALEKYNIEK	ATQALEKYNIEK
171	ECEL1	chr2	ENST00000304546	p.A45T	RSTTGARSGLPR	RSATGARSGLPR
172	ECHDC3	chr10	ENST00000379215	p.A153T	NGLAAATGCQLV	NGLAAAAGCQLV
173	EEF1AKMT1	chr13	ENST00000382754	p.Q57R	FWYSRETALQLA	FWYSQETALQLA
174	EEF2KMT	chr16	ENST00000427587	p.L11H	ENAGTEHLLQSF	ENAGTELLLQSF
175	EFNB2	chr13	ENST00000646441	p.R294G	DIIIPLGTADSV	DIIIPLRTADSV
176	EFTUD2	chr17	ENST00000426333	p.A382T	LYKILTQVVGDV	LYKILAQVVGDV
177	EQ3	chr14	ENST00000250457	p.N33S	FCYLDSFLGEVV	FCYLDNFLGEVV
178	EHD2	chr19	ENST00000263277	p.L512P	FASHPI	FASHLI
179	EIF3L	chr22	ENST00000412331	p.T511A	RSFLKLYATMPV	RSFLKLYTTMPV
180	EIF4G3	chr1	ENST00000400422	p.A1510S	KASIIADSSTFR	KAAIIADSSTFR
181	EIF5A	chr17	ENST00000336452	p.Q125R	NDFQLIGIRDGY	NDFQLIGIQDGY
182	ELMO2	chr20	ENST00000290246	p.L289F	LYVFQVLTF	LYVLQVLTF
183	ELP5	chr17	ENST00000396627	p.H143Y	LPCTTLCQVLYA	LPCTTLCQVLHA
184	EMC1	chr1	ENST00000375199	p.P621H	RHILQSLLLPVM	RPILQSLLLPVM
185	ENTPD6	chr20	ENST00000354989	p.R400W	IAAKYVCWTLET	IAAKYVCRTLET
186	EPB41L2	chr6	ENST00000337057	p.V586M	AMVQDGDGRREV	AVVQDGDGRREV
187	EPC1	chr10	ENST00000263062	p.I74M	KRDNMVMPVPEA	KRDNMVIPVPEA
188	EPG5	chr18	ENST00000282041	p.N2096D	FLGSVLCEVDWV	FLGSVLCEVNWV
189	EPHA4	chr2	ENST00000281821	p.G675R	MRQFDHPNIIHL	MGQFDHPNIIHL
190	EPHB2	chr1	ENST00000374630	p.N755S	VSSNLVCKVSDF	VNSNLVCKVSDF
191	EPPK1	chr8	ENST00000568225	p.D1629G	AERAVTGYTGPY	AERAVTGYTDPY
192	EPS8	chr12	ENST00000281172	p.V398I	LNYTINGDERQL	LNYTVNGDERQL
193	ERCC1	chr19	ENST00000013807	p.K213R	YRAYEQKPADLL	YKAYEQKPADLL
194	ERCC3	chr2	ENST00000285398	p.N499D	MELQNDGYIAKV	MELQNNGYIAKV
195	ERCC6	chr10	ENST00000374127	p.V447I	LKKKIQIQQPNM	LKKKIQVQQPNM
196	ERG28	chr14	ENST00000256319	p.T23A	IIAMGNALQSFR	IIAMGNTLQSFR
197	EXOC3	chr5	ENST00000512944	p.R608W	LWAVMQKRISFR	LRAVMQKRISFR
198	EXOC8	chr1	ENST00000366645	p.A295T	SEKRRREQEETA	SEKRRREQEEAA
199	EYA1	chr8	ENST00000340726	p.A411G	TYNFGTDGFPGA	TYNFGTDGFPAA
200	EYA4	chr6	ENST00000355286	p.A292V	QYYSASTYGVYM	QYYSASTYGAYM
201	F11R	chr1	ENST00000368026	p.L11M	TKAQVERKMLCL	TKAQVERKLLCL
202	FAM13B	chr5	ENST00000033079	p.A460T	VQGETACVSI	VQGEAACVSI
203	FAM160A2	chr11	ENST00000265978	p.P805H	VFQHSVKSLLQV	VFQPSVKSLLQV
204	FAM160B1	chr10	ENST00000369248	p.K297R	AAARCLTQSTCL	AAAKCLTQSTCL
205	FAM161B	chr14	ENST00000286544	p.V162I	RSISSWASSITV	RSVSSWASSITV
206	FAM25G	chr10	ENST00000452267	p.T42I	AKEIGEKAIAEA	AKETGEKAIAEA
207	FAM3A	chrX	ENST00000447601	p.L169P	KLFSEPGSRNAK	KLFSELGSRNAK
208	FANCE	chr6	ENST00000229769	p.L239P	ERPEHKSPE	ERPEHKSLE
209	FARP1	chr13	ENST00000319562	p.P10T	MGEIEQRPTTGS	MGEIEQRPTPGS
210	FBN1	chr15	ENST00000316623	p.D2801G	RYLIESGNEGGF	RYLIESGNEDGF

Number	HUGO Symbol	Chromosome	Transcript ID	HGVSp	MT Epitope Seq	WT Epitope Seq
211	FBN1	chr15	ENST00000316623	p.Y1219C	TFCTNSEGSCEC	TFCTNSEGSYEC
212	FBN3	chr19	ENST00000270509	p.R48W	RRWGSPGIL	RRRGSPGIL
213	FBXO11	chr2	ENST00000403359	p.Q56R	PPPPQQQQQRP	PPPPQQQQQQP
214	FCER2	chr19	ENST00000346664	p.Y189C	QWVHARCACDDM	QWVHARYACDDM
215	FCHO1	chr19	ENST00000597512	p.R672H	TFPAGIVHVF	TFPAGIVRVF
216	FCRL4	chr1	ENST00000271532	p.N180T	YGDENDVFRSTF	YGDENDVFRSNF
217	FGD3	chr9	ENST00000337352	p.R552M	KRMHHCKLCGAV	KRRHHCKLCGAV
218	FGF6	chr12	ENST00000228837	p.G77D	IAGVNWESDYLV	IAGVNWESGYLV
219	FGF6	chr12	ENST00000228837	p.R105Q	FHLQVLPDGQIS	FHLQVLPDGRIS
220	FGF7	chr15	ENST00000267843	p.T179M	RGKKMKKEQKTA	RGKKTKKEQKTA
221	FLII	chr17	ENST00000327031	p.D1193G	FCQDDLADGDIM	FCQDDLADDDIM
222	FLNB	chr3	ENST00000295956	p.Q2296R	TVMSLRESGLKV	TVMSLQESGLKV
223	FMNL3	chr12	ENST00000335154	p.Q1003R	HCRPMVVRHQAR	HCQPMVVRHQAR
224	FMOD	chr1	ENST00000354955	p.A267V	YFRGVPKLLYVR	YFRGAPKLLYVR
225	FOXA1	chr14	ENST00000250448	p.H382P	AHGLAPPES	AHGLAFS
226	FOXD2	chr1	ENST00000334793	p.A139D	YSYIALITMDIL	YSYIALITMAIL
227	FOXP4	chr6	ENST00000307972	p.A642V	HQVQVKEEPVEA	HQVQVKEEPAEA
228	FOXRED2	chr22	ENST00000216187	p.Y391C	ILGTASHSVDCR	ILGTASHSVDYR
229	FRMD1	chr6	ENST00000283309	p.D193G	ACALQAGLGEHR	ACALQADLGEHR
230	FRMPD1	chr9	ENST00000377765	p.S1284N	SEGKSDNSSICL	SEGKSDSSSICL
231	FSD2	chr15	ENST00000334574	p.C608R	SDTHFTRRVAVM	SDTHFTRCVAVM
232	FZD2	chr17	ENST00000315323	p.R89C	KVQCSPELCFFL	KVQCSPELRFFL
233	GAA	chr17	ENST00000302262	p.I823T	LRAGYTIPL	LRAGYIIPL
234	GAB1	chr4	ENST00000262994	p.Q643H	RKHKSSGSGSSV	RKQKSSGSGSSV
235	GABPA	chr21	ENST00000354828	p.I210V	EFSMTDVDLTTL	EFSMTDIDLTTL
236	GAL3ST4	chr7	ENST00000360039	p.A292S	LSWLDSVFDLVM	LAWLDSVFDLVM
237	GAREM1	chr18	ENST00000269209	p.G758V	SVSPDLSEDQYF	SGSPDLSEDQYF
238	GATA5	chr20	ENST00000252997	p.S306P	IAKRSSGPTR	IAKRSSGSTR
239	GCNT1	chr9	ENST00000376730	p.A170T	MGITSCFSNVFV	MGIASCFSNVFV
240	GET4	chr7	ENST00000265857	p.S134G	SGGGSGKLGHPR	SSGGSGKLGHPR
241	GJA4	chr1	ENST00000342280	p.P59S	ECNTAQSGCTNV	ECNTAQPGCTNV
242	GLI2	chr2	ENST00000361492	p.R914W	MSLWTRLALLDA	MSLRTRLALLDA
243	GLMP	chr1	ENST00000362007	p.G242S	LEVATLSQGPDC	LEVATLGQGPDC
244	GNAI2	chr3	ENST00000313601	p.P289H	ICFHEYTGANKY	ICFPEYTGANKY
245	GPC6	chr13	ENST00000377047	p.P305S	ERLEGSFNIESV	ERLEGPFNIESV
246	GPR34	chrX	ENST00000378138	p.R266H	YATTAHNSFIVL	YATTARNSFIVL
247	GRAMD1B	chr11	ENST00000529750	p.V498A	RNKSRLRASTEL	RNKSRLRVSTEL
248	GREM2	chr1	ENST00000318160	p.R90C	VSEEGCRSCTIL	VSEEGCRSRTIL
249	GRM5	chr11	ENST00000305432	p.A1120V	VAKPDLEEL	AAKPDLEEL
250	GRTP1	chr13	ENST00000326039	p.M141T	ALTERLGVLWTL	ALMERLGVLWTL
251	GSTK1	chr7	ENST00000358406	p.L43I	RPSIITGIMKDS	RPSLITGIMKDS
252	GTF3C1	chr16	ENST00000356183	p.T200A	ELQRDLHTAAFK	ELQRDLHTTAFK
253	GTF3C6	chr6	ENST00000329970	p.M105T	YKCHTMKKLSTT	YKCHTMKKLSMT
254	GTPBP2	chr6	ENST00000307126	p.V326A	KIDLCAKTTAER	KIDLCAKTTVER
255	H1FNT	chr12	ENST00000335017	p.A176V	RRNVRAKAKANA	RRNARAKAKANA
256	HCFC1	chrX	ENST00000310441	p.A812V	KVMTSGTGAPVK	KVMTSGTGAPAK
257	HCN2	chr19	ENST00000251287	p.A322V	YKTARVLRIVRF	YKTARALRIVRF
258	HDAC9	chr7	ENST00000401921	p.Y846H	PMGDVEHLEAFR	PMGDVEYLEAFR
259	HECTD2	chr10	ENST00000298068	p.A722T	SDRVPVGGMTDL	SDRVPVGGMADL
260	HELZ2	chr20	ENST00000467148	p.Y552H	FGTGKTHTL	FGTGKTYTL
261	HERC1	chr15	ENST00000443617	p.A171V	LLFVLLRQSWMM	LLFALLRQSWMM
262	HIP1R	chr12	ENST00000253083	p.R1000W	LEKTLEAERMWL	LEKTLEAERMRL
263	HIVEP2	chr6	ENST00000367603	p.T2340M	AIASLRIAMEEA	AIASLRIATEEA

Number	HUGO Symbol	Chromosome	Transcript ID	HGVSp	MT Epitope Seq	WT Epitope Seq
264	HMHB1	chr5	ENST00000289448	p.K19T	KRGSLHVWTSEL	KRGSLHVWKSEL
265	HOXA1	chr7	ENST00000643460	p.Y123C	QEADVSGGCPQC	QEADVSGGYPQC
266	HOXA9	chr7	ENST00000343483	p.Y11C	YCVDSFLLGADA	YYVDSFLLGADA
267	HRCT1	chr9	ENST00000354323	p.H89L	PNVGLHLHHHPR	PNVGLHHHHHPR
268	HRH3	chr20	ENST00000317393	p.N75S	NFFLLSLAISDF	NFFLLNLAISDF
269	HSPG2	chr1	ENST00000374695	p.E2346K	QPIRIKPSSSQV	QPIRIEPSSSQV
270	HUWE1	chrX	ENST00000262854	p.E4064G	LRGWYMIISREM	LREWYMIISREM
271	IDH2	chr15	ENST00000330062	p.V209M	EMYNFPAGGVGM	EVYNFPAGGVGM
272	IFRD2	chr3	ENST00000417626	p.R179C	FLLECRLTLADA	FLLERRLTLADA
273	IFT140	chr16	ENST00000361339	p.K64R	YRKCRRHDL	YRKCKRHDL
274	IGLC2	chr22	ENST00000390323	p.N22S	SEELQASKATLV	SEELQANKATLV
275	IGSF11	chr3	ENST00000354673	p.L207M	IRNISAMSSG	IRNISALSSG
276	IKZF1	chr7	ENST00000331340	p.A181V	CNYVCRRRDALT	CNYACRRRDALT
277	IL17RD	chr3	ENST00000296318	p.N55S	KITFKYDNCT	LYNITFKYDNCT
278	IL36RN	chr2	ENST00000346807	p.R102W	AKESKSFTFYWR	AKESKSFTFYRR
279	IMMT	chr2	ENST00000409258	p.V10M	LRACQLSGMTAA	LRACQLSGVTAA
280	ING3	chr7	ENST00000315870	p.L126S	ERRSLESDTPSQ	ERRSLELDTPSQ
281	INPP5K	chr17	ENST00000421807	p.S353P	DMMVSYSSTPDF	DMMVSYSSTSDF
282	IQCG	chr3	ENST00000265239	p.I37M	MPKETDIEI	IPKETDIEI
283	IQSEC1	chr3	ENST00000618604	p.S808L	RSALSSSLRDLL	RSALSSSLRDLS
284	IQSEC1	chr3	ENST00000273221	p.S930L	ALSSSLRDLLEA	ALSSSLRDLSEA
285	IRF1	chr5	ENST00000245414	p.G45D	AAKHDWDINKDA	AAKHGWDINKDA
286	IRX2	chr5	ENST00000302057	p.N139S	HRKSPYPTK	HRKNPYPTK
287	ITGA9	chr3	ENST00000264741	p.Y513H	EIQHVLMADV	EIQYVLMADV
288	ITGAV	chr2	ENST00000261023	p.S59G	FFVPGASSRMFL	FFVPSASSRMFL
289	ITGB1BP2	chrX	ENST00000373829	p.V288A	FLMPSRAEISLV	FLMPSRVEISLV
290	ITGB6	chr2	ENST00000283249	p.G561A	NGDCDCAECVCR	NGDCDCGECVCR
291	ITM2C	chr2	ENST00000326407	p.R95C	ARDNFFCCGVLY	ARDNFFRCGVLY
292	JPH2	chr20	ENST00000342272	p.P55H	VYTWHSGNTF	VYTWPSGNTF
293	KATNBL1	chr15	ENST00000256544	p.I211V	QYVSLGCCVDLL	QYISLGCCVDLL
294	KCNAB1	chr3	ENST00000471742	p.T76S	ETTRAESGMAYR	ETTRAETGMAYR
295	KCNC3	chr19	ENST00000477616	p.M685V	IDQPAVSPEDKS	IDQPAMSPEDKS
296	KCTD6	chr3	ENST00000355076	p.A49V	FPTVRDPQGNYF	FPTARDPQGNYF
297	KDM4C	chr9	ENST00000381309	p.I692F	MCFFYSEENIEY	MCFIYSEENIEY
298	KDM6B	chr17	ENST00000254846	p.T321A	AAHPPGHRL	TAHPPGHRL
299	KIAA1109	chr4	ENST00000306802	p.W1303R	FMCNTRHLEPTL	FMCNTWHLEPTL
300	KIAA1324	chr1	ENST00000529753	p.S146R	LSANMELDDRAA	LSANMELDDSAA
301	KIAA1324	chr1	ENST00000529753	p.P746L	TVPGSLLLLGTC	TVPGSLLLPGTC
302	KIAA1671	chr22	ENST00000358431	p.R1169C	TLRSCPKDLPVR	TLRSRPKDLPVR
303	KIF26A	chr14	ENST00000315264	p.A1469T	ERPPTGPTL	ERPPTGPAL
304	KIFC2	chr8	ENST00000301332	p.S612T	VTLTLRAATPPR	VTLTLRADPR
305	KIT	chr4	ENST00000288135	p.V532I	VIIAGMMCIIVM	VIVAGMMCIIVM
306	KLF9	chr9	ENST00000377126	p.C82Y	KYRPIQTPSVYS	KYRPIQTPSVCS
307	KLHDC2	chr14	ENST00000298307	p.H221R	ARACATVGNRGF	AHACATVGNRGF
308	KLHL18	chr3	ENST00000232766	p.L193P	SRDEPNVKS	SRDELNVKS
309	KLHL4	chrX	ENST00000373114	p.A430T	PRKSTVGTL	PRKSTVGAL
310	KMT2A	chr11	ENST00000389506	p.R158K	FLGFGSDEEVKV	FLGFGSDEEVRV
311	KNG1	chr3	ENST00000644859	p.G504R	HRKHKNKGK	HGKHKNKGK
312	KRI1	chr19	ENST00000312962	K331_R332delinsN	ENWEETRERKKR	EKREETRERKKR
313	KRT24	chr17	ENST00000264651	p.A319V	RVQYEELAEQNR	RAQYEELAEQNR
314	KRT35	chr17	ENST00000246639	p.R408H	GPSAAHTNCSPR	GPSAARTNCSPR
315	KRTAP26-1	chr21	ENST00000360542	p.H177R	YRPQSLRVVSSS	YRPQSLHVVSSS
316	KSR1	chr17	ENST00000644974	p.L47R	QKRIDISIGSLR	QKLIDISIGSLR

Number	HUGO Symbol	Chromosome	Transcript ID	HGVSp	MT Epitope Seq	WT Epitope Seq
317	L3HYPDH	chr14	ENST00000247194	p.H72R	LMFEPRGRRDMY	LMFEPRGHRDMY
318	LAMA4	chr6	ENST00000230538	p.E503K	VRDAKDMNRATA	VRDAEDMNRATA
319	LAMB1	chr7	ENST00000222399	p.E1202G	NRTFLGKAKA	NRTFLEKAKA
320	LATS1	chr6	ENST00000253339	p.T487A	ANSQPSATTVAA	ANSQPSATTVTA
321	LHCGR	chr2	ENST00000294954	p.S420P	ASVDPQTKGQYY	ASVDSQTKGQYY
322	LINGO1	chr15	ENST00000355300	p.R473W	LVSAKSNGWLTV	LVSAKSNGRLTV
323	LMNTD1	chr12	ENST00000413632	p.S44P	VYSLVHFPPKML	VYSLVHFSPKML
324	LMTK3	chr19	ENST00000270238	p.P1195L	RRLEPAPLR	RRLEPAPPR
325	LRP1B	chr2	ENST00000389484	p.C1042R	FSDEAQINRTKE	FSDEAQINCTKE
326	LRR1	chr14	ENST00000298288	p.R12W	VISWHLPALGLR	VISRHLPALGLR
327	LRRC37A3	chr17	ENST00000319651	p.S90P	FDHLGPPASSEM	FDHLGPSASSEM
328	LRRC7	chr1	ENST00000370958	p.K153E	IKCCECLTIIEA	IKCCKCLTIIEA
329	LRRC9	chr14	ENST00000647410	p.R806G	LGLSVIGRLKTL	LRLSVIGRLKTL
330	LRRFIP2	chr3	ENST00000336686	p.V671A	EKAEDELKAEKR	EKVEDELKAEKR
331	LRSAM1	chr9	ENST00000300417	p.P214S	YYSPSQYLL	YYPPSQYLL
332	LSR	chr19	ENST00000347609	p.I158F	YYQGRRFTI	YYQGRRITI
333	LTC4S	chr5	ENST00000292596	p.L14P	AAVTLPGVLLQA	AAVTLLGVLLQA
334	LUC7L3	chr17	ENST00000393227	p.R312Q	SRSQDHKRSRSR	SRSRDHKRSRSR
335	LY75	chr2	ENST00000263636	p.T1473A	FEWSDGSAF	FEWSDGSTF
336	LYG2	chr2	ENST00000409238	p.R64H	IHGSEMFAEMDL	IRGSEMFAEMDL
337	LZTS2	chr10	ENST00000370220	p.S570I	GILRAQVERLRV	GSLRAQVERLRV
338	MACC1	chr7	ENST00000332878	p.V609A	KIGLAHCKNVKV	KIGLVHCKNVKV
339	MACF1	chr1	ENST00000671089	p.R849W	LEDNSQWTKWKV	LEDNSQRTKWKV
340	MAFB	chr20	ENST00000373313	p.S56P	TRLQPAGSVPST	TRLQPAGSVSST
341	MAGED1	chrX	ENST00000326587	p.P353A	IWQNAVIWPNPI	IWQNPVIWPNPI
342	MAN2A2	chr15	ENST00000360468	p.V418I	KSQLFRSNILLV	KSQLFRSNVLLV
343	MAP6	chr11	ENST00000304771	p.G475C	GLLKGQCPM	GLLKGQGPM
344	MAPK6	chr15	ENST00000261845	p.E407G	PRKYLDGDRGKY	PRKYLDGDREKY
345	MAST1	chr19	ENST00000251472	p.P369L	SKAKKPLGENDF	SKAKKPPGENDF
346	MC4R	chr18	ENST00000299766	p.I289M	FNLYLMLIMCNS	FNLYLILIMCNS
347	MCAT	chr22	ENST00000290429	p.T282M	MQALKAVDI	TQALKAVDI
348	MCM3AP	chr21	ENST00000291688	p.F139C	VNSGCGKTEFSF	VNSGFGKTEFSF
349	MCM7	chr7	ENST00000491245	p.L33S	ICENARPSPSPR	ICENARPSPLPR
350	MDH1B	chr2	ENST00000374412	p.M99T	YYDVTSSTTTEL	YYDVTSSMTTEL
351	MECOM	chr3	ENST00000264674	p.A899V	GVILDDKEDAYF	GAILDDKEDAYF
352	MTL23	chr17	ENST00000341249	p.Q12K	VVLAKYLWFHRR	VVLAQYLWFHRR
353	MFAP1	chr15	ENST00000267812	p.N24S	RSEKGEISMEKV	RNEKGEISMEKV
354	MFSD1	chr3	ENST00000264266	p.Y417H	LDSRGHLFLEVF	LDSRGYLFLEVF
355	MGAM	chr7	ENST00000549489	p.G466V	SSSSKPYVPYDR	SSSSKPYGPYDR
356	MGAT3	chr22	ENST00000341184	p.R514H	TAAGGWHHR	TAAGGWRHR
357	MGAT5B	chr17	ENST00000428789	p.D114N	RAGGNLHFPADR	RAGGDLHFPADR
358	MGAT5B	chr17	ENST00000428789	p.R463Q	KQLIKGGKNM	KRLIKGGKNM
359	MICALL2	chr7	ENST00000297508	p.V384A	RAAAPQTTL	RVAAPQTTL
360	MMP12	chr11	ENST00000571244	p.R402M	QYWMYDERRQMM	QYWRYDERRQMM
361	MRPS31	chr13	ENST00000323563	p.M106T	KDLLGIIKGTKV	KDLLGIIKGMKV
362	MSTO1	chr1	ENST00000649846	p.S428G	DRACHTGHRLMV	DRACHTSHRLMV
363	MSTO1	chr1	ENST00000245564	p.S428G	IDRACHTGQLTP	IDRACHTSQLTP
364	MTA3	chr2	ENST00000407270	p.Y13C	VCFENSSSNPYL	VYFENSSSNPYL
365	MTHFD1L	chr6	ENST00000367321	p.S690A	FANIAHGNASVL	FANIAHGNSSVL
366	MTHFD1L	chr6	ENST00000367321	p.D867E	VEKIRTIAQAVY	VDKIRTIAQAVY
367	MUC17	chr7	ENST00000306151	p.F4199V	SVKVTEELK	SVKFTEELK
368	MYH7B	chr20	ENST00000262873	p.I217V	LRNRDNQSMLVT	LRNRDNQSMLIT
369	MYLK	chr3	ENST00000475616	p.W853R	RYGSLRPGRPAR	RYGSLRPGWPAR

Number	HUGO Symbol	Chromosome	Transcript ID	HGVSp	MT Epitope Seq	WT Epitope Seq
370	MYO18A	chr17	ENST00000527372	p.T1698A	FACAAAVKARKA	FTCAAAVKARKA
371	MYO18B	chr22	ENST00000335473	p.L1591M	LTCDLEDTCVML	LTCDLEDTCVLL
372	MYO5A	chr15	ENST00000356338	p.R903H	RMMAKHELKKLK	RMMAKRELKKLK
373	MYO7A	chr11	ENST00000409619	p.K1297N	LYIALFDNVSSL	LYIALFDKVSSL
374	NAIF1	chr9	ENST00000373078	p.R54M	LRMVNAVATCRR	LRRVNAVATCRR
375	NAT10	chr11	ENST00000257829	p.Y171H	SRHRTEAHQDVV	SRYRTEAHQDVV
376	NCOA7	chr6	ENST00000368357	p.E771A	YYEDEDEAVLPV	YYEDEDEEVLPV
377	NDUFA11	chr19	ENST00000308961	p.T74A	FGLTACISAHVR	FGLTTCISAHVR
378	NF1	chr17	ENST00000356175	p.K2534N	ISDTKAPNRQEM	ISDTKAPKRQEM
379	NFAM1	chr22	ENST00000329021	p.L112P	TENQSHTPDCQV	TENQSHTLDCQV
380	NHLH1	chr1	ENST00000302101	p.R99H	FNLAFAELHKLL	FNLAFAELRKLL
381	NINL	chr20	ENST00000278886	p.D371E	LDNELMTVESAV	LDNELMTVDSAV
382	NOD1	chr7	ENST00000222823	p.W820L	SISEVGMLGNQV	SISEVGMWGNQV
383	NR1I2	chr3	ENST00000337940	p.V68A	PGKPSANADEEV	PGKPSVNADEEV
384	NRAS	chr1	ENST00000369535	p.G12C	EYKLVVVGACGV	EYKLVVVGAGGV
385	NRBF2	chr10	ENST00000277746	p.Y40H	HKKAAAHLSEAM	HKKAAAYLSEAM
386	NSMCE4A	chr10	ENST00000369023	p.R300H	FVVDPHSFPHTV	FVVDPHSFPRTV
387	NSUN4	chr1	ENST00000474844	p.L244P	EPEGDTYDRVLV	ELEGDTYDRVLV
388	NT5C1A	chr1	ENST00000235628	p.A344T	VAGAQEMGTVTA	VAGAQEMGTVAA
389	NT5DC1	chr6	ENST00000319550	p.V84A	NFLKLANNGTAL	NFLKLANNGTVL
390	NTN4	chr12	ENST00000343702	p.Y159H	YKHFATNCSATF	YKYFATNCSATF
391	NUAK2	chr1	ENST00000367157	p.R378H	LKKSHKENDMAQ	LKKSRKENDMAQ
392	NUP85	chr17	ENST00000245544	p.Y268H	CERHLQDSTFAT	CERYLQDSTFAT
393	NUTM2F	chr9	ENST00000253262	p.S408Y	MEELLGYHPGDT	MEELLGSHPGDT
394	NXPH3	chr17	ENST00000328741	p.C237F	KVFPDYNYH	KVCPDYNYH
395	OBSCN	chr1	ENST00000636476	p.R2408W	VWPPKWLLGKTV	VRPPKWLLGKTV
396	OR10G3	chr14	ENST00000303532	p.A120V	FLYTLMVYDRYL	FLYTLMAYDRYL
397	OR13D1	chr9	ENST00000318763	p.M33V	LEHVETRNYSAM	LEHMRNYSAM
398	OR4C16	chr11	ENST00000623907	p.A186F	QFCSETYVVNLL	QACSETYVVNLL
399	OR51I2	chr11	ENST00000341449	p.R131H	LHYATVLTTEVI	LRYATVLTTEVI
400	OR5B17	chr11	ENST00000357377	p.M57V	VYFFLSNLSL	MYFFLSNLSL
401	OSGIN2	chr8	ENST00000451899	p.V42G	YFGDNLGRKGKA	YFGDNLGRKVKA
402	P2RX1	chr17	ENST00000225538	p.R25H	VHNKKVGVIFRL	VRNKKVGVIFRL
403	PAMR1	chr11	ENST00000619888	p.V607G	WNVLADGRSPGF	WNVLADVRSPGF
404	PARP2	chr14	ENST00000250416	p.T398A	THSDYAMTLLDL	THSDYTMTLLDL
405	PCDHA5	chr5	ENST00000529619	p.A99V	RRRVECSIHLEV	RRRAECSIHLEV
406	PCDHA7	chr5	ENST00000356878	p.P183S	FFLDVSTSNQQV	FFLDVPTSNQQV
407	PCDHB1	chr5	ENST00000306549	p.E439K	AETMIKVLISDV	AETMIEVLISDV
408	PCDHGA2	chr5	ENST00000394576	p.A331V	TRVKVIVTVLDV	TRAKVIVTVLDV
409	PDS5A	chr4	ENST00000303538	p.P103S	FRIYASEAPYTS	FRIYAPEAPYTS
410	PER2	chr2	ENST00000254657	p.S99N	CSNDQSSKVDTH	CSSDQSSKVDTH
411	PEX5	chr12	ENST00000266563	p.L381P	VSFTNESPQRQA	VSFTNESLQRQA
412	PGAP1	chr2	ENST00000354764	p.Y158H	LKLHKGQEFAPK	LKLYKGQEFAPK
413	PGBD4	chr15	ENST00000397766	p.I462T	KFFHHLLHTTVL	KFFHHLLHITVL
414	PGM3	chr6	ENST00000512866	p.E315G	LVGIGESLNIGV	LVEIGESLNIGV
415	PHB	chr17	ENST00000300408	p.R157Q	SRQVSDDLTEQA	SRQVSDDLTERA
416	PHF7	chr3	ENST00000327906	p.R362G	ESSRGRGSYSWR	ESSRGRRSYSWR
417	PIGT	chr20	ENST00000279036	p.Y456H	EHTPDPNHGFYV	EYTPDPNHGFYV
418	PIK3C2B	chr1	ENST00000367187	p.S480N	NDDQNPSTLNYL	NDDQSPSTLNYL
419	PIP5K1C	chr19	ENST00000335312	p.A494G	YDLRGGRSYPTL	YDLRGARSYPTL
420	PITPNM2	chr12	ENST00000542749	p.R1088G	KMVVGGDHTF	KMVVRGDHTF
421	PITPNM2	chr12	ENST00000542749	p.R650C	SRSNVDIPC	SRSNVDIPR
422	PJA2	chr5	ENST00000361189	p.I160V	QNGVVHTDSY	QNGIALVHTDSY

Number	HUGO Symbol	Chromosome	Transcript ID	HGVSp	MT Epitope Seq	WT Epitope Seq
423	PKD1L2	chr16	ENST00000525539	p.W1855R	GRASSLRRLISM	GRASSLRWLISM
424	PKN1	chr19	ENST00000242783	p.V328A	ARVVGCRDL	VRVVGCRDL
425	PKP4	chr2	ENST00000389757	p.R521G	SIQKDPGEFAWR	SIQKDPREFAWR
426	PLA2G4C	chr19	ENST00000599921	p.M98T	YTNDGDTEALEA	YTNDGDMEALEA
427	PLCB3	chr11	ENST00000279230	p.L310P	ILPPEALDL	ILPLEALDL
428	PLCH1	chr3	ENST00000334686	p.P1056H	HVVNPTQDL	PVVNPTQDL
429	PLEKHG3	chr14	ENST00000247226	p.S700P	RPSCKKKESALS	RSSCKKKESALS
430	PLOD3	chr7	ENST00000223127	p.R181C	TIHQIVCQWKYK	TIHQIVRQWKYK
431	PLPP3	chr1	ENST00000371250	p.R107Q	IITGEFYQIYYL	IITGEFYRIYYL
432	PLXNC1	chr12	ENST00000258526	p.R1417C	SLPLCFWVNILK	SLPLRFWVNILK
433	PLXNC1	chr12	ENST00000549217	p.S403L	LSTKPSSLLLGK	LSTKPSSSLLGK
434	PMF1	chr1	ENST00000368277	p.Y140C	APCFLQQRDTLR	APYFLQQRDTLR
435	PNPT1	chr2	ENST00000447944	p.R26W	PRWDRALTQLQV	PRRDRALTQLQV
436	POLD2	chr7	ENST00000452185	p.R255G	HSTQSGDSI	HSTQSRDSI
437	POLE	chr12	ENST00000320574	p.Y539C	DDGHVLDSETCV	DDGHVLDSETYV
438	POLQ	chr3	ENST00000264233	p.C1475W	GVEGEWLPVPET	GVEGECLPVPET
439	POLR2A	chr17	ENST00000617998	p.R967G	MREDREVLGVIF	MREDREVLRVIF
440	POU5F2	chr5	ENST00000606183	p.R166H	QTTICHFEAQQL	QTTICRFEAQQL
441	PPRC1B	chr5	ENST00000309241	p.L775P	ETAPEEEDL	ETALEEEDL
442	PPFIA1	chr11	ENST00000253925	p.S458P	TVDKLLSEPNER	TVDKLLSESNER
443	PPIH	chr1	ENST00000304979	p.V140L	VLFGKIIDGLLV	VVFGKIIDGLLV
444	PPM1M	chr3	ENST00000323588	p.R32G	SPVPYGRPRFLR	SPVPYRRPRFLR
445	PPP1R27	chr17	ENST00000330261	p.R16Q	ARYSPRQQRRRM	ARYSPRQRRRRM
446	PPP4R1	chr18	ENST00000400555	p.H211R	EMCCDCRMFRVR	EMCCDCRMFHVR
447	PPP6R1	chr19	ENST00000412770	p.D381V	LALVVPNTMLDL	LALDVPNTMLDL
448	PRDM16	chr1	ENST00000270722	p.V17I	LAKSDGDIVNNM	LAKSDGDVVNNM
449	PRDX2	chr19	ENST00000301522	p.E27D	KDVKLSDYKGKY	KEVKLSDYKGKY
450	PRDX3	chr10	ENST00000298510	p.G80R	NREFKDLSLDDF	NGEFKDLSLDDF
451	PRKCZ	chr1	ENST00000378567	p.W296G	ELVHDDEDIDGV	ELVHDDEDIDWV
452	PRKDC	chr8	ENST00000314191	p.Y3413C	PAAGVIDACMTL	PAAGVIDAYMTL
453	PRLHR	chr10	ENST00000239032	p.V316M	PYAFGLMQLL	PYAFGLVQLL
454	PRMT6	chr1	ENST00000370078	p.T142S	RVHVLPGPVESV	RVHVLPGPVETV
455	PRMT7	chr16	ENST00000441236	p.R363C	ERVRQMCPVCDC	ERVRQMRPVCDC
456	PRMT9	chr4	ENST00000322396	p.V308A	AAECAEIRRHHR	AVECAEIRRHHR
457	PCR	chr20	ENST00000216968	p.Y44C	FRDPYHVWC	FRDPYHVWY
458	PS3	chr19	ENST00000396908	p.T167I	TAVPTAVNVISA	TAVPTAVNVTSA
459	PRPF19	chr11	ENST00000227524	p.S503G	DRSLKFYGL	DRSLKFYSL
460	PRRC2C	chr1	ENST00000426496	p.D162G	TNDGNYGPGPSL	TNDDNYGPGPSL
461	PRRT4	chr7	ENST00000489835	p.P129L	RASSLLLESTSR	RASSLLPESTSR
462	PSD	chr10	ENST00000020673	p.G198A	SLPNGLGAPPER	SLPNGLGGPPER
463	PTCD2	chr5	ENST00000308077	p.K131E	FTLGEYEFGPLF	FTLGEYKFGPLF
464	PTCD3	chr2	ENST00000254630	p.N455D	FIGPDQHRDFYY	FIGPDQHRNFYY
465	PTGER3	chr1	ENST00000306666	p.V201I	QYTIQWPGTWCF	QYTVQWPGTWCF
466	PTPN3	chr9	ENST00000412145	p.P706S	VRSLRVDSESVL	VRSLRVDSEPVL
467	PTPRG	chr3	ENST00000295874	p.M1392V	STKENGNGPVTV	STKENGNGPMTV
468	PTPRJ	chr11	ENST00000418331	p.W474R	ARSSHDAESFQM	AWSSHDAESFQM
469	PWWP2A	chr5	ENST00000307063	p.S584C	ASVCCIDSTDDL	ASVCSIDSTDDL
470	PXDN	chr2	ENST00000252804	p.D828G	GQFLDHGLDSTV	GQFLDHDLDSTV
471	PXK	chr3	ENST00000383716	p.F515L	STSVEHAPL	STSVEHAPF
472	RAB38	chr11	ENST00000243662	p.L61P	RPQLWDIAGQER	RLQLWDIAGQER
473	RAB3GAP2	chr1	ENST00000358951	p.P470T	RVAQFLVIYATR	RVAQFLVIYAPR
474	RAB3GAP2	chr1	ENST00000358951	p.A1130V	PVLDTEDVWLSV	PVLDTEDAWLSV
475	RASGRP2	chr11	ENST00000354024	p.F435L	IEKMVESVLRNF	IEKMVESVFRNF

Number	HUGO Symbol	Chromosome	Transcript ID	HGVSp	MT Epitope Seq	WT Epitope Seq
476	RBL1	chr20	ENST00000344359	p.T719A	VAIPLHGVANDA	VTIPLHGVANDA
477	RD3	chr1	ENST00000367002	p.I159T	RARTSPFASDIR	RARISPFASDIR
478	RECQL4	chr8	ENST00000617875	p.A889V	LSHQVAPGPRRV	LSHQAAPGPRRV
479	RECQL5	chr17	ENST00000423245	p.I47T	ATMAVVKGITTV	ATMAVVKGITIV
480	RECQL5	chr17	ENST00000317905	p.I74T	LPALLAKGITTV	LPALLAKGITIV
481	REST	chr4	ENST00000309042	p.P479A	KKNNVSVIQV	KKPSNNVSVIQV
482	RET	chr10	ENST00000340058	p.R180Q	FRIRENQPPGTF	FRIRENRPPGTF
483	RHO	chr3	ENST00000296271	p.Y30C	QYCLAEPWQF	QYYLAEPWQF
484	RIC1	chr9	ENST00000251879	p.T79A	YKQAEWRPDSAM	YKQAEWRPDSTM
485	RIC1	chr9	ENST00000251879	p.I774V	QRIMLPFHVNIY	QRIMLPFHINIY
486	RIOK3	chr18	ENST00000339486	p.V59A	VFPEAAVAEGPF	VFPEVAVAEGPF
487	RNF126	chr19	ENST00000292363	p.N248S	RQLPCSHLF	RQLPCNHLF
488	RNF208	chr9	ENST00000391553	p.M33T	MEATKIVHPEKF	MEAMKIVHPEKF
489	RNF26	chr11	ENST00000311413	p.S125G	VVSGGHALLRQA	VVSSGHALLRQA
490	RNPEP	chr1	ENST00000295640	p.T309A	FVAPCLLAGDRS	FVTPCLLAGDRS
491	ROR1	chr1	ENST00000371079	p.V145A	KEVVSSTGALFV	KEVVSSTGVLFV
492	RPAP2	chr1	ENST00000610020	p.A85T	RFITPTHYSDVV	RFITPAHYSDVV
493	RPS6KA4	chr11	ENST00000334205	p.L531I	VHRDIKPENILY	VHRDLKPENILY
494	RPS6KB1	chr17	ENST00000225577	p.V96A	FELLRALGKGGY	FELLRVLGKGGY
495	RPS6KB1	chr17	ENST00000225577	p.D393N	TRQTPVNSPDDS	TRQTPVDSPDDS
496	RRBP1	chr20	ENST00000246043	p.E1222D	YAKDVAGLRQLL	YAKEVAGLRQLL
497	RYR1	chr19	ENST00000355481	p.M4985T	EHNLANYTFFLM	EHNLANYMFFLM
498	RYR1	chr19	ENST00000355481	p.A4295P	GATARVVAPAGR	GATARVVAAAGR
499	RYR2	chr1	ENST00000366574	p.N3085D	QFTHTRDQPKGV	QFTHTRNQPKGV
500	SAP18	chr13	ENST00000382533	p.V70A	FSRGNAPSSELQ	FSRGNVPSSELQ
501	SASS6	chr1	ENST00000287482	p.V324A	EKHVNQLQTKAA	EKHVNQLQTKVA
502	SCN11A	chr3	ENST00000668754	p.E415D	EIDAKEKMFQEA	EIEAKEKMFQEA
503	SCN2A	chr2	ENST00000283256	p.R684W	RWSSSYHVSMDL	RRSSSYHVSMDL
504	SCN4A	chr17	ENST00000435607	p.A459T	NEATLTEDKEKE	NEATLAEDKEKE
505	SCUBE2	chr11	ENST00000450649	p.Y726C	GNSRFQVPCV	GNSRFQVPYV
506	SEL1L	chr14	ENST00000336735	p.Y299N	LGNRYWAGIGVL	LGYRYWAGIGVL
507	SELENOS	chr15	ENST00000398226	p.Y45C	FSCILLCVVFQK	FSCILLYVVFQK
508	SELPLG	chr12	ENST00000550948	p.Y51H	YEYLDHDFLPET	YEYLDYDFLPET
509	SELPLG	chr12	ENST00000550948	p.T254A	AQTAPLAAM	AQTTPLAAM
510	SEMA6C	chr1	ENST00000341697	p.W103R	TRRSQDVENCAV	TWRSQDVENCAV
511	SEPHS1	chr10	ENST00000327347	p.E374K	ARIIDKPRIIKV	ARIIDKPRIIEV
512	SEPTIN7	chr7	ENST00000432293	p.R52H	EAQHKELEEKHR	EAQHKELEEKRR
513	SPINC1	chr1	ENST00000367698	p.M283V	SVMYQEGKFRYR	SMMYQEGKFRYR
514	STAD2	chr2	ENST00000313349	p.Q147R	TFCTSRAMQPTA	TFCTSQAMQPTA
515	SEZ6L	chr22	ENST00000248933	p.A192V	WLDRKESVVPTT	WLDRKESAVPTT
516	SHC4	chr15	ENST00000332408	p.R432G	EQSGAIGNVHPR	EQSRAIGNVHPR
517	SHA6	chr17	ENST00000409168	p.A456T	NSYTTLGQSQTA	NSYATLGQSQTA
518	SHKBP1	chr19	ENST00000291842	p.G310R	LIATSHTGRIRV	LIATSHTGRIGV
519	SLC12A4	chr16	ENST00000316341	p.L558P	TWAPLLTALI	TWALLLTALI
520	SLC16A4	chr1	ENST00000369779	p.L178H	AHILFGAIALNL	ALILFGAIALNL
521	SLC22A4	chr5	ENST00000200652	p.A310T	NIAVPTVIFDSV	NIAVPAVIFDSV
522	SLC22A5	chr5	ENST00000245407	p.I551T	MLKDGQERPTTL	MLKDGQERPTIL
523	SLC25A3	chr12	ENST00000188376	p.E238D	FACFDRTVEALY	FACFERTVEALY
524	SLC26A6	chr3	ENST00000358747	p.L650I	FHSIILDLGALS	FHSLILDLGALS
525	SLC26A9	chr1	ENST00000340781	p.M588T	QRRSLFTKTKTV	QRRSLFMKTKTV
526	SLC2A4RG	chr20	ENST00000266077	p.V363M	CRKMYGMERRDL	CRKVYGMERRDL
527	SLC30A10	chr1	ENST00000366926	p.V285A	QCYIDPSLTALM	QCYIDPSLTVLM
528	SLC30A7	chr1	ENST00000357650	p.V237A	QGAFLHILADTL	QGVFLHILADTL

Number	HUGO Symbol	Chromosome	Transcript ID	HGVSp	MT Epitope Seq	WT Epitope Seq
529	SLC9A1	chr1	ENST00000263980	p.H120R	GFRVIPTISSIV	GFHVIPTISSIV
530	SMARCC2	chr12	ENST00000267064	p.I912T	FEELETTMDRER	FEELETIMDRER
531	SMG8	chr17	ENST00000300917	p.S476T	ELTTKILSSIKV	ELTSKILSSIKV
532	SMIM29	chr6	ENST00000636500	p.Y44C	RWCMAGRVATST	RWYMAGRVATST
533	SNTA1	chr20	ENST00000217381	p.M219I	HMSLKIAYVSKR	HMSLKMAYVSKR
534	SON	chr21	ENST00000300278	p.R1986H	RTPSRRSHTPSR	RTPSRRSRTPSR
535	SORCS3	chr10	ENST00000369701	p.R678H	FSLHSEWQLVKV	FSLRSEWQLVKV
536	SPAG5	chr17	ENST00000321765	p.W645R	LQQDRRSMQLDY	LQQDWRSMQLDY
537	SPDYE1	chr7	ENST00000258704	p.Q319R	VSPEELEEIRAY	VSPEELEEIQAY
538	SPOP	chr17	ENST00000393328	p.G49D	NFSFCREEMDEV	NFSFCREEMGEV
539	SPPL2A	chr15	ENST00000261854	p.A12V	QRRLSPAGVL	QRRLSPAGAALL
540	SPSB1	chr1	ENST00000328089	p.R36Q	CKPTQLDLLLDM	CKPTRLDLLLDM
541	SPSB2	chr12	ENST00000523102	p.S126N	GNNSESWGWDIG	GSNSESWGWDIG
542	SPTA1	chr1	ENST00000643759	p.P339H	SHPSDAHQIQEM	SHPSDAPQIQEM
543	SPTAN1	chr9	ENST00000358161	p.M988T	EVTTKKGDILTL	EVTMKKGDILTL
544	SPTBN5	chr15	ENST00000320955	p.A2200V	FEAEVQVHEEVM	FEAEVQAHEEVM
545	SRPRA	chr11	ENST00000332118	p.R401H	ILQPQHRVDMLR	ILQPQRRVDMLR
546	SSC5D	chr19	ENST00000389623	p.S1396P	SRPSTATSM	SRSSTATSM
547	SSH2	chr17	ENST00000269033	p.Y411C	MKECGWNLDRAY	MKEYGWNLDRAY
548	SSX3	chrX	ENST00000298396	p.S155C	INMICGPKRGEH	INMISGPKRGEH
549	STEAP3	chr2	ENST00000393106	p.M185T	MATGFMPVDM	MAMGFMPVDM
550	STRBP	chr9	ENST00000348403	p.M15V	DRHVVVKHSTIY	DRHVMVKHSTIY
551	SUCO	chr1	ENST00000263688	p.V1000A	LSATAAELKREV	LSATVAELKREV
552	SUPT6H	chr17	ENST00000314616	p.E1406K	FKDLDEIVARYV	FEDLDEIVARYV
553	SUSD1	chr9	ENST00000374264	p.R246Q	GNPPEMQHAILV	GNPPEMRHAILV
554	SV2A	chr1	ENST00000369146	p.A653D	FGNSESAMIDLL	FGNSESAMIALL
555	SYNE1	chr6	ENST00000367251	p.R1124C	LRRYCQEVFGCV	LRRYCQEVFGRV
556	TAF6L	chr11	ENST00000294168	p.M600V	RYVQKLPVI	RYVQKLPMI
557	TANC2	chr17	ENST00000424789	p.R1287Q	FQELKVSLLLNL	FRELKVSLLLNL
558	TATDN2	chr3	ENST00000287652	p.C30S	RKRSCLREPSDV	RKRSCLREPCDV
559	TBC1D9B	chr5	ENST00000356834	p.Y857C	LEQCRIDASQFR	LEQYRIDASQFR
560	TBKBP1	chr17	ENST00000361722	p.A297V	VYTELTEEL	AYTELTEEL
561	TBL3	chr16	ENST00000332704	p.V608I	LRFCITWNTNSR	LRFCVTWNTNSR
562	TCF25	chr16	ENST00000263346	p.V546A	NRRKALYQRAPR	NRRKVLYQRAPR
563	TDRD7	chr9	ENST00000355295	p.R830C	KNFPDPHCSINR	KNFPDPHRSINR
564	TEAD3	chr6	ENST00000338863	p.R85W	YIKLRTGKTWTR	YIKLRTGKTRTR
565	TESK2	chr1	ENST00000372084	p.S448L	LLSDGPPPR	SLSDGPPPR
566	TET3	chr2	ENST00000409262	p.E1564G	KLWNPMKGEGGR	KLWNPMKGEEGR
567	TGM2	chr20	ENST00000361475	p.R651K	EAGEEVKVKMDL	EAGEEVKVRMDL
568	TGM2	chr20	ENST00000361475	p.V604M	EPKQKRKLMAEV	EPKQKRKLVAEV
569	TGM5	chr15	ENST00000220420	p.L610Q	EKIQVNKIITLS	EKILVNKIITLS
570	THEM5	chr1	ENST00000368817	p.P195H	FKNLIHVDSLVV	FKNLIPVDSLVV
571	THOC1	chr18	ENST00000261600	p.A90T	TTSTPFVLLGDV	TASTPFVLLGDV
572	TICRR	chr15	ENST00000560985	p.E1803D	REDSEVSKSKDG	REDSEVSKSKEG
573	TIGD6	chr5	ENST00000296736	p.T43A	ITPSALSTF	ITPSTLSTF
574	TIMM44	chr19	ENST00000270538	p.D238E	KESKWYQQWKDF	KDSKWYQQWKDF
575	TJP2	chr9	ENST00000348208	p.R689C	DFWRMCGQRSGV	DFWRMRGQRSGV
576	TLN2	chr15	ENST00000472902	p.A226T	VGGMVDAITEAM	VGGMVDAIAEAM
577	TMCO4	chr1	ENST00000294543	p.T30M	LPMGRELTEANR	LPTGRELTEANR
578	TMEM145	chr19	ENST00000673187	p.S327L	YELPAGYGLIGL	YESPAGYGLIGL
579	TMEM171	chr5	ENST00000454765	p.V31I	IFCFFVFGAILL	IFCFFVFGAVLL
580	TMEM201	chr1	ENST00000340305	p.T23M	GVMACAAAGVLL	GVTACAAAGVLL
581	TMEM259	chr19	ENST00000333175	p.Y209H	KVWPQDEHIVEY	KVWPQDEYIVEY

Number	HUGO Symbol	Chromosome	Transcript ID	HGVSp	MT Epitope Seq	WT Epitope Seq
582	TMEM26	chr10	ENST00000399298	p.Y36H	RHWLLALLNLLL	RYWLLALLNLLL
583	TMPRSS12	chr12	ENST00000398458	p.V106I	KYGRVLVHI	KYGRVLVHV
584	TMX2	chr11	ENST00000278422	p.Q262R	NLNELYRRAKKL	NLNELYQRAKKL
585	TNFAIP2	chr14	ENST00000333007	p.L447M	QNWQVPQDTMSL	QNWQVPQDTLSL
586	TOMM40L	chr1	ENST00000367987	p.P27H	RREEPLPNH	RREEPLPNP
587	TOPBP1	chr3	ENST00000260810	p.K959E	YESVKERGVHIV	YKSVKERGVHIV
588	TP63	chr3	ENST00000264731	p.A139T	YNTDHTQNSVTA	YNTDHAQNSVTA
589	TRANK1	chr3	ENST00000645898	p.Y1688H	LHTAITRARVNL	LYTAITRARVNL
590	TRHDE	chr12	ENST00000261180	p.R770H	ALYPLDKLLDHM	ALYPLDKLLDRM
591	TRIM14	chr9	ENST00000341469	p.Y341C	WVGAACASLRRR	WVGAAYASLRRR
592	TRNAU1AP	chr1	ENST00000373830	p.G8V	ASLWMVDLEPYM	ASLWMGDLEPYM
593	WM6	chr9	ENST00000360774	p.V1847E	IYTFNQEKPQTI	IYTFNQVKPQTI
594	WV1	chr17	ENST00000310522	p.V288A	ARDSVGNTALHA	ARDSVGNTVLHA
595	TTC13	chr1	ENST00000366661	p.H440R	SRYLRAHLDTPL	SRYLHAHLDTPL
596	TTC30A	chr2	ENST00000355689	p.H640Y	IEQPLEEERMYV	IEQPLEEERMHV
597	TTLL7	chr1	ENST00000474957	p.L448V	NVYRIFNRVFNR	NLYRIFNRVFNR
598	UBQLN4	chr1	ENST00000368309	p.P12S	GAEWSIRVTV	GAEWPIRVTV
599	UBR5	chr8	ENST00000220959	p.L1398S	CTADILLSDTLL	CTADILLLDTLL
600	ULBP1	chr6	ENST00000229708	p.E51G	PKSRPEPQWCGV	PKSRPEPQWCEV
601	UMODL1	chr21	ENST00000400421	p.A59T	GNTSTTVSRLLL	GNASTTVSRLLL
602	UMODL1	chr21	ENST00000468982	p.R91H	WEHLHHSVAAAT	WERLHHSVAAAT
603	UNC13B	chr9	ENST00000378495	p.S892F	ERLQDLKFTVDL	ERLQDLKSTVDL
604	UNC5B	chr10	ENST00000335350	p.V243I	RRSTTATVIIYV	RRSTTATVIVYV
605	UNCX	chr7	ENST00000316333	p.N176S	GRPAHSSHPTTC	GRPAHNSHPTTC
606	UQCRC2	chr16	ENST00000268379	p.H310R	GSNTTSHLRQAV	GSNTTSHLHQAV
607	UQCRHL	chr1	ENST00000483273	p.R49Q	AREQLELYDEHV	ARERLELYDEHV
608	USH2A	chr1	ENST00000307340	p.A416V	QYFVRNCGAF	QYFARNCGAF
609	USP14	chr18	ENST00000261601	p.V89I	EPSAKTVFIEDM	EPSAKTVFVEDM
610	USP18	chr22	ENST00000215794	p.R119W	SRQKAVWPL	SRQKAVRPL
611	USP19	chr3	ENST00000398888	p.V682A	KQKALPVFYFAR	KQKVLPVFYFAR
612	USP36	chr17	ENST00000449938	p.V271A	YLDAALEIRQAA	YLDVEIRQAA
613	UST	chr6	ENST00000367463	p.E160A	IKNISTAAQPYL	IKNISTAEQPYL
614	VD	chr19	ENST00000245932	p.Q31L	RWLPAGTGPLAF	RWLPAGTGPQAF
615	VGLL2	chr6	ENST00000326274	p.R307H	ARHYSLCGASLL	ARRYSLCGASLL
616	VPS13A	chr9	ENST00000360280	p.P1902H	SDSFSVLNIHMA	SDSFSVLNIPMA
617	VPS8	chr3	ENST00000287546	p.L455P	VYNSSHFKSPAT	VYNSSHFKSLAT
618	VSIG10	chr12	ENST00000359236	p.Y251C	CRWDGGCPDPDF	CRWDGGYPDPDF
619	VWDE	chr7	ENST00000452576	p.L694S	NSFLQEKKHINL	NLFLQEKKHINL
620	VXN	chr8	ENST00000305454	p.A79V	DRRRFGRLQTVR	DRRRFGRLQTAR
621	WDFY2	chr13	ENST00000298125	p.N368S	HSIVHVHFDATR	HNIVHVHFDATR
622	WDR18	chr19	ENST00000585809	p.F136L	SHLISGGKDCLV	SHFISGGKDCLV
623	WDR24	chr16	ENST00000293883	p.Q685R	YTSYIDLLRRFR	YTSYIDLLQRFR
624	WDR81	chr17	ENST00000409644	p.L1877M	FDMYGSEVVTGT	FDLYGSEVVTGT
625	WDR83	chr19	ENST00000418543	p.D212G	CTLVSSLGSTLR	CTLVSSLDSTLR
626	WIPI2	chr7	ENST00000288828	p.A174E	LEYPGSATIGEV	LAYPGSATIGEV
627	XDH	chr2	ENST00000379416	p.R1176C	HKNLCTDIVMDV	HKNLRTDIVMDV
628	XIAP	chrX	ENST00000371199	p.G232D	NCFFVLDRNLNI	NCFFVLGRNLNI
629	XPNPEP1	chr10	ENST00000322238	p.G42D	GVETDDRMPPKV	GVETDGRMPPKV
630	YBX1	chr1	ENST00000436427	p.P73L	GPAPRACLGAPR	GPAPRACPGAPR
631	ZBTB21	chr21	ENST00000310826	p.K773E	CEYEKLTCLECM	CEYKKLTCLECM
632	ZBTB34	chr9	ENST00000319119	p.V135I	HSKISIGDVDSV	HSKISVGDVDSV
633	ZCCHC7	chr9	ENST00000534928	p.K459T	ETKADRHREV	EKHRKADRHREV
634	ZFP14	chr19	ENST00000270001	p.L411H	SSYSQHHQSI	SSYSQLISHQSI

Number	HUGO Symbol	Chromosome	Transcript ID	HGVSp	MT Epitope Seq	WT Epitope Seq
635	ZFP28	chr19	ENST00000301318	p.S760N	ECNVCGKAFSHR	ECSVCGKAFSHR
636	ZGLP1	chr19	ENST00000403903	p.R128W	SAPPQRRPW	SAPPQRRPR
637	ZMYM6	chr1	ENST00000357182	p.A219T	QNVVHGLCSDTC	QNVVHGLCSDAC
638	ZMYM6	chr1	ENST00000357182	p.R402H	IHGSAAASL	IRGSAAASL
639	ZNF213	chr16	ENST00000396878	p.N234S	KRESSRNTTLGF	KRENSRNTTLGF
640	ZNF236	chr18	ENST00000253159	p.A261T	ACAFCPATF	ACAFCPAAF
641	ZNF438	chr10	ENST00000331737	p.D2Y	MYSESNIPSGTI	MDSESNIPSGTI
642	ZNF469	chr16	ENST00000565624	p.F705S	EVGRGGLQGSPR	EVGRGGLQGFPR
643	ZNF500	chr16	ENST00000589422	p.P44H	DHSPETFRQLFR	DPSPETFRQLFR
644	ZNF611	chr19	ENST00000595798	p.C418Y	EYGKTFGQNSDL	ECGKTFGQNSDL
645	ZNF628	chr19	ENST00000391718	p.T686M	LPHLQAMLSLEV	LPHLQATLSLEV
646	ZNF7	chr8	ENST00000446747	p.V43A	YREAMLENHSSV	YREVMLENHSSV
647	ZNF791	chr19	ENST00000343325	p.R111H	FMHLSSLTRHMR	FMRLSSLTRHMR
648	ZNRF3	chr22	ENST00000406323	p.T285A	TRASMDSHGNPV	TRTSMDSHGNPV
649	ZNRF4	chr19	ENST00000222033	p.R177H	TFDLKVLNAQHA	TFDLKVLNAQRA
650	ZSWIM8	chr10	ENST00000603187	p.R719Q	NPNEIQQALVQC	NPNEIQRALVQC
651	ZSWIM9	chr19	ENST00000328759	p.A312T	VRQLLPCTRVQI	VRQLLPCARVQI
652	ZZEF1	chr17	ENST00000381638	p.C162Y	AYSLVPGFTDIF	ACSLVPGFTDIF
