# Supplementary material for: Stimulating T cell responses against patient-derived breast cancer cells with neoantigen peptide-loaded peripheral blood mononuclear cells
Source: Cancer Immunol Immunother. 2024 Feb 13;73(3):43. doi: 10.1007/s00262-024-03627-3 (PMC10864427; doi:10.1007/s00262-024-03627-3)
Supplement: Supplementary file 9 — (PDF 80 kb) [file 262_2024_3627_MOESM9_ESM.pdf]

Supplementary Table S5a. List of candidate neoantigens of PC-B-142CA

No.	Gene Name	HLA Allele	HGVSp	MT Epitope Seq	WT Epitope Seq	Best IC_{50} MT algorithm	Best IC_{50} MT (nM)	IC_{50} corresponding WT (nM)	Corresponding Fold Change
1	ADGRL1	HLA-A*11:01	p.E274K	KTDIDLAVDK	KTDIDLAVDE	NetMHC	38.97	24602.85	631.33
2	AHCYL2	HLA-A*11:01	p.E555K	ALIELYNAPK	ALIELYNAPE	MHCflurry	19.92	5064.81	254.31
3	WNK4	HLA-B*54:01	p.P87A	SPAPDPPDPA	SPAPDPPDPP	MHCnuggetsI	52.65	11855.12	225.15
4	ATP5F1B	HLA-A*11:01	p.E448K	AILGMDELSK	AILGMDELSE	MHCflurry	20.93	3508.97	167.68
5	PARP1	HLA-A*11:01	p.E619K	AIEHFMKLYK	AIEHFMKLYE	MHCflurry	13.35	2110.72	158.16
6	HIST1H3C	HLA-A*11:01	p.E98K	AVMALQEACK	AVMALQEACE	MHCflurry	15.91	2514.79	158.03
7	CRLF3	HLA-A*11:01	p.E72K	GTLGKLLDK	GTLGKLLDE	MHCflurry	18.00	2681.04	148.94
8	TBC1D17	HLA-A*11:01	p.E76K	SSGGDSCASK	SSGGDSCASE	MHCflurry	57.47	8406.15	146.26
9	TAX1BP1	HLA-B*54:01	p.P724A	CPMCSEQFPA	CPMCSEQFPP	MHCnuggetsI	5.95	790.24	132.87
10	PPP1R9B	HLA-A*11:01	p.E216K	TVSQLSAVFK	TVSQLSAVFE	MHCflurry	10.18	725.24	71.27
11	ATIC	HLA-A*11:01	p.E347K	VSDGIIAPGYK	VSDGIIAPGYE	SMM	50.13	2512.47	50.12
12	HK1	HLA-A*11:01	p.P179V	SVVEGADVVK	SGVEGADVVK	MHCflurry	29.39	1130.03	38.45
13	SEC14L2	HLA-A*11:01	p.R43Q	LQARSFDLQK	LRARSFDLQK	MHCnuggetsI	118.43	3646.32	30.79
14	TFIP11	HLA-A*24:02	p.S691L	WYLGWKSMFL	WYLGWKSMFS	MHCflurry	45.88	1397.25	30.45
15	IFT88	HLA-A*11:01	p.D684H	HTYKDTHRK	DTYKDTHRK	MHCflurry	21.60	178.87	8.28
16	G3BP1	HLA-A*11:01	p.P124L	KLYVHNDIFR	KFYVHNDIFR	MHCflurry	94.68	681.65	7.20
17	FLNA	HLA-A*24:02	p.E2056Q	TFQPAEFII	TFEPAEFII	MHCflurry	122.91	690.21	5.62
18	GLRX3	HLA-A*11:01	p.S94P	FLFFKNFQK	FLFFKNSQK	NetMHC	24.43	105.72	4.33
19	MAP1LC3B	HLA-A*11:01	p.E18Q	RVQDVRLIR	RVEDVRLIR	MHCnuggetsI	61.05	264.15	4.33
20	CC2D1A	HLA-A*24:02	p.E639Q	RFQQRTFSVI	RFEQRTFSVI	MHCflurry	34.76	115.55	3.32
21	FCSK	HLA-A*24:02	p.E208Q	YYQGTEAQI	YYQGTEAEI	MHCnuggetsI	37.24	104.02	2.79
22	TNPO3	HLA-A*11:01	p.L183V	SVLMTCVEK	SLLMTCVEK	MHCflurry	13.58	33.33	2.45
23	DIAPH1	HLA-C*07:02	p.S28L	GRSPDELPL	GRSPDELPS	SMMPMBEC	18.73	42.33	2.26
24	SHPK	HLA-B*54:01	p.Q80H	LPRPHLRSV	LPRPQLRSV	NetMHC	19.52	43.17	2.21
25	SBF2	HLA-A*11:01	p.E987Q	QVSPEVVEIFK	EVSPEVVEIFK	MHCnuggetsI	12.75	25.02	1.96
26	FAM13B	HLA-A*11:01	p.L712P	ASFQKSLLYY	ASLQKSLLYY	MHCnuggetsI	10.57	18.93	1.79
27	SMG7	HLA-C*07:02	p.M1107I	IMHPGPSAL	MMHPGPSAL	SMM	96.20	172.25	1.79
28	SERHL2	HLA-A*11:01	p.S232P	RELCAHFIRK	RELCAHSIRK	NetMHC	64.01	111.44	1.74
29	MAP4	HLA-A*11:01	p.D931N	ATNTSAPNLK	ATNTSAPDLK	MHCnuggetsI	17.47	29.94	1.71
30	EAF2	HLA-C*07:02	p.D11H	HRRERVLKL	DRRERVLKL	SMM	18.93	32.00	1.69
31	CEP57	HLA-A*11:01	p.R30W	SMVWHSSSPY	SMVRHSSSPY	MHCflurry	150.33	239.70	1.59
32	PATZ1	HLA-A*11:01	p.P298D	GILPCGLCDK	GILPCGLCGK	MHCnuggetsI	21.21	32.21	1.52
33	BHLHE41	HLA-A*11:01	p.P81A	KLTTLAHLEK	KLTTLGHLEK	MHCnuggetsI	53.03	72.33	1.36
34	RUFY2	HLA-C*07:02	p.H153D	FYEYDALMM	FYEYHALMM	MHCnuggetsI	36.06	49.08	1.36
35	USP31	HLA-C*07:02	p.S377Y	YYDGFHRYF	YYDGFHRSF	MHCnuggetsI	6.92	9.16	1.32
36	ACP2	HLA-A*24:02	p.E314Q	PYASCHIFQL	PYASCHIFEL	MHCflurry	58.54	73.90	1.26
37	TFIP11	HLA-A*11:01	p.P658L	GLLEKHLFPK	GLLEKHFFPK	MHCnuggetsI	20.12	25.34	1.26

No.	Gene Name	HLA Allele	HGVSp	MT Epitope Seq	WT Epitope Seq	Best IC_{50} MT algorithm	Best IC_{50} MT (nM)	IC_{50} corresponding WT (nM)	Corresponding Fold Change
38	PICALM	HLA-B*54:01	p.Q413E	HPMSTASEVA	HPMSTASQVA	SMM	8.72	10.82	1.24
39	RABGAP1L	HLA-A*11:01	p.E320K	ALMNKIQAAK	ALMNEIQAAK	MHCflurry	23.43	28.54	1.22
40	NAGA	HLA-A*11:01	p.M302I	ILQNPLIIK	ILQNPLMIK	MHCnuggetsI	37.01	45.00	1.22
41	LRRC41	HLA-A*11:01	p.E350Q	ATSHQAPGTK	ATSHEAPGTK	MHCflurry	25.72	30.01	1.17
42	HSPA2	HLA-A*24:02	p.E107Q	QYKGETKTFF	EYKGETKTFF	SMM	46.85	54.29	1.16
43	ADK	HLA-A*11:01	p.E340Q	RTGCTFPQK	RTGCTFPEK	MHCflurry	18.15	20.91	1.15
44	RMDN1	HLA-B*54:01	p.R226T	MPWYQTRIA	MPWYQRRIA	MHCnuggetsI	1.87	2.13	1.14
45	MAFG	HLA-A*11:01	p.V70M	ASCRMKRVTQK	ASCRVKRVTQK	MHCflurry	44.90	50.24	1.12
46	MAZ	HLA-A*11:01	p.E181Q	AVAPVASALQK	AVAPVASALEK	MHCflurry	18.73	20.95	1.12
47	AASDH	HLA-A*11:01	p.L144R	LVLFRRHWK	LVLFRLHWK	MHCnuggetsI	25.00	27.52	1.10
48	ACTR5	HLA-A*11:01	p.L476V	LQYIVDRYPK	LQYILDRYPK	MHCflurry	21.35	22.78	1.07
49	POLD2	HLA-A*24:02	p.P403L	VYLCGNTPSF	VYFCGNTPSF	MHCnuggetsI	17.50	18.54	1.06
50	ZNF326	HLA-A*24:02	p.S155P	NYFSYSSF	NYSSYSSF	MHCflurry	17.20	17.73	1.03
51	RIF1	HLA-C*07:02	p.E1447K	RRKEKEKPL	RRKEEEKPL	MHCnuggetsI	14.63	15.05	1.03
52	TDRD7	HLA-A*11:01	p.E1089K	MSKYLIELSK	MSEYLIELSK	NetMHC	36.04	36.44	1.01
