# Supplementary material for: Stimulating T cell responses against patient-derived breast cancer cells with neoantigen peptide-loaded peripheral blood mononuclear cells
Source: Cancer Immunol Immunother. 2024 Feb 13;73(3):43. doi: 10.1007/s00262-024-03627-3 (PMC10864427; doi:10.1007/s00262-024-03627-3)
Supplement: Supplementary file 10 — (PDF 69 kb) [file 262_2024_3627_MOESM10_ESM.pdf]

Supplementary Table S5b. List of candidate neoantigens of PC-B-148CA

No.	Gene Name	HLA Allele	HGVSp	MT Epitope Seq	WT Epitope Seq	Best IC_{50} MT algorithm	Best IC_{50} MT (nM)	IC_{50} corresponding WT (nM)	Corresponding Fold Change
1	LSR	HLA-A*24:02	p.I158P	YYQGRRFTI	YYQGRRITI	MHCflurry	10.19	34.00	3.34
2	ALKBH6	HLA-A*24:02	p.V83M	RYMDKVSNLSLF	RYVDKVSNLSLF	MHCnuggetsI	8.18	22.60	2.76
3	GAA	HLA-C*07:02	p.I823T	LRAGYTIPL	LRAGYIIPL	MHCflurry	61.56	88.89	1.44
4	ELMO2	HLA-A*24:02	p.L289P	LYVFQVLTF	LYVLQVLTF	MHCflurry	10.19	11.10	1.09
