# Supplementary material for: Stimulating T cell responses against patient-derived breast cancer cells with neoantigen peptide-loaded peripheral blood mononuclear cells
Source: Cancer Immunol Immunother. 2024 Feb 13;73(3):43. doi: 10.1007/s00262-024-03627-3 (PMC10864427; doi:10.1007/s00262-024-03627-3)
Supplement: Supplementary file 11 — (PDF 78 kb) [file 262_2024_3627_MOESM11_ESM.pdf]

Supplementary Table S6. List of candidate neoantigens of PC-B-142 tissue

No.	Gene Name	HLA Allele	HGVSp	MT Epitope Seq	WT Epitope Seq	Best IC_{50} MT algorithm	Best IC_{50} MT (nM)	IC_{50} corresponding WT (nM)	Corresponding Fold Change
1	ADGRL1	HLA-A*11:01	p.E274K	KTDIDLAVDK	KTDIDLAVDE	NetMHC	38.97	24602.85	631.328
2	PARP1	HLA-A*11:01	p.E619K	AIEHFMKLYK	AIEHFMKLYE	MHCflurry	13.346	2110.722	158.156
3	TAX1BP1*	HLA-B*54:01	p.P724A	CPMCSEQFPA	CPMCSEQFPP	MHCnuggetsI	5.947	790.244	132.873
4	SEC14L2	HLA-A*11:01	p.R43Q	LQARSFDLQK	LRARSFDLQK	MHCnuggetsI	118.433	3646.316	30.788
5	IFT88	HLA-A*11:01	p.D684H	HTYKDTHRK	DTYKDTHRK	MHCflurry	21.599	178.872	8.282
6	G3BP1	HLA-A*11:01	p.F124L	KLYVHNDIFR	KFYVHNDIFR	MHCflurry	94.682	681.646	7.199
7	FLNA	HLA-A*24:02	p.E2056Q	TFQPAEFII	TFEPAEFII	MHCflurry	122.91	690.21	5.616
8	MAP1LC3B	HLA-A*11:01	p.E18Q	RVQDVRLIR	RVEDVRLIR	MHCnuggetsI	61.052	264.145	4.327
9	SHL2	HLA-A*11:01	p.S232F	RELCAHFIRK	RELCAHSIRK	NetMHC	64.01	111.44	1.741
10	MAP4	HLA-A*11:01	p.D931N	ATNTSAPNLK	ATNTSAPDLK	MHCnuggetsI	17.467	29.942	1.714
11	TBC1D19	HLA-C*07:02	p.E313Q	YYFVFQDYL	YYFVFEDYL	SMM	20.757	32.447	1.563
12	PATZ1	HLA-A*11:01	p.G298D	GILPCGLCDK	GILPCGLCGK	MHCnuggetsI	21.209	32.211	1.519
13	PAQR6	HLA-A*11:01	p.S14F	ASFSPPTCLK	ASSSPPTCLK	MHCflurry	6.969	9.312	1.336
14	RABGAP1L	HLA-A*11:01	p.E320K	ALMNKIQAAK	ALMNEIQAAK	MHCflurry	23.428	28.542	1.218
15	LRRC41	HLA-A*11:01	p.E350Q	ATSHQAPGTK	ATSHEAPGTK	MHCflurry	25.716	30.014	1.167
16	ADK	HLA-A*11:01	p.E340Q	RTGCTFPQK	RTGCTFPEK	MHCflurry	18.146	20.908	1.152
17	MAFG	HLA-A*11:01	p.V70M	ASCRMKRVTQK	ASCRVKRVTQK	MHCflurry	44.901	50.237	1.119
18	PEX1	HLA-A*11:01	p.E484Q	LVISQEEFIK	LVISEEEFIK	MHCflurry	25.656	27.836	1.085
19	ACTR5	HLA-A*11:01	p.L476V	LQYIVDRYPK	LQYILDRYPK	MHCflurry	21.345	22.778	1.067
20	POLD2	HLA-A*24:02	p.F403L	VYLCGNTPSF	VYFCGNTPSF	MHCnuggetsI	17.502	18.544	1.06
21	RIF1	HLA-C*07:02	p.E1447K	RRKEKEKPL	RRKEEEKPL	MHCnuggetsI	14.627	15.051	1.029

* Unselected peptide due to no healthy donor expressing HLA-B*54:01 is available
